# Would global warming bring an increase of invertebrate-associated cutaneous invasive fungal infections?

**DOI:** 10.1128/mbio.03447-24

**Published:** 2025-02-05

**Authors:** Dimitrios P. Kontoyiannis, Arturo Casadevall

**Affiliations:** 1 Department of Infectious Diseases, Infection Control and Employee Health, The University of Texas MD Anderson Cancer Center, Houston, Texas, USA; 2 Department of Molecular Microbiology and Immunology, Johns Hopkins Bloomberg School of Public Health, Baltimore, Maryland, USA; Vallabhbhai Patel Chest Institute, Delhi, India

**Keywords:** invertebrates, fungal infections, global warming

## Abstract

Invasive mold-associated cutaneous disease is a rare but potentially catastrophic consequence of trauma. However, invertebrate bites are not well recognized as a mechanism for the inoculation of fungi into subcutaneous tissue that can also result in severe infections. Invertebrates often carry fungi with human pathogenic potential as part of their microbiome, and bites break the skin, providing a conduit for them to penetrate subcutaneous tissues where the establishment of infection can produce serious skin and soft tissue fungal diseases. In this essay, we review the existing data for invertebrate bite-associated cutaneous invasive fungal infections (IBA-cIFIs) and consider the potential consequences of global warming on their epidemiology. Climate changes will be associated with changes in the range of invertebrates and adaptation of their associated microbes to warmer temperatures. Fungal adaptation to higher temperatures can defeat the mammalian protective barrier and be associated with both more and different IBA-cIFIs.

## OPINION/HYPOTHESIS

Climate change, including both global warming and its consequent associated natural disasters, is increasingly recognized as a precipitating factor for a variety of infectious diseases (1). These effects are multifactorial and dynamic and include changes in the range of known pathogens and the potential emergence of new pathogens through thermal adaptation (1, 2). In addition, climate change creates enormous stresses in host populations since heat stress affects physiology (3) that can translate into impaired human and animal immunity (4, 5). Furthermore, climate change alters the density and behavior of many vectors of infectious agents that, in turn, can result in an increase in various zoonotic and vector-born infections (1, 2). Whereas a variety of waterborne bacterial infections and insect-associated viral infections have been the focus of much attention, comparatively little attention has been given to fungal diseases. Consequently, the impact of global warming to the type, severity, and prevalence of fungal infections has been incompletely understood and focused on systemic mycoses, which often have a pulmonary source (6), or on emerging thermotolerant fungi (such as the multidrug-resistant yeasts *Candida auris* and *Rhodosporidiobulous fluvialis*) (7, 8).

Superficial and semi-invasive (dermatophytosis, chromoblastomycosis, mycetomas, and sporotrichosis) skin and soft tissue infections have been indirectly associated with global warming (9). In contrast, necrotizing soft issue mold infections have been sporadically described in the setting of natural disasters such as tsunamis, tornados, and earthquakes (6). Sporadic cases have also been described in association with traumatic injuries such as motorcycle accidents or war-related blast injuries (10, 11). A common denominator in many of these situations is injuries that allow the fungus to bypass integument defenses and inoculation of large fungal loads. Herein we wish to bring attention to the underappreciated association of severe necrotizing fungal infections of the skin with invertebrate bites.

**Fig 1:**
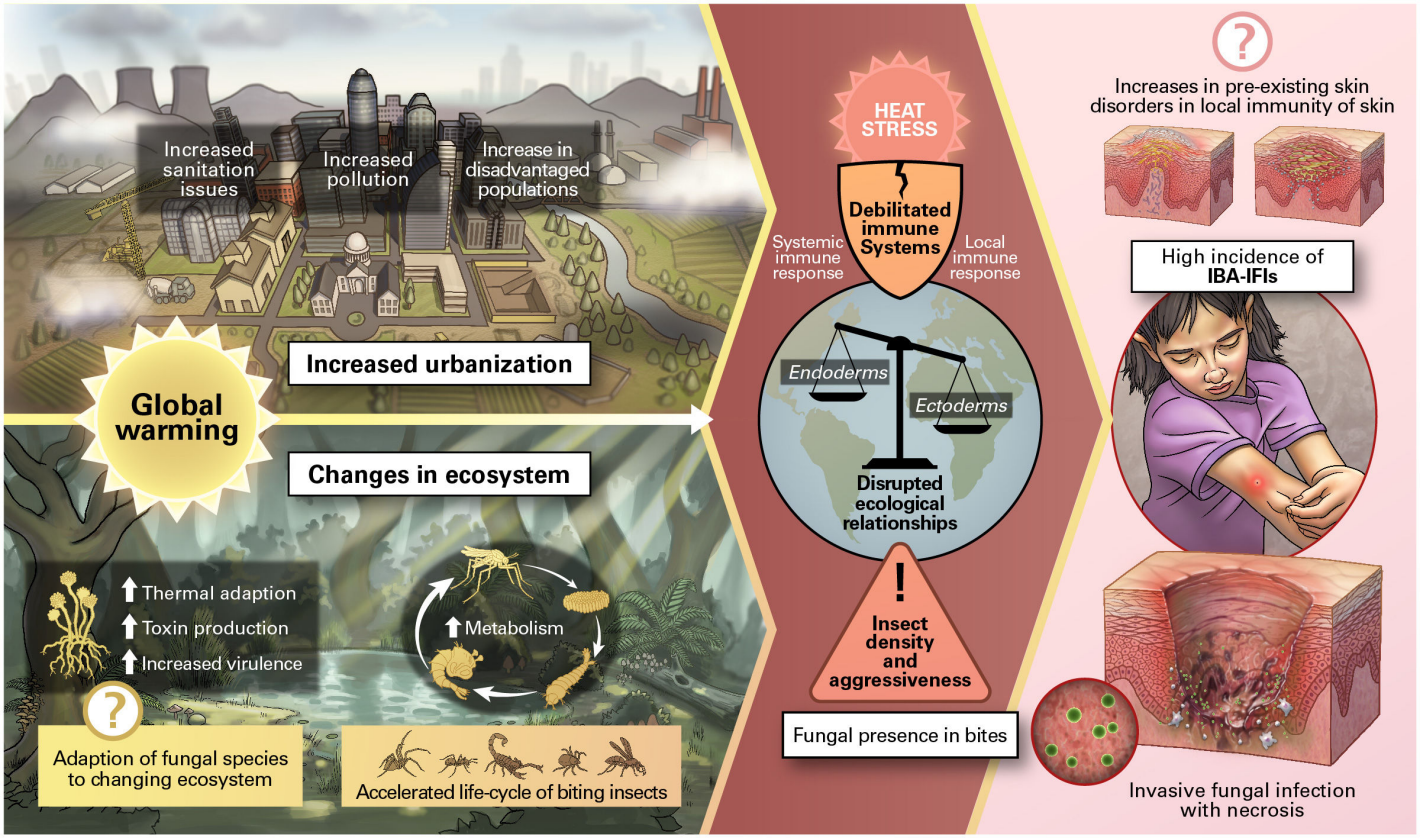
Proposed scheme for association of global warming, invertebrate bites, and cutaneous invasive fungal infections.

Invertebrates such as insects and spiders can carry, as part of their microbiome, fungi with human pathogenic potential (Tables 1 and 2, supplement). Invertebrate bites can pierce the skin, providing a mechanism for direct inoculation of pathogens into subcutaneous tissues. We hypothesize that inexorable increases in environmental temperatures could increase the frequency and severity of such infections through complex changes in arthropod vector density and behavior, fungal thermal tolerance, and increased virulence, coupled with changes in ecosystems associated with pollution and urbanization (Fig. 1). This is a unique risk factor and concern, as most invasive fungal diseases develop through inhalation, mucosal translocation from the gut, or major trauma, while invertebrate bites provide a different situation whereby fungal infections are associated with direct transdermal inoculation through bites.

**TABLE 1:** Published association of pathogenic fungi with invertebrate carriage^*i*^ ^*,**j*^

Fungi	Ants^*a*^	Aphids^*b*^	Bedbugs^*c*^	Bees^*d*^	Beetles^*e*^	Fleas^*f*^	Flies^*g*^	Lice^*h*^
*Saccharomyces*	Saccharomyces + ants	Saccharomyces + aphids	**Saccharomyces + bedbugs**	Saccharomyces + bees	Saccharomyces + beetles	**Saccharomyces + fleas**	Saccharomyces + flies	**Saccharomyces + lice**
*Candida*	Candida + ants	Candida + aphids	**Candida + bedbugs**	Candida + bees	Candida + beetles	**Candida + fleas**	Candida + flies	**Candida + lice**
*Aspergillus*	Aspergillus + ants	Aspergillus + aphids	Aspergillus + bedbugs	Aspergillus + bees	Aspergillus + beetles	**Aspergillus + fleas**	Aspergillus + flies	Aspergillus + lice
*Fusarium*	Aspergillus + ants	Fusarium + aphids	Fusarium + bedbugs	Fusarium + bees	Fusarium + beetles	**Fusarium + fleas**	Fusarium + flies	**Fusarium + lice**
*Penicillium*	Penicillium + ants	Penicillium + aphids	Penicillium + bedbugs	Penicillium + bees	Penicillium + beetles	Penicillium + fleas	Penicillium + flies	**Penicillium + lice**
*Trichoderma*	Trichoderma + ants	Trichoderma + aphids	Trichoderma + bedbugs	Trichoderma + bees	Trichoderma + beetles	**Trichoderma + fleas**	Trichoderma + flies	**Trichoderma + lice**
*Botrytis*	**Botrytis + ants**	Botrytis + aphids	Botrytis + bedbugs	Botrytis + bees	Botrytis + beetles	**Botrytis + fleas**	Botrytis + flies	**Botrytis + lice**
*Pichia*	**Pichia + ants**	**Pichia + aphids**	**Pichia + bedbugs**	Pichia + bees	Pichia + beetles	**Pichia + fleas**	Pichia + flies	**Pichia + lice**
*Cryptococcus*	Cryptococcus + ants	**Cryptococcus + aphids**	**Cryptococcus + bedbugs**	Cryptococcus + bees	Cryptococcus + beetles	**Cryptococcus + fleas**	Cryptococcus + flies	**Cryptococcus + lice**
*Alternaria*	Alternaria + ants	Alternaria + aphids	**Alternaria + bedbugs**	Alternaria + bees	Alternaria + beetles	**Alternaria + fleas**	Alternaria + flies	**Alternaria + lice**
*Phytophthora*	Phytophthora + ants	Phytophthora + aphids	**Phytophthora + bedbugs**	Phytophthora + bees	Phytophthora + beetles	**Phytophthora + fleas**	**Phytophthora + flies**	**Phytophthora + lice**
*Rhizopus*	**Rhizopus + ants**	**Rhizopus + aphids**	Rhizopus + bedbugs	Rhizopus + bees	Rhizopus + beetles	**Rhizopus + fleas**	Rhizopus + flies	**Rhizopus + lice**
*Phanerochaete*	**Phanerochaete + ants**	**Phanerochaete + aphids**	**Phanerochaete + bedbugs**	Phanerochaete + bees	Phanerochaete + beetles	**Phanerochaete + fleas**	**Phanerochaete + flies**	**Phanerochaete + lice**
*Colletotrichum*	Colletotrichum + ants	Colletotrichum + aphids	**Colletotrichum + bedbugs**	Colletotrichum + bees	Colletotrichum + beetles	**Colletotrichum + fleas**	Colletotrichum + flies	**Colletotrichum + lice**
*Trametes*	**Trametes + ants**	**Trametes + aphids**	**Trametes + bedbugs**	Trametes + bees	Trametes + beetles	**Trametes + fleas**	**Trametes *+* flies**	**Trametes + lice**
*Rhizoctonia*	**Rhizoctonia + ants**	Rhizoctonia + aphids	**Rhizoctonia + bedbugs**	**Rhizoctonia + bees**	**Rhizoctonia + beetles**	**Rhizoctonia + fleas**	Rhizoctonia + flies	**Rhizoctonia + lice**
*Pleurotus*	**Pleurotus + ants**	**Pleurotus + aphids**	**Pleurotus + bedbugs**	**Pleurotus + bees**	Pleurotus + beetles	**Pleurotus + fleas**	Pleurotus + flies	**Pleurotus + lice**
*Ganoderma*	**Ganoderma + ants**	**Ganoderma + aphids**	**Ganoderma + bedbugs**	**Ganoderma + bees**	Ganoderma + beetles	**Ganoderma + fleas**	**Ganoderma + flies**	**Ganoderma + lice**
*Neurospora*	**Neurospora + ants**	**Neurospora + aphids**	**Neurospora + bedbugs**	Neurospora + bees	**Neurospora + beetles**	**Neurospora + fleas**	**Neurospora + flies**	**Neurospora + lice**
*Cladosporium*	Cladosporium + ants	Cladosporium + aphids	**Cladosporium + bedbugs**	Cladosporium + bees	Cladosporium + beetles	**Cladosporium + fleas**	Cladosporium + flies	**Cladosporium + lice**
*Yarrowia*	Yarrowia + ants	**Yarrowia + aphids**	**Yarrowia + bedbugs**	**Yarrowia + bees**	Yarrowia + beetles	**Yarrowia + fleas**	**Yarrowia + flies**	**Yarrowia + lice**
*Agaricus*	**Agaricus + ants**	**Agaricus + aphids**	**Agaricus + bedbugs**	Agaricus + bees	Agaricus + beetles	**Agaricus + fleas**	Agaricus + flies	**Agaricus + lice**
*Kluyveromyces*	**Kluyveromyces + ants**	**Kluyveromyces + aphids**	**Kluyveromyces + bedbugs**	**Kluyveromyces + bees**	Kluyveromyces + beetles	**Kluyveromyces + fleas**	Kluyveromyces + flies	**Kluyveromyces + lice**
*Mucor*	Mucor + ants	**Mucor + aphids**	**Mucor + bedbugs**	Mucor + bees	Mucor + beetles	**Mucor + fleas**	Mucor + flies	**Mucor + lice**
*Verticillium*	**Verticillium + ants**	Verticillium + aphids	**Verticillium + bedbugs**	Verticillium + bees	Verticillium + beetles	**Verticillium + fleas**	Verticillium + flies	**Verticillium + lice**
*Sclerotinia*	**Sclerotinia + ants**	Sclerotinia + aphids	**Sclerotinia + bedbugs**	Sclerotinia + bees	Sclerotinia + beetles	**Sclerotinia + fleas**	**Sclerotinia + flies**	**Sclerotinia + lice**
*Rhodotorula*	Rhodotorula + ants	**Rhodotorula + aphids**	**Rhodotorula + bedbugs**	Rhodotorula + bees	Rhodotorula + beetles	**Rhodotorula + fleas**	Rhodotorula + flies	**Rhodotorula + lice**
*Beauveria*	Beauveria + ants	Beauveria + aphids	Beauveria + bedbugs	Beauveria + bees	Beauveria + beetles	Beauveria + fleas	Beauveria + flies	Beauveria + lice
*Puccinia*	**Puccinia + ants**	Puccinia + aphids	**Puccinia + bedbugs**	Puccinia + bees	Puccinia + beetles	**Puccinia + fleas**	Puccinia + flies	**Puccinia + lice**
*Cordyceps*	Cordyceps + ants	Cordyceps + aphids	**Cordyceps + bedbugs**	Cordyceps + bees	Cordyceps + beetles	**Cordyceps + fleas**	Cordyceps + flies	**Cordyceps + lice**
*Trichophyton*	Trichophyton + ants	**Trichophyton + aphids**	**Trichophyton + bedbugs**	Trichophyton + bees	**Trichophyton + beetles**	**Trichophyton + fleas**	Trichophyton + flies	Trichophyton + lice
*Metarhizium*	Metarhizium + ants	Metarhizium + aphids	Metarhizium + bedbugs	Metarhizium + bees	Metarhizium + beetles	Metarhizium + fleas	Metarhizium + flies	**Metarhizium + lice**
*Pythium*	**Pythium + ants**	**Pythium + aphids**	**Pythium + bedbugs**	Pythium + bees	**Pythium + beetles**	**Pythium + fleas**	**Pythium + flies**	**Pythium + lice**
*Funneliformis*	**Funneliformis + ants**	Funneliformis + aphids	**Funneliformis + bedbugs**	**Funneliformis + bees**	**Funneliformis + beetles**	**Funneliformis + fleas**	**Funneliformis + flies**	**Funneliformis + lice**
*Ustilago*	**Ustilago + ants**	**Ustilago + aphids**	**Ustilago + bedbugs**	Ustilago + bees	Ustilago + beetles	**Ustilago + fleas**	**Ustilago + flies**	**Ustilago + lice**
*Rhizoglomus*	**Rhizoglomus + ants**	**Rhizoglomus + aphids**	**Rhizoglomus +** **bedbugs**	**Rhizoglomus + bees**	**Rhizoglomus + beetles**	**Rhizoglomus + fleas**	**Rhizoglomus + flies**	**Rhizoglomus + lice**
*Acremonium*	**Acremonium + ants**	Acremonium + aphids	**Acremonium +** **bedbugs**	Acremonium + bees	Acremonium + beetles	**Acremonium + fleas**	Acremonium + flies	**Acremonium + lice**
*Chaetomium*	Chaetomium + ants	Chaetomium + aphids	**Chaetomium +** **bedbugs**	Chaetomium + bees	Chaetomium + beetles	**Chaetomium + fleas**	Chaetomium + flies	**Chaetomium + lice**
*Paecilomyces*	Paecilomyces + ants	Paecilomyces + aphids	**Paecilomyces + bedbugs**	Paecilomyces + bees	Paecilomyces + beetles	**Paecilomyces + fleas**	Paecilomyces + flies	**Paecilomyces + lice**
*Trichosporon*	Trichosporon + ants	**Trichosporon + aphids**	**Trichosporon + bedbugs**	**Trichosporon + bees**	Trichosporon + beetles	**Trichosporon + fleas**	Trichosporon + flies	Trichosporon + lice
*Malassezia*	**Malassezia + ants**	**Malassezia + aphids**	**Malassezia + bedbugs**	**Malassezia + bees**	**Malassezia + beetles**	**Malassezia + fleas**	Malassezia + flies	**Malassezia + lice**
*Phoma*	**Phoma + ants**	Phoma + aphids	**Phoma + bedbugs**	Phoma + bees	Phoma + beetles	**Phoma + fleas**	Phoma + flies	**Phoma + lice**
*Thermomyces*	**Thermomyces + ants**	**Thermomyces + aphids**	**Thermomyces + bedbugs**	**Thermomyces + bees**	**Thermomyces + beetles**	**Thermomyces + fleas**	**Thermomyces + flies**	**Thermomyces + lice**
*Lentinus*	**Lentinus + ants**	Lentinus + aphids	**Lentinus + bedbugs**	**Lentinus + bees**	**Lentinus + beetles**	**Lentinus + fleas**	**Lentinus + flies**	**Lentinus + lice**
*Mortierella*	Mortierella + ants	Mortierella + aphids	**Mortierella + bedbugs**	Mortierella + bees	Mortierella + beetles	**Mortierella + fleas**	Mortierella + flies	**Mortierella + lice**
*Debaryomyces*	Debaryomyces + ants	**Debaryomyces +** **aphids**	**Debaryomyces + bedbugs**	Debaryomyces + bees	Debaryomyces + beetles	**Debaryomyces + fleas**	Debaryomyces + flies	Debaryomyces + lice
*Metschnikowia*	**Metschnikowia + ants**	Metschnikowia + aphids	**Metschnikowia + bedbugs**	Metschnikowia + bees	Metschnikowia + beetles	**Metschnikowia + fleas**	Metschnikowia + flies	**Metschnikowia + lice**
*Talaromyces*	Talaromyces + ants	Talaromyces + aphids	**Talaromyces + bedbugs**	Talaromyces + bees	Talaromyces + beetles	**Talaromyces + fleas**	Talaromyces + flies	**Talaromyces + lice**
*Geotrichum*	Geotrichum + ants	**Geotrichum + aphids**	**Geotrichum + bedbugs**	Geotrichum + bees	Geotrichum + beetles	**Geotrichum + fleas**	Geotrichum + flies	**Geotrichum + lice**
*Pestalotiopsis*	Pestalotiopsis + ants	**Pestalotiopsis + aphids**	**Pestalotiopsis + bedbugs**	**Pestalotiopsis + bees**	Pestalotiopsis + beetles	**Pestalotiopsis + fleas**	**Pestalotiopsis + flies**	**Pestalotiopsis + lice**
*Microsporum*	**Microsporum + ants**	**Microsporum + aphids**	**Microsporum + bedbugs**	**Microsporum + bees**	Microsporum + beetles	**Microsporum + fleas**	Microsporum + flies	Microsporum + lice
*Curvularia*	Curvularia + ants	**Curvularia + aphids**	Curvularia + bedbugs	Curvularia + bees	Curvularia + beetles	**Curvularia + fleas**	Curvularia + flies	**Curvularia + lice**
*Rhizomucor*	Rhizomucor + ants	**Rhizomucor + aphids**	**Rhizomucor + bedbugs**	**Rhizomucor + bees**	Rhizomucor + beetles	**Rhizomucor + fleas**	**Rhizomucor + flies**	**Rhizomucor + lice**
*Pyricularia*	**Pyricularia + ants**	**Pyricularia + aphids**	**Pyricularia + bedbugs**	**Pyricularia + bees**	**Pyricularia + beetles**	**Pyricularia + fleas**	**Pyricularia + flies**	**Pyricularia + lice**
*Parastagonospora*	**Parastagonospora + ants**	Parastagonospora + aphids	**Parastagonospora + bedbugs**	**Parastagonospora + bees**	**Parastagonospora + beetles**	**Parastagonospora + fleas**	**Parastagonospora + flies**	**Parastagonospora + lice**
*Monascus*	**Monascus + ants**	**Monascus + aphids**	**Monascus + bedbugs**	Monascus + bees	Monascus + beetles	**Monascus + fleas**	**Monascus + flies**	**Monascus + lice**
*Hanseniaspora*	Hanseniaspora + ants	Hanseniaspora + aphids	**Hanseniaspora + bedbugs**	Hanseniaspora + bees	Hanseniaspora + beetles	**Hanseniaspora + fleas**	Hanseniaspora + flies	**Hanseniaspora + lice**
*Paracoccidioides*	**Paracoccidioides + ants**	**Paracoccidioides + aphids**	**Paracoccidioides + bedbugs**	Paracoccidioides + bees	**Paracoccidioides + beetles**	**Paracoccidioides +** **fleas**	Paracoccidioides + flies	**Paracoccidioides + lice**
*Schizophyllum*	**Schizophyllum + ants**	**Schizophyllum +** **aphids**	**Schizophyllum + bedbugs**	**Schizophyllum + bees**	Schizophyllum + beetles	**Schizophyllum + fleas**	**Schizophyllum + flies**	**Schizophyllum + lice**
*Plasmopara*	**Plasmopara + ants**	**Plasmopara + aphids**	**Plasmopara + bedbugs**	**Plasmopara + bees**	**Plasmopara + beetles**	**Plasmopara + fleas**	**Plasmopara + flies**	**Plasmopara + lice**
*Auricularia*	**Auricularia + ants**	**Auricularia + aphids**	**Auricularia + bedbugs**	**Auricularia + bees**	**Auricularia + beetles**	**Auricularia + fleas**	**Auricularia + flies**	**Auricularia + lice**
*Russula*	**Russula + ants**	**Russula + aphids**	**Russula + bedbugs**	**Russula + bees**	Russula + beetles	**Russula + fleas**	**Russula + flies**	**Russula + lice**
*Zygosaccharomyces*	**Zygosaccharomyces + ants**	**Zygosaccharomyces + aphids**	**Zygosaccharomyces + bedbugs**	Zygosaccharomyces + bees	Zygosaccharomyces + beetles	**Zygosaccharomyces + fleas**	Zygosaccharomyces + flies	**Zygosaccharomyces + lice**
*Torulaspora*	**Torulaspora + ants**	**Torulaspora + aphids**	**Torulaspora + bedbugs**	Torulaspora + bees	**Torulaspora + beetles**	**Torulaspora + fleas**	Torulaspora + flies	**Torulaspora + lice**
*Boletus*	**Boletus + ants**	**Boletus + aphids**	**Boletus + bedbugs**	**Boletus + bees**	**Boletus + beetles**	**Boletus + fleas**	**Boletus + flies**	**Boletus + lice**
*Botryosphaeria*	**Botryosphaeria + ants**	**Botryosphaeria + aphids**	**Botryosphaeria + bedbugs**	**Botryosphaeria + bees**	Botryosphaeria + beetles	**Botryosphaeria + fleas**	Botryosphaeria + flies	**Botryosphaeria + lice**
*Cunninghamella*	**Cunninghamella + ants**	Cunninghamella + aphids	**Cunninghamella + bedbugs**	Cunninghamella + bees	Cunninghamella + beetles	**Cunninghamella + fleas**	Cunninghamella + flies	**Cunninghamella + lice**
*Diaporthe*	**Diaporthe + ants**	Diaporthe + aphids	**Diaporthe + bedbugs**	**Diaporthe + bees**	Diaporthe + beetles	**Diaporthe + fleas**	Diaporthe + flies	**Diaporthe + lice**
*Bipolaris*	Bipolaris + ants	**Bipolaris + aphids**	**Bipolaris + bedbugs**	Bipolaris + bees	Bipolaris + beetles	**Bipolaris + fleas**	**Bipolaris + flies**	**Bipolaris + lice**
*Lentinula*	**Lentinula + ants**	Lentinula + aphids	**Lentinula + bedbugs**	**Lentinula + bees**	Lentinula + beetles	**Lentinula + fleas**	Lentinula + flies	**Lentinula + lice**
*Erysiphe*	**Erysiphe + ants**	Erysiphe + aphids	**Erysiphe + bedbugs**	**Erysiphe + bees**	Erysiphe + beetles	**Erysiphe + fleas**	**Erysiphe + flies**	**Erysiphe + lice**
*Scedosporium*	**Scedosporium + ants**	**Scedosporium + aphids**	**Scedosporium + bedbugs**	**Scedosporium + bees**	Scedosporium + beetles	**Scedosporium + fleas**	**Scedosporium + flies**	**Scedosporium + lice**
*Zymoseptoria*	**Zymoseptoria + ants**	**Zymoseptoria + aphids**	**Zymoseptoria + bedbugs**	**Zymoseptoria + bees**	**Zymoseptoria + beetles**	**Zymoseptoria + fleas**	**Zymoseptoria + flies**	**Zymoseptoria + lice**
*Phellinus*	**Phellinus + ants**	**Phellinus + aphids**	**Phellinus + bedbugs**	**Phellinus + bees**	Phellinus + beetles	**Phellinus + fleas**	**Phellinus + flies**	**Phellinus + lice**
*Sporothrix*	**Sporothrix + ants**	**Sporothrix + aphids**	**Sporothrix + bedbugs**	Sporothrix + bees	Sporothrix + beetles	**Sporothrix + fleas**	**Sporothrix + flies**	**Sporothrix + lice**
*Macrophomina*	**Macrophomina + ants**	**Macrophomina + aphids**	**Macrophomina + bedbugs**	**Macrophomina + bees**	Macrophomina + beetles	**Macrophomina + fleas**	Macrophomina + flies	**Macrophomina + lice**
*Flammulina*	**Flammulina + ants**	**Flammulina + aphids**	**Flammulina + bedbugs**	**Flammulina + bees**	**Flammulina + beetles**	**Flammulina + fleas**	Flammulina + flies	**Flammulina + lice**
*Pseudogymnoascus*	**Pseudogymnoascus + ants**	**Pseudogymnoascus + aphids**	**Pseudogymnoascus + bedbugs**	Pseudogymnoascus + bees	**Pseudogymnoascus + beetles**	**Pseudogymnoascus + fleas**	Pseudogymnoascus + flies	**Pseudogymnoascus + lice**
*Podospora*	**Podospora + ants**	**Podospora + aphids**	**Podospora + bedbugs**	**Podospora + bees**	**Podospora + beetles**	**Podospora + fleas**	Podospora + flies	**Podospora + lice**
*Amanita*	Amanita + ants	**Amanita + aphids**	**Amanita + bedbugs**	Amanita + bees	**Amanita + beetles**	**Amanita + fleas**	Amanita + flies	**Amanita + lice**
*Cercospora*	**Cercospora + ants**	**Cercospora + aphids**	**Cercospora + bedbugs**	**Cercospora + bees**	**Cercospora + beetles**	**Cercospora + fleas**	**Cercospora + flies**	**Cercospora + lice**
*Lactarius*	**Lactarius + ants**	**Lactarius + aphids**	**Lactarius + bedbugs**	**Lactarius + bees**	**Lactarius + beetles**	**Lactarius + fleas**	Lactarius + flies	**Lactarius + lice**
*Lasiodiplodia*	Lasiodiplodia + ants	Lasiodiplodia + aphids	**Lasiodiplodia + bedbugs**	**Lasiodiplodia + bees**	Lasiodiplodia + beetles	**Lasiodiplodia + fleas**	Lasiodiplodia + flies	**Lasiodiplodia + lice**
*Exophiala*	Exophiala + ants	**Exophiala + aphids**	**Exophiala + bedbugs**	**Exophiala + bees**	Exophiala + beetles	**Exophiala + fleas**	**Exophiala + flies**	**Exophiala + lice**
*Monilinia*	**Monilinia + ants**	**Monilinia + aphids**	**Monilinia + bedbugs**	Monilinia + bees	Monilinia + beetles	**Monilinia + fleas**	Monilinia + flies	**Monilinia + lice**
*Coccidioides*	**Coccidioides + ants**	**Coccidioides + aphids**	**Coccidioides + bedbugs**	**Coccidioides + bees**	**Coccidioides + beetles**	**Coccidioides + fleas**	**Coccidioides + flies**	**Coccidioides + lice**
*Melampsora*	**Melampsora + ants**	Melampsora + aphids	**Melampsora + bedbugs**	Melampsora + bees	Melampsora + beetles	**Melampsora + fleas**	**Melampsora + flies**	**Melampsora + lice**
*Antrodia*	**Antrodia + ants**	**Antrodia + aphids**	**Antrodia + bedbugs**	**Antrodia + bees**	**Antrodia + beetles**	**Antrodia + fleas**	**Antrodia + flies**	**Antrodia + lice**
*Brettanomyces*	**Brettanomyces + ants**	**Brettanomyces +** **aphids**	**Brettanomyces + bedbugs**	Brettanomyces + bees	**Brettanomyces + beetles**	**Brettanomyces + fleas**	Brettanomyces + flies	**Brettanomyces + lice**
*Ascochyta*	**Ascochyta + ants**	Ascochyta + aphids	**Ascochyta + bedbugs**	**Ascochyta + bees**	**Ascochyta + beetles**	**Ascochyta + fleas**	Ascochyta + flies	**Ascochyta + lice**
*Epichloe*	**Epichloe + ants**	Epichloe + aphids	**Epichloe + bedbugs**	**Epichloe + bees**	Epichloe + beetles	**Epichloe + fleas**	Epichloe + flies	**Epichloe + lice**
*Pyrenophora*	**Pyrenophora + ants**	Pyrenophora + aphids	**Pyrenophora +** **bedbugs**	**Pyrenophora + bees**	**Pyrenophora + beetles**	**Pyrenophora + fleas**	**Pyrenophora + flies**	**Pyrenophora + lice**
*Hymenoscyphus*	Hymenoscyphus + ants	**Hymenoscyphus + aphids**	**Hymenoscyphus + bedbugs**	**Hymenoscyphus + bees**	Hymenoscyphus + beetles	**Hymenoscyphus + fleas**	**Hymenoscyphus + flies**	**Hymenoscyphus + lice**
*Diplodia*	**Diplodia + ants**	**Diplodia + aphids**	**Diplodia + bedbugs**	**Diplodia + bees**	Diplodia + beetles	**Diplodia + fleas**	**Diplodia + flies**	**Diplodia + lice**
*Inonotus*	**Inonotus + ants**	**Inonotus + aphids**	**Inonotus + bedbugs**	**Inonotus + bees**	Inonotus + beetles	**Inonotus + fleas**	Inonotus + flies	**Inonotus + lice**
*Ophiostoma*	Ophiostoma + ants	**Ophiostoma + aphids**	**Ophiostoma + bedbugs**	Ophiostoma + bees	Ophiostoma + beetles	**Ophiostoma + fleas**	**Ophiostoma + flies**	**Ophiostoma + lice**
*Neofusicoccum*	**Neofusicoccum + ants**	**Neofusicoccum + aphids**	**Neofusicoccum + bedbugs**	**Neofusicoccum + bees**	Neofusicoccum + beetles	**Neofusicoccum + fleas**	**Neofusicoccum + flies**	**Neofusicoccum + lice**
*Hericium*	**Hericium + ants**	**Hericium + aphids**	**Hericium + bedbugs**	**Hericium + bees**	**Hericium + beetles**	**Hericium + fleas**	**Hericium + flies**	**Hericium + lice**
*Phakopsora*	**Phakopsora + ants**	**Phakopsora + aphids**	**Phakopsora + bedbugs**	**Phakopsora + bees**	**Phakopsora + beetles**	**Phakopsora + fleas**	**Phakopsora + flies**	**Phakopsora + lice**
*Leptosphaeria*	**Leptosphaeria + ants**	**Leptosphaeria + aphids**	**Leptosphaeria + bedbugs**	**Leptosphaeria + bees**	Leptosphaeria + beetles	**Leptosphaeria + fleas**	**Leptosphaeria + flies**	**Leptosphaeria + lice**

^*a*^ Ants: *Formica rufa* or *Formica fusca* or *Camponotus ligniperda* or *Camponotus vagus* or *Myrmica rubra* or *Atta cephalotes* or *Solenopsis geminata* or *Pogonomyrmex barbatus* or *Tapinoma sessile* or *Odontomachus bauri* or *Leptogenys* or *Solenopsis invicta* or *Lasius niger* or *Eciton burchellii* or *Eciton hamatum*.

^*b*^ Aphids: *Aphis fabae* or *Aphis gossypii* or *Aphis nerii* or *Aphis pomi* or *Aphis varians* or *Rhopalosiphum padi* or *Myzus persicae* or *Schizaphis graminum* or *Toxoptera citricida*.

^*c*^ Bedbugs: *Cimex lectularius* or *Cimex hemipterus* or *Leptocimex boueti* or *Cimex adjunctus*.

^*d*^ Bees: *Apis mellifera* or *Apis cerana* or *Bombus terrestris* or *Bombus impatiens* or *Xylocopa violacea* or *Melipona beecheii* or *Andrena fulva* or *Osmia bicornis*.

^*e*^ Beetles: *Coleoptera* or *Cactophagus* or *Epilachna varivestis* or *Aphidecta oblita* or *Tenebrio molitor* or *Hylobius abietis* or *Acalymma vittatum* or *Scolytus multistriatus* or *Anoplophora glabripennis* or *Diabrotica virgifera* or *Lymantria dispar* or *Agrilus planipennis* or *Oryctes rhinoceros* or *Dendroctonus ponderosae* or *Cryptorhynchus lapathi* or *Balaninus glandium* or *Phyllotreta cruciferae* or *Chrysomela* or *Carabus* or *Staphylinus* or *Cucujus*.

^*f*^ Fleas: *Pulex* or *Ctenocephalides felis* or *Ctenocephalides canis* or *Echidnophaga gallinacea* or *Pulex simulans* or *Xenopsylla cheopis* or *Tunga penetrans*.

^*g*^ Flies: *Musca domestica* or *Drosophila melanogaster* or *Calliphora vomitoria* or *Sarcophaga carnaria* or *Lucilia sericata* or *Fannia canicularis* or *Glossina morsitans* or *Brachyceridae* or *Ceratitis capitata* or *Anopheles gambiae* or *Aedes aegypti* or *Culex pipiens or Chironomus* or *Cladotanytarsus* or *Procladius* or *Glyptotendipes* or *Culicoides* or *Forcipomyia* or *Leptoconops* or *Simulium* or *Prosimulium* or *Stegopterna* or *Dixa* or *Dixella* or *Deuterophlebia* or *Thaumalea* or *Thaumalydia* or *Tephritidae* or *Syrphidae* or *Tachinidae*.

^*h*^ Lice: *Trichodectes canis* or *Linognathus setosus* or *Heterodoxus spiniger* or *Felicola subrostratus*.

^*i*^ Fungi reference: 12.

^*j*^ Normal print: there is an association in the literature; bold print: there is no association found in the literature.

**TABLE 2:** Published association of pathogenic fungi with invertebrate carriage^*i*^ *^,^*^*j*^

Fungi	Mites^*a*^	Mosquitoes^*b*^	Moths^*c*^	Roaches^*d*^	Spiders^*e*^	Termites^*f*^	Ticks^*g*^	Wasps^*h*^
*Saccharomyces*	Saccharomyces + mites	Saccharomyces + mosquitoes	Saccharomyces + moths	Saccharomyces + roaches	**Saccharomyces + spiders**	Saccharomyces + termites	**Saccharomyces + ticks**	Saccharomyces + waspas
*Candida*	Candida + mites	Candida + mosquitoes	Candida + moths	Candida + roaches	Candida + spiders	Candida + termites	Candida + ticks	Candida + wasps
*Aspergillus*	Aspergillus + mites	Aspergillus + Cladosporiummosquitoes	Aspergillus + moths	Aspergillus + roaches	Aspergillus + spiders	Aspergillus + termites	Aspergillus + ticks	Aspergillus + wasps
*Fusarium*	Fusarium + mites	Fusarium + mosquitoes	Fusarium + moths	Fusarium + roaches	Fusarium + spiders	Fusarium + termites	Fusarium + ticks	Fusarium + wasps
*Penicillium*	Penicillium + mites	Penicillium + mosquitoes	Penicillium + moths	Penicillium + roaches	Penicillium + spiders	Penicillium + termites	Penicillium + ticks	Penicillium + wasps
*Penicillium*	Trichoderma + mites	Trichoderma + mosquitoes	Trichoderma + moths	Trichoderma + roaches	Trichoderma + spiders	Trichoderma + termites	Trichoderma + ticks	Trichoderma + wasps
*Botrytis*	Botrytis + mites	**Botrytis + mosquitoes**	Botrytis + moths	**Botrytis + roaches**	**Botrytis + spiders**	**Botrytis + termites**	**Botrytis + ticks**	Botrytis + wasps
*Pichia*	**Pichia + mites**	**Pichia + mosquitoes**	**Pichia + moths**	Pichia + roaches	**Pichia + spiders**	**Pichia + termites**	Pichia + ticks	**Pichia + wasps**
*Cryptococcus*	Cryptococcus + mites	Cryptococcus + mosquitoes	Cryptococcus + moths	Cryptococcus + roaches	**Cryptococcus + spiders**	**Cryptococcus + termites**	**Cryptococcus + ticks**	Cryptococcus + wasps
*Alternaria*	Alternaria + mites	Alternaria + mosquitoes	**Alternaria + moths**	Alternaria + roaches	**Alternaria + spiders**	Alternaria + termites	Alternaria + ticks	**Alternaria + wasps**
*Phytophthora*	**Phytophthora + mites**	**Phytophthora + mosquitoes**	Phytophthora + moths	**Phytophthora + roaches**	**Phytophthora + spiders**	Phytophthora + termites	**Phytophthora + ticks**	**Phytophthora + wasps**
*Rhizopus*	Rhizopus + mites	Rhizopus + mosquitoes	Rhizopus + moths	Rhizopus + roaches	Rhizopus + spiders	Rhizopus + termites	Rhizopus + ticks	**Rhizopus + wasps**
*Phanerochaete*	**Phanerochaete + mites**	**Phanerochaete + mosquitoes**	**Phanerochaete + moths**	**Phanerochaete + roaches**	**Phanerochaete + spiders**	**Phanerochaete + termites**	**Phanerochaete + ticks**	**Phanerochaete + wasps**
*Colletotrichum*	**Colletotrichum + mites**	Colletotrichum + mosquitoes	Colletotrichum + moths	**Colletotrichum + roaches**	**Colletotrichum + spiders**	Colletotrichum + termites	**Colletotrichum + ticks**	Colletotrichum + wasps
*Trametes*	**Trametes + mites**	**Trametes + mosquitoes**	Trametes + moths	**Trametes + roaches**	**Trametes + spiders**	Trametes + termites	**Trametes + ticks**	**Trametes + wasps**
*Rhizoctonia*	**Rhizoctonia + mites**	**Rhizoctonia + mosquitoes**	**Rhizoctonia + moths**	**Rhizoctonia + roaches**	**Rhizoctonia + spiders**	**Rhizoctonia + termites**	**Rhizoctonia + ticks**	**Rhizoctonia + wasps**
*Pleurotus*	Pleurotus + mites	Pleurotus + mosquitoes	**Pleurotus + moths**	Pleurotus + roaches	Pleurotus + spiders	**Pleurotus + termites**	**Pleurotus + ticks**	Pleurotus + wasps
*Ganoderma*	**Ganoderma + mites**	**Ganoderma + mosquitoes**	**Ganoderma + moths**	**Ganoderma + roaches**	**Ganoderma + spiders**	Ganoderma + termites	Ganoderma + ticks	**Ganoderma + wasps**
*Neurospora*	Neurospora + mites	**Neurospora + mosquitoes**	**Neurospora + moths**	**Neurospora + roaches**	**Neurospora + spiders**	Neurospora + termites	**Neurospora + ticks**	**Neurospora + wasps**
*Cladosporium*	Cladosporium + mites	Cladosporium + mosquitoes	Cladosporium + moths	Cladosporium + roaches	Cladosporium + spiders	Cladosporium + termites	Cladosporium + ticks	Cladosporium + wasps
*Yarrowia*	**Yarrowia + mites**	Yarrowia + mosquitoes	**Yarrowia + moths**	**Yarrowia + roaches**	**Yarrowia + spiders**	**Yarrowia + termites**	**Yarrowia + ticks**	**Yarrowia + wasps**
*Agaricus*	Agaricus + mites	Agaricus + mosquitoes	**Agaricus + moths**	**Agaricus + roaches**	**Agaricus + spiders**	**Agaricus + termites**	**Agaricus + ticks**	**Agaricus + wasps**
*Kluyveromyces*	**Kluyveromyces + mites**	**Kluyveromyces + mosquitoes**	**Kluyveromyces + moths**	Kluyveromyces + roaches	**Kluyveromyces + spiders**	**Kluyveromyces + termites**	**Kluyveromyces + ticks**	**Kluyveromyces + wasps**
*Mucor*	Mucor + mites	Mucor + mosquitoes	Mucor + moths	Mucor + roaches	Mucor + spiders	Mucor + termites	Mucor + ticks	Mucor + wasps
*Verticillium*	Verticillium + mites	Verticillium + mosquitoes	Verticillium + moths	Verticillium + roaches	Verticillium + spiders	**Verticillium + termites**	Verticillium + ticks	**Verticillium + wasps**
*Sclerotinia*	Sclerotinia + mites	**Sclerotinia + mosquitoes**	**Sclerotinia + moths**	**Sclerotinia + roaches**	**Sclerotinia + spiders**	Sclerotinia + termites	**Sclerotinia + ticks**	**Sclerotinia + wasps**
*Rhodotorula*	**Rhodotorula + mites**	Rhodotorula + mosquitoes	**Rhodotorula + moths**	Rhodotorula + roaches	Rhodotorula + spiders	Rhodotorula + termites	**Rhodotorula + ticks**	Rhodotorula + wasps
*Beauveria*	Beauveria + mites	Beauveria + mosquitoes	Beauveria + moths	Beauveria + roaches	Beauveria + spiders	Beauveria + termites	Beauveria + ticks	Beauveria + wasps
*Puccinia*	Puccinia + mites	**Puccinia + mosquitoes**	**Puccinia + moths**	**Puccinia + roaches**	**Puccinia + spiders**	**Puccinia + termites**	**Puccinia + ticks**	**Puccinia + wasps**
*Cordyceps*	Cordyceps + mites	Cordyceps + mosquitoes	Cordyceps + moths	**Cordyceps + roaches**	Cordyceps + spiders	**Cordyceps + termites**	**Cordyceps + ticks**	Cordyceps + wasps
*Trichophyton*	Trichophyton + mites	Trichophyton + mosquitoes	**Trichophyton + moths**	**Trichophyton +** **roaches**	**Trichophyton + spiders**	**Trichophyton +** **termites**	**Trichophyton + ticks**	**Trichophyton + wasps**
*Metarhizium*	Metarhizium + mites	Metarhizium + mosquitoes	Metarhizium + moths	Metarhizium + roaches	Metarhizium + spiders	Metarhizium + termites	Metarhizium + ticks	Metarhizium + wasps
*Pythium*	Pythium + mites	Pythium + mosquitoes	Pythium + moths	Pythium + roaches	**Pythium + spiders**	Pythium + termites	**Pythium + ticks**	**Pythium + wasps**
*Pythium*	**Funneliformis + mites**	**Funneliformis + mosquitoes**	**Funneliformis + moths**	**Funneliformis + roaches**	**Funneliformis + spiders**	Funneliformis + termites	**Funneliformis + ticks**	**Funneliformis + wasps**
*Ustilago*	**Ustilago + mites**	**Ustilago + mosquitoes**	Ustilago + moths	**Ustilago + roaches**	**Ustilago + spiders**	**Ustilago + termites**	**Ustilago + ticks**	**Ustilago + wasps**
*Rhizoglomus*	**Rhizoglomus + mites**	**Rhizoglomus + mosquitoes**	**Rhizoglomus + moths**	**Rhizoglomus + roaches**	**Rhizoglomus + spiders**	**Rhizoglomus +** **termites**	**Rhizoglomus + ticks**	**Rhizoglomus + wasps**
*Acremonium*	Acremonium + mites	Acremonium + mosquitoes	Acremonium + moths	Acremonium + roaches	**Acremonium + spiders**	Acremonium + termites	**Acremonium + ticks**	**Acremonium + wasps**
*Chaetomium*	Chaetomium + mites	**Chaetomium + mosquitoes**	Chaetomium + moths	**Chaetomium + roaches**	**Chaetomium + spiders**	Chaetomium + termites	**Chaetomium + ticks**	**Chaetomium + wasps**
*Paecilomyces*	Paecilomyces + mites	Paecilomyces + mosquitoes	Paecilomyces + moths	Paecilomyces + roaches	Paecilomyces + spiders	Paecilomyces + termites	Paecilomyces + ticks	Paecilomyces + wasps
*Paecilomyces*	**Trichosporon + mites**	Trichosporon + mosquitoes	**Trichosporon + moths**	**Trichosporon + roaches**	Trichosporon + spiders	Trichosporon + termites	Trichosporon + ticks	Trichosporon + wasps
*Malassezia*	**Malassezia + mites**	**Malassezia + mosquitoes**	**Malassezia + moths**	**Malassezia + roaches**	**Malassezia + spiders**	**Malassezia + termites**	**Malassezia + ticks**	**Malassezia + wasps**
*Phoma*	Phoma + mites	**Phoma + mosquitoes**	Phoma + moths	**Phoma + roaches**	**Phoma + spiders**	Phoma + termites	Phoma + ticks	Phoma + wasps
*Thermomyces*	**Thermomyces + mites**	**Thermomyces + mosquitoes**	**Thermomyces + moths**	**Thermomyces + roaches**	**Thermomyces + spiders**	**Thermomyces + termites**	**Thermomyces + ticks**	**Thermomyces + wasps**
*Lentinus*	**Lentinus + mites**	**Lentinus + mosquitoes**	**Lentinus + moths**	**Lentinus + roaches**	**Lentinus + spiders**	**Lentinus + termites**	**Lentinus + ticks**	**Lentinus + wasps**
*Mortierella*	Mortierella + mites	**Mortierella + mosquitoes**	Mortierella + moths	**Mortierella + roaches**	Mortierella + spiders	**Mortierella + termites**	Mortierella + ticks	Mortierella + wasps
*Debaryomyces*	Debaryomyces + mites	**Debaryomyces + mosquitoes**	**Debaryomyces + moths**	Debaryomyces + roaches	**Debaryomyces + spiders**	Debaryomyces + termites	**Debaryomyces + ticks**	**Debaryomyces + wasps**
*Metschnikowia*	**Metschnikowia + mites**	**Metschnikowia + mosquitoes**	Metschnikowia + moths	Metschnikowia + roaches	**Metschnikowia + spiders**	Metschnikowia + termites	**Metschnikowia + ticks**	Metschnikowia + wasps
*Talaromyces*	Talaromyces + mites	Talaromyces + mosquitoes	Talaromyces + moths	Talaromyces + roaches	**Talaromyces + spiders**	Talaromyces + termites	Talaromyces + ticks	Talaromyces + wasps
*Geotrichum*	Geotrichum + mites	Geotrichum + mosquitoes	Geotrichum + moths	Geotrichum + roaches	**Geotrichum + spiders**	**Geotrichum + termites**	**Geotrichum + ticks**	**Geotrichum + wasps**
*Pestalotiopsis*	Pestalotiopsis + mites	Pestalotiopsis + mosquitoes	Pestalotiopsis + moths	Pestalotiopsis + roaches	**Pestalotiopsis + spiders**	Pestalotiopsis + termites	**Pestalotiopsis + Tick**	Pestalotiopsis + wasps
*Microsporum*	Microsporum + mites	Microsporum + mosquitoes	**Microsporum + moths**	**Microsporum +** **roaches**	**Microsporum + spiders**	**Microsporum +** **termites**	**Microsporum + ticks**	**Microsporum + wasps**
*Curvularia*	**Curvularia + mites**	**Curvularia +** **mosquitoes**	Curvularia + moths	Curvularia + roaches	**Curvularia + spiders**	Curvularia + termites	**Curvularia + ticks**	**Curvularia + wasps**
*Rhizomucor*	**Rhizomucor + mites**	**Rhizomucor + mosquitoes**	**Rhizomucor + moths**	**Rhizomucor + roaches**	**Rhizomucor + spiders**	Rhizomucor + termites	Rhizomucor + ticks	**Rhizomucor + wasps**
*Pyricularia*	Pyricularia + mites	**Pyricularia + mosquitoes**	**Pyricularia + moths**	**Pyricularia + roaches**	**Pyricularia + spiders**	**Pyricularia + termites**	**Pyricularia + ticks**	**Pyricularia + wasps**
*Parastagonospora*	**Parastagonospora + mites**	**Parastagonospora + mosquitoes**	**Parastagonospora + moths**	**Parastagonospora + roaches**	**Parastagonospora + spiders**	**Parastagonospora + termites**	**Parastagonospora + ticks**	**Parastagonospora + wasps**
*Monascus*	**Monascus + mites**	**Monascus + mosquitoes**	**Monascus + moths**	**Monascus + roaches**	**Monascus + spiders**	**Monascus + termites**	**Monascus + ticks**	**Monascus + wasps**
*Hanseniaspora*	**Hanseniaspora + mites**	**Hanseniaspora + mosquitoes**	Hanseniaspora + moths	**Hanseniaspora + roaches**	**Hanseniaspora + spiders**	**Hanseniaspora + termites**	**Hanseniaspora + ticks**	Hanseniaspora + wasps
*Paracoccidioides*	**Paracoccidioides + mites**	**Paracoccidioides + mosquitoes**	Paracoccidioides + moths	Paracoccidioides + roaches	Paracoccidioides + spiders	Paracoccidioides + termites	Paracoccidioides + ticks	Paracoccidioides + wasps
*Schizophyllum*	**Schizophyllum + mites**	**Schizophyllum + mosquitoes**	**Schizophyllum + moths**	**Schizophyllum + roaches**	**Schizophyllum + spiders**	**Schizophyllum + termites**	**Schizophyllum + ticks**	**Schizophyllum + wasps**
*Plasmopara*	Plasmopara + mites	**Plasmopara + mosquitoes**	Plasmopara + moths	**Plasmopara + roaches**	**Plasmopara + spiders**	**Plasmopara + termites**	**Plasmopara + ticks**	**Plasmopara + wasps**
*Auricularia*	**Auricularia + mites**	**Auricularia + mosquitoes**	**Auricularia + moths**	**Auricularia + roaches**	**Auricularia + spiders**	**Auricularia + termites**	**Auricularia + ticks**	**Auricularia + wasps**
*Russula*	**Russula + mites**	**Russula + mosquitoes**	**Russula + moths**	**Russula + roaches**	**Russula + spiders**	**Russula + termites**	**Russula + ticks**	**Russula + wasps**
*Zygosaccharomyces*	**Zygosaccharomyces + mites**	**Zygosaccharomyces + mosquitoes**	Zygosaccharomyces + moths	Zygosaccharomyces + roaches	**Zygosaccharomyces + spiders**	**Zygosaccharomyces + termites**	**Zygosaccharomyces + ticks**	**Zygosaccharomyces + wasps**
*Torulaspora*	**Torulaspora + mites**	**Torulaspora + mosquitoes**	**Torulaspora + moths**	Torulaspora + roaches	**Torulaspora + spiders**	**Torulaspora + termites**	**Torulaspora + ticks**	**Torulaspora + wasps**
*Boletus*	Boletus + mites	**Boletus + mosquitoes**	**Boletus + moths**	**Boletus + roaches**	**Boletus + spiders**	**Boletus + termites**	**Boletus + ticks**	**Boletus + wasps**
*Botryosphaeria*	**Botryosphaeria + mites**	**Botryosphaeria + mosquitoes**	**Botryosphaeria +** **moths**	**Botryosphaeria + roaches**	**Botryosphaeria + spiders**	**Botryosphaeria + termites**	**Botryosphaeria + ticks**	Botryosphaeria + wasps
*Cunninghamella*	Cunninghamella + mites	**Cunninghamella + mosquitoes**	Cunninghamella + moths	**Cunninghamella + roaches**	**Cunninghamella + spiders**	Cunninghamella + termites	**Cunninghamella + ticks**	Cunninghamella + wasps
*Diaporthe*	**Diaporthe + mites**	Diaporthe + mosquitoes	Diaporthe + moths	**Diaporthe + roaches**	**Diaporthe + spiders**	**Diaporthe + termites**	**Diaporthe + ticks**	Diaporthe + wasps
*Bipolaris*	Bipolaris + mites	**Bipolaris + mosquitoes**	**Bipolaris + moths**	**Bipolaris + roaches**	**Bipolaris + spiders**	**Bipolaris + termites**	**Bipolaris + ticks**	**Bipolaris + wasps**
*Lentinula*	Lentinula + mites	**Lentinula + mosquitoes**	Lentinula + moths	**Lentinula + roaches**	**Lentinula + spiders**	**Lentinula + termites**	**Lentinula + ticks**	Lentinula + wasps
*Erysiphe*	Erysiphe + mites	**Erysiphe + mosquitoes**	Erysiphe + moths	**Erysiphe + roaches**	**Erysiphe + spiders**	**Erysiphe + termites**	**Erysiphe + ticks**	Erysiphe + wasps
*Scedosporium*	**Scedosporium + mites**	**Scedosporium + mosquitoes**	**Scedosporium + moths**	Scedosporium + roaches	**Scedosporium + spiders**	**Scedosporium + termites**	**Scedosporium + ticks**	**Scedosporium + wasps**
*Zymoseptoria*	**Zymoseptoria + mites**	**Zymoseptoria + mosquitoes**	**Zymoseptoria + moths**	**Zymoseptoria + roaches**	**Zymoseptoria + spiders**	**Zymoseptoria + termites**	**Zymoseptoria + ticks**	**Zymoseptoria + wasps**
*Phellinus*	**Phellinus + mites**	**Phellinus + mosquitoes**	**Phellinus + moths**	**Phellinus + roaches**	**Phellinus + spiders**	**Phellinus + termites**	**Phellinus + ticks**	**Phellinus + wasps**
*Sporothrix*	Sporothrix + mites	Sporothrix + mosquitoes	**Sporothrix + moths**	Sporothrix + roaches	**Sporothrix + spiders**	Sporothrix + termites	**Sporothrix + ticks**	**Sporothrix + wasps**
*Macrophomina*	Macrophomina + mites	**Macrophomina + mosquitoes**	**Macrophomina +** **moths**	**Macrophomina + roaches**	**Macrophomina + spiders**	**Macrophomina + termites**	**Macrophomina + ticks**	**Macrophomina + wasps**
*Flammulina*	Flammulina + mites	**Flammulina + mosquitoes**	**Flammulina + moths**	**Flammulina + roaches**	**Flammulina + spiders**	**Flammulina + termites**	**Flammulina + ticks**	**Flammulina + wasps**
*Pseudogymnoascus*	Pseudogymnoascus + mites	**Pseudogymnoascus + mosquitoes**	Pseudogymnoascus + moths	**Pseudogymnoascus + roaches**	**Pseudogymnoascus + spiders**	**Pseudogymnoascus + termites**	**Pseudogymnoascus + ticks**	**Pseudogymnoascus + wasps**
*Podospora*	**Podospora + mites**	Podospora + mosquitoes	**Podospora + moths**	**Podospora + roaches**	**Podospora + spiders**	**Podospora + termites**	**Podospora + ticks**	**Podospora + wasps**
*Amanita*	Amanita + mites	**Amanita + mosquitoes**	**Amanita + moths**	**Amanita + roaches**	**Amanita + spiders**	**Amanita + termites**	**Amanita + ticks**	**Amanita + wasps**
*Cercospora*	**Cercospora + mites**	**Cercospora + mosquitoes**	**Cercospora + moths**	**Cercospora + roaches**	**Cercospora + spiders**	**Cercospora + termites**	**Cercospora + ticks**	**Cercospora + wasps**
*Lactarius*	**Lactarius + mites**	**Lactarius + mosquitoes**	**Lactarius + moths**	**Lactarius + roaches**	**Lactarius + spiders**	**Lactarius + termites**	**Lactarius + ticks**	**Lactarius + wasps**
*Lasiodiplodia*	**Lasiodiplodia + mites**	**Lasiodiplodia + mosquitoes**	Lasiodiplodia + moths	**Lasiodiplodia +** **roaches**	**Lasiodiplodia + spiders**	**Lasiodiplodia +** **termites**	**Lasiodiplodia + ticks**	**Lasiodiplodia + wasps**
*Exophiala*	**Exophiala + mites**	**Exophiala + mosquitoes**	**Exophiala + moths**	**Exophiala + roaches**	**Exophiala + spiders**	**Exophiala + termites**	**Exophiala + ticks**	Exophiala + wasps
*Monilinia*	**Monilinia + mites**	**Monilinia + mosquitoes**	**Monilinia + moths**	**Monilinia + roaches**	**Monilinia + spiders**	**Monilinia + termites**	**Monilinia + ticks**	**Monilinia + wasps**
*Coccidioides*	**Coccidioides + mites**	**Coccidioides + mosquitoes**	**Coccidioides + moths**	**Coccidioides + roaches**	**Coccidioides + spiders**	**Coccidioides + termites**	**Coccidioides + ticks**	**Coccidioides + wasps**
*Melampsora*	**Melampsora + mites**	**Melampsora + mosquitoes**	Melampsora + moths	**Melampsora + roaches**	**Melampsora + spiders**	**Melampsora + termites**	**Melampsora + ticks**	**Melampsora + wasps**
*Antrodia*	**Antrodia + mites**	**Antrodia + mosquitoes**	**Antrodia + moths**	**Antrodia + roaches**	**Antrodia + spiders**	**Antrodia + termites**	**Antrodia + ticks**	**Antrodia + wasps**
*Brettanomyces*	**Brettanomyces + mites**	**Brettanomyces + mosquitoes**	**Brettanomyces + moths**	**Brettanomyces + roaches**	**Brettanomyces + spiders**	**Brettanomyces + termites**	**Brettanomyces + ticks**	**Brettanomyces + wasps**
*Ascochyta*	Ascochyta + mites	Ascochyta + mosquitoes	**Ascochyta + moths**	**Ascochyta + roaches**	Ascochyta + spiders	**Ascochyta + termites**	Ascochyta + ticks	**Ascochyta + wasps**
*Epichloe*	Epichloe + mites	**Epichloe + mosquitoes**	**Epichloe + moths**	**Epichloe + roaches**	**Epichloe + spiders**	**Epichloe + termites**	**Epichloe + ticks**	**Epichloe + wasps**
*Pyrenophora*	**Pyrenophora + mites**	**Pyrenophora + mosquitoes**	**Pyrenophora + moths**	**Pyrenophora + roaches**	**Pyrenophora + spiders**	**Pyrenophora + termites**	**Pyrenophora + ticks**	**Pyrenophora + wasps**
*Hymenoscyphus*	Hymenoscyphus + mites	**Hymenoscyphus + mosquitoes**	**Hymenoscyphus + moths**	**Hymenoscyphus + roaches**	Hymenoscyphus + spiders	**Hymenoscyphus + termites**	**Hymenoscyphus + ticks**	Hymenoscyphus + wasps
*Diplodia*	**Diplodia + mites**	**Diplodia + mosquitoes**	**Diplodia + moths**	Diplodia + roaches	Diplodia + spiders	Diplodia + termites	**Diplodia + ticks**	Diplodia + wasps
*Inonotus*	**Inonotus + mites**	**Inonotus + mosquitoes**	Inonotus + moths	**Inonotus + roaches**	**Inonotus + spiders**	**Inonotus + termites**	**Inonotus + ticks**	Inonotus + wasps
*Ophiostoma*	Ophiostoma + mites	**Ophiostoma + mosquitoes**	**Ophiostoma + moths**	**Ophiostoma + roaches**	**Ophiostoma + spiders**	Ophiostoma + termites	**Ophiostoma + ticks**	**Ophiostoma + wasps**
*Neofusicoccum*	**Neofusicoccum + mites**	**Neofusicoccum + mosquitoes**	Neofusicoccum + moths	**Neofusicoccum + roaches**	**Neofusicoccum + spiders**	**Neofusicoccum + termites**	**Neofusicoccum + ticks**	**Neofusicoccum + wasps**
*Hericium*	**Hericium + mites**	**Hericium + mosquitoes**	**Hericium + moths**	**Hericium + roaches**	**Hericium + spiders**	**Hericium + termites**	**Hericium + ticks**	**Hericium + wasps**
*Phakopsora*	Phakopsora + mites	**Phakopsora + mosquitoes**	**Phakopsora + moths**	**Phakopsora + roaches**	**Phakopsora + spiders**	**Phakopsora + termites**	**Phakopsora + ticks**	**Phakopsora + wasps**
*Leptosphaeria*	**Leptosphaeria + mites**	**Leptosphaeria + mosquitoes**	Leptosphaeria + moths	**Leptosphaeria + roaches**	**Leptosphaeria +** **spiders**	**Leptosphaeria + termites**	**Leptosphaeria + ticks**	**Leptosphaeria + wasps**

^*a*^ Mites: *Demodex canis* or *Sarcoptes scabiei* or *Cheyletiella yasguri* or *Otodectes cynotis* or *Notoedres cati* or *Psoroptes cuniculi*.

^*b*^ Mosquitoes: *Aedes aegypti* or *Anopheles gambiae* or *Culex pipiens* or *Culex quinquefasciatus* or *Aedes albopictus*.

^*c*^ Moths: *Bombyx mori* or *Lymantria dispar* or *Spodoptera frugiperda* or *Tineola bisselliella* or *Plodia interpunctella* or *Noctuidae* or *Lasiocampidae*.

^*d*^ Roaches: *Periplaneta americana* or *Blattella germanica* or *Supella longipalpa* or *Blatta orientalis* or *Nauphoeta cinerea*.

^*e*^ Spiders: *Araneus diadematus* or *Latrodectus mactans* or *Loxosceles reclusa* or *Tegenaria agrestis* or *Atrax robustus*.

^*f*^ Termites: *Coptotermes formosanus* or *Reticulitermes flavipes* or *Zootermopsis angusticollis* or *Macrotermes bellicosus* or *Nasutitermes corniger*.

^*g*^ Ticks: *Ixodes scapularis* or *Rhipicephalus (Boophilus) microplus* or *Dermacentor variabilis* or *Amblyomma americanum* or *Ixodes pacificus*.

^*h*^ Wasps: *Vespula* or *Polistes dominula* or *Dolichovespula maculata* or *Vespula atropilosa*.

^*i*^ Fungi reference: 12.

^*j*^ Normal print: there is an association in the literature; bold print: there is no association found in the literature.

We previously summarized an underappreciated entity, that of invertebrate bite-associated cutaneous invasive fungal infections (IBA-cIFIs) (13). In that series, 12 of 22 (55%) cases occurred in immunocompetent patients. For the cases where information was available, 11 out of 12 involved insects whose bite was associated with toxin release, specifically spiders (*n* = 8), scorpions (*n* = 2), and bees (*n* = 1). The predominant fungal pathogens were mold (Mucorales in eight, *Aspergillus* in 4, and *Fusarium*, *Purpureocillium*, and *Exophiala* spp. in 1 each) pathogens (15 of 22, 68%); 2 cases were mixed fungal infections (*Aspergillus flavus* and *Fusarium proliferatum*, *Aspergillus flavus*, and *Candida* spp., 1 each). Of interest, four of the eight cases of Mucorales infection were caused by uncommon species (*Apophysomycetes elegans* and *Saksenae vasiformis*, two cases each). Furthermore, a variety of bacteria were commonly recovered from these wounds (*Nocardia asteroides*, *Staphylococcus epidermidis*, *Klebsiella* spp., *Bacillus* spp.). Commonly (68%), these cases had necrotizing features, including fasciitis, and were routinely misdiagnosed as recalcitrant bacterial cellulitis. Consequently, the fungal etiologic diagnosis was delayed, and outcomes were poor, despite frequent amputations and surgical debridement. Although the incidence, prevalence, and etiology of IBA-cIFIs are unknown, our study reflected on reporting and publication bias of severe or recalcitrant cases, and there is a high likelihood that these infections are underreported. Specifically, it is likely that our cases represent the tip of the iceberg, such that many patients with an IBA-cIFI had a self-limiting presentation and never sought medical attention. In addition, the incidence of IBA-cIFIs following bites by small arthropods such as mosquitoes, sandflies, bedbugs, head lice, midges, and fleas is likely underestimated due to recall biases.

Global warming worsens pollution and urbanization and puts humans, especially the poor and disadvantaged, at risk of a variety of infections, including a broad spectrum of fungal infections (6, 14). Moreover, global warming was hypothesized to result in increased migration, geographical redistribution, and longer seasons for envenomation in a variety of terrestrial venomous species and insects (15, 16). In addition, given the pollution and urbanization associated with climate change, there is an increased organic waste (17), a natural attractant for insects (18). Given that insects are poikilotherms, higher ambient temperatures drive up their metabolic rate, which in turn increases the demand for food, resulting in an increase in the frequency of bites. Hence, the elevated ambient temperatures associated with global warming can be expected to increase insect aggressiveness and the likelihood of human bites (19).

Through intricate interplay, individual invertebrate-pathogen interactions can change, often in an unanticipated way. In particular, the effect of fungi on thermal tolerance of the insects is complex and variable. Porras et al. examined the effect of fungal pathogen *Beauveria bassiana* on the ranges of thermal tolerance and behavior of an herbivorous insect and its predator beetle and found that fungal infection narrowed the thermal tolerance range and inhibited thermal boldness behaviors to cross extreme temperatures (20). Although not studied in detail, by the nature of environmental interconnectivity, invertebrates carry fungi (Tables 1 and 2), and such interactions shape their antifungal immunity (21, 22). For example, marine invertebrate microbiomes include many fungal species including some known to be pathogenic in humans, such as *Aspergillus* and *Penicillum* spp. (23). Few studies have been done in terrestrial biting invertebrates, but mosquitoes are known to carry a diverse set of fungal species (24).

IBA-cIFIs could occur through a decrease in the barrier of intact skin as high temperatures trigger a suboptimal local immune response (4) and an increase in frequency and severity of pre-existing cutaneous disorders, including atopic dermatitis (25). The establishment of an IBA-IFI is through direct introduction of fungi that pre-colonize the intact or diseased skin (25). This direct inoculation can be augmented by the effect of tissue necrosis by toxins produced by the invertebrates.

**Fig 2:**
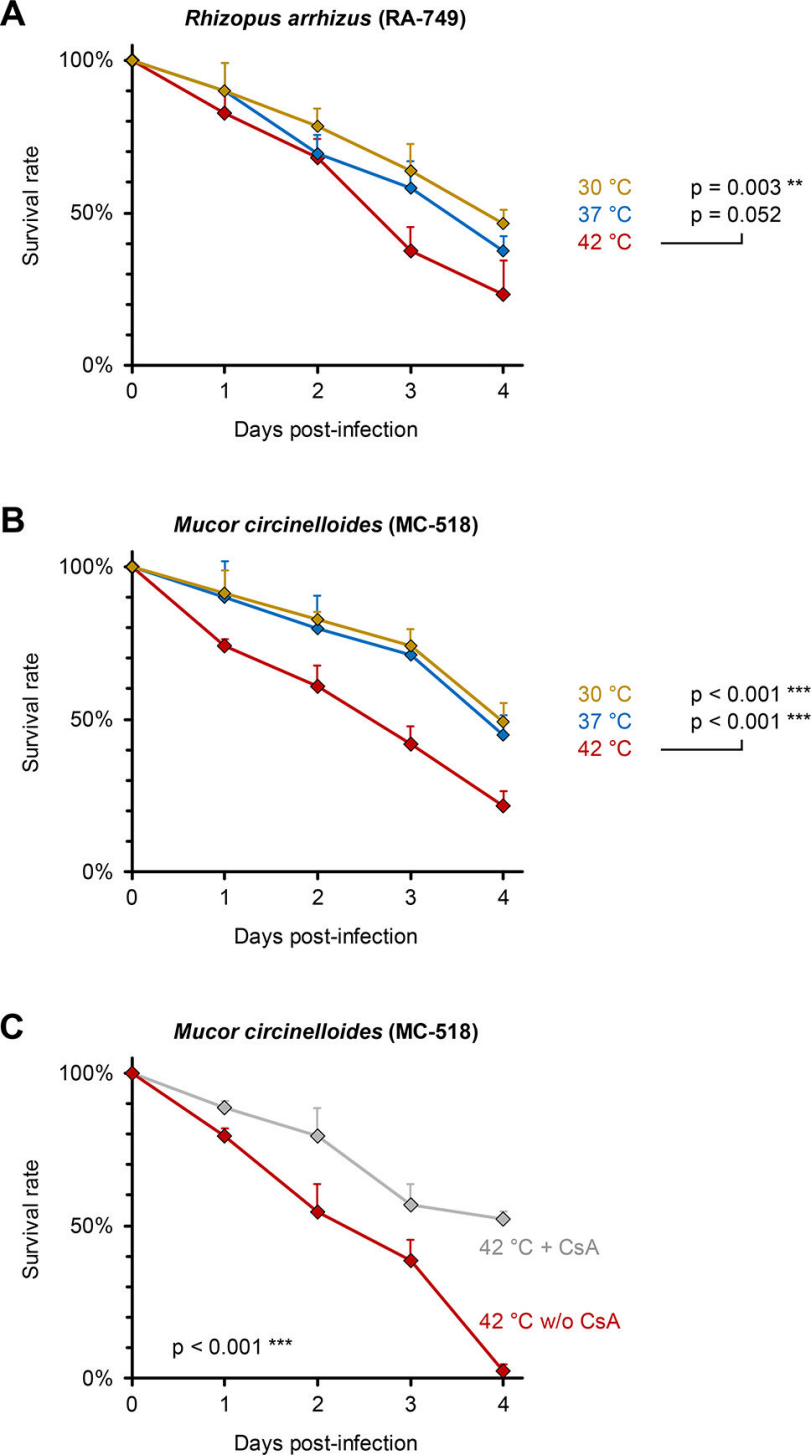
High growth temperature induces calcineurin-dependent hypervirulence in Mucorales. (**A and B**) *Rhizopus arrhizus* (clinical isolate RA-749) and *Mucor circinelloides* (clinical isolate MC-518) were streaked on Sabouraud agar (SAB) plates and grown for 5 days at 30°C, 37°C, or 42°C. Spores were collected, washed with phosphate-buffered saline (PBS), and counted with a hemocytometer. Female wild-type *Drosophila melanogaster* flies were pricked with a needle dipped into the spore suspensions (5 × 10^7^ spores/mL in PBS). Survival of flies was monitored for 4 days. Aggregate survival curves and standard deviations (error bars) from three independent experiments with a total of 69 flies per isolate and condition are shown. (**C**) MC-518 was streaked on SAB plates with or without (w/o) 100 mg/mL cyclosporin A (CsA) and grown for 5 days at 42°C. Flies were infected and monitored as described above. Aggregate survival curves and standard deviations (error bars) from two independent experiments with a total of 44 flies per condition are shown. Statistical significance was assessed using Mantel-Cox log-rank test. **P < 0.01, ***P < 0.001.

As shown in bacteria (26), viruses (27), and metazoan parasites (28), one can hypothesize the effect of high temperatures on the virulence of fungi that extend beyond their effects on local and systemic human immunity, the behavior of invertebrates, and changes in skin and vector mycobiome. Experimental studies have shown that increasing growth temperatures affect fungal biology, such as the *in vivo* adaptation and microevolution of the important human yeast pathogen *Cryptococcus deneoformans* (29) and the uncommon opportunistic multidrug-resistant yeast *Rhodosporidiobolus fluvialis* (8). No comparable studies have been done with molds, the most common causes of IBA-cIFIs (13). Using a fly model of mucormycosis (30), we found increased virulence of Mucorales (most common fungi implicated in IBA-cIFIs) when grown in high temperatures. This change in virulence was not strain- or Mucorales species-dependent. In addition, this phenotype was linked to stress response, as co-incubation with a calcineurin inhibitor abrogated the increased virulence associated with higher temperatures (Fig. 2). The calcineurin-dependent effect of Mucorales virulence following other stresses, such as mechanical stress, simulating the natural disaster condition of tornadic force, has been previously shown (31). Given that the production of a mucotoxin (mucorin) was recently shown to be important in Mucorales (32) and *Candida albicans* (candidalycin) (33) pathogenesis, an intriguing question is whether increased thermal stress during mammalian infection results in increased exotoxin production. Experimentation with calcineurin-deficient fungi can test further the hypothesis, beyond phenocopying with calcineurin inhibitors, and whether virulence increases in fungi following growth in high temperatures are due genetic or epigenetic changes. Finally, the effects of high temperatures on the virulence of other molds and yeasts, the “inoculum effect” (single vs multiple bites) that overcomes the skin infection barrier, are unknown.

Numerous questions are posed by the phenomenon of IBI-cIFA. Specifically, rising temperatures affect the ecological niches of insects and fungal biology in a dynamic way and depend on many environmental parameters (e.g., soil microbiome and mycobiome fluctuations of temperature, light/dark cycles, and other geoclimatic factors), in conjunction with downstream interdependencies with changes in plant, amphibians, and bird physiology and their ecological niches. Central to this hypothesis is that insects are ectotherms, and their body temperature reflects environmental temperatures. Consequently, all their associated microbes experience environmental temperatures. Currently, most fungal species cannot survive at mammalian temperatures (34), and this thermal defensive barrier, combined with the multifaceted antifungal immunity present in vertebrates, is a likely explanation for the paucity of invasive fungal diseases in immunologically intact hosts (35). As the world warms, invertebrate-associated microbes must adapt to warmer temperatures or perish. Those that adapt could gain the ability to grow at mammalian temperatures, defeat their thermal barrier, and potentially acquire the capacity for human virulence and invasive infection, even following an “innocent” bite (Fig. 1). Conceptually, vertebrate-adapted, known, or emerging fungi can survive in mammalian hosts despite their highly effective innate immune mechanisms. Such fungi may have the capacity to overcome mammalian innate immunity and thermal exclusion (36). Thus, the combination of higher thermal tolerance and established ability to persist despite invertebrate immunity could make such organisms particularly dangerous to humans and associated livestock.

In conclusion, global warming, in addition to many other deleterious effects, might create the conditions for a perfect storm to unleash new or more frequent and severe fungal diseases of the skin, even in immunocompetent hosts, creating a formidable stress to the heathcare infrastructure. In addition, such effects can affect in a global sense all endotherms, such as amphibians, birds, and mammals, with direct and indirect effects on their heath, including potentially catastrophic effects on food industry. Studies in the field of thermal ecology and developing a co-evolutionary framework to make predictions are more important and urgent than ever. In addition to insect behavior, future studies should examine the effects of high temperatures on invertebrate reproduction, life cycles, and transmissibility of pathogens. Importantly, the study of the effects of global warming and climate change on the biology of both human hosts and various pathogens, including fungi, should be context dependent, along with the effects of biodiversity loss, pollution, and urbanization on different aquatic and terrestrial systems (37). Similarly, the drivers of climate-induced changes in invertebrate populations are complex and dynamic and can have opposing, both direct and indirect, effects on the population, foraging dynamics, nutrient availability, thermal and reproductive physiologies, and ecological networks of various invertebrates ( 20, 37, 38).

Using the “One Heath” perspective and multidisciplinary approach, coupled with careful surveillance at the regional and global levels (e.g., surveillance for IBA-IFIs rates in temperate vs tropical environments), the study of genotypic and phenotypic plasticity and thermal limits of insects, fungi, and mammalian hosts and their interplay will shed light on the web of complex, direct, and indirect environmental interactions and give us anticipatory knowledge of the problems that might come in the future.

## SUPPLEMENTAL MATERIAL

Supplemental Material mbio.03447-24-s0001.docx Research strategy and bibliography by selected invertebrate groups.

ASM does not own the copyrights to Supplemental Material that may be linked to, or accessed through, an article. The authors have granted ASM a non-exclusive, world-wide license to publish the Supplemental Material files. Please contact the corresponding author directly for reuse.
