# Supplementary material for: Would global warming bring an increase of invertebrate-associated cutaneous invasive fungal infections?
Source: mBio. 2025 Feb 5;16(3):e03447-24. doi: 10.1128/mbio.03447-24 (PMC11898599; doi:10.1128/mbio.03447-24)
Supplement: Supplemental Material — Research strategy and bibliography by selected invertebrate groups. [file mbio.03447-24-s0001.docx]

Supplement

Research strategy and Bibliography by Selected Invertebrate Groups.

**Methods:**

**We performed a comprehensive search of the literature, carried out by a medical librarian (R.S.H.). Medline (Ovid), Embase (Ovid), and Google Scholar were queried, with no date restrictions, utilizing both controlled vocabulary and natural language terms as shown.** To select the list of invertebrates for our search, we used the following publication as guide. (<https://link.springer.com/article/10.1007/s10841-013-9565-9>)

**Selected Common Invertebrates:**

**ANTS:**
Formica rufa OR Formica fusca OR Camponotus ligniperda OR Camponotus vagus OR Myrmica rubra OR Atta cephalotes OR Solenopsis geminata OR Pogonomyrmex barbatus OR Tapinoma sessile OR Odontomachus bauri OR Leptogenys OR Solenopsis invicta OR Lasius niger OR Eciton burchellii OR Eciton hamatum

**APHIDS:**
Aphis fabae OR Aphis gossypii OR Aphis nerii OR Aphis pomi OR Aphis varians OR Rhopalosiphum padi OR Myzus persicae OR Schizaphis graminum OR Toxoptera citricida

**BEDBUGS:**
Cimex lectularius OR Cimex hemipterus OR Leptocimex boueti OR Cimex adjunctus

**BEES:**
Apis mellifera OR Apis cerana OR Bombus terrestris OR Bombus impatiens OR Xylocopa violacea OR Melipona beecheii OR Andrena fulva OR Osmia bicornis

**BEETLES:**
Coleoptera OR Cactophagus OR Epilachna varivestis OR Aphidecta oblita OR Tenebrio molitor OR Hylobius abietis OR Acalymma vittatum OR Scolytus multistriatus OR Anoplophora glabripennis OR Diabrotica virgifera OR Lymantria dispar OR Agrilus planipennis OR Oryctes rhinoceros OR Dendroctonus ponderosae OR Cryptorhynchus lapathi OR Balaninus glandium OR Phyllotreta cruciferae OR Chrysomela OR Carabus OR Staphylinus OR Cucujus

**Fleas:**
Pulex OR Ctenocephalides felis OR Ctenocephalides canis OR Echidnophaga gallinacea OR Pulex simulans OR Xenopsylla cheopis OR Tunga penetrans

**Flies:**Musca domestica OR Drosophila melanogaster OR Calliphora vomitoria OR Sarcophaga carnaria OR Lucilia sericata OR Fannia canicularis OR Glossina morsitans OR Brachyceridae OR Ceratitis capitata OR Anopheles gambiae OR Aedes aegypti OR Culex pipiens OR *Chironomus* OR *Cladotanytarsus* OR *Procladius* OR *Glyptotendipes* OR *Culicoides* OR *Forcipomyia* OR *Leptoconops* OR *Simulium* OR *Prosimulium* OR *Stegopterna* OR *Dixa* OR *Dixella* OR *Deuterophlebia* OR *Thaumalea* OR *Thaumalydia* OR *Tephritidae* OR *Syrphidae* OR *Tachinidae*

**Lice:**
Trichodectes canis OR Linognathus setosus OR Heterodoxus spiniger OR Felicola subrostratus

**Mites:**
Demodex canis OR Sarcoptes scabiei OR Cheyletiella yasguri OR Otodectes cynotis OR Notoedres cati OR Psoroptes cuniculi

**Mosquitoes:**
Aedes aegypti OR Anopheles gambiae OR Culex pipiens OR Culex quinquefasciatus OR Aedes albopictus

**Moths:**
Bombyx mori OR Lymantria dispar OR Spodoptera frugiperda OR Tineola bisselliella OR Plodia interpunctella OR Noctuidae OR Lasiocampidae

**Roaches:**
Periplaneta americana OR Blattella germanica OR Supella longipalpa OR Blatta orientalis OR Nauphoeta cinerea

**Spiders:**
Araneus diadematus OR Latrodectus mactans OR Loxosceles reclusa OR Tegenaria agrestis OR Atrax robustus

**Termites:**
Coptotermes formosanus OR Reticulitermes flavipes OR Zootermopsis angusticollis OR Macrotermes bellicosus OR Nasutitermes corniger

**Ticks:**
Ixodes scapularis OR Rhipicephalus (Boophilus) microplus OR Dermacentor variabilis OR Amblyomma americanum OR Ixodes pacificus

**Wasps:**
Vespula OR Polistes dominula OR Dolichovespula maculata OR Vespula atropilosa

**Each species name was searched in conjunction with the 100 most cited fungal genera.**

(Saccharomyces OR Candida OR Aspergillus OR Fusarium OR Penicillium OR Trichoderma OR Botrytis OR Pichia OR Cryptococcus OR Alternaria OR Phytophthora OR Rhizopus OR Phanerochaete OR Colletotrichum OR Trametes OR Rhizoctonia OR Pleurotus OR Ganoderma OR Neurospora OR Cladosporium OR Yarrowia OR Agaricus OR Kluyveromyces OR Mucor OR Verticillium OR Sclerotinia OR Rhodotorula OR Beauveria OR Puccinia OR Cordyceps OR Trichophyton OR Metarhizium OR Pythium OR Cuneiformis OR Ustilago OR Rhizoglyphus OR Acremonium OR Chaetomium OR Paecilomyces OR Trichosporon OR Malassezia OR Phoma OR Thermomyces OR Lentinus OR Mortierella OR Talaromyces OR Metschnikovii OR Geotrichum OR Pestalotiopsis OR Microsporum OR Curvularia OR Rhizomucor OR Pyricularia OR Stagonospora OR Monascus OR Hapsidospora OR Paracoccidioides OR Schizophyllum OR Plasmodia OR Auricularia OR Russula OR Zygosaccharomyces OR Torulaspora OR Boletus OR Botryosphaeria OR Cunninghamella OR Diaporthe OR Bipolaris OR Lentinula OR Erysiphe OR Scedosporium OR Septoria OR Phellinus OR Sporothrix OR Macrophomina OR Flammulina OR Pseudonymous OR Podospora OR Amanita OR Cercospora OR Lactarius OR Lasiodiplodia OR Exophiala OR Monilinia OR Coccidioides OR Melampsora OR Antrodia OR Gaeumannomyces OR Ascochyta OR Epichloe OR Pyrenophora OR Hyemoschus OR Diplodia OR Inodorus OR Ophiostoma OR Fusicoccum OR Hericium OR Aplacophora OR Leptosphaeria)

Ants: (Formica rufa OR Formica fusca OR Camponotus ligniperda OR Camponotus vagus OR Myrmica rubra OR Atta cephalotes OR Solenopsis geminata OR Pogonomyrmex barbatus OR Dorymyrmex insanus OR Tapinoma sessile OR Odontomachus bauri OR Leptogenys processionalis OR Solenopsis invicta OR Lasius niger OR Aphaenogaster picea OR Eciton burchellii OR Eciton hamatum)

Abonyo, E. A., Maniania, N. K., Warui, C. M., Kokwaro, E. D., Palmer, T. M., Doak, D. F., & Brody, A. K. (2016). Effects of entomopathogenic fungus Metarhizium anisopliae on non-target ants associated with Odontotermes spp. (Isoptera: Termitidae) termite mounds in Kenya. *International Journal of Tropical Insect Science*, *36*(3), 128–134. Scopus. <https://doi.org/10.1017/S1742758416000114>

Abramowski, D., Currie, C. R., & Poulsen, M. (2011). Caste specialization in behavioral defenses against fungus garden parasites in Acromyrmex octospinosus leaf-cutting ants. *Insectes Sociaux*, *58*(1), 65–75. Scopus. <https://doi.org/10.1007/s00040-010-0117-y>

Abril, A. B., & Bucher, E. H. (2004). Nutritional sources of the fungus cultured by leaf-cutting ants. *Applied Soil Ecology*, *26*(3), 243–247. Scopus. <https://doi.org/10.1016/j.apsoil.2003.12.008>

Abril, A. B., & Bucher, E. H. (2007). Genetic diversity of fungi occurring in nests of three Acromyrmex leaf-cutting ant species from Córdoba, Argentina. *Microbial Ecology*, *54*(3), 417–423. Scopus. <https://doi.org/10.1007/s00248-007-9252-z>

Adams, R. M. M., Liberti, J., Illum, A. A., Jones, T. H., Nash, D. R., & Boomsma, J. J. (2013). Chemically armed mercenary ants protect fungus-farming societies. *Proceedings of the National Academy of Sciences of the United States of America*, *110*(39), 15752–15757. Scopus. <https://doi.org/10.1073/pnas.1311654110>

Adams, R. M. M., & Longino, J. T. (2007). Nesting biology of the arboreal fungus-growing ant Cyphomyrmex cornutus and behavioral interactions with the social-parasitic ant Megalomyrmex mondabora. *Insectes Sociaux*, *54*(2), 136–143. Scopus. <https://doi.org/10.1007/s00040-007-0922-0>

Adams, R. M. M., Mueller, U. G., Holloway, A. K., Green, A. M., & Narozniak, J. (2000). Garden sharing and garden stealing in fungus-growing ants. *Naturwissenschaften*, *87*(11), 491–493. Scopus. <https://doi.org/10.1007/s001140050765>

Adams, R. M. M., Mueller, U. G., Schultz, T. R., & Norden, B. (2000). Agro-predation: Usurpation of attine fungus gardens by Megalomyrmex ants. *Naturwissenschaften*, *87*(12), 549–554. Scopus. <https://doi.org/10.1007/s001140050777>

Advani, N. K., & Mueller, U. G. (2006). A preference assay for quantifying symbiont choice in fungus-growing ants (Attini, Formicidae). *Insectes Sociaux*, *53*(4), 446–455. Scopus. <https://doi.org/10.1007/s00040-005-0892-7>

Aguilar-Colorado, Á. S., & Rivera-Chávez, J. (2023). Ants/Nest-Associated Fungi and Their Specialized Metabolites: Taxonomy, Chemistry, and Bioactivity. *Revista Brasileira de Farmacognosia*, *33*(5), 901–923. Scopus. <https://doi.org/10.1007/s43450-023-00417-3>

Akanbi, M. O. (1986). OBSERVATIONS OF THE BIOTIC FACTORS AFFECTING THE POPULATIONS OF EPICERURA-PULVERULENTA HAMPSON (LEPIDOPTERA, NOTODONTIDAE). *INSECT SCIENCE AND ITS APPLICATION*, *7*(6), 785–789. <https://doi.org/10.1017/S1742758400011905>

Allen, M. F., Shulman, H., Rundel, P. W., Harmon, T. C., & Aronson, E. L. (2023). Leaf-cutter ants – mycorrhizal fungi: Observations and research questions from an unexpected mutualism. *Frontiers in Fungal Biology*, *4*. Scopus. <https://doi.org/10.3389/ffunb.2023.1241916>

Alves, S. B., Pereira, R. M., Stimac, J. L., & Vieira, S. A. (1996). Delayed germination of Beauveria bassiana conidia after prolonged storage at low, above-freezing temperatures. *BIOCONTROL SCIENCE AND TECHNOLOGY*, *6*(4), 575–581. <https://doi.org/10.1080/09583159631217>

Amaral, K. D., Gandra, L. C., de Souza, D. J., & Della Lucia, T. M. C. (2020). Deleterious action of azadirachtin against the mutualistic fungus of leaf-cutting ants. *Journal of Basic Microbiology*, *60*(11–12), 931–937. Scopus. <https://doi.org/10.1002/jobm.202000541>

Andriolli, F. S., Ishikawa, N. K., Vargas-Isla, R., Cabral, T. S., De Bekker, C., & Baccaro, F. B. (2019). Do zombie ant fungi turn their hosts into light seekers? *Behavioral Ecology*, *30*(3), 609–616. Scopus. <https://doi.org/10.1093/beheco/ary198>

Angelone, S., & Bidochka, M. J. (2018). Diversity and abundance of entomopathogenic fungi at ant colonies. *Journal of Invertebrate Pathology*, *156*, 73–76. Scopus. <https://doi.org/10.1016/j.jip.2018.07.009>

Aquino, R. S. S., Silveira, S. S., Pessoa, W. F. B., Rodrigues, A., Andrioli, J. L., Delabie, J. H. C., & Fontana, R. (2013). Filamentous fungi vectored by ants (Hymenoptera: Formicidae) in a public hospital in north-eastern Brazil. *Journal of Hospital Infection*, *83*(3), 200–204. Scopus. <https://doi.org/10.1016/j.jhin.2012.11.022>

Araújo, J. P. M., Evans, H. C., Fernandes, I. O., Ishler, M. J., & Hughes, D. P. (2020). Zombie-ant fungi cross continents: II. Myrmecophilous hymenostilboid species and a novel zombie lineage. *Mycologia*, *112*(6), 1138–1170. Scopus. <https://doi.org/10.1080/00275514.2020.1822093>

Araújo, J. P. M., Evans, H. C., Geiser, D. M., Mackay, W. P., & Hughes, D. P. (2015). Unravelling the diversity behind the Ophiocordyceps unilateralis (Ophiocordycipitaceae) complex: Three new species of zombie-ant fungi from the Brazilian Amazon. *Phytotaxa*, *220*(3), 224–238. Scopus. <https://doi.org/10.11646/phytotaxa.220.3.2>

Araújo, J. P. M., Evans, H. C., Kepler, R., & Hughes, D. P. (2018). Zombie-ant fungi across continents: 15 new species and new combinations within Ophiocordyceps. I. Myrmecophilous hirsutelloid species. *Studies in Mycology*, *90*, 119–160. Scopus. <https://doi.org/10.1016/j.simyco.2017.12.002>

Araújo, J. P. M., & Hughes, D. P. (2019). Zombie-Ant Fungi Emerged from Non-manipulating, Beetle-Infecting Ancestors. *Current Biology*, *29*(21), 3735-3738.e2. Scopus. <https://doi.org/10.1016/j.cub.2019.09.004>

Araújo, J. P. M., Lebert, B. M., Vermeulen, S., Brachmann, A., Ohm, R. A., Evans, H. C., & de Bekker, C. (2022). Masters of the manipulator: Two new hypocrealean genera, Niveomyces (Cordycipitaceae) and Torrubiellomyces (Ophiocordycipitaceae), parasitic on the zombie ant fungus Ophiocordyceps camponoti-floridani. *Persoonia: Molecular Phylogeny and Evolution of Fungi*, *49*, 174–194. Scopus. <https://doi.org/10.3767/persoonia.2022.49.05>

Arcuri, S. L., Pagnocca, F. C., Melo, W. G. D., Nagamoto, N. S., Komura, D. L., & Rodrigues, A. (2014). Yeasts found on an ephemeral reproductive caste of the leaf-cutting ant Atta sexdens rubropilosa. *ANTONIE VAN LEEUWENHOEK INTERNATIONAL JOURNAL OF GENERAL AND MOLECULAR MICROBIOLOGY*, *106*(3), 475–487. <https://doi.org/10.1007/s10482-014-0216-2>

Arenas, A., & Roces, F. (2016). Gardeners and midden workers in leaf-cutting ants learn to avoid plants unsuitable for the fungus at their worksites. *Animal Behaviour*, *115*, 167–174. Scopus. <https://doi.org/10.1016/j.anbehav.2016.03.016>

Arenas, A., & Roces, F. (2017). Avoidance of plants unsuitable for the symbiotic fungus in leaf-cutting ants: Learning can take place entirely at the colony dump. *PLoS ONE*, *12*(3). Scopus. <https://doi.org/10.1371/journal.pone.0171388>

Arsene, M. M. J., Viktorovna, P. I., Mikhailovitch, M. K., Davares, A. K. L., Parfait, K., Rehailia, M., Nikolayevich, S. A., Stefanovna, G. V., Sarra, S., Sulikoevich, K. Z., Anatolyevna, C. Z., & Shommiya, D. (2022). In vitro antimicrobial activity, antibioresistance reversal properties, and toxicity screen of ethanolic extracts of Heracleum mantegazzianum Sommier and Levier (giant hogweed), Centaureajacea L. (brown knapweed), and Chenopodium album L. (Pigweed): Three invasive plants. *OPEN VETERINARY JOURNAL*, *12*(4), 584–594. <https://doi.org/10.5455/OVJ.2022.v12.i4.22>

Aylward, F. O., Burnum, K. E., Scott, J. J., Suen, G., Tringe, S. G., Adams, S. M., Barry, K. W., Nicora, C. D., Piehowski, P. D., Purvine, S. O., Starrett, G. J., Goodwin, L. A., Smith, R. D., Lipton, M. S., & Currie, C. R. (2012). Metagenomic and metaproteomic insights into bacterial communities in leaf-cutter ant fungus gardens. *ISME Journal*, *6*(9), 1688–1701. Scopus. <https://doi.org/10.1038/ismej.2012.10>

Aylward, F. O., Burnum-Johnson, K. E., Tringe, S. G., Teiling, C., Tremmel, D. M., Moeller, J. A., Scott, J. J., Barry, K. W., Piehowski, P. D., Nicor, C. D., Malfatti, S. A., Monroe, M. E., Purvine, S. O., Goodwin, L. A., Smith, R. D., Weinstock, G. M., Gerardo, N. M., Suen, G., Lipton, M. S., & Currie, C. R. (2013). Leucoagaricus gongylophorus Produces Diverse Enzymes for the Degradation of Recalcitrant Plant Polymers in Leaf-Cutter Ant Fungus Gardens. *Applied and Environmental Microbiology*, *79*(12), 3770–3778. Scopus. <https://doi.org/10.1128/AEM.03833-12>

Aylward, F. O., Tremmel, D. M., Bruce, D. C., Chain, P., Chen, A., Davenport, K. W., Detter, C., Han, C. S., Han, J., Huntemann, M., Ivanova, N. N., Kyrpides, N. C., Markowitz, V., Mavrommatis, K., Nolan, M., Pagani, I., Pati, A., Pitluck, S., Deshpande, S., … Currie, C. R. (2013). Complete genome of Enterobacteriaceae bacterium strain FGI 57, a strain associated with leaf-cutter ant fungus gardens. *Genome Announcements*, *1*(1). Scopus. <https://doi.org/10.1128/genomeA.00238-12>

Aylward, F. O., Tremmel, D. M., Starrett, G. J., Bruce, D. C., Chain, P., Chen, A., Davenport, K. W., Detter, C., Han, C. S., Han, J., Huntemann, M., Ivanova, N. N., Kyrpides, N. C., Markowitz, V., Mavrommatis, K., Nolan, M., Pagani, I., Pati, A., Pitluck, S., … Currie, C. R. (2013). Complete genome of Serratia sp. Strain FGI 94, a strain associated with leaf-cutter ant fungus gardens. *Genome Announcements*, *1*(2). Scopus. <https://doi.org/10.1128/genomeA.00239-12>

Ba, A. S., Guo, D. A., Norton, R. A., Phillips, S. A., & Nes, W. D. (1995). DEVELOPMENTAL DIFFERENCES IN THE STEROL COMPOSITION OF SOLENOPSIS-INVICTA. *ARCHIVES OF INSECT BIOCHEMISTRY AND PHYSIOLOGY*, *29*(1), 1–9. <https://doi.org/10.1002/arch.940290102>

Ba, A. S., & Phillips Jr, S. A. (1996). Yeast biota of the red imported fire ant. *Mycological Research*, *100*(6), 740–746. Scopus. <https://doi.org/10.1016/S0953-7562(96)80208-5>

Ba, A. S., Phillips, S. A., & Anderson, J. T. (2000). Yeasts in mound soil of the red imported fire ant. *Mycological Research*, *104*(8), 969–973. <https://doi.org/10.1017/S0953756299002385>

Bacci Jr, M., Anversa, M. M., & Pagnocca, F. C. (1995). Cellulose degradation by Leucocoprinus gongylophorus, the fungus cultured by the leaf-cutting ant Atta sexdens rubropilosa. *Antonie van Leeuwenhoek*, *67*(4), 385–386. Scopus. <https://doi.org/10.1007/BF00872939>

Baer, B., & Boomsma, J. J. (2004). Male reproductive investment and queen mating-frequency in fungus-growing ants. *Behavioral Ecology*, *15*(3), 426–432. Scopus. <https://doi.org/10.1093/beheco/arh025>

Baer, B., den Boer, S. P. A., Kronauer, D. J. C., Nash, D. R., & Boomsma, J. J. (2009). Fungus gardens of the leafcutter ant Atta Colombica function as egg nurseries for the snake Leptodeira annulata. *Insectes Sociaux*, *56*(3), 289–291. Scopus. <https://doi.org/10.1007/s00040-009-0026-0>

Baer, B., Dijkstra, M. B., Mueller, U. G., Nash, D. R., & Boomsma, J. J. (2009). Sperm length evolution in the fungus-growing ants. *Behavioral Ecology*, *20*(1), 38–45. Scopus. <https://doi.org/10.1093/beheco/arn112>

Baird, R., Woolfolk, S., & Watson, C. E. (2007). Survey of bacterial and fungal associates of black/hybrid imported fire ants from mounds in Mississippi. *SOUTHEASTERN NATURALIST*, *6*(4), 615–632. [https://doi.org/10.1656/1528-7092(2007)6[615:SOBAFA]2.0.CO;2](https://doi.org/10.1656/1528-7092(2007)6%5b615:SOBAFA%5d2.0.CO;2)

Balafif, F. F., Satari, M. H., & Dhianawaty, D. (2017). Antifungal Activity of Ant Hill Myrmecodia Pendens Water Fraction against Candida Albicans ATCC 10231. *MAJALAH KEDOKTERAN BANDUNG-MKB-BANDUNG MEDICAL JOURNAL*, *49*(1), 28–34. <https://doi.org/10.15395/mkb.v49n1.984>

Bamisile, B. S., Siddiqui, J. A., Nie, L., Idrees, A., Aguila, L. C. R., Jia, C., & Xu, Y. (2023). Baseline Analysis of Endophytic Fungal Associates of Solenopsis invicta Buren from Mounds across Five Counties of Guangdong Province, China. *J Fungi (Basel)*, *9*(3). <https://doi.org/10.3390/jof9030377>

Barbier, D., Papa, J., & Papa, F. (1981). Serologic study of the fungus cultivated by the leaf-cutting ant Acromyrmex octospinosus (Reich) in Guadeloupe. *Bulletin de la Societe de Pathologie Exotique et de ses Filiales*, *74*(2), 164–170. Scopus.

Barbosa, B. C., Halfeld, V. R., de Araújo, J. P. M., Maciel, T. T., & Prezoto, F. (2015). Record of Ophiocordyceps unilateralis sensu lato, the zombie-ant fungus, parasitizing Camponotus in an urban fragment of Atlantic Rainforest in southeastern Brazil. *Studies on Neotropical Fauna and Environment*, *50*(1), 21–23. Scopus. <https://doi.org/10.1080/01650521.2014.991213>

Barke, J., Seipke, R. F., Grüschow, S., Heavens, D., Drou, N., Bibb, M. J., Goss, R. J. M., Yu, D. W., & Hutchings, M. I. (2010). A mixed community of actinomycetes produce multiple antibiotics for the fungus farming ant Acromyrmex octospinosus. *BMC Biology*, *8*. Scopus. <https://doi.org/10.1186/1741-7007-8-109>

Barrett, B. T., Kubik, T. D., Golightly, P. R., Kellner, K., Kardish, M. R., & Mueller, U. G. (2023). Ant genotype, but not genotype of cultivated fungi, predicts queen acceptance in the asexual fungus-farming ant Mycocepurus smithii (Hymenoptera: Formicidae). *Behavioral Ecology and Sociobiology*, *77*(1). Scopus. <https://doi.org/10.1007/s00265-022-03276-5>

Barros, L. A. C., Rabeling, C., Teixeira, G. A., dos Santos Ferreira Mariano, C., Delabie, J. H. C., & de Aguiar, H. J. A. C. (2022). Decay of homologous chromosome pairs and discovery of males in the thelytokous fungus-growing ant Mycocepurus smithii. *Scientific Reports*, *12*(1). Scopus. <https://doi.org/10.1038/s41598-022-08537-x>

Bass, M. (1997). The effects of leaf deprivation on leaf-cutting ants and their mutualistic fungus. *Ecological Entomology*, *22*(4), 384–389. Scopus. <https://doi.org/10.1046/j.1365-2311.1997.00080.x>

Bass, M., & Cherrett, J. M. (1994). The role of leaf‐cutting ant workers (Hymenoptera: Formicidae) in fungus garden maintenance. *Ecological Entomology*, *19*(3), 215–220. Scopus. <https://doi.org/10.1111/j.1365-2311.1994.tb00412.x>

Bass, M., & Cherrett, J. M. (1996a). Fungus garden structure in the leaf-cutting ant Atta sexdens (Formicidae, Attini). *Symbiosis*, *21*(1), 9–24. Scopus.

Bass, M., & Cherrett, J. M. (1996b). Leaf-cutting ants (Formicidae, Attini) prune their fungus to increase and direct its productivity. *Functional Ecology*, *10*(1), 55–61. Scopus. <https://doi.org/10.2307/2390262>

Batey, S. F. D., Greco, C., Hutchings, M. I., & Wilkinson, B. (2020). Chemical warfare between fungus-growing ants and their pathogens. *Current Opinion in Chemical Biology*, *59*, 172–181. Scopus. <https://doi.org/10.1016/j.cbpa.2020.08.001>

Báthori, F., Pfliegler, W. P., Rádai, Z., & Tartally, A. (2018). Host age determines parasite load of Laboulbeniales fungi infecting ants: Implications for host-parasite relationship and fungal life history. *Mycoscience*, *59*(2), 166–171. Scopus. <https://doi.org/10.1016/j.myc.2017.09.004>

Batista, K. O. M., Silva, D. V., Nascimento, V. L., & de Souza, D. J. (2022). Effects of Trichoderma strigosellum in Eucalyptus urophylla Development and Leaf-Cutting Ant Behavior. *JOURNAL OF FUNGI*, *8*(1). <https://doi.org/10.3390/jof8010015>

Bautz, K. R., Caixeta, M. C. S., Del Puppo, N. P., Rodrigues, A., Kloss, T. G., & Elliot, S. L. (2023). Limited impacts of the fungus Syncephalastrum on nests of leaf-cutting ants. *Fungal Ecology*, *62*. Scopus. <https://doi.org/10.1016/j.funeco.2022.101198>

Beattie, A. J., Turnbull, C., Hough, T., Jobson, S., & Knox, R. B. (1985). THE VULNERABILITY OF POLLEN AND FUNGAL SPORES TO ANT SECRETIONS - EVIDENCE AND SOME EVOLUTIONARY IMPLICATIONS. *AMERICAN JOURNAL OF BOTANY*, *72*(4), 606–614. <https://doi.org/10.2307/2443594>

Benfradj, N., Vettraino, A. M., Tomassini, A., Bruni, N., Vannini, A., & Boughalleb-M’Hamdi, N. (2018). Citrus gummosis incidence and role of ants (Lasius grandis) and snails (Helix aspersa) as vectors of the disease in Tunisia. *FOREST PATHOLOGY*, *48*(3). <https://doi.org/10.1111/efp.12423>

Benmoussa, K., Authier, H., Prat, M., AlaEddine, M., Lefèvre, L., Rahabi, M. C., Bernad, J., Aubouy, A., Bonnafé, E., Leprince, J., Pipy, B., Treilhou, M., & Coste, A. (2017). P17, an Original Host Defense Peptide from Ant Venom, Promotes Antifungal Activities of Macrophages through the Induction of C-Type Lectin Receptors Dependent on LTB4-Mediated PPARγ Activation. *FRONTIERS IN IMMUNOLOGY*, *8*. <https://doi.org/10.3389/fimmu.2017.01650>

Bextine, B. R., & Thorvilson, H. G. (2002a). Field applications of bait-formulated Beauveria bassiana alginate pellets for biological control of the red imported fire ant (Hymenoptera: Formicidae). *ENVIRONMENTAL ENTOMOLOGY*, *31*(4), 746–752. <https://doi.org/10.1603/0046-225X-31.4.746>

Bextine, B. R., & Thorvilson, H. G. (2002b). Monitoring Solenopsis invicta (Hymenoptera: Formicidae) foraging with peanut oil-baited, UV-reflective Beauveria bassiana alginate pellets. *SOUTHWESTERN ENTOMOLOGIST*, *27*(1), 31–36.

Bich, G. A., Castrillo, M. L., Villalba, L. L., & Zapata, P. D. (2017). Isolation of the symbiotic fungus of Acromyrmex pubescens and phylogeny of Leucoagaricus gongylophorus from leaf-cutting ants. *Saudi Journal of Biological Sciences*, *24*(4), 851–856. Scopus. <https://doi.org/10.1016/j.sjbs.2016.05.010>

Bieber, A. G. D., Pereira, E. R., & Bottcher, C. (2020). Flower perianth: First observations on a novel diet item for the ponerine ant Odontomachus chelifer(Latreille). *REVISTA BRASILEIRA DE ENTOMOLOGIA*, *64*(3). <https://doi.org/10.1590/1806-9665-RBENT-2020-0019>

Bigi, M. F. M. A., Torkomian, V. L. V., De Groote, S. T. C. S., Hebling, M. J. A., Bueno, O. C., Pagnocca, F. C., Fernandes, J. B., Vieira, P. C., & Da Silva, M. F. G. F. (2004). Activity of Ricinus communis (euphorbiaceae) and ricinine against the leaf-cutting ant Atta sexdens rubropilosa (hymenoptera: Formicidae) and the symbiotic fungus Leucoagaricus gongylophorus. *Pest Management Science*, *60*(9), 933–938. Scopus. <https://doi.org/10.1002/ps.892>

Birnbaum, S. S. L., & Gerardo, N. M. (2016). Patterns of specificity of the pathogen Escovopsis across the fungus-growing ant symbiosis. *American Naturalist*, *188*(1), 52–65. Scopus. <https://doi.org/10.1086/686911>

Bittleston, L. S., Brockmann, F., Wcislo, W., & Van Bael, S. A. (2011). Endophytic fungi reduce leaf-cutting ant damage to seedlings. *Biology Letters*, *7*(1), 30–32. Scopus. <https://doi.org/10.1098/rsbl.2010.0456>

Bizarria, R., de Castro Pietrobon, T., & Rodrigues, A. (2023). Uncovering the Yeast Communities in Fungus-Growing Ant Colonies. *Microbial Ecology*, *86*(1), 624–635. Scopus. <https://doi.org/10.1007/s00248-022-02099-1>

Bizarria, R., Moia, I. C., Montoya, Q. V., Polezel, D. A., & Rodrigues, A. (2018). Soluble Compounds of Filamentous Fungi Harm the Symbiotic Fungus of Leafcutter Ants. *Current Microbiology*, *75*(12), 1602–1608. Scopus. <https://doi.org/10.1007/s00284-018-1566-1>

Blatrix, R., Djiéto-Lordon, C., Mondolot, L., la Fisca, P., Voglmayr, H., & McKey, D. (2012). Plant-ants use symbiotic fungi as a food source: New insight into the nutritional ecology of ant-plant interactions. *Proceedings of the Royal Society B: Biological Sciences*, *279*(1744), 3940–3947. Scopus. <https://doi.org/10.1098/rspb.2012.1403>

Blum, M. S., Brand, J. M., & Amante, E. (1981). o-Aminoacetophenone: Identification in a primitive fungus-growing ant (Mycocepurus goeldii). *Experientia*, *37*(8), 816–817. Scopus. <https://doi.org/10.1007/BF01985656>

Bollazzi, M., Forti, L. C., & Roces, F. (2012). Ventilation of the giant nests of Atta leaf-cutting ants: Does underground circulating air enter the fungus chambers? *Insectes Sociaux*, *59*(4), 487–498. Scopus. <https://doi.org/10.1007/s00040-012-0243-9>

Bollazzi, M., & Roces, F. (2002). Thermal preference for fungus culturing and brood location by workers of the thatching grass-cutting ant Acromyrmex heyeri. *Insectes Sociaux*, *49*(2), 153–157. Scopus. <https://doi.org/10.1007/s00040-002-8295-x>

Bonadies, E., Wcislo, W. T., Gálvez, D., Hughes, W. O. H., & Fernández-Marín, H. (2019). Hygiene defense behaviors used by a fungus-growing ant depend on the fungal pathogen stages. *Insects*, *10*(5). Scopus. <https://doi.org/10.3390/insects10050130>

Borba, R. D. S., Loeck, A. E., Branco, J. S. C., Bonow, J., & De Oliveira, A. C. (2008). Fusion of fungi cultivated by different species of cutter ants in Rio Grande do Sul, Brazil. *Ciencia Rural*, *38*(5), 1214–1219. Scopus. <https://doi.org/10.1590/S0103-84782008000500002>

Borba, R. D. S., Loeck, A. E., Branco, J. S. C., Kopp, M. M., & De Oliveira, A. C. (2007). Polymorphism of the cutting ants symbiotic fungus submitted to ultraviolet light. *Ciencia Rural*, *37*(5), 1221–1226. Scopus. <https://doi.org/10.1590/s0103-84782007000500001>

Bordoni, A., Dapporto, L., Tatini, I., Celli, M., Bercigli, M., Barrufet, S. R., Perito, B., & Turillazzi, S. (2018). Trans-generational immunization in the acrobat ant Crematogaster scutellaris. *BIOLOGY LETTERS*, *14*(4). <https://doi.org/10.1098/rsbl.2017.0761>

Bordoni, A., Matejkova, Z., Chimenti, L., Massai, L., Perito, B., Dapporto, L., & Turillazzi, S. (2019). Home economics in an oak gall: Behavioural and chemical immune strategies against a fungal pathogen in Temnothorax ant nests. *SCIENCE OF NATURE*, *106*(11–12). <https://doi.org/10.1007/s00114-019-1659-0>

Bordoni, A., Miroddi, M. A., Dapporto, L., & Turillazzi, S. (2017). Long-term assessment reveals the hidden and hiding effects of experimental stress on ant colonies. *BEHAVIORAL ECOLOGY AND SOCIOBIOLOGY*, *71*(10). <https://doi.org/10.1007/s00265-017-2373-6>

Bos, N., Kankaanpää-Kukkonen, V., Freitak, D., Stucki, D., & Sundström, L. (2019). Comparison of Twelve Ant Species and Their Susceptibility to Fungal Infection. *INSECTS*, *10*(9). <https://doi.org/10.3390/insects10090271>

Bos, N., Lefèvre, T., Jensen, A. B., & d’Ettorre, P. (2012). Sick ants become unsociable. *JOURNAL OF EVOLUTIONARY BIOLOGY*, *25*(2), 342–351. <https://doi.org/10.1111/j.1420-9101.2011.02425.x>

Bot, A. N. M., Ortius-Lechner, D., Finster, K., Maile, R., & Boomsma, J. J. (2002). Variable sensitivity of fungi and bacteria to compounds produced by the metapleural glands of leaf-cutting ants. *Insectes Sociaux*, *49*(4), 363–370. Scopus. <https://doi.org/10.1007/PL00012660>

Bot, A. N. M., Rehner, S. A., & Boomsma, J. J. (2001). Partial incompatibility between ants and symbiotic fungi in two sympatric species of Acromyrmex leaf-cutting ants. *Evolution*, *55*(10), 1980–1991. Scopus. <https://doi.org/10.1111/j.0014-3820.2001.tb01315.x>

Bouaziz, A., Walgraffe, D., Bouillot, C., Herman, J., Foguenne, J., Gothot, A., Louis, R., Hentges, F., Jacquet, A., Mailleux, A. C., Chevigné, A., Galleni, M., Adam, E., & Dumez, M. E. (2015). Development of recombinant stable house dust mite allergen Der p 3 molecules for component-resolved diagnosis and specific immunotherapy. *CLINICAL AND EXPERIMENTAL ALLERGY*, *45*(4), 823–834. <https://doi.org/10.1111/cea.12452>

Boya, C. A., Fernández-Marín, H., Mejiá, L. C., Spadafora, C., Dorrestein, P. C., & Gutiérrez, M. (2017). Imaging mass spectrometry and MS/MS molecular networking reveals chemical interactions among cuticular bacteria and pathogenic fungi associated with fungus-growing ants. *Scientific Reports*, *7*(1). Scopus. <https://doi.org/10.1038/s41598-017-05515-6>

Boyd, N. D., & Martin, M. M. (1975a). Faecal proteinases of the fungus-growing ant, Atta texana: Properties, significance and possible origin. *Insect Biochemistry*, *5*(5), 619–635. Scopus. <https://doi.org/10.1016/0020-1790(75)90043-8>

Boyd, N. D., & Martin, M. M. (1975b). Faecal proteinases of the fungus-growing ant, Atta texana: Their fungal origin and ecological significance. *Journal of Insect Physiology*, *21*(11), 1815–1820. Scopus. <https://doi.org/10.1016/0022-1910(75)90247-4>

Brand, J. M., Cruden, D. L., & Markovetz, A. J. (1987). SYNTHESIS OF ENANTIOMERICALLY ENRICHED 2-HEPTANOL AND 3-OCTANOL BY MICROBIAL REDUCTASES OF CURVULARIA-FALCATA AND MUCOR SPECIES. *JOURNAL OF CHEMICAL ECOLOGY*, *13*(2), 357–361. <https://doi.org/10.1007/BF01025895>

Brandão, C. R. F., & Mayhé-Nunes, A. J. (2001). A new fungus-growing ant genus, Mycetagroicus gen. N., with the description of three new species and comments on the monophyly of the attini (Hymenoptera: Formicidae). *Sociobiology*, *38*(3 B), 639–665. Scopus.

Brandão, C. R. F., & Mayhé-Nunes, A. J. (2008). A new species of the fungus-farming ant genus Mycetagroicus Brandão & Mayhé-Nunes (Hymenoptera, Formicidae, Attini). *Revista Brasileira de Entomologia*, *52*(3), 349–352. Scopus. <https://doi.org/10.1590/S0085-56262008000300006>

Brennan, M., Thomas, D. Y., Whiteway, M., & Kavanagh, K. (2002). Correlation between virulence of Candida albicans mutants in mice and Galleria mellonella larvae. *FEMS IMMUNOLOGY AND MEDICAL MICROBIOLOGY*, *34*(2), 153–157. <https://doi.org/10.1111/j.1574-695X.2002.tb00617.x>

Brennan, R. J., Kandikonda, S., Khrimian, A. P., DeMilo, A. B., Liquido, N. J., & Schiestl, R. H. (1996). Saturated and monofluoro analogs of the oriental fruit fly attractant methyl eugenol show reduced genotoxic activities in yeast. *MUTATION RESEARCH-GENETIC TOXICOLOGY*, *369*(3–4), 175–181. <https://doi.org/10.1016/S0165-1218(96)90024-5>

Bresinsky, A. (2014). Ants, Plants and Fungi: A View on Some Patterns of Interaction and Diversity. In U. Luttge, W. Beyschlag, & J. Cushman (Eds.), *PROGRESS IN BOTANY 75* (WOS:000327317500001; Vol. 75, pp. 3–54). <https://doi.org/10.1007/978-3-642-38797-5_1>

Bringhurst, B., Allert, M., Greenwold, M., Kellner, K., & Seal, J. N. (2023). Environments and Hosts Structure the Bacterial Microbiomes of Fungus-Gardening Ants and their Symbiotic Fungus Gardens. *Microbial Ecology*, *86*(2), 1374–1392. Scopus. <https://doi.org/10.1007/s00248-022-02138-x>

Bringhurst, B., Greenwold, M., Kellner, K., & Seal, J. N. (2024). Symbiosis, dysbiosis and the impact of horizontal exchange on bacterial microbiomes in higher fungus-gardening ants. *Scientific Reports*, *14*(1). Scopus. <https://doi.org/10.1038/s41598-024-53218-6>

Brinkman, M. A., & Gardner, W. A. (2000). Enhanced activity of Beauveria bassiana to red imported fire ant workers (Hymenoptera: Formicidae) infected with Thelohania solenopsae. *JOURNAL OF AGRICULTURAL AND URBAN ENTOMOLOGY*, *17*(4), 191–195.

Bruner, G., Fernández-Marín, H., Touchon, J. C., & Wcislo, W. T. (2012). Eggs of the blind snake, Liotyphlops albirostris, are incubated in a nest of the lower fungus-growing ant, Apterostigma cf. Goniodes. *Psyche (London)*. Scopus. <https://doi.org/10.1155/2012/532314>

Bruner, G., Wcislo, W. T., & Fernández-Marín, H. (2014). Prudent inquilines and proactive hosts: Behavioral dynamics between an ant social parasite, Megalomyrmex symmetochus and its fungus-growing ant host, Sericomyrmex amabilis. *Insectes Sociaux*, *61*(1), 83–88. Scopus. <https://doi.org/10.1007/s00040-013-0331-5>

Bruner-Montero, G., Wood, M., Horn, H. A., Gemperline, E., Li, L., & Currie, C. R. (2021). Symbiont-Mediated Protection of Acromyrmex Leaf-Cutter Ants from the Entomopathogenic Fungus Metarhizium anisopliae. *mBio*, *12*(6). Scopus. <https://doi.org/10.1128/mBio.01885-21>

Brütsch, T., & Chapuisat, M. (2014). Wood ants protect their brood with tree resin. *ANIMAL BEHAVIOUR*, *93*, 157–161. <https://doi.org/10.1016/j.anbehav.2014.04.024>

Brütsch, T., Felden, A., Reber, A., & Chapuisat, M. (2014). Ant queens (Hymenoptera: Formicidae) are attracted to fungal pathogens during the initial stage of colony founding. *MYRMECOLOGICAL NEWS*, *20*, 71–76.

Brütsch, T., Jaffuel, G., Vallat, A., Turlings, T. C. J., & Chapuisat, M. (2017). Wood ants produce a potent antimicrobial agent by applying formic acid on tree-collected resin. *ECOLOGY AND EVOLUTION*, *7*(7), 2249–2254. <https://doi.org/10.1002/ece3.2834>

Buren, W. F. (1944). A New Fungus Growing Ant from Mexico. *Psyche (New York)*, *51*(1–2), 5–7. Scopus. <https://doi.org/10.1155/1944/96127>

Cafaro, M. J., & Currie, C. R. (2005). Phylogenetic analysis of mutualistic filamentous bacteria associated with fungus-growing ants. *Canadian Journal of Microbiology*, *51*(6), 441–446. Scopus. <https://doi.org/10.1139/w05-023>

Cafaro, M. J., Poulsen, M., Little, A. E. F., Price, S. L., Gerardo, N. M., Wong, B., Stuart, A. E., Larget, B., Abbot, P., & Currie, C. R. (2011). Specificity in the symbiotic association between fungus-growing ants and protective Pseudonocardia bacteria. *Proceedings of the Royal Society B: Biological Sciences*, *278*(1713), 1814–1822. Scopus. <https://doi.org/10.1098/rspb.2010.2118>

Camargo, R. S., Forti, L. C., De Melo Rocha, M., De Matos, C. A. O., Lopes, J. F., De Andrade, A. P. P., & Verza, S. S. (2003). The effect of plant diversity on fungus garden development and foraging behavior of leaf-cutting ants (Hymenoptera: Formicidae). *Sociobiology*, *42*(2), 359–368. Scopus.

Camargo, R. S., Lopes, J. F. S., & Forti, L. C. (2013). Fungus garden acts as a template for the construction of chambers in ants? *Ciencia Rural*, *43*(4), 565–570. Scopus. <https://doi.org/10.1590/S0103-84782013005000017>

Camargo, T. S., Nickele, M. A., Filho, W. R., do Rocio Chiarello Penteado, S., de Queiroz, E. C., & Auer, C. G. (2023). Fungal Community Associated with the Leaf-Cutting Ant Acromyrmex crassispinus (Hymenoptera: Formicidae) Colonies: A Search for Potential Biocontrol Agents. *Microb Ecol*, *86*(2), 1281–1291. <https://doi.org/10.1007/s00248-023-02217-7>

Campanella, D. M., McEvoy, P. B., & Mundt, C. C. (2009). Interaction effects of two biological control organisms on resistant and susceptible weed biotypes of Chondrilla juncea in western North America. *BIOLOGICAL CONTROL*, *50*(1), 50–59. <https://doi.org/10.1016/j.biocontrol.2009.01.005>

Cardoso, D. C., Cristiano, M. P., da Costa-Milanez, C. B., & Heinze, J. (2016). Agro-predation by Megalomyrmex ants on Mycetophylax fungus-growing ants. *Insectes Sociaux*, *63*(3), 483–486. Scopus. <https://doi.org/10.1007/s00040-016-0487-x>

Cardoso, D. C., Heinze, J., Moura, M. N., & Cristiano, M. P. (2018). Chromosomal variation among populations of a fungus-farming ant: Implications for karyotype evolution and potential restriction to gene flow. *BMC Evolutionary Biology*, *18*(1). Scopus. <https://doi.org/10.1186/s12862-018-1247-5>

Cardoso Neto, J. A., Leal, L. C., & Baccaro, F. B. (2019). Temporal and spatial gradients of humidity shape the occurrence and the behavioral manipulation of ants infected by entomopathogenic fungi in Central Amazon. *Fungal Ecology*, *42*. Scopus. <https://doi.org/10.1016/j.funeco.2019.100871>

Cardoso, S. R. S., Nagamoto, N. S., Forti, L. C., & Souza, E. S. (2012). Carrying and Effect of Granulated Baits Formulated with Entomopathogenic Fungi among Atta sexdens rubropilosa Colonies (Hymenoptera: Formicidae). *SOCIOBIOLOGY*, *59*(3), 681–689.

Cardoso, S. R. S., Rodrigues, A., Forti, L. C., & Nagamoto, N. S. (2022). Pathogenicity of filamentous fungi towards Atta sexdens rubropilosa (Hymenoptera: Formicidae). *INTERNATIONAL JOURNAL OF TROPICAL INSECT SCIENCE*, *42*(2), 1215–1223. <https://doi.org/10.1007/s42690-021-00640-2>

Carlos, A. A., Forti, L. C., Rodrigues, A., Verza, S. S., Nagamoto, N. S., & Camargo, R. S. (2009). Influence of Fungal Contamination on Substrate Carrying by the Leafcutter Ant Atta sexdens rubropilosa (Hymenoptera: Formicidae). *SOCIOBIOLOGY*, *53*(3), 785–794.

Carlos, A. A., Rodrigues, A., Forti, L. C., Passador, M. M., & Sierra, J. F. (2011). Filamentous fungi found in Atta sexdens rubropilosa colonies after treatment with different toxic bait formulations. *JOURNAL OF APPLIED ENTOMOLOGY*, *135*(4), 326–331. <https://doi.org/10.1111/j.1439-0418.2010.01551.x>

Carlson, A. L., Ishak, H. D., Kurian, J., Mikheyev, A. S., Gifford, I., & Mueller, U. G. (2017). Nuclear populations of the multinucleate fungus of leafcutter ants can be dekaryotized and recombined to manipulate growth of nutritive hyphal nodules harvested by the ants. *Mycologia*, *109*(5), 832–846. Scopus. <https://doi.org/10.1080/00275514.2017.1400304>

Carr, G., Derbyshire, E. R., Caldera, E., Currie, C. R., & Clardy, J. (2012). Antibiotic and antimalarial quinones from fungus-growing ant-associated pseudonocardia sp. *Journal of Natural Products*, *75*(10), 1806–1809. Scopus. <https://doi.org/10.1021/np300380t>

Carreiro, S. C., Pagnocca, F. C., Bacci, M., Bueno, O. C., Hebling, M. J. A., & Middelhoven, W. J. (2002). Occurrence of killer yeasts in leaf-cutting ant nests. *FOLIA MICROBIOLOGICA*, *47*(3), 259–262. <https://doi.org/10.1007/BF02817648>

Carreiro, S. C., Pagnocca, F. C., Bueno, O. C., Bacci, M. B., Hebling, M. J. A., & daSilva, O. A. (1997). Yeasts associated with nests of the leaf-cutting ant Atta sexdens rubropilosa Forel, 1908. *ANTONIE VAN LEEUWENHOEK INTERNATIONAL JOURNAL OF GENERAL AND MOLECULAR MICROBIOLOGY*, *71*(3), 243–248. <https://doi.org/10.1023/A:1000182108648>

Carver, M., Inkerman, P. A., & Ashbolt, N. J. (1987). ANAGYRUS-SACCHARICOLA TIMBERLAKE (HYMENOPTERA, ENCYRTIDAE) AND OTHER BIOTA ASSOCIATED WITH SACCHARICOCCUS-SACCHARI (COCKERELL) (HOMOPTERA, PSEUDOCOCCIDAE) IN AUSTRALIA. *JOURNAL OF THE AUSTRALIAN ENTOMOLOGICAL SOCIETY*, *26*, 367–368.

Castanõ-Meneses, G., Palacios-Vargas, J. G., Delabie, J. H. C., Zeppelini, D., & Mariano, C. S. F. (2017). Springtails (Collembola) Associated with Nests of Fungus-Growing Ants (Formicidae: Myrmicinae: Attini) in Southern Bahia, Brazil. *Florida Entomologist*, *100*(4), 740–742. Scopus. <https://doi.org/10.1653/024.100.0421>

Castella, G., Chapuisat, M., & Christe, P. (2008). Prophylaxis with resin in wood ants. *ANIMAL BEHAVIOUR*, *75*, 1591–1596. <https://doi.org/10.1016/j.anbehav.2007.10.014>

Castellani, M. A., Forti, L. C., Fenille, R. C., Raetano, C. G., Moreira, A. A., Andrade, A. P. P., Camargo, R. S., Lemos, R. N. S., Aguiar, A. G., & Nagamoto, N. S. (2009). Growth of the symbiotic fungus of the grass-cutting ant atta capiguara (Hymenoptera: Formicidae): Effect of grass extracts. *Sociobiology*, *54*(1), 283–298. Scopus.

Castilho, A. M. C., Fraga, M. E., Aguiar-Menezes, E. D., & Rosa, C. A. D. (2010). Selection of Metarhizium anisopliae and Beauveria bassiana isolates pathogenic to Atta bisphaerica and Atta sexdens rubropilosa soldiers under laboratory conditions. *CIENCIA RURAL*, *40*(6), 1243–1249. <https://doi.org/10.1590/S0103-84782010005000100>

Castrillo, M. L., Bich, G. A., Zapata, P. D., & Villalba, L. L. (2016). Biocontrol of Leucoagaricus gongylophorus of leaf-cutting ants with the mycoparasitic agent Trichoderma koningiopsis. *MYCOSPHERE*, *7*(6), 810–819. <https://doi.org/10.5943/mycosphere/7/6/12>

Catalani, G. C., Camargo, R. S., Sousa, K. K. A., Caldato, N., Silva, A. A. C., & Forti, L. C. (2020). Fat-Soluble Substance Flow During Symbiotic Fungus Cultivation by Leaf-Cutter Ants. *Neotropical Entomology*, *49*(1), 116–123. Scopus. <https://doi.org/10.1007/s13744-019-00718-0>

Catalani, G. C., Sousa, K. K. A., Camargo, R. S. D., Caldato, N., Matos, C. A. O., & Forti, L. C. (2019). Chemical control of leaf-cutting ants: How do workers disperse toxic bait fragments onto fungus garden? *Revista Brasileira de Entomologia*, *63*(4), 290–295. Scopus. <https://doi.org/10.1016/j.rbe.2019.09.004>

Cattaneo, A. M., Witzgall, P., Kwadha, C. A., Becher, P. G., & Walker, W. B., III. (2023). Heterologous expression and functional characterization of Drosophila suzukii OR69a transcript variants unveiled response to kairomones and to a candidate pheromone. *JOURNAL OF PEST SCIENCE*, *96*(3), 1149–1171. <https://doi.org/10.1007/s10340-022-01585-2>

Cazin Jr, J., Wiemer, D. F., & Howard, J. J. (1989). Isolation, growth characteristics, and long-term storage of fungi cultivated by attine ants. *Applied and Environmental Microbiology*, *55*(6), 1346–1350. Scopus. <https://doi.org/10.1128/aem.55.6.1346-1350.1989>

Cedeno, A. (1989). Terpenoids and the leaf-cutting ant-fungus mutualism. *Ecotropicos*, *2*(2), 73–79. Scopus.

Chapela, I. H., Rehner, S. A., Schultz, T. R., & Mueller, U. G. (1994). Evolutionary history of the symbiosis between fungus-growing ants and their fungi. *Science*, *266*(5191), 1691–1694. Scopus.

Chapuisat, M., Oppliger, A., Magliano, P., & Christe, P. (2007). Wood ants use resin to protect themselves against pathogens. *PROCEEDINGS OF THE ROYAL SOCIETY B-BIOLOGICAL SCIENCES*, *274*(1621), 2013–2017. <https://doi.org/10.1098/rspb.2007.0531>

Chatterjee, A., Valasubramanian, R., Ma, W. L., Vachhani, A. K., Gnanamanickam, S., & Chatterjee, A. K. (1996). Isolation of ant mutants of Pseudomonas fluorescens strain Pf7-14 altered in antibiotic production, cloning of ant(+) DNA, and evaluation of the role of antibiotic production in the control of blast and sheath blight of rice. *BIOLOGICAL CONTROL*, *7*(2), 185–195. <https://doi.org/10.1006/bcon.1996.0083>

Chen, Y., & Zhou, S. Y. (2017). Phylogenetic Relationships Based on DNA Barcoding Among 16 Species of the Ant Genus Formica (Hymenoptera: Formicidae) from China. *JOURNAL OF INSECT SCIENCE*, *17*(6). <https://doi.org/10.1093/jisesa/iex092>

Christopher, Y., Aguilar, C., Gálvez, D., Wcislo, W. T., Gerardo, N. M., & Fernández-Marín, H. (2021). Interactions among escovopsis, antagonistic microfungi associated with the fungus-growing ant symbiosis. *Journal of Fungi*, *7*(12). Scopus. <https://doi.org/10.3390/jof7121007>

Christopher, Y., Wcislo, W. T., Martínez-Luis, S., Hughes, W. O. H., Gerardo, N. M., & Fernández-Marín, H. (2021). Disease management in two sympatric Apterostigma fungus-growing ants for controlling the parasitic fungus Escovopsis. *Ecology and Evolution*, *11*(11), 6041–6052. Scopus. <https://doi.org/10.1002/ece3.7379>

Coblentz, K. E., & Van Bael, S. A. (2013). Field colonies of leaf-cutting ants select plant materials containing low abundances of endophytic fungi. *Ecosphere*, *4*(5). Scopus. <https://doi.org/10.1890/ES13-00012.1>

Cole Jr, A. C. (1939). The life history of a fungus-growing ant of the Mississippi Gulf Coast. *Lloydia*, *2*(2), 153–160. Scopus.

Correa, K. C. S., Facchinatto, W. M., Habitzreuter, F. B., Ribeiro, G. H., Rodrigues, L. G., Micocci, K. C., Campana, S. P., Colnago, L. A., & Souza, D. H. F. (2024). Activity of a Recombinant Chitinase of the Atta sexdens Ant on Different Forms of Chitin and Its Fungicidal Effect against Lasiodiplodia theobromae. *POLYMERS*, *16*(4). <https://doi.org/10.3390/polym16040529>

Costello, M. J., & Welch, M. D. (2014). Influence of Weeds on Argentine Ant (Hymenoptera: Formicidae) and Obscure Mealybug (Hemiptera: Pseudococcidae) in a Central California Vineyard. *JOURNAL OF ECONOMIC ENTOMOLOGY*, *107*(3), 1194–1200. <https://doi.org/10.1603/EC13469>

Cotazo-Calambas, K. M., Niño-Castro, A., Valencia-Giraldo, S. M., Gómez-Díaz, J. S., & Montoya-Lerma, J. (2022). Behavioral Response of the Leaf-Cutting Ant Atta cephalotes (Hymenoptera: Formicidae) to Trichoderma sp. *JOURNAL OF INSECT BEHAVIOR*, *35*(4), 92–102. <https://doi.org/10.1007/s10905-022-09800-9>

Couceiro, J. D. C., Marcelino, W. L., Amaral, K. D., Gandra, L. C., de Souza, D. J., & Della Lucia, T. M. C. (2016). Effects of entomopathogenic fungi on the mortality and immune system of the leaf-cutting ant Acromyrmex subterraneus subterraneus. *Entomologia Experimentalis et Applicata*, *161*(2), 152–159. Scopus. <https://doi.org/10.1111/eea.12500>

Cristopher, A. B. P., Christian, M. H., Fernandez-Marin, H., & Gutierrez, M. (2019). Fungus-growing ant’s microbial interaction of streptomyces sp. And escovopsis sp. Through molecular networking and MALDI imaging. *Natural Product Communications*, *14*(1), 63–66. Scopus. <https://doi.org/10.1177/1934578X1901400117>

Cross, J., Fountain, M., Markó, V., & Nagy, C. (2015). Arthropod ecosystem services in apple orchards and their economic benefits. *ECOLOGICAL ENTOMOLOGY*, *40*, 82–96. <https://doi.org/10.1111/een.12234>

Crumière, A. J. J., Mallett, S., Michelsen, A., Rinnan, R., & Shik, J. Z. (2022). Nutritional challenges of feeding a mutualist: Testing for a nutrient–toxin tradeoff in fungus-farming leafcutter ants. *Ecology*, *103*(6). Scopus. <https://doi.org/10.1002/ecy.3684>

Csata, E., Billen, J., Barbu-Tudoran, L., & Markó, B. (2021). Inside Pandora’s box: Development of the lethal myrmecopathogenic fungus Pandora formicae within its ant host. *Fungal Ecology*, *50*. Scopus. <https://doi.org/10.1016/j.funeco.2020.101022>

Csata, E., Erős, K., & Markó, B. (2014). Effects of the ectoparasitic fungus Rickia wasmannii on its ant host Myrmica scabrinodis: Changes in host mortality and behavior. *Insectes Sociaux*, *61*(3), 247–252. Scopus. <https://doi.org/10.1007/s00040-014-0349-3>

Csata, E., Pérez-Escudero, A., Laury, E., Leitner, H., Latil, G., Heinze, J., Simpson, S. J., Cremer, S., & Dussutour, A. (2024). Fungal infection alters collective nutritional intake of ant colonies. *CURRENT BIOLOGY*, *34*(4). <https://doi.org/10.1016/j.cub.2024.01.017>

Csősz, S., Rádai, Z., Tartally, A., Ballai, L. E., & Báthori, F. (2021). Ectoparasitic fungi Rickia wasmannii infection is associated with smaller body size in Myrmica ants. *Scientific Reports*, *11*(1). Scopus. <https://doi.org/10.1038/s41598-021-93583-0>

Currie, C. R., Mueller, U. G., & Malloch, D. (1999). The agricultural pathology of ant fungus gardens. *Proceedings of the National Academy of Sciences of the United States of America*, *96*(14), 7998–8002. Scopus. <https://doi.org/10.1073/pnas.96.14.7998>

Currie, C. R., Poulsen, M., Mendenhall, J., Boomsma, J. J., & Billen, J. (2006). Coevolved crypts and exocrine glands support mutualistic bacteria in fungus-growing ants. *Science*, *311*(5757), 81–83. Scopus. <https://doi.org/10.1126/science.1119744>

Currie, C. R., Scottt, J. A., Summerbell, R. C., & Malloch, D. (1999). Fungus-growing ants use antibiotic-producing bacteria to control garden parasites. *Nature*, *398*(6729), 701–704. Scopus. <https://doi.org/10.1038/19519>

Da Silva Borba, R., Loeck, A. E., De Magalhães Bandeira, J., Moraes, C. L., & Centenaro, E. D. (2006). Growth of symbiont fungi of cutter ants of the genus Acromyrmex in means of culture with different extracts. *Ciencia Rural*, *36*(3), 725–730. Scopus. <https://doi.org/10.1590/S0103-84782006000300002>

da Silva Camargo, R., & Forti, L. C. (2015). What is the stimulus for the excavation of fungus chamber in leaf-cutting ants? *Acta Ethologica*, *18*(1), 31–35. Scopus. <https://doi.org/10.1007/s10211-014-0181-9>

da Silva, D. G., Lucena, A., Sarmento, R. D., Silva, C. L. S., Tenório, A. C. T., de Souza, D. J., & Melo, M. S. (2020). Encapsulated baits containing zinc sulfate and Trichoderma harzianum reduce fungal garden in Atta sexdens colonies. *REVISTA DE AGRICULTURA NEOTROPICAL*, *7*(3), 46–52. <https://doi.org/10.32404/rean.v7i3.5064>

da Silva, E. A., Paludo, C. R., Valadares, L., Lopes, N. P., do Nascimento, F. S., & Pupo, M. T. (2017). Aflatoxins produced by Aspergillus nomius ASR3, a pathogen isolated from the leaf-cutter ant Atta sexdens rubropilosa. *REVISTA BRASILEIRA DE FARMACOGNOSIA-BRAZILIAN JOURNAL OF PHARMACOGNOSY*, *27*(4), 529–532. <https://doi.org/10.1016/j.bjp.2017.05.001>

Dai, L. Y., Jacob, M. R., Khan, S. I., Khan, I. A., Cark, A. M., & Li, X. C. (2011). Synthesis and Antifungal Activity of Natural Product-Based 6-Alkyl-2,3,4,5-tetrahydropyridines. *JOURNAL OF NATURAL PRODUCTS*, *74*(9), 2023–2026. <https://doi.org/10.1021/np200644s>

Dallongeville, A., Le Cann, P., Zmirou-Navier, D., Chevrier, C., Costet, N., Annesi-Maesano, I., & Blanchard, O. (2015). Concentration and determinants of molds and allergens in indoor air and house dust of French dwellings. *SCIENCE OF THE TOTAL ENVIRONMENT*, *536*, 964–972. <https://doi.org/10.1016/j.scitotenv.2015.06.039>

Dângelo, R. A. C., de Souza, D. J., Mendes, T. D., Couceiro, J. C., & Lucia, T. M. C. D. (2016). Actinomycetes inhibit filamentous fungi from the cuticle of Acromyrmex leafcutter ants. *Journal of Basic Microbiology*, *56*(3), 229–237. Scopus. <https://doi.org/10.1002/jobm.201500593>

Davidson, D. W., Anderson, N. F., Cook, S. C., Bernau, C. R., Jones, T. H., Kamariah, A. S., Lim, L. B., Chan, C. M., & Clark, D. A. (2009). An Experimental Study of Microbial Nest Associates of Borneo’s Exploding Ants (Camponotus [Colobopsis] species). *JOURNAL OF HYMENOPTERA RESEARCH*, *18*(2), 341–360.

Dawadi, S., Baysal-Gurel, F., Addesso, K. M., Liyanapathiranage, P., & Simmons, T. (2021). Fire Ant Venom Alkaloids: Possible Control Measure for Soilborne and Foliar Plant Pathogens. *PATHOGENS*, *10*(6). <https://doi.org/10.3390/pathogens10060659>

Daza, F. F. F., Roman, G. R., Rodriguez, M. V., Vargas, I. A. G., Heano, H. C., Cereda, M. P., & Mulet, R. A. C. (2019). Spores of Beauveria bassiana and Trichoderma lignorum as a bioinsecticide for the control of Atta cephalotes. *BIOLOGICAL RESEARCH*, *52*(1). <https://doi.org/10.1186/s40659-019-0259-y>

de Bekker, C., & Das, B. (2022). Hijacking time: How Ophiocordyceps fungi could be using ant host clocks to manipulate behavior. *Parasite Immunology*, *44*(3). Scopus. <https://doi.org/10.1111/pim.12909>

de Benedictis, F. M., Franceschini, F., Hill, D., Naspitz, C., Simons, F. E. R., Wahn, U., Warner, J. O., de Longueville, M., & Grp, E. S. (2009). The allergic sensitization in infants with atopic eczema from different countries. *ALLERGY*, *64*(2), 295–303. <https://doi.org/10.1111/j.1398-9995.2008.01779.x>

De Fine Licht, H. H., & Boomsma, J. J. (2010). Forage collection, substrate preparation, and diet composition in fungus-growing ants. *Ecological Entomology*, *35*(3), 259–269. Scopus. <https://doi.org/10.1111/j.1365-2311.2010.01193.x>

De Fine Licht, H. H., & Boomsma, J. J. (2014). Variable interaction specificity and symbiont performance in Panamanian Trachymyrmex and Sericomyrmex fungus-growing ants. *BMC Evolutionary Biology*, *14*(1). Scopus. <https://doi.org/10.1186/s12862-014-0244-6>

De Fine Licht, H. H., Schiøtt, M., Mueller, U. G., & Boomsma, J. J. (2010). Evolutionary transitions in enzyme activity of ant fungus gardens. *Evolution*, *64*(7), 2055–2069. Scopus. <https://doi.org/10.1111/j.1558-5646.2010.00948.x>

De Fine Licht, H. H., Schitøt, M., Rogowska-Wrzesinska, A., Nygaard, S., Roepstorff, P., & Boomsma, J. J. (2013). Laccase detoxification mediates the nutritional alliance between leaf-cutting ants and fungus-garden symbionts. *Proceedings of the National Academy of Sciences of the United States of America*, *110*(2), 583–587. Scopus. <https://doi.org/10.1073/pnas.1212709110>

de Jesus, M. S., Rodrigues, W. C., Barbosa, G., Trilles, L., Wanke, B., Lazera, M. D., & da Silva, M. (2012). Cryptococcus neoformans carried by Odontomachus bauri ants. *MEMORIAS DO INSTITUTO OSWALDO CRUZ*, *107*(4), 466–469. <https://doi.org/10.1590/S0074-02762012000400004>

De, K., & Pande, Y. D. (1988). BIONOMICS AND SOME BEHAVIORAL-ASPECTS OF THE MANGO STONE WEEVIL, STERNOCHETUS-GRAVIS (FABRICIUS) (COLEOPTERA, CURCULIONIDAE). *ENTOMON*, *13*(1), 17–24.

De Mana, T. J. B., Stajich, J. E., Kubicek, C. P., Teiling, C., Chenthamara, K., Atanasova, L., Druzhinina, I. S., Levenkova, N., Birnbaum, S. S. L., Barribeau, S. M., Bozick, B. A., Suen, G., Currie, C. R., & Gerardo, N. M. (2016). Small genome of the fungus Escovopsis weberi, a specialized disease agent of ant agriculture. *Proceedings of the National Academy of Sciences of the United States of America*, *113*(13), 3567–3572. Scopus. <https://doi.org/10.1073/pnas.1518501113>

De Zarzuela, M. F. M., Campos-Farinha, A. E. D. C., Russomanno, O. M. R., Kruppa, P. C., & Gonçalez, E. (2007). Evaluation of urban ants (hymenoptera: Formicidae) as vectors of microorganisms in residential and industrial environments: II. Fungi. *Sociobiology*, *50*(2), 653–658. Scopus.

Defossez, E., Selosse, M. A., Dubois, M. P., Mondolot, L., Faccio, A., Djieto-Lordon, C., McKey, D., & Blatrix, R. (2009). Ant-plants and fungi: A new threeway symbiosis. *New Phytologist*, *182*(4), 942–949. Scopus. <https://doi.org/10.1111/j.1469-8137.2009.02793.x>

Dejean, A., Azémar, F., Naskrecki, P., Tindo, M., Rossi, V., Faucher, C., & Gryta, H. (2023). Mutualistic interactions between ants and fungi: A review. *Ecology and Evolution*, *13*(8). Scopus. <https://doi.org/10.1002/ece3.10386>

Detrain, C., & Leclerc, J. B. (2022). Spatial distancing by fungus-exposed Myrmica ants is prompted by sickness rather than contagiousness. *Journal of Insect Physiology*, *139*. Scopus. <https://doi.org/10.1016/j.jinsphys.2022.104384>

D’Ettorre, P., Mora, P., Dibangou, V., Rouland, C., & Errard, C. (2002). The role of the symbiotic fungus in the digestive metabolism of two species of fungus-growing ants. *Journal of Comparative Physiology B: Biochemical, Systemic, and Environmental Physiology*, *172*(2), 169–176. Scopus. <https://doi.org/10.1007/s00360-001-0241-0>

Dhodary, B., Schilg, M., Wirth, R., & Spiteller, D. (2018). Secondary Metabolites from Escovopsis weberi and Their Role in Attacking the Garden Fungus of Leaf-Cutting Ants. *Chemistry - A European Journal*, *24*(17), 4445–4452. Scopus. <https://doi.org/10.1002/chem.201706071>

Diaz Napal, G. N., Buffa, L. M., Nolli, L. C., Defagó, M. T., Valladares, G. R., Carpinella, M. C., Ruiz, G., & Palacios, S. M. (2015). Screening of native plants from central Argentina against the leaf-cutting ant Acromyrmex lundi (Guérin) and its symbiotic fungus. *Industrial Crops and Products*, *76*, 275–280. Scopus. <https://doi.org/10.1016/j.indcrop.2015.07.001>

Diehl-Fleig, E., & Diehl, E. (2007). Nest architecture and colony size of the fungus-growing ant Mycetophylax simplex Emery, 1888 (Formicidae, Attini). *Insectes Sociaux*, *54*(3), 242–247. Scopus. <https://doi.org/10.1007/s00040-007-0936-7>

Diez, L., Urbain, L., Lejeune, P., & Detrain, C. (2015). Emergency measures: Adaptive response to pathogen intrusion in the ant nest. *BEHAVIOURAL PROCESSES*, *116*, 80–86. <https://doi.org/10.1016/j.beproc.2015.04.016>

Dijkstra, M. B., & Boomsma, J. J. (2003). Gnamptogenys hartmani Wheeler (Ponerinae: Ectatommini): An agro-predator of Trachymyrmex and Sericomyrmex fungus-growing ants. *Naturwissenschaften*, *90*(12), 568–571. Scopus. <https://doi.org/10.1007/s00114-003-0478-4>

Dijkstra, M. B., & Boomsma, J. J. (2008). Sex allocation in fungus-growing ants: Worker or queen control without symbiont-induced female bias. *Oikos*, *117*(12), 1892–1906. Scopus. <https://doi.org/10.1111/j.1600-0706.2008.16822.x>

Diniz, E. A., & Bueno, O. C. (2009). Substrate preparation behaviors for the cultivation of the symbiotic fungus in leaf-cutting ants of the genus atta (Hymenoptera: Formicidae). *Sociobiology*, *53*(3), 651–666. Scopus.

Diniz, E. A., & Bueno, O. C. (2010). Evolution of substrate preparation behaviors for cultivation of symbiotic fungus in Attine ants (Hymenoptera: Formicidae). *Journal of Insect Behavior*, *23*(3), 205–214. Scopus. <https://doi.org/10.1007/s10905-010-9207-y>

Diniz, E. A., Bueno, O. C., & Carlos, A. A. (2010). Behavioral repertoire of basal fungus-growing ant sexuals (Hymenoptera: Formicidae) in the parental nest. *Sociobiology*, *55*(2), 387–394. Scopus.

Diniz, J. L. M., Roberto, C., Brandão, F., & Yamamoto, C. I. (1998). Biology of Blepharidatta ants, the sister group of the attini: A possible origin of fungus-ant symbiosis. *Naturwissenschaften*, *85*(6), 270–274. Scopus. <https://doi.org/10.1007/s001140050497>

Dionisi, M., Ozier-Lafontaine, H., & Laplace, D. (2021). Control of Amazonian Leaf-Cutting Ants (Hymenoptera: Formicidae): A Multi-criteria Analysis. *JOURNAL OF ECONOMIC ENTOMOLOGY*, *114*(2), 493–504. <https://doi.org/10.1093/jee/toaa331>

do Nascimento, M. O., Sarmento, R. D., dos Santos, G. R., de Oliveira, C. A., & de Souza, D. J. (2017). Antagonism of Trichoderma isolates against Leucoagaricus gongylophorus (Singer) Moller. *JOURNAL OF BASIC MICROBIOLOGY*, *57*(8), 699–704. <https://doi.org/10.1002/jobm.201600755>

do Nascimento, M. O., Teles Tenório, A. C., Sarmento, R. A., Melo, R. D. C. C., Della Lucia, T. M. C., Dias Amaral, K., & de Souza, D. J. (2022). Soil actinobacteria inhibit antagonistic fungi of leafcutter ant colonies. *Journal of Basic Microbiology*, *62*(1), 63–73. Scopus. <https://doi.org/10.1002/jobm.202100476>

Doherty, K. R., Zweifel, E. W., Elde, N. C., McKone, M. J., & Zweifel, S. G. (2003). Random amplified polymorphic DNA markers reveal genetic variation in the symbiotic fungus of leaf-cutting ants. *Mycologia*, *95*(1), 19–23. Scopus. <https://doi.org/10.1080/15572536.2004.11833127>

Dong, A. Z., Cokcetin, N., Carter, D. A., & Fernandes, K. E. (2023). Unique antimicrobial activity in honey from the Australian honeypot ant (Camponotus inflatus). *PEERJ*, *11*. <https://doi.org/10.7717/peerj.15645>

Dornelas, A. S. P., Sarmento, R. D., Pedro-Neto, M., da Silva, D. G., dos Santos, G. R., do Nascimento, M. O., Oliveira, C. A., & de Souza, D. J. (2017). Susceptibility of Atta sexdens worker ants treated with the immunosuppressant Sandimmun Neoral to Metarhizium anisopliae. *PESQUISA AGROPECUARIA BRASILEIRA*, *52*(2), 133–136. <https://doi.org/10.1590/S0100-204X2017000200008>

Duarte, A. P. M., Attili-Angelis, D., Baron, N. C., Forti, L. C., & Pagnocca, F. C. (2014). Leaf-cutting ants: An unexpected microenvironment holding human opportunistic black fungi. *Antonie van Leeuwenhoek, International Journal of General and Molecular Microbiology*, *106*(3), 465–473. Scopus. <https://doi.org/10.1007/s10482-014-0215-3>

Duff, L. B., Urichuk, T. M., Hodgins, L. N., Young, J. R., & Untereiner, W. A. (2016). Diversity of fungi from the mound nests of Formica ulkei and adjacent non-nest soils. *Canadian Journal of Microbiology*, *62*(7), 562–571. Scopus. <https://doi.org/10.1139/cjm-2015-0628>

ElHamalawi, Z. A., & Menge, J. A. (1996). The role of snails and ants in transmitting the avocado stem canker pathogen, Phytophthora citricola. *JOURNAL OF THE AMERICAN SOCIETY FOR HORTICULTURAL SCIENCE*, *121*(5), 973–977. <https://doi.org/10.21273/JASHS.121.5.973>

Erthal Jr, M., Silva, C. P., Cooper, R. M., & Samuels, R. I. (2009). Hydrolytic enzymes of leaf-cutting ant fungi. *Comparative Biochemistry and Physiology - B Biochemistry and Molecular Biology*, *152*(1), 54–59. Scopus. <https://doi.org/10.1016/j.cbpb.2008.09.086>

Eschenbrenner, V. (1994). The influence of fungus-cultivating ants (Hymenoptera, Formicidae, Attini) on the morphology of Andosols in Martinique. *Soil Micromorphology*, 405–410. Scopus.

Espadaler, X., Lebas, C., Wagenknecht, J., & Tragust, S. (2011). Laboulbenia formicarum (Ascomycota, Laboulbeniales), an exotic parasitic fungus, on an exotic ant in France. *Vie et Milieu*, *61*(1), 41–44. Scopus.

Espadaler, X., & Santamaria, S. (2012). Ecto- and endoparasitic fungi on ants from the Holarctic Region. *Psyche (London)*. Scopus. <https://doi.org/10.1155/2012/168478>

Estrada, C., Degner, E. C., Rojas, E. I., Wcislo, W. T., & Van Bael, S. A. (2015). The role of endophyte diversity in protecting plants from defoliation by leaf-cutting ants. *Current Science*, *109*(1), 55–61. Scopus.

Estrada, C., Rojas, E. I., Wcislo, W. T., & Van Bael, S. A. (2014). Fungal endophyte effects on leaf chemistry alter the invitro growth rates of leaf-cutting ants’ fungal mutualist, Leucocoprinus gongylophorus. *Fungal Ecology*, *8*(1), 37–45. Scopus. <https://doi.org/10.1016/j.funeco.2013.12.009>

Estrada, C., Wcislo, W. T., & Van Bael, S. A. (2013). Symbiotic fungi alter plant chemistry that discourages leaf-cutting ants. *New Phytologist*, *198*(1), 241–251. Scopus. <https://doi.org/10.1111/nph.12140>

Evans, H. C., Araújo, J. P. M., Halfeld, V. R., & Hughes, D. P. (2018). Epitypification and re-description of the zombie-ant fungus, Ophiocordyceps unilateralis (Ophiocordycipitaceae). *Fungal Systematics and Evolution*, *1*, 13–22. Scopus. <https://doi.org/10.3114/fuse.2018.01.02>

Evans, H. C., Elliot, S. L., & Hughes, D. P. (2011). Hidden diversity behind the zombie-ant fungus ophiocordyceps unilateralis: Four new species described from carpenter ants in Minas Gerais, Brazil. *PLoS ONE*, *6*(3). Scopus. <https://doi.org/10.1371/journal.pone.0017024>

Evans, H. C., & Samson, R. A. (1982). CORDYCEPS SPECIES AND THEIR ANAMORPHS PATHOGENIC ON ANTS (FORMICIDAE) IN TROPICAL FOREST ECOSYSTEMS .1. THE CEPHALOTES (MYRMICINAE) COMPLEX. *TRANSACTIONS OF THE BRITISH MYCOLOGICAL SOCIETY*, *79*(DEC), 431–453. <https://doi.org/10.1016/S0007-1536(82)80037-5>

Evans, H. C., & Samson, R. A. (1984). CORDYCEPS SPECIES AND THEIR ANAMORPHS PATHOGENIC ON ANTS (FORMICIDAE) IN TROPICAL FOREST ECOSYSTEMS .2. THE CAMPONOTUS (FORMICINAE) COMPLEX. *TRANSACTIONS OF THE BRITISH MYCOLOGICAL SOCIETY*, *82*(JAN), 127–150. <https://doi.org/10.1016/S0007-1536(84)80219-3>

Faly, L. I., Brygadyrenko, V. V., Orzekauskaite, A., & Paulauskas, A. (2023). Sensitivity of non-target groups of invertebrates to cypermethrin. *BIOSYSTEMS DIVERSITY*, *31*(3), 393–400. <https://doi.org/10.15421/012347>

Fan, Y. H., Pereira, R. M., Kilic, E., Casella, G., & Keyhani, N. O. (2012). Pyrokinin β-Neuropeptide Affects Necrophoretic Behavior in Fire Ants (S. invicta), and Expression of β-NP in a Mycoinsecticide Increases Its Virulence. *PLOS ONE*, *7*(1). <https://doi.org/10.1371/journal.pone.0026924>

Fang, C. C., Chang, F. H., Duong, P., Kurian, J., & Mueller, U. G. (2020). Colony fitness and garden growth in the asexual fungus-growing ant Mycocepurus smithii (Attini, Formicidae). *Insectes Sociaux*, *67*(1), 35–49. Scopus. <https://doi.org/10.1007/s00040-019-00741-9>

Fekete, A., Emri, T., Gyetvai, A., Gazdag, Z., Pesti, M., Varga, Z., Balla, J., Cserháti, C., Emody, L., Gergely, L., & Pócsi, I. (2007). Development of oxidative stress tolerance resulted in reduced ability to undergo morphologic transitions and decreased pathogenicity in a t-butylhydroperoxide-tolerant mutant of Candida albicans. *FEMS YEAST RESEARCH*, *7*(6), 834–847. <https://doi.org/10.1111/j.1567-1364.2007.00244.x>

Fernandes, T. V., Fernandes, O. L., Gomes, I. J. M. T., Solar, R. R. C., & Campos, R. I. (2024). Ant identity determines the fungi richness and composition of a myrmecochorous seed. *PLoS ONE*, *19*(3 March). Scopus. <https://doi.org/10.1371/journal.pone.0293377>

Fernàndez-Marín, H., Bruner, G., Gomez, E. B., Nash, D. R., Boomsma, J. J., & Wcislo, W. T. (2013). Dynamic disease management in Trachymyrmex fungus-growing ants (Attini: Formicidae). *American Naturalist*, *181*(4), 571–582. Scopus. <https://doi.org/10.1086/669664>

Fernández-Marín, H., Nash, D. R., Higginbotham, S., Estrada, C., van Zweden, J. S., d’Ettorre, P., Wcislo, W. T., & Boomsma, J. J. (2015). Functional role of phenylacetic acid from metapleural gland secretions in controlling fungal pathogens in evolutionarily derived leaf-cutting ants. *PROCEEDINGS OF THE ROYAL SOCIETY B-BIOLOGICAL SCIENCES*, *282*(1807). <https://doi.org/10.1098/rspb.2015.0212>

Fernández-Marín, H., Zimmerman, J. K., & Wcislo, W. T. (2004). Ecological traits and evolutionary sequence of nest establishment in fungus-growing ants (Hymenoptera, Formicidae, Attini). *Biological Journal of the Linnean Society*, *81*(1), 39–48. Scopus. <https://doi.org/10.1111/j.1095-8312.2004.00268.x>

Fernández-Marín, H., Zimmerman, J. K., & Wcislo, W. T. (2007). Fungus garden platforms improve hygiene during nest establishment in Acromyrmex ants (Hymenoptera, Formicidae, Attini). *Insectes Sociaux*, *54*(1), 64–69. Scopus. <https://doi.org/10.1007/s00040-007-0907-z>

Fernández-Marín, H., Zimmerman, J. K., Wcislo, W. T., & Rehner, S. A. (2005). Colony foundation, nest architecture and demography of a basal fungus-growing ant, Mycocepurus smithii (Hymenoptera, Formicidae). *Journal of Natural History*, *39*(20), 1735–1743. Scopus. <https://doi.org/10.1080/00222930400027462>

Filho, T. M. M. M., Stefanelli, L. E. P., Camargo, R. D. S., de Matos, C. A. O., & Forti, L. C. (2021). Biological control in leaf‐cutting ants, atta sexdens (Hymenoptera: Formicidae), using pathogenic fungi. *Revista Arvore*, *45*. Scopus. <https://doi.org/10.1590/1806-908820210000016>

Fisher, P. J., Stradling, D. J., & Pegler, D. N. (1994). Leaf cutting ants, their fungus gardens and the formation of basidiomata of Leucoagaricus gongylophorus. *Topics in Catalysis*, *8*(3), 128–131. Scopus. <https://doi.org/10.1016/S0269-915X(09)80159-6>

Fisher, P. J., Stradling, D. J., Sutton, B. C., & Petrini, L. E. (1996). Microfungi in the fungus gardens of the leaf-cutting ant Atta cephalotes: A preliminary study. *Mycological Research*, *100*(5), 541–546. Scopus. <https://doi.org/10.1016/S0953-7562(96)80006-2>

Folgarait, P. J., & Goffré, D. (2021). Biological control of leaf-cutter ants using pathogenic fungi: Experimental laboratory and field studies. *Entomologia Experimentalis et Applicata*, *169*(9), 813–824. Scopus. <https://doi.org/10.1111/eea.13078>

Folgarait, P. J., & Goffré, D. (2023). Control of pest ants by pathogenic fungi: State of the art. *Frontiers in Fungal Biology*, *4*. Scopus. <https://doi.org/10.3389/ffunb.2023.1199110>

Forslund, K., Pettersson, J., Bryngelsson, T., & Jonsson, L. (2000). Aphid infestation induces PR-proteins differently in barley susceptible or resistant to the birdcherry-oat aphid (Rhopalosiphum padi). *Physiologia Plantarum*, *110*(4), 496–502. Scopus. <https://doi.org/10.1034/j.1399-3054.2000.1100411.x>

Fountain, T., & Hughes, W. O. H. (2011). Weaving resistance: Silk and disease resistance in the weaver ant Polyrhachis dives. *INSECTES SOCIAUX*, *58*(4), 453–458. <https://doi.org/10.1007/s00040-011-0162-1>

Fowler, H. G., Bueno, O. C., Sadatsune, T., & Montelli, A. C. (1993). ANTS AS POTENTIAL VECTORS OF PATHOGENS IN HOSPITALS IN THE STATE OF SAO-PAULO, BRAZIL. *INSECT SCIENCE AND ITS APPLICATION*, *14*(3), 367–370. <https://doi.org/10.1017/S1742758400014879>

Fraser, D. (2019). Discussion of development processes in insect-fungus association derived from the shaggy parasol fruiting on the nests of hairy wood ants. *Ecology and Evolution*, *9*(20), 11619–11630. Scopus. <https://doi.org/10.1002/ece3.5611>

Freinkman, E., Oh, D. C., Scott, J. J., Currie, C. R., & Clardy, J. (2009). Bionectriol A, a polyketide glycoside from the fungus Bionectria sp. Associated with the fungus-growing ant, Apterostigma dentigerum. *Tetrahedron Letters*, *50*(49), 6834–6837. Scopus. <https://doi.org/10.1016/j.tetlet.2009.09.120>

Friese, C. F., & Allen, M. F. (1993). The interaction of harvester ants and vesicular-arbuscular mycorrhizal fungi in a patchy semi-arid environment: The effects of mound structure on fungal dispersion and establishment. *Functional Ecology*, *7*(1), 13–20. Scopus. <https://doi.org/10.2307/2389862>

Frost, C. L., FernÁndez-MarÍn, H., Smith, J. E., & Hughes, W. O. H. (2010). Multiple gains and losses of Wolbachia symbionts across a tribe of fungus-growing ants. *Molecular Ecology*, *19*(18), 4077–4085. Scopus. <https://doi.org/10.1111/j.1365-294X.2010.04764.x>

Fuchs, S., Sundström, L., Bos, N., Stucki, D., & Freitak, D. (2018). Induced immune responses in Formica fusca (Hymenoptera: Formicidae). *MYRMECOLOGICAL NEWS*, *28*, 53–66. <https://doi.org/10.25849/myrmecol.news_028:053>

Fukutani, Y., Hori, A., Tsukada, S., Sato, R., Ishii, J., Kondo, A., Matsunami, H., & Yohda, M. (2015). Improving the odorant sensitivity of olfactory receptor-expressing yeast with accessory proteins. *ANALYTICAL BIOCHEMISTRY*, *471*, 1–8. <https://doi.org/10.1016/j.ab.2014.10.012>

Fuxa, J. R., & Richter, A. R. (2004). Effects of soil moisture and composition and fungal isolate on prevalence of Beauveria bassiana in laboratory colonies of the red imported fire ant (Hymenoptera: Formicidae). *ENVIRONMENTAL ENTOMOLOGY*, *33*(4), 975–981. <https://doi.org/10.1603/0046-225X-33.4.975>

Galvanho, J. P., Carrera, M. P., Moreira, D. D. O., Erthal, M., Silva, C. P., & Samuels, R. I. (2013). Imidacloprid Inhibits Behavioral Defences of the Leaf-Cutting Ant Acromyrmex subterraneus subterraneus (Hymenoptera: Formicidae). *JOURNAL OF INSECT BEHAVIOR*, *26*(1), 1–13. <https://doi.org/10.1007/s10905-012-9328-6>

Gálvez, D., & Chapuisat, M. (2014). Immune priming and pathogen resistance in ant queens. *ECOLOGY AND EVOLUTION*, *4*(10), 1761–1767. <https://doi.org/10.1002/ece3.1070>

Garrido-Jurado, I., Ruano, F., Campos, M., & Quesada-Moraga, E. (2011). Effects of soil treatments with entomopathogenic fungi on soil dwelling non-target arthropods at a commercial olive orchard. *BIOLOGICAL CONTROL*, *59*(2), 239–244. <https://doi.org/10.1016/j.biocontrol.2011.07.001>

Gasch, T., & Vilcinskas, A. (2014). The chemical defense in larvae of the earwig Forficula auricularia. *JOURNAL OF INSECT PHYSIOLOGY*, *67*, 1–8. <https://doi.org/10.1016/j.jinsphys.2014.05.019>

Gegenbauer, C., Bellaire, A., Schintlmeister, A., Schmid, M. C., Kubicek, M., Voglmayr, H., Zotz, G., Richter, A., & Mayer, V. E. (2023). Exo- and endophytic fungi enable rapid transfer of nutrients from ant waste to orchid tissue. *New Phytologist*, *238*(5), 2210–2223. Scopus. <https://doi.org/10.1111/nph.18761>

Gemperline, E., Horn, H. A., Delaney, K., Currie, C. R., & Li, L. (2017). Imaging with Mass Spectrometry of Bacteria on the Exoskeleton of Fungus-Growing Ants. *ACS Chemical Biology*, *12*(8), 1980–1985. Scopus. <https://doi.org/10.1021/acschembio.7b00038>

Gerardo, N. M., & Caldera, E. J. (2007). Labile associations between fungus-growing ant cultivars and their garden pathogens. *ISME Journal*, *1*(5), 373–384. Scopus. <https://doi.org/10.1038/ismej.2007.57>

Gerardo, N. M., Mueller, U. G., & Currie, C. R. (2006). Complex host-pathogen coevolution in the Apterostigma fungus-growing ant-microbe symbiosis. *BMC Evolutionary Biology*, *6*. Scopus. <https://doi.org/10.1186/1471-2148-6-88>

Gerardo, N. M., Mueller, U. G., Price, S. L., & Currie, C. R. (2004). Exploiting a mutualism: Parasite specialization on cultivars within the fungus-growing ant symbiosis. *Proceedings of the Royal Society B: Biological Sciences*, *271*(1550), 1791–1798. Scopus. <https://doi.org/10.1098/rspb.2004.2792>

Gerstner, A. T., Poulsen, M., & Currie, C. R. (2011). Recruitment of minor workers for defense against a specialized parasite of Atta leaf-cutting ant fungus gardens. *Ethology Ecology and Evolution*, *23*(1), 61–75. Scopus. <https://doi.org/10.1080/03949370.2010.529828>

Gianoli, F., Guex-Crosier, Y., Marchetti, O., Wolfensberger, T. J., & Spahn, B. (2005). Anterior segment necrosis in multidrug-resistant Fusarium keratomycosis: A case study. *JOURNAL FRANCAIS D OPHTALMOLOGIE*, *28*(5), 498–501. <https://doi.org/10.1016/S0181-5512(05)81086-1>

Giehr, J., Heinze, J., & Schrempf, A. (2015). The Ant Cardiocondyla elegans as Host of the Enigmatic Endoparasitic Fungus Myrmicinosporidium durum. *Psyche (London)*, *2015*. Scopus. <https://doi.org/10.1155/2015/364967>

Gilbert, J. C., Chinn, J. T., & Tanaka, J. S. (1966). SPIDER MITE TOLERANCE IN MULTIPLE DISEASE RESISTANT TOMATOES. *PROCEEDINGS OF THE AMERICAN SOCIETY FOR HORTICULTURAL SCIENCE*, *89*(DEC), 559-+.

Gippet, J. M. W., Colin, T., Grangier, J., Winkler, F., Haond, M., Dumet, A., Tragust, S., Mondy, N., & Kaufmann, B. (2021). Land-cover and climate factors contribute to the prevalence of the ectoparasitic fungus Laboulbenia formicarum in its invasive ant host Lasius neglectus. *Fungal Ecology*, *51*. Scopus. <https://doi.org/10.1016/j.funeco.2021.101045>

Glesner, J., Kapingidza, A. B., Godzwon, M., Offermann, L. R., Mueller, G. A., DeRose, E. F., Wright, P., Richardson, C. M., Woodfolk, J. A., Vailes, L. D., Wünschmann, S., London, R. E., Chapman, M. D., Ohlin, M., Chruszcz, M., & Pomés, A. (2019). A Human IgE Antibody Binding Site on Der p 2 for the Design of a Recombinant Allergen for Immunotherapy. *JOURNAL OF IMMUNOLOGY*, *203*(9), 2545–2556. <https://doi.org/10.4049/jimmunol.1900580>

Godoy, M. F. P., Victor, S. R., Bellini, A. M., Guerreiro, G., Rocha, W. C., Bueno, O. C., Hebling, M. J. A., Bacci Jr, M., Da Silva, M. F. G. F., Vieira, P. C., Fernandes, J. B., & Pagnocca, F. C. (2005). Inhibition of the symbiotic fungus of leaf-cutting ants by coumarins. *Journal of the Brazilian Chemical Society*, *16*(3 B), 669–672. Scopus. <https://doi.org/10.1590/S0103-50532005000400031>

Goes, A. C., Kooij, P. W., Culot, L., Bueno, O. C., & Rodrigues, A. (2022). Distinct and enhanced hygienic responses of a leaf-cutting ant toward repeated fungi exposures. *Ecology and Evolution*, *12*(7). Scopus. <https://doi.org/10.1002/ece3.9112>

Goffré, D., Cavallo, E. C., Cavalitto, S. F., & Folgarait, P. J. (2018). Selection and yield optimisation of a Beauveria bassiana isolate for the biological control of leaf cutter ants. *BIOCONTROL SCIENCE AND TECHNOLOGY*, *28*(7), 672–687. <https://doi.org/10.1080/09583157.2018.1479730>

Goffré, D., & Folgarait, P. J. (2018). Insights into the biodiversity and causes of distribution of potential entomopathogens associated with leaf-cutting ants. *INSECTES SOCIAUX*, *65*(1), 103–115. <https://doi.org/10.1007/s00040-017-0592-5>

Goffre, D., & Folgarait, P. J. (2019). Phorid species from Acromyrmex’s hosts and effect on their survival of two fungi proposed for the control of leafcutter ants. *Entomologia Experimentalis et Applicata*, *167*(11), 939–949. Scopus. <https://doi.org/10.1111/eea.12842>

Goffre, D., & Folgarait, P. J. (2023). Entomopathogenic Strains of the Fungus Purpureocillium lilacinum Damage the Fungus Cultivar of Pest Leaf-Cutter Ants. *Neotropical Entomology*, *52*(4), 731–741. Scopus. <https://doi.org/10.1007/s13744-023-01052-2>

Goldstein, S. L., & Klassen, J. L. (2020). Pseudonocardia Symbionts of Fungus-Growing Ants and the Evolution of Defensive Secondary Metabolism. *Frontiers in Microbiology*, *11*. Scopus. <https://doi.org/10.3389/fmicb.2020.621041>

Gomes De Siqueira, C., Bacci Jr, M., Pagnocca, F. C., Bueno, O. C., & Hebling, M. J. A. (1998). Metabolism of plant polysaccharides by Leucoagaricus gongylophorus, the symbiotic fungus of the leaf-cutting ant Atta sexdens L. *Applied and Environmental Microbiology*, *64*(12), 4820–4822. Scopus. <https://doi.org/10.1128/aem.64.12.4820-4822.1998>

Gómez, K., Espadaler, X., & Santamaria, S. (2016). Ant-fungus interactons: Laboulbenia camponot batra in Italy and a new host for L. formicarum thaxter (Fungi: Ascomycota, laboulbeniales). *Sociobiology*, *63*(3), 950–955. Scopus. <https://doi.org/10.13102/sociobiology.v63i3.1057>

Gómez, K., Espadaler, X., & Santamaria, S. (2017). First record of an epizoic Laboulbenia (Fungi: Laboulbeniales) on ants (Hymenoptera: Formicidae) in Africa. *Sociobiology*, *64*(2), 155–158. Scopus. <https://doi.org/10.13102/sociobiology.v64i2.1532>

Gómez-Martínez, M., Rodríguez-Herrera, R., González-Domínguez, J. R., Santos-Fernández, M., & Gómez-Martínez, S. (2020). Seed quality of moringa and its adaptability in the field in association with buffelgrass. *ECOSISTEMAS Y RECURSOS AGROPECUARIOS*, *7*(2). <https://doi.org/10.19136/era.a7n2.2408>

Gonçalves, C., Patanita, I., & Espadaler, X. (2012). Substantial, and significant, expansion of ant hosts range for myrmicinosporidium hölldobler, 1933 (Fungi). *Insectes Sociaux*, *59*(3), 395–399. Scopus. <https://doi.org/10.1007/s00040-012-0232-z>

González, C. T., Saltonstall, K., & Fernández-Marín, H. (2019). Garden microbiomes of Apterostigma dentigerum and Apterostigma pilosum fungus-growing ants (Hymenoptera: Formicidae). *Journal of Microbiology*, *57*(10), 842–851. Scopus. <https://doi.org/10.1007/s12275-019-8639-0>

Gopinath, P. M., Narchonai, G., Dhanasekaran, D., Ranjani, A., & Thajuddin, N. (2015). Mycosynthesis, characterization and antibacterial properties of AgNPs against multidrug resistant (MDR) bacterial pathogens of female infertility cases. *ASIAN JOURNAL OF PHARMACEUTICAL SCIENCES*, *10*(2), 138–145. <https://doi.org/10.1016/j.ajps.2014.08.007>

Gossner, M. M., Perret-Gentil, A., Britt, E., Queloz, V., Glauser, G., Ladd, T., Roe, A. D., Cleary, M., Liziniewicz, M., Nielsen, L. R., Ghosh, S. K., Bonello, P., & Eisenring, M. (2023). A glimmer of hope—Ash genotypes with increased resistance to ash dieback pathogen show cross-resistance to emerald ash borer. *NEW PHYTOLOGIST*, *240*(3), 1219–1232. <https://doi.org/10.1111/nph.19068>

Gotting, K., May, D. S., Sosa-Calvo, J., Khadempour, L., Francoeur, C. B., Berasategui, A., Thairu, M. W., Sandstrom, S., Carlson, C. M., Chevrette, M. G., Pupo, M. T., Bugni, T. S., Schultz, T. R., Johnston, J. S., Gerardo, N. M., & Currie, C. R. (2022). Genomic diversification of the specialized parasite of the fungus-growing ant symbiosis. *Proceedings of the National Academy of Sciences of the United States of America*, *119*(51). Scopus. <https://doi.org/10.1073/pnas.2213096119>

Gray, K. W., Cover, S. P., Johnson, R. A., & Rabeling, C. (2018). The dacetine ant Strumigenys arizonica, an apparent obligate commensal of the fungus-growing ant Trachymyrmex arizonensis in southwestern North America. *Insectes Sociaux*, *65*(3), 401–410. Scopus. <https://doi.org/10.1007/s00040-018-0625-8>

Graystock, P., & Hughes, W. O. H. (2011). Disease resistance in a weaver ant, Polyrhachis dives, and the role of antibiotic-producing glands. *BEHAVIORAL ECOLOGY AND SOCIOBIOLOGY*, *65*(12), 2319–2327. <https://doi.org/10.1007/s00265-011-1242-y>

Green, A. M., Mueller, U. G., & Adams, R. M. M. (2002). Extensive exchange of fungal cultivars between sympatric species of fungus-growing ants. *Molecular Ecology*, *11*(2), 191–195. Scopus. <https://doi.org/10.1046/j.1365-294X.2002.01433.x>

Grier, T. J., Hazelhurst, D. M., Duncan, E. A., West, T. K., & Esch, R. E. (2002). Major allergen measurements: Sources of variability, validation, quality assurance, and utility for laboratories, manufacturers, and clinics. *ALLERGY AND ASTHMA PROCEEDINGS*, *23*(2), 125–131.

Grier, T. J., LeFevre, D. M., Duncan, E. A., Esch, R. E., & Coyne, T. C. (2012). Allergen stabilities and compatibilities in mixtures of high-protease fungal and insect extracts. *ANNALS OF ALLERGY ASTHMA & IMMUNOLOGY*, *108*(6), 439–447. <https://doi.org/10.1016/j.anai.2012.04.012>

Guedes, F. L. A., Attili-Angelis, D., & Pagnocca, F. C. (2012). Selective isolation of dematiaceous fungi from the workers of Atta laevigata (Formicidae: Attini). *Folia Microbiologica*, *57*(1), 21–26. Scopus. <https://doi.org/10.1007/s12223-011-0081-6>

Guo, H. G., Miao, S. Z., Ai, P. P., Zhang, M. Z., Yan, Z., & Du, Y. L. (2023). Bioactive volatile compounds from Penicillium digitatum-infected apples: Oviposition attractants for yellow peach moth Conogethes punctiferalis (Lepidoptera: Crambidae). *FRONTIERS IN SUSTAINABLE FOOD SYSTEMS*, *7*. <https://doi.org/10.3389/fsufs.2023.1119717>

Haeder, S., Wirth, R., Herz, H., & Spiteller, D. (2009). Candicidin-producing Streptomyces support leaf-cutting ants to protect their fungus garden against the pathogenic fungus Escovopsis. *Proceedings of the National Academy of Sciences of the United States of America*, *106*(12), 4742–4746. Scopus. <https://doi.org/10.1073/pnas.0812082106>

Haelewaters, D., Boer, P., Gort, G., & Noordijk, J. (2015). Studies of laboulbeniales (Fungi, Ascomycota) on myrmica ants (II): Variation of infection by Rickia wasmannii over habitats and time. *Animal Biology*, *65*(3–4), 219–231. Scopus. <https://doi.org/10.1163/15707563-00002472>

Haelewaters, D., Boer, P., & Noordijk, J. (2015). Studies of Laboulbeniales (Fungi, Ascomycota) on Myrmica ants: Rickia wasmannii in the Netherlands. *Journal of Hymenoptera Research*, *44*, 39–47. Scopus. <https://doi.org/10.3897/JHR.44.4951>

Hagley, E. A. C., & Allen, W. R. (1990). THE GREEN APPLE APHID, APHIS-POMI DEGEER (HOMOPTERA, APHIDIDAE), AS PREY OF POLYPHAGOUS ARTHROPOD PREDATORS IN ONTARIO. *CANADIAN ENTOMOLOGIST*, *122*(11–12), 1221–1228. <https://doi.org/10.4039/Ent1221221-11>

Hamilton, N., Jones, T. H., Shik, J. Z., Wall, B., Schultz, T. R., Blair, H. A., & Adams, R. M. M. (2018). Context is everything: Mapping Cyphomyrmex-derived compounds to the fungus-growing ant phylogeny. *Chemoecology*, *28*(4–5), 137–144. Scopus. <https://doi.org/10.1007/s00049-018-0265-5>

Hanisch, P. E., Sosa-Calvo, J., & Schultz, T. R. (2022). The Last Piece of the Puzzle? Phylogenetic Position and Natural History of the Monotypic Fungus-Farming Ant Genus Paramycetophylax (Formicidae: Attini). *Insect Systematics and Diversity*, *6*(1). Scopus. <https://doi.org/10.1093/isd/ixab029>

Hansen, K. A., Kim, R. R., Lawton, E. S., Tran, J., Lewis, S. K., Deol, A. S., & Van Arnam, E. B. (2022). Bacterial Associates of a Desert Specialist Fungus-Growing Ant Antagonize Competitors with a Nocamycin Analog. *ACS Chemical Biology*, *17*(7), 1824–1830. Scopus. <https://doi.org/10.1021/acschembio.2c00187>

Harinikumar, K. M., & Bagyaraj, D. J. (1994). Potential of earthworms, ants, millipedes, and termites for dissemination of vesicular-arbuscular mycorrhizal fungi in soil. *Biology and Fertility of Soils*, *18*(2), 115–118. Scopus. <https://doi.org/10.1007/BF00336456>

Hassan, A., Kang, L. D., Zhang, K. X., Wang, L., Qin, X. J., Fang, G. B., Lu, Y. Y., & Huang, Q. Y. (2024). Effect of entomopathogenic fungi on behavior and physiology of Solenopsis invicta (Hymenoptera, Formicidae). *JOURNAL OF ECONOMIC ENTOMOLOGY*, *117*(3), 825–833. <https://doi.org/10.1093/jee/toae068>

Hastenreiter, I. N., Sales, T. A., Camargo, R. S., Forti, L. C., & Lopes, J. F. S. (2015). Use of Seeds as Fungus Garden Substrate Changes the Organization of Labor Among Leaf-Cutting Ant Workers. *Neotropical Entomology*, *44*(4), 351–356. Scopus. <https://doi.org/10.1007/s13744-015-0299-0>

Hervey, A., & Nair, M. S. (1979). Antibiotic metabolite of a fungus cultivated by gardening ants. *Mycologia*, *71*(5), 1064–1066. Scopus. <https://doi.org/10.2307/3759295>

Herz, H., Hölldobler, B., & Roces, F. (2008). Delayed rejection in a leaf-cutting ant after foraging on plants unsuitable for the symbiotic fungus. *Behavioral Ecology*, *19*(3), 575–582. Scopus. <https://doi.org/10.1093/beheco/arn016>

Himler, A. G., Caldera, E. J., Baer, B. C., Fernández-Marín, H., & Mueller, U. G. (2009). No sex in fungus-farming ants or their crops. *Proceedings of the Royal Society B: Biological Sciences*, *276*(1667), 2611–2616. Scopus. <https://doi.org/10.1098/rspb.2009.0313>

Hinkle, G., Wetterer, J. K., Schultz, T. R., & Sogin, M. L. (1994). Phytogeny of the attine ant fungi based on analysis of small subunit ribosomal RNA gene sequences. *Science*, *266*(5191), 1695–1697. Scopus. <https://doi.org/10.1126/science.7992052>

Howard, J. J., Cazin Jr, J., & Wiemer, D. F. (1988). Toxicity of terpenoid deterrents to the leafcutting ant Atta cephalotes and its mutualistic fungus. *Journal of Chemical Ecology*, *14*(1), 59–69. Scopus. <https://doi.org/10.1007/BF01022531>

Howe, J., Schiøtt, M., & Boomsma, J. J. (2019). Horizontal partner exchange does not preclude stable mutualism in fungus-growing ants. *Behavioral Ecology*, *30*(2), 372–382. Scopus. <https://doi.org/10.1093/beheco/ary176>

Hu, Q. B., Liu, S. Y., Yin, F., Cai, S. J., Zhong, G. H., & Ren, S. X. (2011). Diversity and virulence of soil-dwelling fungi Isaria spp. And Paecilomyces spp. Against Solenopsis invicta (Hymenoptera: Formicidae). *BIOCONTROL SCIENCE AND TECHNOLOGY*, *21*(2), 225–234. <https://doi.org/10.1080/09583157.2010.515733>

Huang, E. L., Aylward, F. O., Kim, Y. M., Webb-Robertson, B. J. M., Nicora, C. D., Hu, Z., Metz, T. O., Lipton, M. S., Smith, R. D., Currie, C. R., & Burnum-Johnson, K. E. (2014). The fungus gardens of leaf-cutter ants undergo a distinct physiological transition during biomass degradation. *Environmental Microbiology Reports*, *6*(4), 389–395. Scopus. <https://doi.org/10.1111/1758-2229.12163>

Hubbell, S. P., Wiemer, D. F., & Adejare, A. (1983). An antifungal terpenoid defends a neotropical tree (Hymenaea) against attack by fungus-growing ants (Atta). *Oecologia*, *60*(3), 321–327. Scopus. <https://doi.org/10.1007/BF00376846>

Hughes, W. O. H., & Boomsma, J. J. (2004). Let your enemy do the work: Within-host interactions between two fungal parasites of leaf-cutting ants. *PROCEEDINGS OF THE ROYAL SOCIETY B-BIOLOGICAL SCIENCES*, *271*, S104–S106. <https://doi.org/10.1098/rsbl.2003.0115>

Hughes, W. O. H., Eilenberg, J., & Boomsma, J. J. (2002). Trade-offs in group living: Transmission and disease resistance in leaf-cutting ants. *PROCEEDINGS OF THE ROYAL SOCIETY B-BIOLOGICAL SCIENCES*, *269*(1502), 1811–1819. <https://doi.org/10.1098/rspb.2002.2113>

Hughes, W. O. H., Pagliarini, R., Madsen, H. B., Dijkstra, M. B., & Boomsma, J. J. (2008). Antimicrobial defense shows an abrupt evolutionary transition in the fungus-growing ants. *Evolution*, *62*(5), 1252–1257. Scopus. <https://doi.org/10.1111/j.1558-5646.2008.00347.x>

Hughes, W. O. H., Thomsen, L., Eilenberg, J., & Boomsma, J. J. (2004). Diversity of entomopathogenic fungi near leaf-cutting ant nests in a neotropical forest, with particular reference to Metarhizium anisopliae var. Anisopliae. *Journal of Invertebrate Pathology*, *85*(1), 46–53. Scopus. <https://doi.org/10.1016/j.jip.2003.12.005>

Hurd, H. (2015). Ant brains: Manipulated by a fungus. *Microbiology Today*, *42*(1), 18–21. Scopus.

Hywel-Jones, N. L. (1996). Cordyceps myrmecophila-like fungi infecting ants in the leaf litter of tropical forest in Thailand. *Mycological Research*, *100*(5), 613–619. Scopus. <https://doi.org/10.1016/S0953-7562(96)80017-7>

Iqbal, M. F., Feng, W. W., Guan, M., Xiang, L. Z., & Feng, Y. L. (2020). BIOLOGICAL CONTROL OF NATURAL HERBIVORES ON AMBROSIA SPECIES AT LIAONING PROVINCE IN NORTHEAST CHINA. *APPLIED ECOLOGY AND ENVIRONMENTAL RESEARCH*, *18*(1), 1419–1436. <https://doi.org/10.15666/aeer/1801_14191436>

Ishak, H. D., Miller, J. L., Sen, R., Dowd, S. E., Meyer, E., & Mueller, U. G. (2011). Microbiomes of ant castes implicate new microbial roles in the fungus-growing ant Trachymyrmex septentrionalis. *Scientific Reports*, *1*. Scopus. <https://doi.org/10.1038/srep00204>

Ivens, A. B. F., Nash, D. R., Poulsen, M., & Boomsma, J. J. (2009). Caste-specific symbiont policing by workers of Acromyrmex fungus-growing ants. *Behavioral Ecology*, *20*(2), 378–384. Scopus. <https://doi.org/10.1093/beheco/arn150>

Jaccoud, D. B., Hughes, W. O. H., & Jackson, C. W. (1999). The epizootiology of a Metarhizium infection in mini-nests of the leaf-cutting ant Atta sexdens rubropilosa. *ENTOMOLOGIA EXPERIMENTALIS ET APPLICATA*, *93*(1), 51–61. <https://doi.org/10.1046/j.1570-7458.1999.00561.x>

Jackson, J. C., Higgins, L. A., & Lin, X. R. (2009). Conidiation Color Mutants of Aspergillus fumigatus Are Highly Pathogenic to the Heterologous Insect Host Galleria mellonella. *PLOS ONE*, *4*(1). <https://doi.org/10.1371/journal.pone.0004224>

Jaffé, K., & Villegas, G. (1985). On the communication systems of the fungus-growing ant trachymyrmex urichi. *Insectes Sociaux*, *32*(3), 257–274. Scopus. <https://doi.org/10.1007/BF02224915>

Jeong, K. Y., Yi, M. H., Son, M., Lyu, D., Lee, J. H., Yong, T. S., & Park, J. W. (2016). IgE Reactivity of Recombinant Pac c 3 from the Asian Needle Ant (Pachycondyla chinensis). *INTERNATIONAL ARCHIVES OF ALLERGY AND IMMUNOLOGY*, *169*(2), 93–100. <https://doi.org/10.1159/000444364>

Ješovnik, A., Chaul, J., & Schultz, T. (2018). Natural history and nest architecture of the fungus-farming ant genus Sericomyrmex (Hymenoptera: Formicidae). *Myrmecological News*, *26*, 65–80. Scopus.

Ješovnik, A., & Schultz, T. R. (2017). Revision of the fungus-farming ant genus Sericomyrmex Mayr (Hymenoptera, Formicidae, Myrmicinae). *ZooKeys*, *2017*(670), 1–109. Scopus. <https://doi.org/10.3897/zookeys.670.11839>

Jesovnik, A., Sosa-Calvo, J., Lopes, C. T., Vasconcelos, H. L., & Schultz, T. R. (2013). Nest architecture, fungus gardens, queen, males and larvae of the fungus-growing ant Mycetagroicus inflatus Brandão & Mayhé-Nunes. *Insectes Sociaux*, *60*(4), 531–542. Scopus. <https://doi.org/10.1007/s00040-013-0320-8>

Jin, X., Streett, D., Huang, Y., & Ugine, T. (2012). Development of a novel bioassay system to assess the effectiveness of entomopathogenic fungi against imported fire ants. *Biocontrol Science and Technology*, *22*(2), 233–241. Scopus. <https://doi.org/10.1080/09583157.2011.648166>

Jitjak, W., & Sanoamuang, N. (2019). A novel fungus, Mycodomus formicartus associated with black ant, Dolichoderus thoracicus (smith) on bamboo. *Asia-Pacific Journal of Science and Technology*, *24*(3). Scopus. <https://www.scopus.com/inward/record.uri?eid=2-s2.0-85074012739&partnerID=40&md5=89b1527500d3bddfd6bc8df53c9b9994>

Jones, E. R. H., Meakins, G. D., Miners, J. O., Pragnell, J. H., & Wilkins, A. L. (1975). Microbiological hydroxylation. Part XIX. The action of an ant fungus ('Acromyrmex Fungus’) on oxygenated androstanes, pregnanes, and cholestanes. *Journal of the Chemical Society, Perkin Transactions 1*, *16*, 1552–1554. Scopus. <https://doi.org/10.1039/p19750001552>

Jorge, N. D. C., Vasconcelos, H. L., Freitas, M. D. S. C., Vale, F. H. A., & Isaias, R. M. D. S. (2022). The peculiar post-senescence in globoid stem galls triggered by fungi and arboreal ants on Eremanthus erythropappus (DC.) McLeisch (Asteraceae). *South African Journal of Botany*, *150*, 1026–1030. Scopus. <https://doi.org/10.1016/j.sajb.2022.09.015>

Jouvenaz, D. P., & Kimbrough, J. W. (1991). Myrmecomyces annellisae gen. Nov., sp. Nov. (Deuteromycotina: Hyphomycetes), an endoparasitic fungus of fire ants, Solenopsis spp. (Hymenoptera: Formicidae). *Mycological Research*, *95*(12), 1395–1401. Scopus. <https://doi.org/10.1016/S0953-7562(09)80392-4>

Judd, W. W., & Benjamin, R. K. (1958). The Ant Lasius alienus (Foerster) Parasitized by the Fungus Laboulbenia formicarum Thaxter at London, Ontario. *The Canadian Entomologist*, *90*(7), 419. Scopus. <https://doi.org/10.4039/Ent90419-7>

Kafle, L., Wu, W. J., Kao, S. S., & Shih, C. J. (2011). Efficacy of Beauveria bassiana against the red imported fire ant, Solenopsis invicta (Hymenoptera: Formicidae), in Taiwan. *PEST MANAGEMENT SCIENCE*, *67*(11), 1434–1438. <https://doi.org/10.1002/ps.2192>

Kalvelage, E. M., Behrens, F. H., Rauch, C., Voegele, R. T., & Fischer, M. (2022). Arthropods as vectors of esca-related pathogens: Transmission efficiency of ants and earwigs and the potential of earwig feces as inoculum source in vineyards. *VITIS*, *61*(2), 77–85. <https://doi.org/10.5073/vitis.2022.61.77-85>

Kang, Y., Clark, R., Makiyama, M., & Fewell, J. (2011). Mathematical modeling on obligate mutualism: Interactions between leaf-cutter ants and their fungus garden. *Journal of Theoretical Biology*, *289*(1), 116–127. Scopus. <https://doi.org/10.1016/j.jtbi.2011.08.027>

Kasimova, R. G., Tishin, D., Obnosov, Y., Dlussky, G. M., Baksht, F. B., & Kacimov, A. R. (2014). Ant mound as an optimal shape in constructal design: Solar irradiation and circadian brood/fungi-warming sorties. *Journal of Theoretical Biology*, *355*, 21–32. Scopus. <https://doi.org/10.1016/j.jtbi.2014.01.038>

Kaspari, M., Stevenson, B. S., Shik, J., & Kerekes, J. F. (2010). Scaling community structure: How bacteria, fungi, and ant taxocenes differentiate along a tropical forest floor. *Ecology*, *91*(8), 2221–2226. Scopus. <https://doi.org/10.1890/09-2089.1>

Keiser, C. N., Vojvodic, S., Butler, I. O., Sartain, E., Rudolf, V. H. W., & Saltz, J. B. (2018). Queen presence mediates the relationship between collective behaviour and disease susceptibility in ant colonies. *JOURNAL OF ANIMAL ECOLOGY*, *87*(2), 379–387. <https://doi.org/10.1111/1365-2656.12696>

Kelber, C., Rössler, W., Roces, F., & Kleineidam, C. J. (2009). The antennal lobes of fungus-growing ants (Attini): Neuroanatomical traits and evolutionary trends. *Brain, Behavior and Evolution*, *73*(4), 273–284. Scopus. <https://doi.org/10.1159/000230672>

Kelleytunis, K. K., Reid, B. L., & Andis, M. (1995). ACTIVITY OF ENTOMOPATHOGENIC FUNGI IN FREE-FORAGING WORKERS OF CAMPONOTUS-PENNSYLVANICUS (HYMENOPTERA, FORMICIDAE). *JOURNAL OF ECONOMIC ENTOMOLOGY*, *88*(4), 937–943. <https://doi.org/10.1093/jee/88.4.937>

Kellner, K., Fernández-Marín, H., Ishak, H. D., Sen, R., Linksvayer, T. A., & Mueller, U. G. (2013). Co-evolutionary patterns and diversification of ant-fungus associations in the asexual fungus-farming ant Mycocepurus smithii in Panama. *Journal of Evolutionary Biology*, *26*(6), 1353–1362. Scopus. <https://doi.org/10.1111/jeb.12140>

Kellner, K., Ishak, H. D., Linksvayer, T. A., & Mueller, U. G. (2015). Bacterial community composition and diversity in an ancestral ant fungus symbiosis. *FEMS Microbiology Ecology*, *91*(7). Scopus. <https://doi.org/10.1093/femsec/fiv073>

Kereselidze, M., Wegensteiner, R., Goginashvili, N., Tvaradze, M., & Pilarska, D. (2010). Further Studies on the Occurrence of Natural Enemies of Ips typographus (Coleoptera: Curculionidae: Scolytinae) in Georgia. *ACTA ZOOLOGICA BULGARICA*, *62*(2), 131–138.

Kesäniemi, J., Koskimäki, J. J., & Jurvansuu, J. (2019). Corpse management of the invasive Argentine ant inhibits growth of pathogenic fungi. *Scientific Reports*, *9*(1). Scopus. <https://doi.org/10.1038/s41598-019-44144-z>

Khadempour, L., Fan, H., Keefover-Ring, K., Carlos-Shanley, C., Nagamoto, N. S., Dam, M. A., Pupo, M. T., & Currie, C. R. (2020). Metagenomics Reveals Diet-Specific Specialization of Bacterial Communities in Fungus Gardens of Grass- and Dicot-Cutter Ants. *Frontiers in Microbiology*, *11*. Scopus. <https://doi.org/10.3389/fmicb.2020.570770>

Kim, A. S., Majmudar, S. P., & Yu, B. H. (2006). Allergic rhinitis in Korean immigrants to the United States. *ALLERGY AND ASTHMA PROCEEDINGS*, *27*(1), 59–62.

Kim, J. H., Scherer, G., Lumpkin, D. S., Rao, K., Puentes Flores, C. D., & Van Arnam, E. B. (2023). Amycolatopsis from Desert Specialist Fungus-Growing Ants Suppresses Contaminant Fungi Using the Antibiotic ECO-0501. *Applied and Environmental Microbiology*, *89*(2). Scopus. <https://doi.org/10.1128/aem.01838-22>

Klingenberg, C., & Brandão, C. R. F. (2005). The type specimens of fungus growing ants, attini (Hymenoptera, formicidae, myrmicinae) deposited in the museu de zoologia da Universidade de São Paulo, Brazil. *Papeis Avulsos de Zoologia*, *45*(4), 41–50. Scopus. <https://doi.org/10.1590/s0031-10492005000400001>

Klingenberg, C., & BrandãO, C. R. F. (2009). Revision of the fungus-growing ant genera Mycetophylax Emery And Paramycetophylax Kusnezov rev. Stat., and description of Kalathomyrmex n. Gen. (Formicidae: Myrmicinae: Attini). *Zootaxa*, *2052*, 1–31. Scopus. <https://doi.org/10.11646/zootaxa.2052.1.1>

Kobmoo, N., Mongkolsamrit, S., Arnamnart, N., Luangsa-ard, J. J., & Giraud, T. (2019). Population genomics revealed cryptic species within host-specific zombie-ant fungi (Ophiocordyceps unilateralis). *Molecular Phylogenetics and Evolution*, *140*. Scopus. <https://doi.org/10.1016/j.ympev.2019.106580>

Konorov, E. A., & Nikitin, M. A. (2015). Amplification of CYP9 genes as a preadaptation of the black garden ant Lasius niger to urban conditions. *MOLECULAR BIOLOGY*, *49*(3), 403–407. <https://doi.org/10.1134/S0026893315030103>

Konrad, M., Grasse, A. V., Tragust, S., & Cremer, S. (2015). Anti-pathogen protection versus survival costs mediated by an ectosymbiont in an ant host. *PROCEEDINGS OF THE ROYAL SOCIETY B-BIOLOGICAL SCIENCES*, *282*(1799). <https://doi.org/10.1098/rspb.2014.1976>

Konrad, M., Vyleta, M. L., Theis, F. J., Stock, M., Tragust, S., Klatt, M., Drescher, V., Marr, C., Ugelvig, L. V., & Cremer, S. (2012). Social transfer of pathogenic fungus promotes active immunisation in ant colonies. *PLoS Biology*, *10*(4). Scopus. <https://doi.org/10.1371/journal.pbio.1001300>

Kooij, P. W., Pullens, J. W. M., Boomsma, J. J., & Schiøtt, M. (2016). Ant mediated redistribution of a xyloglucanase enzyme in fungus gardens of Acromyrmex echinatior. *BMC Microbiology*, *16*(1). Scopus. <https://doi.org/10.1186/s12866-016-0697-4>

Kopac, S., Beatty, H., Gialopsos, P., Huntemann, M., Clum, A., Spunde, A., Pillay, M., Palaniappan, K., Varghese, N., Mikhailova, N., Stamatis, D., Reddy, T. B. K., Daum, C., Ng, V., Ivanova, N., Kyrpides, N., Woyke, T., & Klassen, J. L. (2018). High-quality draft genome sequences of eight bacteria isolated from fungus gardens grown by trachymyrmex septentrionalis ants. *Microbiology Resource Announcements*, *7*(2). Scopus. <https://doi.org/10.1128/MRA.00871-18>

Kost, C., Lakatos, T., Böttcher, I., Arendholz, W. R., Redenbach, M., & Wirth, R. (2007). Non-specific association between filamentous bacteria and fungus-growing ants. *Naturwissenschaften*, *94*(10), 821–828. Scopus. <https://doi.org/10.1007/s00114-007-0262-y>

Kunat, M., Wagner, G. K., Staniec, B., Jaszek, M., Matuszewska, A., Stefaniuk, D., & Ptaszyńska, A. A. (2020). Aqueous extracts of jet-black ant Lasius fuliginosus nests for controlling nosemosis, a disease of honeybees caused by fungi of the genus Nosema. *European Zoological Journal*, *87*(1), 770–780. Scopus. <https://doi.org/10.1080/24750263.2020.1845405>

Kurmanbayeva, A., Ospanov, M., Tamang, P., Shah, F. M., Ali, A., Ibrahim, Z. M. A., Cantrell, C. L., Dinara, S., Datkhayev, U., Khan, I. A., & Ibrahim, M. A. (2023). Regioselective Claisen-Schmidt Adduct of 2-Undecanone from Houttuynia cordata Thunb as Insecticide/Repellent against Solenopsis invicta and Repositioning Plant Fungicides against Colletotrichum fragariae. *MOLECULES*, *28*(16). <https://doi.org/10.3390/molecules28166100>

Kurze, C., Jenkins, N. E., & Hughes, D. P. (2020). Evaluation of direct and indirect transmission of fungal spores in ants. *JOURNAL OF INVERTEBRATE PATHOLOGY*, *172*. <https://doi.org/10.1016/j.jip.2020.107351>

Kweskin, M. P. (2004). Jigging in the fungus-growing ant Cyphomyrmex costatus: A response to collembolan garden invaders? *Insectes Sociaux*, *51*(2), 158–162. Scopus. <https://doi.org/10.1007/s00040-003-0712-2>

Kyle, K. E., Puckett, S. P., Caraballo-Rodríguez, A. M., Rivera-Chávez, J., Samples, R. M., Earp, C. E., Raja, H. A., Pearce, C. J., Ernst, M., van der Hooft, J. J. J., Adams, M. E., Oberlies, N. H., Dorrestein, P. C., Klassen, J. L., & Balunas, M. J. (2023). Trachymyrmex septentrionalis ants promote fungus garden hygiene using Trichoderma-derived metabolite cues. *Proceedings of the National Academy of Sciences of the United States of America*, *120*(25). Scopus. <https://doi.org/10.1073/pnas.2219373120>

Lacerda, F. G., Della Lucia, T. M. C., Desouza, O., Pereira, O. L., Kasuya, M. C. M., De Souza, L. M., Couceiro, J. C., & De Souza, D. J. (2014). Social interactions between fungus garden and external workers of Atta sexdens (Linnaeus) (Hymenoptera: Formicidae). *ITALIAN JOURNAL OF ZOOLOGY*, *81*(2), 298–303. <https://doi.org/10.1080/11250003.2014.911369>

Laird, R. A., & Addicott, J. F. (2009). Testing for mycorrhizal fungi-plant-ant indirect effects. *Journal of Plant Interactions*, *4*(1), 7–14. Scopus. <https://doi.org/10.1080/17429140802484049>

Lancellotti, I. R., Araujo, F. V., Rocha, L. M., & Santos, M. G. (2024). Chemical analysis and hydroalcoholic extract activity of ferns on the mutualist fungus of leafcutter ants. *Rodriguesia*, *75*. Scopus. <https://doi.org/10.1590/2175-7860202475023>

Lapeva-Gjonova, A., Csősz, S., & Báthori, F. (2022). Fungi Associated with Messor Ants on the Balkan Peninsula: First Biogeographical Data. *Diversity*, *14*(12). Scopus. <https://doi.org/10.3390/d14121132>

LaPolla, J. S., Mueller, U. G., Seid, M., & Cover, S. P. (2002). Predation by the army ant Neivamyrmex rugulosus on the fungus-growing ant Trachymyrmex arizonensis. *Insectes Sociaux*, *49*(3), 251–256. Scopus. <https://doi.org/10.1007/s00040-002-8310-2>

Lash, C. L., Sturiale, S. L., Kisare, S. A., & Kwit, C. (2020). The effects of a myrmecochore-produced chemical on entomopathogenic fungal growth and seed-dispersing ant survival rates and foraging patterns. *INSECTES SOCIAUX*, *67*(4), 495–505. <https://doi.org/10.1007/s00040-020-00786-1>

Lattke, J. E. (1999). A new species of fungus-growing ant and its implications for attine phylogeny (Hymenoptera: Formicidae). *Systematic Entomology*, *24*(1), 1–6. Scopus. <https://doi.org/10.1046/j.1365-3113.1999.00061.x>

Leach, J. G., & Dosdall, L. (1938). Observations on the dissemination of fungi by ants. *PHYTOPATHOLOGY*, *28*(6), 444–446.

Leal, I. R., Silva, P. S. D., & Oliveira, P. S. (2011). Natural history and ecological correlates of fungus-growing ants (Formicidae: Attini) in the neotropical cerrado savanna. *Annals of the Entomological Society of America*, *104*(5), 901–908. Scopus. <https://doi.org/10.1603/AN11067>

Leal, L. J. A., de la Peña, A. H., Fletes, I. O., López, P. A., Villa-Ruano, N., & Romero-Arenas, O. (2024). Virulence Bioassay of Entomopathogenic Fungi against Adults of Atta mexicana under Controlled Conditions. *APPLIED SCIENCES-BASEL*, *14*(7). <https://doi.org/10.3390/app14073039>

Leal-Dutra, C. A., Yuen, L. M., Guedes, B. A. M., Contreras-Serrano, M., Marques, P. E., & Shik, J. Z. (2023). Evidence that the domesticated fungus Leucoagaricus gongylophorus recycles its cytoplasmic contents as nutritional rewards to feed its leafcutter ant farmers. *IMA Fungus*, *14*(1). Scopus. <https://doi.org/10.1186/s43008-023-00126-5>

Leclerc, J. B., & Detrain, C. (2016). Ants detect but do not discriminate diseased workers within their nest. *SCIENCE OF NATURE*, *103*(7–8). <https://doi.org/10.1007/s00114-016-1394-8>

Leclerc, J. B., & Detrain, C. (2017). Loss of attraction for social cues leads to fungal-infected Myrmica rubra ants withdrawing from the nest. *ANIMAL BEHAVIOUR*, *129*, 133–141. <https://doi.org/10.1016/j.anbehav.2017.05.002>

Leclerc, J. B., & Detrain, C. (2018). Impact of colony size on survival and sanitary strategies in fungus-infected ant colonies. *Behavioral Ecology and Sociobiology*, *72*(1). Scopus. <https://doi.org/10.1007/s00265-017-2415-0>

Leclerc, J. B., Silva, J. P., & Detrain, C. (2018). Impact of soil contamination on the growth and shape of ant nests. *ROYAL SOCIETY OPEN SCIENCE*, *5*(7). <https://doi.org/10.1098/rsos.180267>

Leite, A. C., Oliveira, C. G., Godoy, M. P., Bueno, F. C., De Oliveira, M. D. F. S. D. S., Forim, M. R., Fernandes, J. B., Vieira, P. C., Da Silva, M. F. D. G. F., Bueno, O. C., Pagnocca, F. C., Hebling, M. J. A., & Bacci Jr, M. (2005). Toxicity of Cipadessa fruticosa to the leaf-cutting ants Atta sexdens rubropilosa (Hymenoptera: Formicidae) and their symbiotic fungus. *Sociobiology*, *46*(1), 17–26. Scopus.

Lemes, P. G., de Matos, M. F., Araújo, C. A., Serrao, J. E., & Zanuncio, J. C. (2021). An organic bait based on Palicourea marcgravii (Rubiaceae) and Tephrosia candida (Fabaceae) does not control nests of Atta laevigata (Hymenoptera: Formicidae) in eucalyptus plantations. *AGRICULTURAL AND FOREST ENTOMOLOGY*, *23*(4), 512–517. <https://doi.org/10.1111/afe.12455>

Leroy, C., Maes, A. Q., Louisanna, E., Carrias, J. F., Céréghino, R., Corbara, B., & Séjalon-Delmas, N. (2022). Ants mediate community composition of root-associated fungi in an ant-plant mutualism. *Biotropica*, *54*(3), 645–655. Scopus. <https://doi.org/10.1111/btp.13079>

Li, H. J., Conner, R. L., Liu, Z. Y., Li, Y. W., Chen, Y., Zhou, Y. L., Duan, X. Y., Shen, T. M., Chen, Q., Graf, R. J., & Jia, X. (2007). Characterization of wheat-triticale lines resistant to powdery mildew, stem rust, stripe rust, wheat curl mite, and limitation on spread of WSMV. *PLANT DISEASE*, *91*(4), 368–374. <https://doi.org/10.1094/PDIS-91-4-0368>

Li, S. Z., Jin, X. X., & Chen, J. (2012). Effects of piperidine and piperideine alkaloids from the venom of red imported fire ants, Solenopsis invicta Buren, on Pythium ultimum Trow growth in vitro and the application of piperideine alkaloids to control cucumber damping-off in the greenhouse. *PEST MANAGEMENT SCIENCE*, *68*(12), 1546–1552. <https://doi.org/10.1002/ps.3337>

Li, X. W., Yi, S. W., Chen, L. M., Hafeez, M., Zhang, Z. J., Zhang, J. M., Zhou, S. X., Dong, W. Y., Huang, J., & Lu, Y. B. (2024). The application of entomopathogenic nematode modified microbial communities within nesting mounds of the red imported fire ants, Solenopsis invicta. *SCIENCE OF THE TOTAL ENVIRONMENT*, *912*. <https://doi.org/10.1016/j.scitotenv.2023.168748>

Liberti, J., Sapountzis, P., Hansen, L. H., Sørensen, S. J., Adams, R. M. M., & Boomsma, J. J. (2015). Bacterial symbiont sharing in Megalomyrmex social parasites and their fungus-growing ant hosts. *Molecular Ecology*, *24*(12), 3151–3169. Scopus. <https://doi.org/10.1111/mec.13216>

Lin, W. J., Lee, Y. I., Liu, S. L., Lin, C. C., Chung, T. Y., & Chou, J. Y. (2020). Evaluating the tradeoffs of a generalist parasitoid fungus, Ophiocordyceps unilateralis, on different sympatric ant hosts. *Scientific Reports*, *10*(1). Scopus. <https://doi.org/10.1038/s41598-020-63400-1>

Lindström, S., Timonen, S. S., & Sundström, L. (2023). Microbial communities of the ant Formica exsecta and its nest material. *EUROPEAN JOURNAL OF SOIL SCIENCE*, *74*(3). <https://doi.org/10.1111/ejss.13364>

Little, A. E. F., & Currie, C. R. (2008). Black yeast Symbionts compromise the efficiency of antibiotic defenses in fungus-growing ants. *Ecology*, *89*(5), 1216–1222. Scopus. <https://doi.org/10.1890/07-0815.1>

Little, A. E. F., Murakami, T., Mueller, U. G., & Currie, C. R. (2003). The infrabuccal pellet piles of fungus-growing ants. *Naturwissenschaften*, *90*(12), 558–562. Scopus. <https://doi.org/10.1007/s00114-003-0480-x>

Little, A. E. F., Murakami, T., Mueller, U. G., & Currie, C. R. (2006). Defending against parasites: Fungus-growing ants combine specialized behaviours and microbial symbionts to protect their fungus gardens. *Biology Letters*, *2*(1), 12–16. Scopus. <https://doi.org/10.1098/rsbl.2005.0371>

Loos-Frank, B., & Zimmermann, G. (1976). Dicrocoelium dendriticum analogous changes in behavior of ants of the genus Formica after infections with a fungus of the genus Entomophthora. *Zeitschrift für Parasitenkunde*, *49*(3), 281–289. Scopus. <https://doi.org/10.1007/BF00380597>

Lopes, B. C., & Fowler, H. G. (2000). Fungus-growing ants (Hymenoptera: Formicidae) on Santa Catarina Island, Brazil: Patterns of occurrence. *Revista de Biologia Tropical*, *48*(2–3), 643–646. Scopus.

Lopez, E., & Orduz, S. (2003). Metarhizium anisopliae and Trichoderma viride for control of nests of the fungus-growing ant, Atta cephalotes. *Biological Control*, *27*(2), 194–200. Scopus. <https://doi.org/10.1016/S1049-9644(03)00005-7>

Loreto, R. G., & Hughes, D. P. (2016). Disease in the Society: Infectious Cadavers Result in Collapse of Ant Sub-Colonies. *PLOS ONE*, *11*(8). <https://doi.org/10.1371/journal.pone.0160820>

Lu, S. J., Salleh, A. H. M., Mohamad, M. S., Denis, S., Omatu, S., & Yoshioka, M. (2014). Identification of gene knockout strategies using a hybrid of an ant colony optimization algorithm and flux balance analysis to optimize microbial strains. *COMPUTATIONAL BIOLOGY AND CHEMISTRY*, *53*, 175–183. <https://doi.org/10.1016/j.compbiolchem.2014.09.008>

Luo, A. R., Hassler, M. F., Jones, T. H., Vander Meer, R. K., & Adams, R. M. M. (2022). The Evolution of Tyramides in Male Fungus-Growing Ants (Formicidae: Myrmicinae: Attini: Attina). *Journal of Chemical Ecology*, *48*(9–10), 782–790. Scopus. <https://doi.org/10.1007/s10886-022-01382-2>

Lupala, A. S., Oh, S. Y., Park, M. S., Kim, T., Yoo, J. S., Seelan, J. S. S., & Lim, Y. W. (2019). Co-occurrence patterns of wood-decaying fungi and ants in dead pines of South Korea. *Journal of Asia-Pacific Entomology*, *22*(4), 1154–1160. Scopus. <https://doi.org/10.1016/j.aspen.2019.10.009>

Mackintosh, J. A., Trimble, J. E., Jones, M. K., Karuso, P. H., Beattie, A. J., & Veal, D. A. (1995). ANTIMICROBIAL MODE OF ACTION OF SECRETIONS FROM THE METAPLEURAL GLAND OF MYRMECIA-GULOSA (AUSTRALIAN BULL ANT). *CANADIAN JOURNAL OF MICROBIOLOGY*, *41*(2), 136–144. <https://doi.org/10.1139/m95-018>

Mackintosh, J. A., Veal, D. A., Beattie, A. J., & Gooley, A. A. (1998). Isolation from an ant Myrmecia gulosa of two inducible O-glycosylated proline-rich antibacterial peptides. *JOURNAL OF BIOLOGICAL CHEMISTRY*, *273*(11), 6139–6143. <https://doi.org/10.1074/jbc.273.11.6139>

Mains, E. B. (1948). ENTOMOGENOUS FUNGI. *MYCOLOGIA*, *40*(4), 402–416. <https://doi.org/10.2307/3755147>

Maksimova, I. A., Glushakova, A. M., Kachalkin, A. V., Chernov, I. Y., Panteleeva, S. N., & Reznikova, Z. I. (2016). Yeast communities of Formica aquilonia colonies. *MICROBIOLOGY*, *85*(1), 124–129. <https://doi.org/10.1134/S0026261716010045>

Małagocka, J., Eilenberg, J., & Jensen, A. B. (2019). Social immunity behaviour among ants infected by specialist and generalist fungi. *Current Opinion in Insect Science*, *33*, 99–104. Scopus. <https://doi.org/10.1016/j.cois.2019.05.001>

Małagocka, J., Jensen, A. B., & Eilenberg, J. (2017). Pandora formicae, a specialist ant pathogenic fungus: New insights into biology and taxonomy. *Journal of Invertebrate Pathology*, *143*, 108–114. Scopus. <https://doi.org/10.1016/j.jip.2016.12.007>

Mangone, D. M., & Currie, C. R. (2007). Garden substrate preparation behaviours in fungus-growing ants. *Canadian Entomologist*, *139*(6), 841–849. Scopus. <https://doi.org/10.4039/n06-105>

Mankowski, M. E., & Morrell, J. J. (2004). Yeasts associated with the infrabuccal pocket and colonies of the carpenter ant Camponotus vicinus. *MYCOLOGIA*, *96*(2), 226–231. <https://doi.org/10.2307/3762058>

Markó, V., Blommers, L. H. M., Bogya, S., & Helsen, H. (2008). Kaolin particle films suppress many apple pests, disrupt natural enemies and promote woolly apple aphid. *JOURNAL OF APPLIED ENTOMOLOGY*, *132*(1), 26–35. <https://doi.org/10.1111/j.1439-0418.2007.01233.x>

Marti, H. E., Carlson, A. L., Brown, B. V., & Mueller, U. G. (2015). Foundress queen mortality and early colony growth of the leafcutter ant, Atta texana (Formicidae, Hymenoptera). *INSECTES SOCIAUX*, *62*(3), 357–363. <https://doi.org/10.1007/s00040-015-0413-7>

Marti, O. G., & Olson, D. M. (2007). Effect of tillage on cotton aphids (Homoptera: Aphididae), pathogenic fungi, and predators in south central Georgia cotton fields. *Journal of Entomological Science*, *42*(3), 354–367. Scopus. <https://doi.org/10.18474/0749-8004-42.3.354>

Martin, M. M. (1970). The biochemical basis of the fungus-attine ant symbiosis. *Science*, *169*(3940), 16–20. Scopus. <https://doi.org/10.1126/science.169.3940.16>

Martin, M. M., & Martin, J. S. (1970). The biochemical basis for the symbiosis between the ant, Atta colombica tonsipes, and its food fungus. *Journal of Insect Physiology*, *16*(1), 109–119. Scopus. <https://doi.org/10.1016/0022-1910(70)90118-6>

Martin, M. M., & Weber, N. A. (1969). The cellulose-utilizing capability of the fungus cultured by the attine ant Atta colombica tonsipes. *Annals of the Entomological Society of America*, *62*(6), 1386–1387. Scopus. <https://doi.org/10.1093/aesa/62.6.1386>

Martinez, P. A., & Porta, A. O. (2023). A new species of Scatoglyphus Berlese (Acariformes: Astigmata: Scatoglyphidae) associated with nests of the fungus growing ants Acromyrmex lundi (Guérin-Méneville) (Formicidae: Myrmicinae: Attini). *Acarologia*, *63*(1), 201–219. Scopus. <https://doi.org/10.24349/aex4-l3kc>

Masiulionis, V. E., Cabello, M. N., Seifert, K. A., Rodrigues, A., & Pagnocca, F. C. (2015). Escovopsis trichodermoides sp nov., isolated from a nest of the lower attine ant Mycocepurus goeldii. *ANTONIE VAN LEEUWENHOEK INTERNATIONAL JOURNAL OF GENERAL AND MOLECULAR MICROBIOLOGY*, *107*(3), 731–740. <https://doi.org/10.1007/s10482-014-0367-1>

Masiulionis, V. E., & Pagnocca, F. C. (2020). In vitro study of volatile organic compounds produced by the mutualistic fungus of leaf-cutter ants and the antagonist Escovopsis. *Fungal Ecology*, *48*. Scopus. <https://doi.org/10.1016/j.funeco.2020.100986>

Masiulionis, V. E., Rabeling, C., De Fine Licht, H. H., Schultz, T., Bacci Jr, M., Santos Bezerra, C. M., & Pagnocca, F. C. (2014). A Brazilian population of the asexual fungus-growing ant Mycocepurus smithii (formicidae, myrmicinae, attini) cultivates fungal symbionts with gongylidia-like structures. *PLoS ONE*, *9*(8). Scopus. <https://doi.org/10.1371/journal.pone.0103800>

Matos, F. S., da Silva, D. Z., de Souza, B. R., Moura, D. R., Lopes, V. D., Carvalho, D. D. C., & Araujo, M. D. (2015). ANALYSIS OF Rhizoctonia SP GROWTH, INCIDENCE AND ANTIXENOSIS EFFECT FOR LEAF-CUTTING ANT Atta sexdens rubropilosa F. IN CLONES OF Eucalyptus grandis x Eucalyptus urophylla TREATED WITH GIBBERELLIN. *REVISTA ARVORE*, *39*(5), 915–922. <https://doi.org/10.1590/0100-67622015000500014>

Matthews, A. E., Kellner, K., & Seal, J. N. (2021). Male-biased dispersal in a fungus-gardening ant symbiosis. *Ecology and Evolution*, *11*(5), 2307–2320. Scopus. <https://doi.org/10.1002/ece3.7198>

Mattoso, T. C., Moreira, D. D. O., & Samuels, R. I. (2012). Symbiotic bacteria on the cuticle of the leaf-cutting ant Acromyrmex subterraneus subterraneus protect workers from attack by entomopathogenic fungi. *Biology Letters*, *8*(3), 461–464. Scopus. <https://doi.org/10.1098/rsbl.2011.0963>

Máximo, H. J., Felizatti, H. L., Ceccato, M., Cintra-Socolowski, P., & Beretta, A. L. (2014). Ants as vectors of pathogenic microorganisms in a hospital in São Paulo county, Brazil. *BMC Res Notes*, *7*, 554. <https://doi.org/10.1186/1756-0500-7-554>

Mayhé-Nunes, A. J., & Brandão, C. R. F. (2006). Revisionary notes on the fungus-growing ant genus Mycetarotes Emery (Hymenoptera, Formicidae). *Revista Brasileira de Entomologia*, *50*(4), 463–472. Scopus. <https://doi.org/10.1590/S0085-56262006000400005>

Mehdiabadi, N. J., Mueller, U. G., Brady, S. G., Himler, A. G., & Schultz, T. R. (2012). Symbiont fidelity and the origin of species in fungus-growing ants. *Nature Communications*, *3*. Scopus. <https://doi.org/10.1038/ncomms1844>

Mehdiabadi, N. J., & Schultz, T. R. (2009). Natural history and phylogeny of the fungus-farming ants (Hymenoptera: Formicidae: Myrmicinae: Attini). *Myrmecological News*, *13*, 37–55. Scopus.

Meirelles, L. A., Montoya, Q. V., Solomon, S. E., & Rodrigues, A. (2015). New light on the systematics of fungi associated with attine ant gardens and the description of Escovopsis kreiselii sp. Nov. *PLoS ONE*, *10*(1). Scopus. <https://doi.org/10.1371/journal.pone.0112067>

Meirelles, L. A., Solomon, S. E., Bacci, M., Wright, A. M., Mueller, U. G., & Rodrigues, A. (2015). Shared Escovopsis parasites between leaf-cutting and non-leaf-cutting ants in the higher attine fungus-growing ant symbiosis. *Royal Society Open Science*, *2*(9). Scopus. <https://doi.org/10.1098/rsos.150257>

Melo, W. G. P., Arcuri, S. L., Rodrigues, A., Morais, P. B., Meirelles, L. A., & Pagnocca, F. C. (2014). Starmerella aceti f.a., sp. Nov., an ascomycetous yeast species isolated from fungus garden of the leafcutter ant Acromyrmex balzani. *International Journal of Systematic and Evolutionary Microbiology*, *64*(PART 4), 1428–1433. Scopus. <https://doi.org/10.1099/ijs.0.058818-0>

Mendonça, A. D., da Silva, C. E., de Mesquita, F. L. T., Campos, R. D., Do Nascimento, R. R., Ximenes, E., & Sant’Ana, A. E. G. (2009). Antimicrobial activities of components of the glandular secretions of leaf cutting ants of the genus Atta. *ANTONIE VAN LEEUWENHOEK INTERNATIONAL JOURNAL OF GENERAL AND MOLECULAR MICROBIOLOGY*, *95*(4), 295–303. <https://doi.org/10.1007/s10482-009-9312-0>

Mendonça, D. M. F. D., Caixeta, M. C. S., Martins, G. L., Moreira, C. C., Kloss, T. G., & Elliot, S. L. (2021). Low Virulence of the Fungi Escovopsis and Escovopsioides to a Leaf-Cutting Ant-Fungus Symbiosis. *Frontiers in Microbiology*, *12*. Scopus. <https://doi.org/10.3389/fmicb.2021.673445>

Mera-Rodríguez, D., Serna, F., Sosa-Calvo, J., Lattke, J., & Rabeling, C. (2020). A checklist of the non-leaf-cutting fungus-growing ants (Hymenoptera, formicidae) from colombia, with new biogeographic records. *Check List*, *16*(5), 1205–1227. Scopus. <https://doi.org/10.15560/16.5.1205>

Metzler, S., Kirchner, J., Grasse, A. V., & Cremer, S. (2023). Trade-offs between immunity and competitive ability in fighting ant males. *BMC ECOLOGY AND EVOLUTION*, *23*(1). <https://doi.org/10.1186/s12862-023-02137-7>

Michaud, J. P., & Browning, H. W. (1999). Seasonal abundance of the brown citrus aphid Toxoptera citricida, (Homoptera: Aphididae) and its natural enemies in Puerto Rico. *FLORIDA ENTOMOLOGIST*, *82*(3), 424–447. <https://doi.org/10.2307/3496869>

Micocci, K. C., Moreira, A. C., Sanchez, A. D., Pettinatti, J. L., Rocha, M. C., Dionizio, B. S., Correa, K. C. S., Malavazi, I., Wouters, F. C., Bueno, O. C., & Souza, D. H. F. (2023). Identification, cloning, and characterization of a novel chitinase from leaf-cutting ant Atta sexdens: An enzyme with antifungal and insecticidal activity. *BIOCHIMICA ET BIOPHYSICA ACTA-GENERAL SUBJECTS*, *1867*(1). <https://doi.org/10.1016/j.bbagen.2022.130249>

Micolino, R., Cristiano, M. P., & Cardoso, D. C. (2020). Karyotype and putative chromosomal inversion suggested by integration of cytogenetic and molecular data of the fungus-farming ant Mycetomoellerius iheringi emery, 1888. *Comparative Cytogenetics*, *14*(2), 197–210. Scopus. <https://doi.org/10.3897/COMPCYTOGEN.V14I2.49846>

Middelhoven, W. J., Fonseca, A., Carreiro, S. C., Pagnocca, F. C., & Bueno, O. C. (2003). Cryptococcus haglerorum, sp nov., an anamorphic basidiomycetous yeast isolated from nests of the leaf-cutting ant Atta sexdens. *ANTONIE VAN LEEUWENHOEK INTERNATIONAL JOURNAL OF GENERAL AND MOLECULAR MICROBIOLOGY*, *83*(2), 167–174. <https://doi.org/10.1023/A:1023384830802>

Mighell, K., & Van Bael, S. A. (2016). Selective elimination of microfungi in leaf-cutting ant gardens. *FUNGAL ECOLOGY*, *24*, 15–20. <https://doi.org/10.1016/j.funeco.2016.08.009>

Mikheyev, A. S. (2004). Male accessory gland size and the evolutionary transition from single to multiple mating in the fungus-gardening ants. *Journal of Insect Science*, *4*. Scopus. <https://doi.org/10.1093/jis/4.1.37>

Mikheyev, A. S., Mueller, U. G., & Abbot, P. (2006). Cryptic sex and many-to-one coevolution in the fungus-growing ant symbiosis. *Proceedings of the National Academy of Sciences of the United States of America*, *103*(28), 10702–10706. Scopus. <https://doi.org/10.1073/pnas.0601441103>

Mikheyev, A. S., Mueller, U. G., & Boomsma, J. J. (2007). Population genetic signatures of diffuse co-evolution between leaf-cutting ants and their cultivar fungi. *Molecular Ecology*, *16*(1), 209–216. Scopus. <https://doi.org/10.1111/j.1365-294X.2006.03134.x>

Mikheyev, A. S., Vo, T., & Mueller, U. G. (2008). Phylogeography of post-Pleistocene population expansion in a fungus-gardening ant and its microbial mutualists. *Molecular Ecology*, *17*(20), 4480–4488. Scopus. <https://doi.org/10.1111/j.1365-294X.2008.03940.x>

Miyashira, C. H., Tanigushi, D. G., Gugliotta, A. M., & Santos, D. Y. (2012). Influence of caffeine on the survival of leaf-cutting ants Atta sexdens rubropilosa and in vitro growth of their mutualistic fungus. *Pest Management Science*, *68*(6), 935–940. Scopus. <https://doi.org/10.1002/ps.3254>

Monteiro, M. R., Torkomian, V. L. V., Pagnocca, F. C., Vieira, P. C., Fernandes, J. B., Da Silva, M. F. D. G. F., Bueno, O. C., & Hebling, M. J. A. (1998). Activity of Extracts and Fatty Acids of Canavalia ensiformis (Leguminosae) Against the Symbiotic Fungus of the Leaf-Cutting Ants Atta sexdens. *Anais Da Academia Brasileira de Ciencias*, *70*(4 PART 1), 732–736. Scopus.

Montgomery, M. P., Vanderwoude, C., & Lynch, A. J. J. (2015). Palatability of baits containing (S)-methoprene to Wasmannia auropunctata (Hymenoptera: Formicidae). *FLORIDA ENTOMOLOGIST*, *98*(2), 451–455. <https://doi.org/10.1653/024.098.0210>

Montoya, Q. V., Martiarena, M. J. S., Polezel, D. A., Kakazu, S., & Rodrigues, A. (2019). More pieces to a huge puzzle: Two new Escovopsis species from fungus gardens of attine ants. *MycoKeys*, *46*, 97–118. Scopus. <https://doi.org/10.3897/mycokeys.46.30951>

Montoya, Q. V., Martiarena, M. J. S., & Rodrigues, A. (2023). Taxonomy and systematics of the fungus-growing ant associate Escovopsis (Hypocreaceae). *Studies in Mycology*, *106*, 349–397. Scopus. <https://doi.org/10.3114/sim.2022.106.06>

Montoya, Q. V., Meirelles, L. A., Chaverri, P., & Rodrigues, A. (2016). Unraveling Trichoderma species in the attine ant environment: Description of three new taxa. *ANTONIE VAN LEEUWENHOEK INTERNATIONAL JOURNAL OF GENERAL AND MOLECULAR MICROBIOLOGY*, *109*(5), 633–651. <https://doi.org/10.1007/s10482-016-0666-9>

Morais, W. C. C., Lima, M. A. P., Zanuncio, J. C., Oliveira, M. A., Bragança, M. A. L., Serrão, J. E., & Lucia, T. M. C. D. (2015). Extracts of Ageratum conyzoides, Coriandrum sativum and Mentha piperita inhibit the growth of the symbiotic fungus of leaf-cutting ants. *Industrial Crops and Products*, *65*, 463–466. Scopus. <https://doi.org/10.1016/j.indcrop.2014.10.054>

Moreno, L. F., Mayer, V., Voglmayr, H., Blatrix, R., Benjamin Stielow, J., Teixeira, M. M., Vicente, V. A., & de Hoog, S. (2019). Genomic analysis of ant domatia-associated melanized fungi (Chaetothyriales, Ascomycota). *Mycological Progress*, *18*(4), 541–552. Scopus. <https://doi.org/10.1007/s11557-018-01467-x>

Moreno, L. F., Stielow, J. B., Vries, M., Weiss, V. A., Vicente, V. A., & de Hoog, S. (2015). Draft genome sequence of the ant-associated fungus Phialophora attae (CBS 131958). *Genome Announcements*, *3*(6). Scopus. <https://doi.org/10.1128/genomeA.01099-15>

Moura, M. N., Cardoso, D. C., Baldez, B. C., & Cristiano, M. P. (2020). Intraspecific variation in the karyotype length and genome size of fungus-farming ants (genus mycetophylax), with remarks on procedures for the estimation of genome size in the formicidae by flow cytometry. *PLoS ONE*, *15*(8 August). Scopus. <https://doi.org/10.1371/journal.pone.0237157>

Mudd, A., & Bateman, G. L. (1979). Rates of growth of the food fungus of the leaf-cutting ant Atta cephalotes (L.) (Hymenoptera: Formicidae) on different substrates gathered by the ants. *Bulletin of Entomological Research*, *69*(1), 141–148. Scopus. <https://doi.org/10.1017/S0007485300017971>

Mudd, A., Peregrine, D. J., & Cherrett, J. M. (1978). The chemical basis for the use of citrus pulp as a fungus garden substrate by the leaf-cutting ants Atta cephalotes (L.) and Acromyrmex octospinosus (Reich) (Hymenoptera: Formicidae). *Bulletin of Entomological Research*, *68*(4), 673–685. Scopus. <https://doi.org/10.1017/S0007485300009639>

Mueller, U. G., Himler, A. G., & Farrior, C. E. (2023). Life history, nest longevity, sex ratio, and nest architecture of the fungus-growing ant Mycetosoritis hartmanni (Formicidae: Attina). *PLoS ONE*, *18*(7 JULY). Scopus. <https://doi.org/10.1371/journal.pone.0289146>

Mueller, U. G., Ishak, H. D., Bruschi, S. M., Smith, C. C., Herman, J. J., Solomon, S. E., Mikheyev, A. S., Rabeling, C., Scott, J. J., Cooper, M., Rodrigues, A., Ortiz, A., Brandão, C. R. F., Lattke, J. E., Pagnocca, F. C., Rehner, S. A., Schultz, T. R., Vasconcelos, H. L., Adams, R. M. M., … Bacci, M. (2017). Biogeography of mutualistic fungi cultivated by leafcutter ants. *Molecular Ecology*, *26*(24), 6921–6937. Scopus. <https://doi.org/10.1111/mec.14431>

Mueller, U. G., Kardish, M. R., Ishak, H. D., Wright, A. M., Solomon, S. E., Bruschi, S. M., Carlson, A. L., & Bacci, M. (2018). Phylogenetic patterns of ant–fungus associations indicate that farming strategies, not only a superior fungal cultivar, explain the ecological success of leafcutter ants. *Molecular Ecology*, *27*(10), 2414–2434. Scopus. <https://doi.org/10.1111/mec.14588>

Mueller, U. G., Lipari, S. E., & Milgroom, M. G. (1996). Amplified fragment length polymorphism (AFLP) fingerprinting of symbiotic fungi cultured by the fungus-growing ant Cyphomyrmex minutus. *Molecular Ecology*, *5*(1), 119–122. Scopus. <https://doi.org/10.1111/j.1365-294X.1996.tb00297.x>

Mueller, U. G., Mikheyev, A. S., Hong, E., Sen, R., Warren, D. L., Solomon, S. E., Ishak, H. D., Cooper, M., Miller, J. L., Shaffer, K. A., & Juenger, T. E. (2011). Evolution of cold-tolerant fungal symbionts permits winter fungiculture by leafcutter ants at the northern frontier of a tropical ant-fungus symbiosis. *Proceedings of the National Academy of Sciences of the United States of America*, *108*(10), 4053–4056. Scopus. <https://doi.org/10.1073/pnas.1015806108>

Mueller, U. G., Mikheyev, A. S., Solomon, S. E., & Cooper, M. (2011). Frontier mutualism: Coevolutionary patterns at the northern range limit of the leaf-cutter ant-fungus symbiosis. *Proceedings of the Royal Society B: Biological Sciences*, *278*(1721), 3050–3059. Scopus. <https://doi.org/10.1098/rspb.2011.0125>

Mueller, U. G., Ortiz, A., & Bacci Jr, M. (2010). Planting of fungus onto hibernating workers of the fungusgrowing ant Mycetosoritis clorindae (Attini, Formicidae). *Insectes Sociaux*, *57*(2), 209–215. Scopus. <https://doi.org/10.1007/s00040-010-0072-7>

Mueller, U. G., Poulin, J., & Adams, R. M. M. (2004). Symbiont choice in a fungus-growing ant (Attini, Formicidae). *Behavioral Ecology*, *15*(2), 357–364. Scopus. <https://doi.org/10.1093/beheco/arh020>

Mueller, U. G., & Wcislo, W. T. (1998). Nesting biology of the fungus-growing ant Cyphomyrmex longiscapus Weber (Attini, Formicidae). *Insectes Sociaux*, *45*(2), 181–189. Scopus. <https://doi.org/10.1007/s000400050078>

Munkacsi, A. B., Pan, J. J., Villesen, P., Mueller, U. G., Blackwell, M., & McLaughlin, D. J. (2004). Convergent coevolution in the domestication of coral mushrooms by fungus-growing ants. *Proceedings of the Royal Society B: Biological Sciences*, *271*(1550), 1777–1782. Scopus. <https://doi.org/10.1098/rspb.2004.2759>

Murakami, T., Higashi, S., & Windsor, D. (2000). Mating frequency, colony size, polyethism and sex ratio in fungus-growing ants (Attini). *Behavioral Ecology and Sociobiology*, *48*(4), 276–284. Scopus. <https://doi.org/10.1007/s002650000243>

Muratore, I. B., & Traniello, J. F. A. (2020). Fungus-Growing Ants: Models for the Integrative Analysis of Cognition and Brain Evolution. *Frontiers in Behavioral Neuroscience*, *14*. Scopus. <https://doi.org/10.3389/fnbeh.2020.599234>

Mwamburi, L. A., Laing, M. D., & Miller, R. M. (2015). Effect of surfactants and temperature on germination and vegetative growth of Beauveria bassiana. *BRAZILIAN JOURNAL OF MICROBIOLOGY*, *46*(1), 67–74. <https://doi.org/10.1590/S1517-838246120131077>

Nichols-orians, C. (1991). Condensed tannins, attine ants, and the performance of a symbiotic fungus. *Journal of Chemical Ecology*, *17*(6), 1177–1195. Scopus. <https://doi.org/10.1007/BF01402942>

Nizami, D., Mustafa, M., Gulbol, D. G., Burcin, O., & Yusuf, O. (2010). *SYNERGISTIC ACTIVITIES OF TWO PROPOLIS WITH AMPHOTERICIN B AGAINST SOME AZOLE-RESISTANT CANDIDA STRAINS. PART I* (WOS:000291667100035). 211–215.

Norman, V. C., Butterfield, T., Drijfhout, F., Tasman, K., & Hughes, W. O. H. (2017). Alarm Pheromone Composition and Behavioral Activity in Fungus-Growing Ants. *Journal of Chemical Ecology*, *43*(3), 225–235. Scopus. <https://doi.org/10.1007/s10886-017-0821-4>

North, R. D., Jackson, C. W., & Howse, P. E. (1997). Evolutionary aspects of ant-fungus interactions in leaf-cutting ants. *Trends in Ecology and Evolution*, *12*(10), 386–389. Scopus. <https://doi.org/10.1016/S0169-5347(97)87381-8>

North, R. D., Jackson, C. W., & Howse, P. E. (1999). Communication between the fungus garden and workers of the leaf-cutting ant, Atta sexdens rubropilosa, regarding choice of substrate for the fungus. *Physiological Entomology*, *24*(2), 127–133. Scopus. <https://doi.org/10.1046/j.1365-3032.1999.00122.x>

Novgorodova, T. (2021). Preventing Transmission of Lethal Disease: Removal Behaviour of Lasius fuliginosus (Hymenoptera: Formicidae) Towards Fungus Contaminated Aphids. *INSECTS*, *12*(2). <https://doi.org/10.3390/insects12020099>

Novgorodova, T. A. (2020). Quarantining behaviour in ants: Are Myrmica aphid milkers able to detect and get rid of fungus-contaminated aphids? *Entomologia Experimentalis et Applicata*, *168*(11), 869–877. Scopus. <https://doi.org/10.1111/eea.12985>

Novgorodova, T. A., & Kryukov, V. Y. (2017). Quarantining behaviour of ants towards infected aphids as an antifungal mechanism in ant-aphid interactions. *ENTOMOLOGIA EXPERIMENTALIS ET APPLICATA*, *162*(3), 293–301. <https://doi.org/10.1111/eea.12552>

Nygaard, S., Hu, H., Li, C., Schiøtt, M., Chen, Z., Yang, Z., Xie, Q., Ma, C., Deng, Y., Dikow, R. B., Rabeling, C., Nash, D. R., Wcislo, W. T., Brady, S. G., Schultz, T. R., Zhang, G., & Boomsma, J. J. (2016). Reciprocal genomic evolution in the ant-fungus agricultural symbiosis. *Nature Communications*, *7*. Scopus. <https://doi.org/10.1038/ncomms12233>

Obrtel, R., & Holisova, V. (1981). THE DIET OF HEDGEHOGS IN AN URBAN-ENVIRONMENT. *FOLIA ZOOLOGICA*, *30*(3), 193–201.

Oi, D. H., & Pereira, R. M. (1993). ANT BEHAVIOR AND MICROBIAL PATHOGENS (HYMENOPTERA, FORMICIDAE). *FLORIDA ENTOMOLOGIST*, *76*(1), 63–74. <https://doi.org/10.2307/3496014>

Oi, D. H., Pereira, R. M., Stimac, J. L., & Wood, L. A. (1994). FIELD APPLICATIONS OF BEAUVERIA-BASSIANA FOR CONTROL OF THE RED IMPORTED FIRE ANT (HYMENOPTERA, FORMICIDAE). *JOURNAL OF ECONOMIC ENTOMOLOGY*, *87*(3), 623–630. <https://doi.org/10.1093/jee/87.3.623>

Okuno, M., Tsuji, K., Sato, H., & Fujisaki, K. (2012). Plasticity of grooming behavior against entomopathogenic fungus Metarhizium anisopliae in the ant Lasius japonicus. *Journal of Ethology*, *30*(1), 23–27. Scopus. <https://doi.org/10.1007/s10164-011-0285-x>

Oliveira, A. M., Feitosa, R. M., Vasconcelos, H. L., & Maravalhas, J. (2016). New distribution records of the savanna specialist fungus-farming ant Cyatta Sosa-Calvo et al. (Hymenoptera: Formicidae: Myrmicinae). *Biodiversity Data Journal*, *4*(1). Scopus. <https://doi.org/10.3897/BDJ.4.e10673>

Olm, M. R., West, P. T., Brooks, B., Firek, B. A., Baker, R., Morowitz, M. J., & Banfield, J. F. (2019). Genome-resolved metagenomics of eukaryotic populations during early colonization of premature infants and in hospital rooms. *MICROBIOME*, *7*. <https://doi.org/10.1186/s40168-019-0638-1>

Ortiz, A., & Orduz, S. (2001). In vitro evaluation of Trichoderma and Gliocladium antagonism against the symbiotic fungus of the leaf-cutting ant Atta cephalotes. *Mycopathologia*, *150*(2), 53–60. Scopus. <https://doi.org/10.1023/A:1010843413085>

Osti, J. F., & Rodrigues, A. (2018). Escovopsioides as a fungal antagonist of the fungus cultivated by leafcutter ants. *BMC Microbiology*, *18*(1). Scopus. <https://doi.org/10.1186/s12866-018-1265-x>

Pagnocca, F. C., Bacci, M., Fungaro, M. H., Bueno, O. C., Hebling, M. J., Sant’anna, A., & Capelari, M. (2001). RAPD analysis of the sexual state and sterile mycelium of the fungus cultivated by the leaf-cutting ant Acromyrmex hispidus fallax. *Mycological Research*, *105*(2), 173–176. Scopus. <https://doi.org/10.1017/S0953756200003191>

Pagnocca, F. C., Da Silva, O. A., Hebling-Beraldo, M. J., Bueno, O. C., Fernandes, J. B., & Vieira, P. C. (1990). Toxicity of sesame extracts to the symbiotic fungus of leaf-cutting ants. *Bulletin of Entomological Research*, *80*(3), 349–352. Scopus. <https://doi.org/10.1017/S0007485300050550>

Pagnocca, F. C., Legaspe, M. F. C., Rodrigues, A., Ruivo, C. C. C., Nagamoto, N. S., Bacci Jr, M., & Forti, L. C. (2010). Yeasts isolated from a fungus-growing ant nest, including the description of Trichosporon chiarellii sp. Nov., an anamorphic basidiomycetous yeast. *International Journal of Systematic and Evolutionary Microbiology*, *60*(6), 1454–1459. Scopus. <https://doi.org/10.1099/ijs.0.015727-0>

Pagnocca, F. C., Ribeiro, S. B., Torkomian, V. L. V., Hebling, M. J. A., Bueno, O. C., Da Silva, O. A., Fernandes, J. B., Vieira, P. C., Da Silva, M. F. D. G. F., & Ferreira, A. G. (1996). Toxicity of lignans to symbiotic fungus of leaf-cutting ants. *Journal of Chemical Ecology*, *22*(7), 1325–1330. Scopus. <https://doi.org/10.1007/BF02266969>

Pagnocca, F. C., Rodrigues, A., Nagamoto, N. S., & Bacci Jr, M. (2008). Yeasts and filamentous fungi carried by the gynes of leaf-cutting ants. *Antonie van Leeuwenhoek, International Journal of General and Molecular Microbiology*, *94*(4), 517–526. Scopus. <https://doi.org/10.1007/s10482-008-9268-5>

Pagnocca, F. C., Victor, S. R., Bueno, F. C., Crisóstomo, F. R., Castral, T. C., Fernandes, J. B., Corrêa, A. G., Bueno, O. C., Bacci, M., Hebling, M. J. A., Vieira, P. C., & Da Silva, M. F. G. F. (2006). Synthetic amides toxic to the leaf-cutting ant Atta sexdens rubropilosa L. and its symbiotic fungus. *Agricultural and Forest Entomology*, *8*(1), 17–23. Scopus. <https://doi.org/10.1111/j.1461-9555.2006.00278.x>

Pandey, R., Prabhu, A. A., & Dasu, V. V. (2018). Purification of recombinant human interferon gamma from fermentation broth using reverse micellar extraction: A process optimization study. *SEPARATION SCIENCE AND TECHNOLOGY*, *53*(3), 487–495. <https://doi.org/10.1080/01496395.2017.1395463>

Pantoja, L. D. M., Filho, R. E. M., Brito, E. H. S., Aragão, T. B., Brilhante, R. S. N., Cordeiro, R. A., Rocha, M. F. G., Monteiro, A. J., Quinet, Y. P., & Sidrim, J. J. C. (2009). Ants (Hymenoptera: Formicidae) as carriers of fungi in hospital environments: An emphasis on the genera Tapinoma and Pheidole. *Journal of Medical Entomology*, *46*(4), 895–899. Scopus. <https://doi.org/10.1603/033.046.0423>

Park, Y., Vatanparast, M., & Sajjadian, S. M. (2022). Pathogenicity of Beauveria bassiana ANU1 to the red imported fire ant, Solenopsis invicta workers in Korea. *JOURNAL OF ASIA-PACIFIC ENTOMOLOGY*, *25*(2). <https://doi.org/10.1016/j.aspen.2022.101913>

Peñaflor, M. F. G. V., Victor, S. R., Bueno, O. C., Hebling, M. J. A., Pagnocca, F. C., Leite, A. C., Fernandes, J. B., Vieira, P. C., & Da Silva, M. F. G. F. (2006). Toxicity of straight-chain fatty acids to leaf-cutting ants Atta sexdens rubropilosa (Hymenoptera: Formicidae) and the symbiotic fungus Leucoagaricus gongylophorus. *Sociobiology*, *47*(3), 843–858. Scopus.

Pereira, D. I. B., Botton, S. A., de Azevedo, M. I., Monteiro, D. U., Weiblen, C., Machado, V. S., Donatti, M. G., & Loeck, A. E. (2015). Isolation and molecular characterization of symbiotic fungus from Acromyrmex ambiguus and Acromyrmex heyeri ants of Rio Grande do Sul State, Brazil. *Ciencia Rural*, *45*(7), 1256–1261. Scopus. <https://doi.org/10.1590/0103-8478cr20141064>

Pereira, H., & Detrain, C. (2020). Pathogen avoidance and prey discrimination in ants. *ROYAL SOCIETY OPEN SCIENCE*, *7*(2). <https://doi.org/10.1098/rsos.191705>

Pereira, H., Jossart, M., & Detrain, C. (2020). Waste management by ants: The enhancing role of larvae. *ANIMAL BEHAVIOUR*, *168*, 187–198. <https://doi.org/10.1016/j.anbehav.2020.08.017>

Pereira, H., Willeput, R., & Detrain, C. (2021). A fungus infected environment does not alter the behaviour of foraging ants. *Scientific Reports*, *11*(1). Scopus. <https://doi.org/10.1038/s41598-021-02817-8>

Pereira, J. S., Costa, R. R., Nagamoto, N. S., Forti, L. C., Pagnocca, F. C., & Rodrigues, A. (2016). Comparative analysis of fungal communities in colonies of two leaf-cutting ant species with different substratum preferences. *Fungal Ecology*, *21*, 68–75. Scopus. <https://doi.org/10.1016/j.funeco.2016.03.004>

Pereira, R. M., & Stimac, J. L. (1992). TRANSMISSION OF BEAUVERIA-BASSIANA WITHIN NESTS OF SOLENOPSIS-INVICTA (HYMENOPTERA, FORMICIDAE) IN THE LABORATORY. *ENVIRONMENTAL ENTOMOLOGY*, *21*(6), 1427–1432. <https://doi.org/10.1093/ee/21.6.1427>

Pereira Rdos, S., & Ueno, M. (2008). [Ants as carriers of microorganisms in hospital environments]. *Rev Soc Bras Med Trop*, *41*(5), 492–495. <https://doi.org/10.1590/s0037-86822008000500011>

Pereyra, M., Zeballos, S. R., Galetto, L., & Oliveira, P. S. (2022). Influence of secondary dispersal by ants on invasive processes of exotic species with fleshy fruits. *BIOLOGICAL INVASIONS*, *24*(10), 3275–3289. <https://doi.org/10.1007/s10530-022-02845-z>

Pessoa, C. M., Ranzan, C., Trienveder, L. F., & Trierweiler, J. O. (2015). *Development of Ant Colony Optimization (ACO) Algorithms Based on Statistical Analysis and Hypothesis Testing for Variable Selection* (WOS:000375842300152). *48*, 900–905. <https://doi.org/10.1016/j.ifacol.2015.09.084>

Peters, R. L., Xsoriano, V., Lycett, K., Flynn, C., Idrose, N. S., Tang, M. L. K., Wijesuriya, R., Allen, K. J., Ranganathan, S., Lowe, A. J., Perrett, K. P., Lodge, C. J., Koplin, J. J., & Dharmage, S. C. (2023). Infant food allergy phenotypes and association with lung function deficits and asthma at age 6 years: A population- based, prospective cohort study in Australia. *LANCET CHILD & ADOLESCENT HEALTH*, *7*(9), 636–647. <https://doi.org/10.1016/S2352-4642(23)00133-5>

Pinto-Tomás, A. A., Anderson, M. A., Suen, G., Stevenson, D. M., Chu, F. S. T., Wallace Cleland, W., Weimer, P. J., & Currie, C. R. (2009). Symbiotic nitrogen fixation in the fungus gardens of leaf-cutter ants. *Science*, *326*(5956), 1120–1123. Scopus. <https://doi.org/10.1126/science.1173036>

Pires, L. P., & Del-Claro, K. (2014). Variation in the outcomes of an ant-plant system: Fire and leaf fungus infection reduce benefits to plants with extrafloral nectaries. *Journal of Insect Science*, *14*. Scopus. <https://doi.org/10.1673/031.014.84>

Pontieri, L., Vojvodic, S., Graham, R., Pedersen, J. S., & Linksvayer, T. A. (2014). Ant Colonies Prefer Infected over Uninfected Nest Sites. *PLOS ONE*, *9*(11). <https://doi.org/10.1371/journal.pone.0111961>

Poulsen, M. (2010). Biomedical exploitation of the fungus-growing ant symbiosis. *Drug News and Perspectives*, *23*(3), 203–210. Scopus. <https://doi.org/10.1358/dnp.2010.23.3.1489981>

Poulsen, M., & Boomsma, J. J. (2005). Mutualistic fungi control crop diversity in fungus-growing ants. *Science*, *307*(5710), 741–744. Scopus. <https://doi.org/10.1126/science.1106688>

Poulsen, M., Bot, A. N. M., Nielsen, M. G., & Boomsma, J. J. (2002). Experimental evidence for the costs and hygienic significance of the antibiotic metapleural gland secretion in leaf-cutting ants. *BEHAVIORAL ECOLOGY AND SOCIOBIOLOGY*, *52*(2), 151–157. <https://doi.org/10.1007/s00265-002-0489-8>

Poulsen, M., & Currie, C. R. (2010). Symbiont interactions in a tripartite mutualism: Exploring the presence and impact of antagonism between two fungus-growing ant mutualists. *PLoS ONE*, *5*(1). Scopus. <https://doi.org/10.1371/journal.pone.0008748>

Poulsen, M., Erhardt, D. P., Molinaro, D. J., Lin, T. L., & Currie, C. R. (2007). Antagonistic bacterial interactions help shape host-symbiont dynamics within the fungus-growing ant-microbe mutualism. *PLoS ONE*, *2*(9). Scopus. <https://doi.org/10.1371/journal.pone.0000960>

Poulsen, M., Hughes, W. O. H., & Boomsma, J. J. (2006). Differential resistance and the importance of antibiotic production in Acromyrmex echinatior leaf-cutting ant castes towards the entomopathogenic fungus Aspergillus nomius. *Insectes Sociaux*, *53*(3), 349–355. Scopus. <https://doi.org/10.1007/s00040-006-0880-y>

Poulsen, M., Little, A. E. F., & Currie, C. R. (2009). Fungus-growing ant-microbe symbiosis: Using microbes to defend beneficial associations within symbiotic communities. In *Def. Mutual. In Microb. Symbiosis* (pp. 149–164). CRC Press; Scopus. <https://www.scopus.com/inward/record.uri?eid=2-s2.0-69249170502&partnerID=40&md5=eb57747846e5392b352f85fa683df3c4>

Presa-Parra, E., García-Martínez, M. A., Rosas, F. H., & Núñez-Pastrana, R. (2024). Soil Fungal Pathogens from Different Land Uses/Covers and Their Virulence to Leaf-Cutter Ant, Atta cephalotes. *SOUTHWESTERN ENTOMOLOGIST*, *49*(1), 300–314.

Pull, C. D., & Cremer, S. (2017). Co-founding ant queens prevent disease by performing prophylactic undertaking behaviour. *BMC EVOLUTIONARY BIOLOGY*, *17*. <https://doi.org/10.1186/s12862-017-1062-4>

Pull, C. D., Hughes, W. O. H., & Brown, M. J. F. (2013). Tolerating an infection: An indirect benefit of co-founding queen associations in the ant Lasius niger. *NATURWISSENSCHAFTEN*, *100*(12), 1125–1136. <https://doi.org/10.1007/s00114-013-1115-5>

Pull, C. D., Ugelvig, L. V., Wiesenhofer, F., Grasse, A. V., Tragust, S., Schmitt, T., Brown, M. J. F., & Cremer, S. (2018). Destructive disinfection of infected brood prevents systemic disease spread in ant colonies. *ELIFE*, *7*. <https://doi.org/10.7554/eLife.32073>

Purcell, J., Brütsch, T., & Chapuisat, M. (2012). Effects of the social environment on the survival and fungal resistance of ant brood. *BEHAVIORAL ECOLOGY AND SOCIOBIOLOGY*, *66*(3), 467–474. <https://doi.org/10.1007/s00265-011-1293-0>

Purcell, J., & Chapuisat, M. (2014). Foster carers influence brood pathogen resistance in ants. *PROCEEDINGS OF THE ROYAL SOCIETY B-BIOLOGICAL SCIENCES*, *281*(1792). <https://doi.org/10.1098/rspb.2014.1338>

Qaseem, A. S., Singh, I., Pathan, A. A., Layhadi, J. A., Parkin, R., Alexandra, F., Durham, S. R., Kishore, U., & Shamji, M. H. (2017). A Recombinant Fragment of Human Surfactant Protein D Suppresses Basophil Activation and T-Helper Type 2 and B-Cell Responses in Grass Pollen-induced Allergic Inflammation. *AMERICAN JOURNAL OF RESPIRATORY AND CRITICAL CARE MEDICINE*, *196*(12), 1526–1534. <https://doi.org/10.1164/rccm.201701-0225OC>

Qiu, H. L., Fox, E. G. P., Qin, C. S., Yang, H., Tian, L. Y., Wang, D. S., & Xu, J. Z. (2023). First record of Fusarium concentricum (Hypocreales: Hypocreaceae) isolated from the moth Polychrosis cunninhamiacola (Lepidoptera: Tortricidae) as an entomopathogenic fungus. *Journal of Insect Science*, *23*(2). Scopus. <https://doi.org/10.1093/jisesa/iead008>

Qiu, H. L., Fox, E. G. P., Qin, C. S., Zhao, D. Y., Yang, H., & Xu, J. Z. (2019). Microcapsuled entomopathogenic fungus against fire ants, Solenopsis invicta. *Biological Control*, *134*, 141–149. Scopus. <https://doi.org/10.1016/j.biocontrol.2019.03.018>

Qiu, H. L., Lu, L. H., Shi, Q. X., & He, Y. R. (2014). Fungus Exposed Solenopsis invicta Ants Benefit from Grooming. *Journal of Insect Behavior*, *27*(5), 678–691. Scopus. <https://doi.org/10.1007/s10905-014-9459-z>

Qiu, H. L., Lu, L. H., Zalucki, M. P., & He, Y. R. (2016). Metarhizium anisopliae infection alters feeding and trophallactic behavior in the ant Solenopsis invicta. *JOURNAL OF INVERTEBRATE PATHOLOGY*, *138*, 24–29. <https://doi.org/10.1016/j.jip.2016.05.005>

Qiu, H. L., Lu, L. H., Zhang, C. Y., & He, Y. R. (2014). Pathogenicity of individual isolates of entomopathogenic fungi affects feeding preference of red imported fire ants Solenopsis invicta. *Biocontrol Science and Technology*, *24*(11), 1286–1296. Scopus. <https://doi.org/10.1080/09583157.2014.933313>

Quan, Y., da Silva, N. M., de Souza Lima, B. J. F., de Hoog, S., Vicente, V. A., Mayer, V., Kang, Y., & Shi, D. (2022). Black fungi and ants: A genomic comparison of species inhabiting carton nests versus domatia. *IMA Fungus*, *13*(1). Scopus. <https://doi.org/10.1186/s43008-022-00091-5>

Queiroz, R. R. S., Teodoro, T. B. P., Carolino, A. T., Bitencourt, R. O. B., Souza, W. G., Boechat, M. S. B., Sobrinho, R. R., Silva, G. A., & Samuels, R. I. (2024). Production of Escovopsis conidia and the potential use of this parasitic fungus as a biological control agent of leaf-cutting ant fungus gardens. *Archives of Microbiology*, *206*(3). Scopus. <https://doi.org/10.1007/s00203-024-03862-3>

Quinlan, R. J., & Cherrett, J. M. (1977). The role of substrate preparation in the symbiosis between the leaf‐cutting ant Acromyrmex octospinosus (Reich) and its food fungus. *Ecological Entomology*, *2*(2), 161–170. Scopus. <https://doi.org/10.1111/j.1365-2311.1977.tb00877.x>

Quinlan, R. J., & Cherrett, J. M. (1978). Aspects of the symbiosis of the leaf‐cutting ant Acromyrmex octospinosus (Reich) and its food fungus. *Ecological Entomology*, *3*(3), 221–230. Scopus. <https://doi.org/10.1111/j.1365-2311.1978.tb00922.x>

Quinlan, R. J., & Cherrett, J. M. (1979). The role of fungus in the diet of the leaf‐cutting ant Atta cephalotes (L.). *Ecological Entomology*, *4*(2), 151–160. Scopus. <https://doi.org/10.1111/j.1365-2311.1979.tb00570.x>

Quondam, M., Barbato, C., Pickford, A., Helmer-Citterich, M., & Macino, G. (1997). Homology modeling of Neurospora crassa geranylgeranyl pyrophosphate synthase: Structural interpretation of mutant phenotypes. *PROTEIN ENGINEERING*, *10*(9), 1047–1055. <https://doi.org/10.1093/protein/10.9.1047>

Rabeling, C., & Bacci Jr, M. (2010). A new workerless inquiline in the Lower Attini (Hymenoptera: Formicidae), with a discussion of social parasitism in fungus-growing ants. *Systematic Entomology*, *35*(3), 379–392. Scopus. <https://doi.org/10.1111/j.1365-3113.2010.00533.x>

Rabeling, C., Cover, S. P., Johnson, R. A., & Mueller, U. G. (2007). A review of the North American species of the fungus-gardening ant genus Trachymyrmex (Hymenoptera: Formicidae). *Zootaxa*, *1664*, 1–53. Scopus.

Rabeling, C., Gonzales, O., Schultz, T. R., Bacci Jr, M., Garciad, M. V. B., Verhaaghe, M., Ishaka, H. D., & Muellera, U. G. (2011). Cryptic sexual populations account for genetic diversity and ecological success in a widely distributed, asexual fungus-growing ant. *Proceedings of the National Academy of Sciences of the United States of America*, *108*(30), 12366–12371. Scopus. <https://doi.org/10.1073/pnas.1105467108>

Rabeling, C., Lino-Neto, J., Cappellari, S. C., Dos-Santos, I. A., Mueller, U. G., & Bacci Jr, M. (2009). Thelytokous parthenogenesis in the fungus-gardening ant Mycocepurus smithii (Hymenoptera: Formicidae). *PLoS ONE*, *4*(8). Scopus. <https://doi.org/10.1371/journal.pone.0006781>

Rabeling, C., Schultz, T. R., Pierce, N. E., & Bacci, M. (2014). A social parasite evolved reproductive isolation from its fungus-growing ant host in sympatry. *Current Biology*, *24*(17), 2047–2052. Scopus. <https://doi.org/10.1016/j.cub.2014.07.048>

Rabeling, C., Verhaagh, M., & Engels, W. (2007). Comparative study of nest architecture and colony structure of the fungus-growing ants, Mycocepurus goeldii and M. smithii. *Journal of Insect Science*, *7*. Scopus. <https://doi.org/10.1673/031.007.4001>

Rahman, A. U., Abdullah, A., Faisal, S., Mansour, B., & Yahya, G. (2024). Unlocking the therapeutic potential of Nigella sativa extract: Phytochemical analysis and revealing antimicrobial and antioxidant marvels. *BMC COMPLEMENTARY MEDICINE AND THERAPIES*, *24*(1). <https://doi.org/10.1186/s12906-024-04470-w>

Rahman, M. U., Hanif, M., Shah, F., & Wang, X. P. (2019). BOTRYTIS CINEREA STRUCTURAL DEVELOPMENT AND HOST RESPONSES IN RESISTANT AND SUSCEPTIBLE GRAPEVINE CULTIVARS: A PHYSIOLOGICAL AND STRUCTURAL STUDY. *FRESENIUS ENVIRONMENTAL BULLETIN*, *28*(4A), 3162–3171.

Raines, S. M., Rane, H. S., Bernardo, S. M., Binder, J. L., Lee, S. A., & Parra, K. J. (2013). Deletion of Vacuolar Proton-translocating ATPase Voa Isoforms Clarifies the Role of Vacuolar pH as a Determinant of Virulence-associated Traits in Candida albicans. *JOURNAL OF BIOLOGICAL CHEMISTRY*, *288*(9), 6190–6201. <https://doi.org/10.1074/jbc.M112.426197>

Ramos-Lacau, L. S., Silva, P. S. D., Delabie, J. H. C., Lacau, S., & Bueno, O. C. (2015). Nest biology and demography of the fungus-growing ant cyphomyrmex lectus forel (Myrmicinae: Attini) at a disturbed area located in Rio Claro-SP, Brazil. *Sociobiology*, *62*(3), 462–466. Scopus. <https://doi.org/10.13102/sociobiology.v62i3.709>

Ramos-Lacau, L. S., Silva, P. S. D., Lacau, S., Delabie, J. H. C., & Bueno, O. C. (2012). Nesting architecture and population structure of the fungus-growing ant cyphomyrmex transversus (Formicidae: Myrmicinae: Attini) in the brazilian coastal zone of ilhéus, bahia. *Annales de La Societe Entomologique de France*, *48*(3–4), 439–445. Scopus. <https://doi.org/10.1080/00379271.2012.10697789>

Rayner, A. D. M., & Franks, N. R. (1987). Evolutionary and ecological parallels between ants and fungi. *Trends in Ecology and Evolution*, *2*(5), 127–133. Scopus. <https://doi.org/10.1016/0169-5347(87)90053-X>

Reber, A., Castella, G., Christe, P., & Chapuisat, M. (2008). Experimentally increased group diversity improves disease resistance in an ant species. *ECOLOGY LETTERS*, *11*(7), 682–689. <https://doi.org/10.1111/j.1461-0248.2008.01177.x>

Reber, A., & Chapuisat, M. (2012a). Diversity, prevalence and virulence of fungal entomopathogens in colonies of the ant Formica selysi. *INSECTES SOCIAUX*, *59*(2), 231–239. <https://doi.org/10.1007/s00040-011-0209-3>

Reber, A., & Chapuisat, M. (2012b). No Evidence for Immune Priming in Ants Exposed to a Fungal Pathogen. *PLOS ONE*, *7*(4). <https://doi.org/10.1371/journal.pone.0035372>

Reber, A., Purcell, J., Buechel, S. D., Buri, P., & Chapuisat, M. (2011). The expression and impact of antifungal grooming in ants. *JOURNAL OF EVOLUTIONARY BIOLOGY*, *24*(5), 954–964. <https://doi.org/10.1111/j.1420-9101.2011.02230.x>

Reinert, J. A. (1978). NATURAL ENEMY COMPLEX OF SOUTHERN CHINCH BUG IN FLORIDA. *ANNALS OF THE ENTOMOLOGICAL SOCIETY OF AMERICA*, *71*(5), 728–731. <https://doi.org/10.1093/aesa/71.5.728>

Reis, B., Silva, A., Alvarez, M. R., Oliveira, T. B., & Rodrigues, A. (2015). Fungal communities in gardens of the leafcutter ant Atta cephalotes in forest and cabruca agrosystems of southern Bahia State (Brazil). *Fungal Biol*, *119*(12), 1170–1178. <https://doi.org/10.1016/j.funbio.2015.09.001>

Revis, H. C., & Waller, D. A. (2004). Bactericidal and fungicidal activity of ant chemicals on feather parasites: An evaluation of anting behavior as a method of self-medication in songbirds. *AUK*, *121*(4), 1262–1268. [https://doi.org/10.1642/0004-8038(2004)121[1262:BAFAOA]2.0.CO;2](https://doi.org/10.1642/0004-8038(2004)121%5b1262:BAFAOA%5d2.0.CO;2)

Richard, F. J., Hefetz, A., Christides, J. P., & Errard, C. (2004). Food influence on colonial recognition and chemical signature between nestmates in the fungus-growing ant Acromyrmex subterraneus subterraneus. *Chemoecology*, *14*(1), 9–16. Scopus. <https://doi.org/10.1007/s00049-003-0251-3>

Richard, F. J., Poulsen, M., Drijfhout, F., Jones, G., & Boomsma, J. J. (2007). Specificity in chemical profiles of workers, brood and mutualistic fungi in Atta, Acromyrmex, and Sericomyrmex fungus-growing ants. *Journal of Chemical Ecology*, *33*(12), 2281–2292. Scopus. <https://doi.org/10.1007/s10886-007-9385-z>

Ridley, P., Howse, P. E., & Jackson, C. W. (1996). Control of the behaviour of leaf-cutting ants by their “symbiotic” fungus. *Experientia*, *52*(6), 631–635. Scopus. <https://doi.org/10.1007/BF01969745>

Riveros, A. J., Seid, M. A., & Wcislo, W. T. (2012). Evolution of brain size in class-based societies of fungus-growing ants (Attini). *Animal Behaviour*, *83*(4), 1043–1049. Scopus. <https://doi.org/10.1016/j.anbehav.2012.01.032>

Roces, F., & Kleineidam, C. (2000). Humidity preference for fungus culturing by workers of the leaf-cutting ant Atta sexdens rubropilosa. *Insectes Sociaux*, *47*(4), 348–350. Scopus. <https://doi.org/10.1007/PL00001728>

Rocha, S. L., Evans, H. C., Jorge, V. L., Cardoso, L. A. O., Pereira, F. S. T., Rocha, F. B., Barreto, R. W., Hart, A. G., & Elliot, S. L. (2017). Recognition of endophytic Trichoderma species by leaf-cutting ants and their potential in a Trojan-horse management strategy. *ROYAL SOCIETY OPEN SCIENCE*, *4*(4). <https://doi.org/10.1098/rsos.160628>

Rocha, S. L., Jorge, V. L., Della Lucia, T. M. C., Barreto, R. W., Evans, H. C., & Elliot, S. L. (2014). Quality control by leaf-cutting ants: Evidence from communities of endophytic fungi in foraged and rejected vegetation. *Arthropod-Plant Interactions*, *8*(5), 485–493. Scopus. <https://doi.org/10.1007/s11829-014-9329-9>

Rodrigues, A., Bacci, M., Mueller, U. G., Ortiz, A., & Pagnocca, F. C. (2008). Microfungal “Weeds” in the Leafcutter Ant Symbiosis. *MICROBIAL ECOLOGY*, *56*(4), 604–614. <https://doi.org/10.1007/s00248-008-9380-0>

Rodrigues, A., Cable, R. N., Mueller, U. G., Bacci, M., & Pagnocca, F. C. (2009). Antagonistic interactions between garden yeasts and microfungal garden pathogens of leaf-cutting ants. *ANTONIE VAN LEEUWENHOEK INTERNATIONAL JOURNAL OF GENERAL AND MOLECULAR MICROBIOLOGY*, *96*(3), 331–342. <https://doi.org/10.1007/s10482-009-9350-7>

Rodrigues, A., Pagnocca, F. C., Bueno, O. C., Pfenning, L. H., & Bacci Jr, M. (2005). Assessment of microfungi in fungus gardens free of the leaf-cutting ant Atta sexdens rubropilosa (Hymenoptera: Formicidae). *Sociobiology*, *46*(2), 329–334. Scopus.

Rodrigues, A., Passarini, M. R. Z., Ferro, M., Nagamoto, N. S., Forti, L. C., Bacci, M., Sette, L. D., & Pagnocca, F. C. (2014). Fungal communities in the garden chamber soils of leaf-cutting ants. *JOURNAL OF BASIC MICROBIOLOGY*, *54*(11), 1186–1196. <https://doi.org/10.1002/jobm.201200458>

Rodrigues, A., Silva, A., Bacci Jr, M., Forti, L. C., & Pagnocca, F. C. (2010). Filamentous fungi found on foundress queens of leaf-cutting ants (Hymenoptera: Formicidae). *Journal of Applied Entomology*, *134*(4), 342–345. Scopus. <https://doi.org/10.1111/j.1439-0418.2009.01466.x>

Rodriguez Rodriguez, M., Smith, N., Phan, T., Woodbury, J., & Kang, Y. (2018). Interactions between leaf-cutter ants and fungus garden: Effects of division of labor, age polyethism, and egg cannibalism. *Mathematical Modelling of Natural Phenomena*, *13*(3). Scopus. <https://doi.org/10.1051/mmnp/2018039>

Rojas, M. G., Elliott, R. B., & Morales-Ramos, J. A. (2018). Mortality of Solenopsis invicta Workers (Hymenoptera: Formicidae) After Indirect Exposure to Spores of Three Entomopathogenic Fungi. *JOURNAL OF INSECT SCIENCE*, *18*(3). <https://doi.org/10.1093/jisesa/iey050>

Römer, D., Aguilar, G. P., Meyer, A., & Roces, F. (2022). Symbiont demand guides resource supply: Leaf-cutting ants preferentially deliver their harvested fragments to undernourished fungus gardens. *Science of Nature*, *109*(3). Scopus. <https://doi.org/10.1007/s00114-022-01797-7>

Römer, D., Bollazzi, M., & Roces, F. (2017). Carbon dioxide sensing in an obligate insectfungus symbiosis: CO2 preferences of leafcutting ants to rear their mutualistic fungus. *PLoS ONE*, *12*(4). Scopus. <https://doi.org/10.1371/journal.pone.0174597>

Römer, D., & Roces, F. (2014). Nest enlargement in leaf-cutting ants: Relocated brood and fungus trigger the excavation of new chambers. *PLoS ONE*, *9*(5). Scopus. <https://doi.org/10.1371/journal.pone.0097872>

Römer, D., & Roces, F. (2015). Available space, symbiotic fungus and colony brood influence excavation and lead to the adjustment of nest enlargement in leaf-cutting ants. *Insectes Sociaux*, *62*(4), 401–413. Scopus. <https://doi.org/10.1007/s00040-015-0419-1>

Rønhede, S., Boomsma, J. J., & Rosendahl, S. (2004). Fungal enzymes transferred by leaf-cutting ants in their fungus gardens. *Mycological Research*, *108*(1), 101–106. Scopus. <https://doi.org/10.1017/S0953756203008931>

Ronque, M. U. V., Migliorini, G. H., & Oliveira, P. S. (2018). Thievery in rainforest fungus-growing ants: Interspecific assault on culturing material at nest entrance. *Insectes Sociaux*, *65*(3), 507–510. Scopus. <https://doi.org/10.1007/s00040-018-0632-9>

Rosas-Mejia, M., Gaona-Garcia, G., Cesar Chacon Hernandez, J., Mora-Ravelo, S., & Vanoye-Eligio, V. (2020). Four New Occurrences of Fungus-Growing Ants (Hymenoptera: Formicidae) in Agroecosystems of Tamaulipas State in Northeastern Mexico. *Journal of Entomological Science*, *55*(3), 433–435. Scopus. <https://doi.org/10.18474/0749-8004-55.3.433>

Rose, E. A. F., Harris, R. J., & Glare, T. R. (1999). Possible pathogens of social wasps (Hymenoptera: Vespidae) and their potential as biological control agents. *NEW ZEALAND JOURNAL OF ZOOLOGY*, *26*(3), 179–190. <https://doi.org/10.1080/03014223.1999.9518188>

Saepua, S., Kornsakulkarn, J., Auncharoen, P., Rachtawee, P., Kongthong, S., Boonyuen, N., Harding, D. J., Nehira, T., Thongpanchang, T., & Thongpanchang, C. (2021). Secondary metabolites from cultures of the ant pathogenic fungus Ophiocordyceps irangiensis BCC 2728. *Natural Product Research*, *35*(21), 3556–3561. Scopus. <https://doi.org/10.1080/14786419.2020.1713119>

Sakhanokho, H. F., Sampson, B. J., Tabanca, N., Wedge, D. E., Demirci, B., Baser, K. H. C., Bernier, U. R., Tsikolia, M., Agramonte, N. M., Becnel, J. J., Chen, J., Rajasekaran, K., & Spiers, J. M. (2013). Chemical Composition, Antifungal and Insecticidal Activities of Hedychium Essential Oils. *MOLECULES*, *18*(4), 4308–4327. <https://doi.org/10.3390/molecules18044308>

Sakolrak, B., Blatrix, R., Sangwanit, U., Arnamnart, N., Noisripoom, W., Thanakitpipattana, D., Buatois, B., Hossaert-McKey, M., & Kobmoo, N. (2018). Ant-produced chemicals are not responsible for the specificity of their Ophiocordyceps fungal pathogens. *FUNGAL ECOLOGY*, *32*, 80–86. <https://doi.org/10.1016/j.funeco.2017.11.005>

Salazar, L. C., Ortiz-Reyes, A., Rosero, D. M., & Lobo-Echeverri, T. (2020). Dillapiole in Piper holtonii as an Inhibitor of the Symbiotic Fungus Leucoagaricus gongylophorus of Leaf-Cutting Ants. *Journal of Chemical Ecology*, *46*(8), 668–674. Scopus. <https://doi.org/10.1007/s10886-020-01170-w>

Saltamachia, S. J., & Araújo, J. P. M. (2020). Ophiocordyceps desmidiospora, a basal lineage within the “Zombie-Ant Fungi” clade. *Mycologia*, *112*(6), 1171–1183. Scopus. <https://doi.org/10.1080/00275514.2020.1732147>

Sanchez-Pea, S. R. (2010). Some fungus-growing ants (Hymenoptera: Formicidae) from northeastern Mexico. *Florida Entomologist*, *93*(4), 501–504. Scopus. <https://doi.org/10.1653/024.093.0404>

Sánchez-Peña, S. R., Chacón-Cardosa, M. C., Canales-del-Castillo, R., Ward, L., & Resendez-Pérez, D. (2017). A new species of Trachymyrmex (Hymenoptera, Formicidae) fungus-growing ant from the Sierra Madre Oriental of northeastern Mexico. *ZooKeys*, *2017*(706), 73–94. Scopus. <https://doi.org/10.3897/zookeys.706.12539>

Sánchez-Peña, S. R., Sánchez-Ovalle, M. R., Gallegos-Morales, G., & Sánchez-Arizpe, A. (2008). Note: In vitro antagonism of actinomycetes isolated from fungus-growing ants against plant pathogenic fungi. *Phytoparasitica*, *36*(4), 322–325. Scopus. <https://doi.org/10.1007/BF02980811>

Sanchezpena, S. R., & Thorvilson, H. G. (1995). EFFECT OF LONG-TERM CRYOGENIC STORAGE AND CONIDIAL SUSPENDING AGENTS ON THE VIRULENCE OF BEAUVERIA-BASSIANA TOWARD SOLENOPSIS-INVICTA. *JOURNAL OF INVERTEBRATE PATHOLOGY*, *65*(3), 248–252. <https://doi.org/10.1006/jipa.1995.1038>

Sanhudo, C. E. D., Izzo, T. J., & Brandão, C. R. F. (2008). Parabiosis between basal fungus-growing ants (Formicidae, Attini). *Insectes Sociaux*, *55*(3), 296–300. Scopus. <https://doi.org/10.1007/s00040-008-1005-6>

Sanjuán, T., Henao, L. G., & Amat, G. (2001). Spatial distribution of Cordyceps spp. (Ascomycotina: Clavicipitaceae) andits impact on the ants in forests of the Amazonian Colombian foothill. *REVISTA DE BIOLOGIA TROPICAL*, *49*(3–4), 945–955.

Santos, A. V., De Oliveira, B. L., & Samuels, R. I. (2007). Selection of entomopathogenic fungi for use in combination with sub-lethal doses of imidacloprid: Perspectives for the control of the leaf-cutting ant Atta sexdens rubropilosa Forel (Hymenoptera: Formicidae). *Mycopathologia*, *163*(4), 233–240. Scopus. <https://doi.org/10.1007/s11046-007-9009-8>

Santos, A. V., Dillon, R. J., Dillon, V. M., Reynolds, S. E., & Samuels, R. I. (2004). Ocurrence of the antibiotic producing bacterium Burkholderia sp in colonies of the leaf-cutting ant Atta sexdens rubropilosa. *FEMS MICROBIOLOGY LETTERS*, *239*(2), 319–323. <https://doi.org/10.1016/j.femsle.2004.09.005>

Santos, V. S. D., Santos Jr, L. C. D., De Almeida, S. S., De Souza, L. E., & Antonialli Jr, W. F. (2011). Evaluation of methods of baiting ants and record of associated fungi occurring in hospitals in mato grosso do sul, Brazil. *Sociobiology*, *57*(1), 143–152. Scopus.

Sapountzis, P., Nash, D. R., Schiøtt, M., & Boomsma, J. J. (2019). The evolution of abdominal microbiomes in fungus-growing ants. *Molecular Ecology*, *28*(4), 879–899. Scopus. <https://doi.org/10.1111/mec.14931>

Sapountzis, P., Zhukova, M., Shik, J. Z., Schiott, M., & Boomsma, J. J. (2018). Reconstructing the functions of endosymbiotic mollicutes in fungus-growing ants. *eLife*, *7*. Scopus. <https://doi.org/10.7554/eLife.39209>

Satyal, P., Dosoky, N. S., Kincer, B. L., & Setzer, W. N. (2012). Chemical Compositions and Biological Activities of Amomum subulatum Essential Oils from Nepal. *NATURAL PRODUCT COMMUNICATIONS*, *7*(9), 1233–1236.

Satyal, P., Paudel, P., Poudel, A., Dosoky, N. S., Pokharel, K. K., & Setzer, W. N. (2013). Bioactivities and Compositional Analyses of Cinnamomum Essential Oils from Nepal: C. camphora, C. tamala, and C. glaucescens. *NATURAL PRODUCT COMMUNICATIONS*, *8*(12), 1777–1784.

Saverschek, N., Herz, H., Wagner, M., & Roces, F. (2010). Avoiding plants unsuitable for the symbiotic fungus: Learning and long-term memory in leaf-cutting ants. *Animal Behaviour*, *79*(3), 689–698. Scopus. <https://doi.org/10.1016/j.anbehav.2009.12.021>

Saverschek, N., & Roces, F. (2011). Foraging leafcutter ants: Olfactory memory underlies delayed avoidance of plants unsuitable for the symbiotic fungus. *Animal Behaviour*, *82*(3), 453–458. Scopus. <https://doi.org/10.1016/j.anbehav.2011.05.015>

Schaffner, U., Boeve, J. L., Gfeller, H., & Schlunegger, U. P. (1994). SEQUESTRATION OF VERATRUM ALKALOIDS BY SPECIALIST RHADINOCERAEA-NODICORNIS KONOW (HYMENOPTERA, TENTHREDINIDAE) AND ITS ECOETHOLOGICAL IMPLICATIONS. *JOURNAL OF CHEMICAL ECOLOGY*, *20*(12), 3233–3250. <https://doi.org/10.1007/BF02033723>

Schär, S., Larsen, L. L. M., Meyling, N. V., & Nash, D. R. (2015). Reduced entomopathogen abundance in Myrmica ant nests-testing a possible immunological benefit of myrmecophily using Galleria mellonella as a model. *ROYAL SOCIETY OPEN SCIENCE*, *2*(10). <https://doi.org/10.1098/rsos.150474>

Schildknecht, H., Reed, P. B., Dewitt Reed, F., & Koob, K. (1973). Auxin activity in the symbiosis of leaf-cutting ants and their fungus. *Insect Biochemistry*, *3*(12), 439–442. Scopus. <https://doi.org/10.1016/0020-1790(73)90077-2>

Schiøtt, M., De Fine Licht, H. H., Lange, L., & Boomsma, J. J. (2008). Towards a molecular understanding of symbiont function: Identification of a fungal gene for the degradation of xylan in the fungus gardens of leaf-cutting ants. *BMC Microbiology*, *8*. Scopus. <https://doi.org/10.1186/1471-2180-8-40>

Schiøtt, M., Rogowska-Wrzesinska, A., Roepstorff, P., & Boomsma, J. J. (2010). Leaf-cutting ant fungi produce cell wall degrading pectinase complexes reminiscent of phytopathogenic fungi. *BMC Biology*, *8*. Scopus. <https://doi.org/10.1186/1741-7007-8-156>

Schlick-Steiner, B. C., Steiner, F. M., Konrad, H., Seifert, B., Christian, E., Moder, K., Stauffer, C., & Crozier, R. H. (2008). Specificity and transmission mosaic of ant nest-wall fungi. *Proceedings of the National Academy of Sciences of the United States of America*, *105*(3), 940–943. Scopus. <https://doi.org/10.1073/pnas.0708320105>

Schultz, T. R., Bekkevold, D., & Boomsma, J. J. (1998). Acromyrmex insinuator new species: An incipient social parasite of fungus-growing ants. *Insectes Sociaux*, *45*(4), 457–471. Scopus. <https://doi.org/10.1007/s000400050101>

Schultz, T. R., & Meier, R. (1995). A phylogenetic analysis of the fungus‐growing ants (Hymenoptera: Formicidae: Attini) based on morphological characters of the larvae. *Systematic Entomology*, *20*(4), 337–370. Scopus. <https://doi.org/10.1111/j.1365-3113.1995.tb00100.x>

Schultz, T. R., Solomon, S. A., Mueller, U. G., Villesen, P., Boomsma, J. J., Adams, R. M. M., & Norden, B. (2002). Cryptic speciation in the fungus-growing ants Cyphomyrmex longiscapus weber and Cyphomyrmex muelleri Schultz and Solomon, new species (Formicidae, attini). *Insectes Sociaux*, *49*(4), 331–343. Scopus. <https://doi.org/10.1007/PL00012657>

Schultz, T. R., Sosa-Calvo, J., Brady, S. G., Lopes, C. T., Mueller, U. G., Bacci, M., & Vasconcelos, H. L. (2015). The most relictual fungus-farming ant species cultivates the most recently evolved and highly domesticated fungal symbiont species. *American Naturalist*, *185*(5), 693–703. Scopus. <https://doi.org/10.1086/680501>

Schürch, S., Pfunder, M., & Roy, B. A. (2000). Effects of ants on the reproductive success of Euphorbia cyparissias and associated pathogenic rust fungi. *Oikos*, *88*(1), 6–12. Scopus. <https://doi.org/10.1034/j.1600-0706.2000.880102.x>

Sciascia, Q. L., Sullivan, P. A., & Farley, P. C. (2004). Deletion of the Candida albicans G-protein-coupled receptor, encoded by orf19.1944 and its allele orf19.9499, produces mutants defective in filamentous growth. *CANADIAN JOURNAL OF MICROBIOLOGY*, *50*(12), 1081–1085. <https://doi.org/10.1139/W04-095>

Scott, J. J., Budsberg, K. J., Suen, G., Wixon, D. L., Balser, T. C., & Currie, C. R. (2010). Microbial community structure of leaf-cutter ant fungus gardens and refuse dumps. *PLoS ONE*, *5*(3). Scopus. <https://doi.org/10.1371/journal.pone.0009922>

Scott, J. J., Weskin, M. K., Cooper, M., & Mueller, U. G. (2009). Polymorphic microsatellite markers for the symbiotic fungi cultivated by leaf cutter ants (Attini, Formicidae). *Molecular Ecology Resources*, *9*(5), 1391–1394. Scopus. <https://doi.org/10.1111/j.1755-0998.2009.02684.x>

Seal, J. N. (2009). Scaling of body weight and fat content in fungus-gardening ant queens: Does this explain why leaf-cutting ants found claustrally? *Insectes Sociaux*, *56*(2), 135–141. Scopus. <https://doi.org/10.1007/s00040-009-0002-8>

Seal, J. N., Gus, J., & Mueller, U. G. (2012). Fungus-gardening ants prefer native fungal species: Do ants control their crops? *Behavioral Ecology*, *23*(6), 1250–1256. Scopus. <https://doi.org/10.1093/beheco/ars109>

Seal, J. N., Schiøtt, M., & Mueller, U. G. (2014). Ant-fungus species combinations engineer physiological activity of fungus gardens. *Journal of Experimental Biology*, *217*(14), 2540–2547. Scopus. <https://doi.org/10.1242/jeb.098483>

Seal, J. N., & Tschinkel, W. R. (2006). Colony productivity of the fungus-gardening ant Trachymyrmex septentrionalis (Hymenoptera: Formicidae) in a Florida pine forest. *Annals of the Entomological Society of America*, *99*(4), 673–682. Scopus. [https://doi.org/10.1603/0013-8746(2006)99[673:CPOTFA]2.0.CO;2](https://doi.org/10.1603/0013-8746(2006)99%5b673:CPOTFA%5d2.0.CO;2)

Seal, J. N., & Tschinkel, W. R. (2007a). Co-evolution and the superorganism: Switching cultivars does not alter the performance of fungus-gardening ant colonies. *Functional Ecology*, *21*(5), 988–997. Scopus. <https://doi.org/10.1111/j.1365-2435.2007.01294.x>

Seal, J. N., & Tschinkel, W. R. (2007b). Complexity in an obligate mutualism: Do fungus-gardening ants know what makes their garden grow? *Behavioral Ecology and Sociobiology*, *61*(8), 1151–1160. Scopus. <https://doi.org/10.1007/s00265-006-0328-4>

Seal, J. N., & Tschinkel, W. R. (2007c). Energetics of newly-mated queens and colony founding in the fungus-gardening ants Cyphomyrmex rimosus and Trachymyrmex septentrionalis (Hymenoptera: Formicidae). *Physiological Entomology*, *32*(1), 8–15. Scopus. <https://doi.org/10.1111/j.1365-3032.2006.00534.x>

Seal, J. N., & Tschinkel, W. R. (2010). Distribution of the fungus-gardening ant (Trachymyrmex septentrionalis) during and after a record drought. *Insect Conservation and Diversity*, *3*(2), 134–142. Scopus. <https://doi.org/10.1111/j.1752-4598.2010.00085.x>

Seaman, F. C. (1984). The effects of tannic acid and other phenolics on the growth of the fungus cultivated by the leaf-cutting ant, Myrmicocrypta buenzlii. *Biochemical Systematics and Ecology*, *12*(2), 155–158. Scopus. <https://doi.org/10.1016/0305-1978(84)90028-0>

Sebayang, A. H., Masjuki, H. H., Ong, H. C., Dharma, S., Silitonga, A. S., Kusumo, F., & Milano, J. (2017). Optimization of bioethanol production from sorghum grains using artificial neural networks integrated with ant colony. *INDUSTRIAL CROPS AND PRODUCTS*, *97*, 146–155. <https://doi.org/10.1016/j.indcrop.2016.11.064>

Seipke, R. F., Barke, J., Brearley, C., Hill, L., Yu, D. W., Goss, R. J. M., & Hutchings, M. I. (2011). A single Streptomyces symbiont makes multiple antifungals to support the fungus farming ant acromyrmex octospinosus. *PLoS ONE*, *6*(8). Scopus. <https://doi.org/10.1371/journal.pone.0022028>

Seipke, R. F., Barke, J., Ruiz-Gonzalez, M. X., Orivel, J., Yu, D. W., & Hutchings, M. I. (2012). Fungus-growing Allomerus ants are associated with antibiotic-producing actinobacteria. *Antonie van Leeuwenhoek, International Journal of General and Molecular Microbiology*, *101*(2), 443–447. Scopus. <https://doi.org/10.1007/s10482-011-9621-y>

Seipke, R. F., Grüschow, S., Goss, R. J. M., & Hutchings, M. I. (2012). Isolating antifungals from fungus-growing ant symbionts using a genome-guided chemistry approach. In *Methods Enzymol.* (Vol. 517, pp. 47–70). Academic Press Inc.; Scopus. <https://doi.org/10.1016/B978-0-12-404634-4.00003-6>

Sekhon, A. S., Kaufman, L., Moledina, N., Summerbell, R. C., Padhye, A. A., Ambrosie, E. A., & Panter, T. (1995). AN EXOANTIGEN TEST FOR THE RAPID IDENTIFICATION OF MEDICALLY SIGNIFICANT FUSARIUM SPECIES. *JOURNAL OF MEDICAL AND VETERINARY MYCOLOGY*, *33*(5), 287–289.

Semenova, T. A., Hughes, D. P., Boomsma, J. J., & Schiøtt, M. (2011). Evolutionary patterns of proteinase activity in attine ant fungus gardens. *BMC Microbiology*, *11*. Scopus. <https://doi.org/10.1186/1471-2180-11-15>

Sen, R., Ishak, H. D., Estrada, D., Dowd, S. E., Hong, E., & Mueller, U. G. (2009). Generalized antifungal activity and 454-screening of Pseudonocardia and Amycolatopsis bacteria in nests of fungus-growing ants. *Proceedings of the National Academy of Sciences of the United States of America*, *106*(42), 17805–17810. Scopus. <https://doi.org/10.1073/pnas.0904827106>

Senna, C. C., Sena, F. P., da Paz, J. S., de Barros Rios, A., Ferraz, C. M., Lenz, D., Soares, F. E. F., Tobias, F. L., Hiura, E., de Araújo, J. V., & Braga, F. R. (2018). Colonization and destruction of ants of the genus Camponotus sp. (Hymenoptera: Formicidae) in vitro by the fungus Pochonia chlamydosporia in the southeast region of Brazil. *3 Biotech*, *8*(8). Scopus. <https://doi.org/10.1007/s13205-018-1365-1>

Senula, S. F., Scavetta, J. T., Banta, J. A., Mueller, U. G., Seal, J. N., & Kellner, K. (2019). Potential Distribution of Six North American Higher-Attine Fungus-Farming Ant (Hymenoptera: Formicidae) Species. *Journal of Insect Science (Online)*, *19*(6). Scopus. <https://doi.org/10.1093/jisesa/iez118>

Shang, H. M., Song, H., Jiang, Y. Y., Ding, G. D., Xing, Y. L., Niu, S. L., Wu, B., & Wang, L. N. (2014). Influence of fermentation concentrate of Hericium caput-medusae (Bull.: Fr.) Pers. On performance, antioxidant status, and meat quality in broilers. *ANIMAL FEED SCIENCE AND TECHNOLOGY*, *198*, 166–175. <https://doi.org/10.1016/j.anifeedsci.2014.09.011>

Shik, J. Z., Gomez, E. B., Kooij, P. W., Santos, J. C., Wcislo, W. T., & Boomsma, J. J. (2016). Nutrition mediates the expression of cultivar-farmer conflict in a fungus-growing ant. *Proceedings of the National Academy of Sciences of the United States of America*, *113*(36), 10121–10126. Scopus. <https://doi.org/10.1073/pnas.1606128113>

Shoji, H., Horiuchi, H., & Takagi, M. (1999). Production of recombinant Der fI (a major mite allergen) by Aspergillus oryzae. *BIOSCIENCE BIOTECHNOLOGY AND BIOCHEMISTRY*, *63*(4), 703–709. <https://doi.org/10.1271/bbb.63.703>

Siedlecki, I., Gorczak, M., Okrasińska, A., & Wrzosek, M. (2021). Chance or necessity—The fungi co−occurring with formica polyctena ants. *Insects*, *12*(3), 1–13. Scopus. <https://doi.org/10.3390/insects12030204>

Siedlecki, I., Piątek, M., Majchrowska, M., Okrasińska, A., Owczarek-Kościelniak, M., & Pawłowska, J. (2023). Discovery of Formicomyces microglobosus gen. Et sp. Nov. Strengthens the hypothesis of independent evolution of ant-associated fungi in Trichomeriaceae. *Fungal Biology*, *127*(12), 1466–1474. Scopus. <https://doi.org/10.1016/j.funbio.2023.10.005>

Silva, A., Bacci Jr, M., Pagnocca, F. C., Bueno, O. C., & Hebling, M. J. A. (2006a). Production of polysaccharidases in different carbon sources by Leucoagaricus gongylophorus Möller (Singer), the symbiotic fungus of the leaf-cutting ant Atta sexdens Linnaeus. *Current Microbiology*, *53*(1), 68–71. Scopus. <https://doi.org/10.1007/s00284-005-0431-1>

Silva, A., Bacci Jr, M., Pagnocca, F. C., Bueno, O. C., & Hebling, M. J. A. (2006b). Starch metabolism in Leucoagaricus gongylophorus, the symbiotic fungus of leaf-cutting ants. *Microbiological Research*, *161*(4), 299–303. Scopus. <https://doi.org/10.1016/j.micres.2005.11.001>

Silva, E. S., Marchi, R. C., Matos, C. S. P., Silva, M. F. G. F., Fernandes, J. B., Bueno, O. C., & Carlos, R. M. (2021). Insecticidal and fungicidal activity of a magnesium compound containing isovanillic acid against leaf-cutting ant and its symbiotic fungus. *Quimica Nova*, *44*(3), 267–271. Scopus. <https://doi.org/10.21577/0100-4042.20170693>

Silva-Pinhati, A. C. O., Bacci Jr, M., Siqueira, C. G., Silva, A., Pagnocca, F. C., Bueno, O. C., & Hebling, M. J. A. (2005). Isolation and maintenance of symbiotic fungi of ants in the tribe Attini (Hymenoptera: Formicidae). *Neotropical Entomology*, *34*(1), 1–5. Scopus. <https://doi.org/10.1590/S1519-566X2005000100001>

Silva-Pinhati, A. C. O., Bacci, M., Hinkle, G., Sogin, M. L., Pagnocca, F. C., Martins, V. G., Bueno, O. C., & Hebling, M. J. A. (2004). Low variation in ribosomal DNA and internal transcribed spacers of the symbiotic fungi of leaf-cutting ants (Attini: Formicidae). *Brazilian Journal of Medical and Biological Research*, *37*(10), 1463–1472. Scopus. <https://doi.org/10.1590/S0100-879X2004001000004>

Singer, T. L., & Espelie, K. E. (1998). Nest and nestmate recognition by a fungus-growing ant, Apterostigma collare emery (Hymenoptera: Formicidae). *Ethology*, *104*(11), 929–939. Scopus. <https://doi.org/10.1111/j.1439-0310.1998.tb00042.x>

Sinotte, V. M., Freedman, S. N., Ugelvig, L. V., & Seid, M. A. (2018). Camponotusfloridanus Ants Incur a Trade-Off between Phenotypic Development and Pathogen Susceptibility from Their Mutualistic Endosymbiont Blochmannia. *INSECTS*, *9*(2). <https://doi.org/10.3390/insects9020058>

Sinski, J. T., Adrouny, G. A., Derbes, V. J., & Jung, R. C. (1959). FURTHER CHARACTERIZATION OF HEMOLYTIC COMPONENT OF FIRE ANT VENOM, MYCOLOGICAL ASPECTS. *PROCEEDINGS OF THE SOCIETY FOR EXPERIMENTAL BIOLOGY AND MEDICINE*, *102*(3), 659–662.

Sit, C. S., Ruzzini, A. C., Van Arnam, E. B., Ramadhar, T. R., Currie, C. R., & Clardy, J. (2015). Variable genetic architectures produce virtually identical molecules in bacterial symbionts of fungus-growing ants. *Proceedings of the National Academy of Sciences of the United States of America*, *112*(43), 13150–13154. Scopus. <https://doi.org/10.1073/pnas.1515348112>

Slater, J. L., Gregson, L., Denning, D. W., & Warn, P. A. (2011). Pathogenicity of Aspergillus fumigatus mutants assessed in Galleria mellonella matches that in mice. *MEDICAL MYCOLOGY*, *49*, S107–S113. <https://doi.org/10.3109/13693786.2010.523852>

Snyder, S. R., Crist, T. O., & Friese, C. F. (2002). Variability in soil chemistry and arbuscular mycorrhizal fungi in harvester ant nests: The influence of topography, grazing and region. *Biology and Fertility of Soils*, *35*(6), 406–413. Scopus. <https://doi.org/10.1007/s00374-002-0487-z>

Sobczak, J. F., Costa, L. F. A., Carvalho, J. L. V. R., Salgado-Neto, G., Moura-Sobczak, J. C. M. S., & Messas, Y. F. (2017). The zombie ants parasitized by the fungi Ophiocordyceps camponoti-atricipis (Hypocreales: Ophiocordycipitaceae): New occurrence and natural history. *Mycosphere*, *8*(9), 1261–1266. Scopus. <https://doi.org/10.5943/mycosphere/8/9/1>

Solomon, S. E., Lopes, C. T., Mueller, U. G., Rodrigues, A., Sosa-Calvo, J., Schultz, T. R., & Vasconcelos, H. L. (2011). Nesting biology and fungiculture of the fungus-growing ant, Mycetagroicus cerradensis: New light on the origin of higher attine agriculture. *Journal of Insect Science*, *11*. Scopus. <https://doi.org/10.1673/031.011.0112>

Solomon, S. E., Mueller, U. G., Schultz, T. R., Currie, C. R., Price, S. L., Oliveira Da Silva-Pinhati, A. C., Bacci Jr, M., & Vasconcelos, H. L. (2004). Nesting biology of the fungus growing ants Mycetarotes Emery (Attini, Formicidae). *Insectes Sociaux*, *51*(4), 333–338. Scopus. <https://doi.org/10.1007/s00040-004-0742-4>

Somera, A. F., Lima, A. M., Dos Santos-Neto, A. J., Lanças, F. M., & Bacci, M. (2015). Leaf-cutter ant fungus gardens are biphasic mixed microbial bioreactors that convert plant biomass to polyols with biotechnological applications. *Applied and Environmental Microbiology*, *81*(13), 4525–4535. Scopus. <https://doi.org/10.1128/AEM.00046-15>

Sosa-Calvo, J., Brady, S. G., & Schultz, T. R. (2009). The gyne of the enigmatic fungus-fanning ant species Mycetosoritis explicata. *Journal of Hymenoptera Research*, *18*(1), 113–120. Scopus.

Sosa-Calvo, J., Fernández, F., & Schultz, T. R. (2019). Phylogeny and evolution of the cryptic fungus-farming ant genus Myrmicocrypta F. Smith (Hymenoptera: Formicidae) inferred from multilocus data. *Systematic Entomology*, *44*(1), 139–162. Scopus. <https://doi.org/10.1111/syen.12313>

Sosa-Calvo, J., Ješovnik, A., Lopes, C. T., Rodrigues, A., Rabeling, C., Bacci, M., Vasconcelos, H. L., & Schultz, T. R. (2017). Biology of the relict fungus-farming ant Apterostigma megacephala Lattke, including descriptions of the male, gyne, and larva. *Insectes Sociaux*, *64*(3), 329–346. Scopus. <https://doi.org/10.1007/s00040-017-0550-2>

Sosa-Calvo, J., Jesovnik, A., Okonski, E., & Schultz, T. R. (2015). Locating, collecting, and maintaining colonies of fungus-farming ants (Hymenoptera: Formicidae: Myrmicinae: Attini). *Sociobiology*, *62*(2), 300–320. Scopus. <https://doi.org/10.13102/sociobiology.v62i2.300-320>

Sosa-Calvo, J., Ješovnik, A., Vasconcelos, H. L., Bacci, M., & Schultz, T. R. (2017). Rediscovery of the enigmatic fungus-farming ant “Mycetosoritis” asper Mayr (Hymenoptera: Formicidae): Implications for taxonomy, phylogeny, and the evolution of agriculture in ants. *PLoS ONE*, *12*(5). Scopus. <https://doi.org/10.1371/journal.pone.0176498>

Sosa-Calvo, J., & Schultz, T. R. (2010). Three remarkable new fungus-growing ant species of the genus Myrmicocrypta (Hymenoptera: Formicidae), with a reassessment of the characters that define the genus and its position within the attini. *Annals of the Entomological Society of America*, *103*(2), 181–195. Scopus. <https://doi.org/10.1603/AN09108>

Sosa-Calvo, J., Schultz, T. R., Brandão, C. R. F., Klingenberg, C., Feitosa, R. M., Rabeling, C., Bacci Jr, M., Lopes, C. T., & Vasconcelos, H. L. (2013). Cyatta abscondita: Taxonomy, evolution, and natural history of a new fungus-farming ant genus from Brazil. *PLoS ONE*, *8*(11). Scopus. <https://doi.org/10.1371/journal.pone.0080498>

Sosa-Calvo, J., Schultz, T. R., JeŠovnik, A., Dahan, R. A., & Rabeling, C. (2018). Evolution, systematics, and natural history of a new genus of cryptobiotic fungus-growing ants. *Systematic Entomology*, *43*(3), 549–567. Scopus. <https://doi.org/10.1111/syen.12289>

Sousa, K. K. A., Catalani, G. C., Gianeti, T. M. R., Camargo, R. S., Caldato, N., Ramos, V. M., & Forti, L. C. (2020). A volatile semiochemical released by the fungus garden of leaf-cutting ants. *Florida Entomologist*, *103*(1), 1–8. Scopus. <https://doi.org/10.1653/024.103.0401>

Souza, U. P., Cabrera, S. P., da Silva, T. M. G., da Silva, E. M. S., Camara, C. A., & Silva, T. M. S. (2019). Geopropolis gel for the adjuvant treatment of candidiasis—Formulation and in vitro release assay. *REVISTA BRASILEIRA DE FARMACOGNOSIA-BRAZILIAN JOURNAL OF PHARMACOGNOSY*, *29*(3), 278–286. <https://doi.org/10.1016/j.bjp.2019.02.010>

Spor, A., Wang, S., Dillmann, C., de Vienne, D., & Sicard, D. (2008). "Ant’’ and "Grasshopper’’ Life-History Strategies in Saccharomyces cerevisiae. *PLOS ONE*, *3*(2). <https://doi.org/10.1371/journal.pone.0001579>

Stan, T., Teodor, E. D., Gatea, F., Chifiriuc, M. C., & Lazar, V. (2017). Antioxidant and antifungal activity of Romanian propolis. *ROMANIAN BIOTECHNOLOGICAL LETTERS*, *22*(6), 13116–13124.

Stefanelli, L. E. P., Filho, T. M. M. M., Camargo, R. D. S., de Matos, C. A. O., & Forti, L. C. (2021). Effects of entomopathogenic fungi on individuals as well as groups of workers and immatures of atta sexdens rubropilosa leaf-cutting ants. *Insects*, *11*(1), 1–13. Scopus. <https://doi.org/10.3390/insects12010010>

Stefanelli, L. E. P., Mota, T. M. M., Camargo, R. D., de Matos, C. A. O., & Forti, L. C. (2021). Effects of Entomopathogenic Fungi on Individuals as Well as Groups of Workers and Immatures of Atta sexdens rubropilosa Leaf-Cutting Ants. *INSECTS*, *12*(1). <https://doi.org/10.3390/insects12010010>

Stimac, J. L., Pereira, R. M., Alves, S. B., & Wood, L. A. (1993a). BEAUVERIA-BASSIANA (BALSAMO) VUILLEMIN (DEUTEROMYCETES) APPLIED TO LABORATORY COLONIES OF SOLENOPSIS-INVICTA BUREN (HYMENOPTERA, FORMICIDAE) IN SOIL. *JOURNAL OF ECONOMIC ENTOMOLOGY*, *86*(2), 348–352. <https://doi.org/10.1093/jee/86.2.348>

Stimac, J. L., Pereira, R. M., Alves, S. B., & Wood, L. A. (1993b). MORTALITY IN LABORATORY COLONIES OF SOLENOPSIS-INVICTA (HYMENOPTERA, FORMICIDAE) TREATED WITH BEAUVERIA-BASSIANA (DEUTEROMYCETES). *JOURNAL OF ECONOMIC ENTOMOLOGY*, *86*(4), 1083–1087. <https://doi.org/10.1093/jee/86.4.1083>

Storey, G. K., Vander Meer, R. K., Boucias, D. G., & McCoy, C. W. (1991). Effect of fire ant (Solenopsis invicta) venom alkaloids on the in vitro germination and development of selected entomogenous fungi. *Journal of Invertebrate Pathology*, *58*(1), 88–95. Scopus. <https://doi.org/10.1016/0022-2011(91)90166-N>

Stradling, D. J., & Powell, R. J. (1986). The cloning of more highly productive fungal strains: A factor in the speciation of fungus-growing ants. *Experientia*, *42*(8), 962–964. Scopus. <https://doi.org/10.1007/BF01941781>

Stucki, D., Sundström, L., & Freitak, D. (2017). Caste-specific expression of constitutive and Beauveria bassiana induced immunity in the ant Formica exsecta (Hymenoptera: Formicidae). *MYRMECOLOGICAL NEWS*, *25*, 83–93.

Stürup, M., Baer, B., & Boomsma, J. J. (2014). Short independent lives and selection for maximal sperm survival make investment in immune defences unprofitable for leaf-cutting ant males. *BEHAVIORAL ECOLOGY AND SOCIOBIOLOGY*, *68*(6), 947–955. <https://doi.org/10.1007/s00265-014-1707-x>

Suárez, A. M., Núñez, E. M. D., Cruz, I. G., Ardisana, E. F. H., Domínguez, Y., Guerrero-Morales, S., Medina, J. A. C., Bonilla, C. M. E., & Pérez-Alvarez, S. (2022). Isolation and characterization of one autochthonous Beauveria sp. Isolate from Cuban fields. *BIOTECNIA*, *24*(1), 142–150.

Suen, G., Scott, J. J., Aylward, F. O., & Currie, C. R. (2011). The Microbiome of Leaf-Cutter Ant Fungus Gardens. In *Handb. Of Mol. Microb. Ecol. II: Metagenomics in Differ. Habitats* (pp. 367–379). John Wiley and Sons; Scopus. <https://doi.org/10.1002/9781118010549.ch36>

Sun, P. F., Chen, P. H., Lin, W. J., Lin, C. C., & Chou, J. Y. (2018). Variation in the ability of fungi in the extrafloral nectar of Mallotus paniculatus to attract ants as plant defenders. *Mycosphere*, *9*(2), 178–188. Scopus. <https://doi.org/10.5943/mycosphere/9/2/2>

Swift, R. J., Craig, S. H., Wiebe, M. G., Robson, G. D., & Trinci, A. P. J. (2000). Evolution of Aspergillus niger and A-nidulans in glucose-limited chemostat cultures, as indicated by oscillations in the frequency of cycloheximide resistant and morphological mutants. *MYCOLOGICAL RESEARCH*, *104*, 333–337. <https://doi.org/10.1017/S0953756299001136>

Szentiványi, T., Haelewaters, D., Rádai, Z., Mizsei, E., Pfliegler, W. P., Báthori, F., Tartally, A., Christe, P., & Glaizot, O. (2019). Climatic effects on the distribution of ant- and bat fly-associated fungal ectoparasites (Ascomycota, Laboulbeniales). *Fungal Ecology*, *39*, 371–379. Scopus. <https://doi.org/10.1016/j.funeco.2019.03.003>

Takahashi, K., Takai, T., Yasuhara, T., Yokota, T., & Okumura, Y. (2001). Effects of site-directed mutagenesis in the cysteine residues and the N-glycosylation motif in recombinant Der f 1 on secretion and protease activity. *INTERNATIONAL ARCHIVES OF ALLERGY AND IMMUNOLOGY*, *124*(4), 454–460. <https://doi.org/10.1159/000053780>

Takai, T., Mizuuchi, E., Kikuchi, Y., Nagamune, T., Okumura, K., & Ogawa, H. (2006). Glycosylation of recombinant proforms of major house dust mite allergens Der p 1 and Der f 1 decelerates the speed of maturation. *INTERNATIONAL ARCHIVES OF ALLERGY AND IMMUNOLOGY*, *139*(3), 181–187. <https://doi.org/10.1159/000091163>

Tang, D., Huang, O., Zou, W., Wang, Y., Wang, Y., Dong, Q., Sun, T., Yang, G., & Yu, H. (2023). Six new species of zombie-ant fungi from Yunnan in China. *IMA Fungus*, *14*(1). Scopus. <https://doi.org/10.1186/s43008-023-00114-9>

Tang, D., Xu, Z., Wang, Y., Wang, Y., Tran, N. L., & Yu, H. (2023). Multigene phylogeny and morphology reveal two novel zombie-ant fungi in Ophiocordyceps (Ophiocordycipitaceae, Hypocreales). *Mycological Progress*, *22*(4). Scopus. <https://doi.org/10.1007/s11557-023-01874-9>

Tang, D., Zhao, J., Lu, Y., Wang, Z., Sun, T., Liu, Z., & Yu, H. (2023). Morphology, phylogeny and host specificity of two new Ophiocordyceps species belonging to the “zombie-ant fungi” clade (Ophiocordycipitaceae, Hypocreales). *MycoKeys*, *99*, 269–296. Scopus. <https://doi.org/10.3897/mycokeys.99.107565>

Tartally, A., Szabó, N., Somogyi, A. Á., Báthori, F., Haelewaters, D., Mucsi, A., Fürjes-Mikó, Á., & Nash, D. R. (2021). Ectoparasitic fungi of Myrmica ants alter the success of parasitic butterflies. *Scientific Reports*, *11*(1). Scopus. <https://doi.org/10.1038/s41598-021-02800-3>

Taylor, B., & Adedoyin, S. F. (1978). ABUNDANCE AND INTERSPECIFIC RELATIONS OF COMMON ANT SPECIES (HYMENOPTERA-FORMICIDAE) ON COCOA FARMS IN WESTERN NIGERIA. *BULLETIN OF ENTOMOLOGICAL RESEARCH*, *68*(1), 105–121. <https://doi.org/10.1017/S0007485300007197>

Teixeira, G. A., Barros, L. A. C., de Aguiar, H. J. A. C., & Lopes, D. M. (2021). Distribution of GC-rich heterochromatin and ribosomal genes in three fungus-farming ants (Myrmicinae, Attini, Attina): Insights on chromosomal evolution. *Comparative Cytogenetics*, *15*(4), 413–428. Scopus. <https://doi.org/10.3897/compcytogen.v15.i4.73769>

Teixeira, G. A., Jacintho, G. D. F., De Aguiar, H. J. A. C., Lopes, D. M., & Barros, L. A. C. (2023). Cytogenetic Analysis of the Fungus-Farming Ant Cyphomyrmex rimosus (Spinola, 1851) (Formicidae: Myrmicinae: Attini) Highlights Karyotypic Variation. *Cytogenetic and Genome Research*, *162*(10), 579–585. Scopus. <https://doi.org/10.1159/000529607>

Tejeda, C. S., García, A. A., Torres, B. C. P., & Olguín, J. F. L. (2017). Agroecological Alternative for Managing Atta mexicana in Puebla, Mexico. *SOUTHWESTERN ENTOMOLOGIST*, *42*(1), 261–273.

Téné, N., Bonnafé, E., Berger, F., Rifflet, A., Guilhaudis, L., Ségalas-Milazzo, I., Pipy, B., Coste, A., Leprince, J., & Treilhou, M. (2016). Biochemical and biophysical combined study of bicarinalin, an ant venom antimicrobial peptide. *PEPTIDES*, *79*, 103–113. <https://doi.org/10.1016/j.peptides.2016.04.001>

Terezan, A. P., Rossi, R. A., Almeida, R. N. A., Freitas, T. G., Fernandes, J. B., Da Silva, M. F. D. G. F., Vieira, P. C., Bueno, O. C., Pagnocca, F. C., & Pirani, J. R. (2010). Activities of extracts and compounds from Spiranthera odoratissima St. Hil. (Rutaceae) in leaf-cutting ants and their symbiotic fungus. *Journal of the Brazilian Chemical Society*, *21*(5), 882–886. Scopus. <https://doi.org/10.1590/S0103-50532010000500016>

Theis, F. J., Ugelvig, L. V., Marr, C., & Cremer, S. (2015). Opposing effects of allogrooming on disease transmission in ant societies. *PHILOSOPHICAL TRANSACTIONS OF THE ROYAL SOCIETY B-BIOLOGICAL SCIENCES*, *370*(1669). <https://doi.org/10.1098/rstb.2014.0108>

Tian, X. F., Cao, H., Yan, J. T., Li, C. Y., Li, F. Y., Li, Y. K., Huang, F., Bao, C. L., Cao, Y. H., & Rao, Z. H. (2024). Effect of dietary supplementation with recombinant human lysozyme on growth performance, antioxidative characteristics, and intestinal health in broiler chickens. *JOURNAL OF ANIMAL SCIENCE*, *102*. <https://doi.org/10.1093/jas/skae121>

Tin, M. M. Y., Rheindt, F. E., Cros, E., & Mikheyev, A. S. (2015). Degenerate adaptor sequences for detecting PCR duplicates in reduced representation sequencing data improve genotype calling accuracy. *MOLECULAR ECOLOGY RESOURCES*, *15*(2), 329–336. <https://doi.org/10.1111/1755-0998.12314>

Torres, J. A., Santiago, M., & Salgado, M. (1999). The effects of the fungus-growing ant, Trachymyrmex jamaicensis, on soil fertility and seed germination in a subtropical dry forest. *Tropical Ecology*, *40*(2), 237–245. Scopus.

Tragust, S., Feldhaar, H., Espadaler, X., & Pedersen, J. S. (2015). Rapid increase of the parasitic fungus Laboulbenia formicarum in supercolonies of the invasive garden ant Lasius neglectus. *Biological Invasions*, *17*(10), 2795–2801. Scopus. <https://doi.org/10.1007/s10530-015-0917-0>

Tragust, S., Mitteregger, B., Barone, V., Konrad, M., Ugelvig, L. V., & Cremer, S. (2013). Ants disinfect fungus-exposed brood by oral uptake and spread of their poison. *Current Biology*, *23*(1), 76–82. Scopus. <https://doi.org/10.1016/j.cub.2012.11.034>

Tragust, S., Tartally, A., Espadaler, X., & Billen, J. (2016). Histopathology of Laboulbeniales (Ascomycota: Laboulbeniales): Ectoparasitic fungi on ants (Hymenoptera: Formicidae). *Myrmecological News*, *23*, 81–89. Scopus.

Tranter, C., Graystock, P., Shaw, C., Lopes, J. F. S., & Hughes, W. O. H. (2014). Sanitizing the fortress: Protection of ant brood and nest material by worker antibiotics. *BEHAVIORAL ECOLOGY AND SOCIOBIOLOGY*, *68*(3), 499–507. <https://doi.org/10.1007/s00265-013-1664-9>

Trinh, T., Ouellette, R., & de Bekker, C. (2021). Getting lost: The fungal hijacking of ant foraging behaviour in space and time. *ANIMAL BEHAVIOUR*, *181*, 165–184. <https://doi.org/10.1016/j.anbehav.2021.09.003>

Tschinkel, W. R., & Seal, J. N. (2016). Bioturbation by the fungus-gardening ant, Trachymyrmex septentrionalis. *PLoS ONE*, *11*(7). Scopus. <https://doi.org/10.1371/journal.pone.0158920>

Tunvongvinis, T., Jaitrong, W., Suriyachadkun, C., Sripreechasak, P., Tanasupawat, S., & Phongsopitanun, W. (2024). Streptomyces odontomachi sp. Nov., a novel actinobacterium with antimicrobial potential isolated from ants (Odontomachus simillimus Smith, 1858). *JOURNAL OF ANTIBIOTICS*. <https://doi.org/10.1038/s41429-024-00766-8>

Turner, J., & Hughes, W. O. H. (2018). The effect of parasitism on personality in a social insect. *BEHAVIOURAL PROCESSES*, *157*, 532–539. <https://doi.org/10.1016/j.beproc.2018.06.004>

Ugelvig, L. V., Kronauer, D. J. C., Schrempf, A., Heinze, J., & Cremer, S. (2010). Rapid anti-pathogen response in ant societies relies on high genetic diversity. *PROCEEDINGS OF THE ROYAL SOCIETY B-BIOLOGICAL SCIENCES*, *277*(1695), 2821–2828. <https://doi.org/10.1098/rspb.2010.0644>

Valadares, L., & do Nascimento, F. S. (2017). Changes in the cuticular hydrocarbon profile associated with the molting cycle correlate with the hydrocarbon profile of the fungus cultivated by the ant Atta sexdens. *Insectes Sociaux*, *64*(4), 591–596. Scopus. <https://doi.org/10.1007/s00040-017-0581-8>

Valencia-Giraldo, S. M., Castaño-Quintana, K., Giraldo-Echeverri, C., Armbrecht, I., & Montoya-Lerma, J. (2020). Refuse dumps in Atta cephalotes (Hymenoptera: Myrmicinae) nests as a source of native entomopathogens for biological control. *BIOCONTROL SCIENCE AND TECHNOLOGY*, *30*(2), 132–142. <https://doi.org/10.1080/09583157.2019.1695102>

Valencia-Giraldo, S. M., Gutiérrez-Urrego, A., Niño-Castro, A., López-Pena, A., & Montoya-Lerma, J. (2024). Bacterial microbiota associated with the leaf-cutting ant Atta cephalotes (Hymenoptera: Myrmicinae): Dynamics during development and potential role in defence. *PHYSIOLOGICAL ENTOMOLOGY*, *49*(1), 23–38. <https://doi.org/10.1111/phen.12423>

Valles, S. M., & Pereira, R. M. (2005). Solenopsis invicta transferrin: cDNA cloning, gene architecture, and up-regulation in response to Beauveria bassiana infection. *GENE*, *358*, 60–66. <https://doi.org/10.1016/j.gene.2005.05.017>

Van Bael, S. A., Fernández-Marín, H., Valencia, M. C., Rojas, E. I., Wcislo, W. T., & Herre, E. A. (2009). Two fungal symbioses collide: Endophytic fungi are not welcome in leaf-cutting ant gardens. *Proceedings of the Royal Society B: Biological Sciences*, *276*(1666), 2419–2426. Scopus. <https://doi.org/10.1098/rspb.2009.0196>

Van Bael, S. A., Seid, M. A., & Wcislo, W. T. (2012). Endophytic fungi increase the processing rate of leaves by leaf-cutting ants (Atta). *Ecological Entomology*, *37*(4), 318–321. Scopus. <https://doi.org/10.1111/j.1365-2311.2012.01364.x>

Varanda-Haifig, S. S., Albarici, T. R., Nunes, P. H., Haifig, I., Vieira, P. C., & Rodrigues, A. (2017). Nature of the interactions between hypocrealean fungi and the mutualistic fungus of leaf-cutter ants. *Antonie van Leeuwenhoek, International Journal of General and Molecular Microbiology*, *110*(4), 593–605. Scopus. <https://doi.org/10.1007/s10482-016-0826-y>

Vasconcelos, H. L., Araújo, B. B., & Mayhé-Nunes, A. J. (2008). Patterns of diversity and abundance of fungus-growing ants (Formicidae: Attini) in areas of the Brazilian Cerrado. *Revista Brasileira de Zoologia*, *25*(3), 445–450. Scopus. <https://doi.org/10.1590/S0101-81752008000300009>

Verkhusha, V. V., Shavlovsky, M. M., Nevzglyadova, O. V., Gaivoronsky, A. A., Artemov, A. V., Stepanenko, O. V., Kuznetsova, I. M., & Turoverov, K. K. (2003). Expression of recombinant GFP-actin fusion protein in the methylotrophic yeast Pichia pastoris. *FEMS YEAST RESEARCH*, *3*(1), 105–111. <https://doi.org/10.1111/j.1567-1364.2003.tb00145.x>

Viana, A. M. M., Frézard, A., Malosse, C., Della Lucia, T. M. C., Errard, C., & Lenoir, A. (2001). Colonial recognition of fungus in the fungus-growing ant Acromyrmex subterraneus subterraneus (Hymenoptera: Formicidae). *Chemoecology*, *11*(1), 29–36. Scopus. <https://doi.org/10.1007/PL00001829>

Victor, S. R., Crisóstomo, F. R., Bueno, F. C., Pagnocca, F. C., Fernandes, J. B., Correa, A. G., Bueno, O. C., José A Hebling, M., Jr, M. B., Vieira, P. C., & Fátima G F Da Silva, M. (2001). Toxicity of synthetic piperonyl compounds to leaf-cutting ants and their symbiotic fungus. *Pest Management Science*, *57*(7), 603–608. Scopus. <https://doi.org/10.1002/ps.333>

Vieira, A. S., Bueno, O. C., & Camargo-Mathias, M. I. (2012). Morphophysiological differences between the metapleural glands of fungus-growing and non-fungus-growing ants (Hymenoptera, Formicidae). *PLoS ONE*, *7*(8). Scopus. <https://doi.org/10.1371/journal.pone.0043570>

Vieira, A. S., Bueno, O. C., & Camargo-Mathias, M. I. (2014). The metapleural glands of fungus-growing and non-fungus-growing ants: Ultrastructural study. *Animal Biology*, *64*(3), 277–294. Scopus. <https://doi.org/10.1163/15707563-00002446>

Vieira, A. S., Morgan, E. D., Drijfhout, F. P., & Camargo-Mathias, M. I. (2012). Chemical Composition of Metapleural Gland Secretions of Fungus-Growing and Non-fungus-growing Ants. *Journal of Chemical Ecology*, *38*(10), 1289–1297. Scopus. <https://doi.org/10.1007/s10886-012-0185-8>

Vigueras, G., Paredes-Hernández, D., Revah, S., Valenzuela, J., Olivares-Hernández, R., & Le Borgne, S. (2017). Growth and enzymatic activity of Leucoagaricus gongylophorus, a mutualistic fungus isolated from the leaf-cutting ant Atta mexicana, on cellulose and lignocellulosic biomass. *Letters in Applied Microbiology*, *65*(2), 173–181. Scopus. <https://doi.org/10.1111/lam.12759>

Villesen, P., & Boomsma, J. J. (2003). Patterns of male parentage in the fungus-growing ants. *Behavioral Ecology and Sociobiology*, *53*(4), 246–253. Scopus. <https://doi.org/10.1007/s00265-002-0577-9>

Villesen, P., Gertsch, P. J., & Boomsma, J. J. (2002). Microsatellite primers for fungus-growing ants. *Molecular Ecology Notes*, *2*(3), 320–322. Scopus. <https://doi.org/10.1046/j.1471-8286.2002.00229.x>

Villesen, P., Gertsch, P. J., Frydenberg, J., Mueller, U. G., & Boomsma, J. J. (1999). Evolutionary transition from single to multiple mating in fungus-growing ants. *Molecular Ecology*, *8*(11), 1819–1825. Scopus. <https://doi.org/10.1046/j.1365-294X.1999.00767.x>

Villesen, P., Mueller, U. G., Schultz, T. R., Adams, R. M. M., & Bouck, A. C. (2004). Evolution of ant-cultivar specialization and cultivar switching in Apterostigma fungus-growing ants. *Evolution*, *58*(10), 2252–2265. Scopus. <https://doi.org/10.1111/j.0014-3820.2004.tb01601.x>

Villesen, P., Murakami, T., Schultz, T. R., & Boomsma, J. J. (2002). Identifying the transition between single and multiple mating of queens in fungus-growing ants. *Proceedings of the Royal Society B: Biological Sciences*, *269*(1500), 1541–1548. Scopus. <https://doi.org/10.1098/rspb.2002.2044>

Visagie, C. M., Renaud, J. B., Burgess, K. M. N., Malloch, D. W., Clark, D., Ketch, L., Urb, M., Louis-Seize, G., Assabgui, R., Sumarah, M. W., & Seifert, K. A. (2016). Fifteen new species of Penicillium. *PERSOONIA*, *36*, 247–280. <https://doi.org/10.3767/003158516X691627>

Vo, T. L., Mueller, U. G., & Mikheyev, A. S. (2009). Free-living fungal symbionts (Lepiotaceae) of fungus-growing ants (Attini: Formicidae). *Mycologia*, *101*(2), 206–210. Scopus. <https://doi.org/10.3852/07-055>

Walker, T. N., & Hughes, W. O. H. (2009). Adaptive social immunity in leaf-cutting ants. *BIOLOGY LETTERS*, *5*(4), 446–448. <https://doi.org/10.1098/rsbl.2009.0107>

Waller, D. A. (2023). Ecological Similarities of Fungus-growing Ants (Attini) and Termites (Macrotermitinae). In *Advances in Myrmecology* (pp. 337–345). Brill; Scopus. <https://www.scopus.com/inward/record.uri?eid=2-s2.0-85200849499&partnerID=40&md5=c6781df5f6e1b2add5459657f5742d7e>

Wang, L., Elliott, B., Jin, X. X., Zeng, L., & Chen, J. (2015). Antimicrobial properties of nest volatiles in red imported fire ants, Solenopsis invicta (hymenoptera: Formicidae). *SCIENCE OF NATURE*, *102*(11–12). <https://doi.org/10.1007/s00114-015-1316-1>

Wang, M., Kornsakulkarn, J., Srichomthong, K., Feng, T., Liu, J. K., Isaka, M., & Thongpanchang, C. (2019). Antimicrobial anthraquinones from cultures of the ant pathogenic fungus Cordyceps morakotii BCC 56811. *Journal of Antibiotics*, *72*(3), 141–147. Scopus. <https://doi.org/10.1038/s41429-018-0135-y>

Wang, Y., Mueller, U. G., & Clardy, J. (1999). Antifungal diketopiperazines from symbiotic fungus of fungus-growing ant Cyphomyrmex minutus. *Journal of Chemical Ecology*, *25*(4), 935–941. Scopus. <https://doi.org/10.1023/A:1020861221126>

Wasuwan, R., Phosrithong, N., Promdonkoy, B., Sangsrakru, D., Sonthirod, C., Tangphatsornruang, S., Likhitrattanapisal, S., Ingsriswang, S., Srisuksam, C., Klamchao, K., Suksangpanomrung, M., Hleepongpanich, T., Reungpatthanaphong, S., Tanticharoen, M., & Amnuaykanjanasin, A. (2022). The Fungus Metarhizium sp. BCC 4849 Is an Effective and Safe Mycoinsecticide for the Management of Spider Mites and Other Insect Pests. *Insects*, *13*(1). Scopus. <https://doi.org/10.3390/insects13010042>

Weber, N. A. (1966). Fungus-growing ants. *Science*, *153*(3736), 587–604. Scopus. <https://doi.org/10.1126/science.153.3736.587>

Wei, Z., Ortiz-Urquiza, A., & Keyhani, N. O. (2021). Altered Expression of Chemosensory and Odorant Binding Proteins in Response to Fungal Infection in the Red Imported Fire Ant, Solenopsis invicta. *FRONTIERS IN PHYSIOLOGY*, *12*. <https://doi.org/10.3389/fphys.2021.596571>

Wetterer, J. K., Schultz, T. R., & Meier, R. (1998). Phylogeny of Fungus-Growing Ants (Tribe Attini) Based on mtDNA Sequence and Morphology. *Molecular Phylogenetics and Evolution*, *9*(1), 42–47. Scopus. <https://doi.org/10.1006/mpev.1997.0466>

Will, I., Attardo, G. M., & de Bekker, C. (2023). Multiomic interpretation of fungus-infected ant metabolomes during manipulated summit disease. *Scientific Reports*, *13*(1). Scopus. <https://doi.org/10.1038/s41598-023-40065-0>

Will, I., Beckerson, W. C., & de Bekker, C. (2023). Using machine learning to predict protein–protein interactions between a zombie ant fungus and its carpenter ant host. *Scientific Reports*, *13*(1). Scopus. <https://doi.org/10.1038/s41598-023-40764-8>

Williams, P. B., Siegel, C., & Portnoy, J. (2001). Efficacy of a single diagnostic test for sensitization to common inhalant allergens. *ANNALS OF ALLERGY ASTHMA & IMMUNOLOGY*, *86*(2), 196–202. <https://doi.org/10.1016/S1081-1206(10)62691-9>

Woolfolk, S., Stokes, C. E., Watson, C., Baker, G., Brown, R., & Baird, R. (2016). Fungi associated with solenopsis invicta buren (Red Imported Fire Ant, Hymenoptera: Formicidae) from mounds in Mississippi. *Southeastern Naturalist*, *15*(2), 220–234. Scopus. <https://doi.org/10.1656/058.015.0203>

Wrzosek, M., Dubiel, G., Gorczak, M., Pawlowska, J., Tischer, M., & Balazy, S. (2016). New insights on the phylogeny and biology of the fungal ant pathogen Aegeritella. *JOURNAL OF INVERTEBRATE PATHOLOGY*, *133*, 1–7. <https://doi.org/10.1016/j.jip.2015.11.005>

Wu, H., Batzer, D. P., Yan, X. M., Lu, X. G., & Wu, D. H. (2013). Contributions of ant mounds to soil carbon and nitrogen pools in a marsh wetland of Northeastern China. *APPLIED SOIL ECOLOGY*, *70*, 9–15. <https://doi.org/10.1016/j.apsoil.2013.04.004>

Wu, H. T., Lu, X. G., Tong, S. Z., & Batzer, D. P. (2015). Soil engineering ants increase CO2 and N2O emissions by affecting mound soil physicochemical characteristics from a marsh soil: A laboratory study. *APPLIED SOIL ECOLOGY*, *87*, 19–26. <https://doi.org/10.1016/j.apsoil.2014.11.011>

Wu, H. T., Lu, X. G., Wu, D. H., Song, L. H., Yan, X. M., & Liu, J. (2013). Ant mounds alter spatial and temporal patterns of CO2, CH4 and N2O emissions from a marsh soil. *SOIL BIOLOGY & BIOCHEMISTRY*, *57*, 884–891. <https://doi.org/10.1016/j.soilbio.2012.10.034>

Wu, H. X., Xu, Y. T., Zafar, J., De Mandal, S., Lin, L. J., Lu, Y. Y., Jin, F. L., Pang, R., & Xu, X. X. (2023). Transcriptomic Analysis Reveals the Impact of the Biopesticide Metarhizium anisopliae on the Immune System of Major Workers in Solenopsis invicta. *INSECTS*, *14*(8). <https://doi.org/10.3390/insects14080701>

Wu, Y. Y., Liu, Y. X., Yu, J. Y., Xu, Y. J., & Chen, S. Q. (2022). Observation of the Antimicrobial Activities of Two Actinomycetes in the Harvester Ant Messor orientalis. *INSECTS*, *13*(8). <https://doi.org/10.3390/insects13080691>

Yan, Y. J., An, Y., Wang, X. Z., Chen, Y. Q., Jacob, M. R., Tekwani, B. L., Dai, L. Y., & Li, X. C. (2017). Synthesis and Antimicrobial Evaluation of Fire Ant Venom Alkaloid Based 2-Methyl-6-alkyl-Δ1,6-piperideines. *JOURNAL OF NATURAL PRODUCTS*, *80*(10), 2795–2798. <https://doi.org/10.1021/acs.jnatprod.7b00625>

Yek, S. H., Boomsma, J. J., & Schiott, M. (2013). Differential gene expression in Acromyrmex leaf-cutting ants after challenges with two fungal pathogens. *MOLECULAR ECOLOGY*, *22*(8), 2173–2187. <https://doi.org/10.1111/mec.12255>

Yin, C. M., Fan, X. Z., Liu, C. Y., Fan, Z., Shi, D. F., Yao, F., Cheng, W., & Gao, H. (2019). The Antioxidant Properties, Tyrosinase and α-Glucosidase Inhibitory Activities of Phenolic Compounds in Different Extracts from the Golden Oyster Mushroom, Pleurotus citrinopileatus (Agaricomycetes). *INTERNATIONAL JOURNAL OF MEDICINAL MUSHROOMS*, *21*(9), 865–874. <https://doi.org/10.1615/IntJMedMushrooms.2019031857>

Zafar, J., Wu, H. X., Xu, Y. T., Lin, L. J., Kang, Z. H., Zhang, J., Zhang, R. N., Lu, Y. Y., Jin, F. L., & Xu, X. X. (2023). Transcriptomic Analysis of Metarhizium anisopliae-Induced Immune-Related Long Non-Coding RNAs in Polymorphic Worker Castes of Solenopsis invicta. *INTERNATIONAL JOURNAL OF MOLECULAR SCIENCES*, *24*(18). <https://doi.org/10.3390/ijms241813983>

Zettler, J. A., McInnis Jr, T. M., Allen, C. R., & Spira, T. P. (2002). Biodiversity of fungi in red imported fire ant (Hymenoptera:Formicidae) mounds. *Annals of the Entomological Society of America*, *95*(4), 487–491. Scopus. [https://doi.org/10.1603/0013-8746(2002)095[0487:BOFIRI]2.0.CO;2](https://doi.org/10.1603/0013-8746(2002)095%5b0487:BOFIRI%5d2.0.CO;2)

Zhang, R., Liu, X., & Wei, C. (2022). Dynamic analysis of stochastic delay mutualistic system of leaf-cutter ants with stage structure and their fungus garden. *Journal of Biological Dynamics*, *16*(1), 565–584. Scopus. <https://doi.org/10.1080/17513758.2022.2099590>

Zheng, X. Y., & Sinclair, J. B. (1996). Screening and isolation of mutants of Bacillus megaterium B153-2-2 for motility, chemotaxis, antagonism, and sporulation. *PHYSIOLOGICAL AND MOLECULAR PLANT PATHOLOGY*, *48*(4), 233–244. <https://doi.org/10.1006/pmpp.1996.0020>

Zhigulskaya, Z. A. (2011). ANTS (HYMENOPTERA, FORMICIDAE) OF THE KYRAISKAYA DEPRESSION OF THE SOUTHEASTERN ALTAI. *TOMSK STATE UNIVERSITY JOURNAL*, *350*, 189-+.

Zhigulskaya, Z. A., Shekhovtsov, S. V., Poluboyarova, T. V., & Berman, D. I. (2022). Formica picea and F. candida (Hymenoptera: Formicidae): Synonyms or Two Species? *DIVERSITY-BASEL*, *14*(8). <https://doi.org/10.3390/d14080613>

Zielinska, D. F., Gnad, F., Schropp, K., Wisniewski, J. R., & Mann, M. (2012). Mapping N-Glycosylation Sites across Seven Evolutionarily Distant Species Reveals a Divergent Substrate Proteome Despite a Common Core Machinery. *MOLECULAR CELL*, *46*(4), 542–548. <https://doi.org/10.1016/j.molcel.2012.04.031>

Zucconi, L., Bert, C., Pagano, S., & Mulas, B. (1994). Inftuence of anomalous contents of heavy metals on litter ant oil fungi in a mine area (S.w. Sardinia)-preliminary ree. Ts. *Giornale Botanico Italiano*, *128*(1), 404. Scopus. <https://doi.org/10.1080/11263509409437222>

Aphids (Aphis fabae OR Aphis gossypii OR Aphis nerii OR Aphis pomi OR Aphis rumicis OR Aphis varians OR Rhopalosiphum padi OR Myzus persicae OR Schizaphis graminum OR Toxoptera citricida):

Abbas, M. A., Al Husani, A. H., & Mohammadali, M. T. (2023). Evaluation of the Efficacy of Some Alcoholic Plant Extracts and the Biocide Naturalis-L Against Aphis fabae Scopoli (Homoptera: Aphididae) Under Laboratory Conditions. *Pakistan Journal of Scientific and Industrial Research Series B: Biological Sciences*, *66 B*(3), 215–219. Scopus.

Abd El-Salam, A. M. E., Salem, S. A., & El-Kholy, M. Y. (2012). Efficiency of Nimbecidine and certain entomopathogenic fungi formulations against bean aphids, Aphis craccivora in broad bean field. *Archives of Phytopathology and Plant Protection*, *45*(19), 2272–2277. Scopus. <https://doi.org/10.1080/03235408.2012.726144>

Abdelaziz, O., Senoussi, M. M., Oufroukh, A., Birgücü, A. K., Karaca, I., Kouadri, F., Naima, B., & Bensegueni, A. (2018). Pathogenicity of three entomopathogenic fungi, to the aphid species, Metopolophium dirhodum (Walker) (Hemiptera: Aphididae), and their Alkaline protease activities. *EGYPTIAN JOURNAL OF BIOLOGICAL PEST CONTROL*, *28*. <https://doi.org/10.1186/s41938-018-0030-7>

Abdel-Baky, N. F., & Abdel-Salam, A. H. (2003). Natural incidence of Cladosporium spp. As a bio-control agent against whiteflies and aphids in Egypt. *JOURNAL OF APPLIED ENTOMOLOGY*, *127*(4), 228–235. <https://doi.org/10.1046/j.1439-0418.2003.00662.x>

Abd-Elnabi, A. D., El-sawy, E. A. F., & El-Adawy, E. M. (2024). Insecticidal effects of the fast pyrolysis bio-oil against Spodoptera littoralis and Aphis gossypii insect pests. *JOURNAL OF ASIA-PACIFIC ENTOMOLOGY*, *27*(2). <https://doi.org/10.1016/j.aspen.2024.102237>

Abdel-Raheem, M. A., Reyad, N. F., Abdel-Rahman, I. E., & Al-Shuraym, L. A. (2016). Evaluation of some isolates of entomopathogenic fungi on some insect pests infesting potato crop in Egypt. *International Journal of ChemTech Research*, *9*(8), 479–485. Scopus.

Abe, M., Imai, T., Ishii, N., & Usui, M. (2006). Synthesis of quinolactacide via an acyl migration reaction and dehydrogenation with manganese dioxide, and its insecticidal activities. *BIOSCIENCE BIOTECHNOLOGY AND BIOCHEMISTRY*, *70*(1), 303–306. <https://doi.org/10.1271/bbb.70.303>

Abe, M., Imai, T., Ishii, N., Usui, M., Okuda, T., & Oki, T. (2005). Quinolactacide, a new quinolone insecticide from Penicillium citrinum Thom F 1539. *BIOSCIENCE BIOTECHNOLOGY AND BIOCHEMISTRY*, *69*(6), 1202–1205. <https://doi.org/10.1271/bbb.69.1202>

Abe, M., Imai, T., Ishii, N., Usui, M., Okuda, T., & Oki, T. (2007). Isolation of an insecticidal compound oxalicine B from Penicillium sp TAMA 71 and confirmation of its chemical structure by X-ray crystallographic analysis. *JOURNAL OF PESTICIDE SCIENCE*, *32*(2), 124–127. <https://doi.org/10.1584/jpestics.G06-36>

Acharya, S. N., Huang, H. C., Carcamo, H. A., Basu, S. K., Entz, T., Erickson, S., & Friebel, D. (2008). Do cultivar and burning affect forage yield and incidence of verticillium wilt or insect pests in alfalfa stands? *AGRONOMY JOURNAL*, *100*(3), 742–747. <https://doi.org/10.2134/agronj2007.0182>

Addisu, S., Fininsa, C., Bekeko, Z., Mohammad, A., Kumar, A., & Fikre, A. (2023). Distribution of Chickpea (Cicer arietinum L.) Ascochyta blight (Didymella rabiei) and analyses of factors affecting disease epidemics in Central Ethiopia. *European Journal of Plant Pathology*, *166*(4), 425–444.

Afanasenko, O. S., Khiutti, A., Mironenko, N., & Lashina, N. M. (2022). Transmission of potato spindle tuber viroid between Phytophthora infestans and host plants. *VAVILOVSKII ZHURNAL GENETIKI I SELEKTSII*, *26*(3), 272–280.

Afkhami, M. E., & Rudgers, J. A. (2009). Endophyte-mediated resistance to herbivores depends on herbivore identity in the wild grass festuca subverticillata. *Environmental Entomology*, *38*(4), 1086–1095. Scopus. <https://doi.org/10.1603/022.038.0416>

Ahmad, S., Veyrat, N., Gordon-Weeks, R., Zhang, Y., Martin, J., Smart, L., Glauser, G., Erb, M., Flors, V., Frey, M., & Ton, J. (2011). Benzoxazinoid metabolites regulate innate immunity against aphids and fungi in maize. *Plant Physiology*, *157*(1), 317–327. Scopus. <https://doi.org/10.1104/pp.111.180224>

Åhman, I., & Wilson, F. (2008). Symptoms of pests, rust and other disorders on leaves of willow fertilised with wastewater, urine or sewage sludge. *BIOMASS & BIOENERGY*, *32*(11), 1001–1008. <https://doi.org/10.1016/j.biombioe.2008.01.023>

Ahmed, M., Sajid, A. R., Javeed, A., Aslam, M., Ahsan, T., Hussain, D., Mateen, A., Li, X., Qin, P., & Ji, M. (2022). Antioxidant, antifungal, and aphicidal activity of the triterpenoids spinasterol and 22,23-dihydrospinasterol from leaves of Citrullus colocynthis L. *Scientific Reports*, *12*(1). Scopus. <https://doi.org/10.1038/s41598-022-08999-z>

Ahn, Y. J., Lee, H. S., Oh, H. S., Kim, H. T., & Lee, Y. H. (2005). Antifungal activity and mode of action of Galla rhois-derived phenolics against phytopathogenic fungi. *PESTICIDE BIOCHEMISTRY AND PHYSIOLOGY*, *81*(2), 105–112. <https://doi.org/10.1016/j.pestbp.2004.10.003>

Ahuja, D. B., Ahuja, U. R., Kalyan, R. K., Sharma, Y. K., Dhandapani, A., & Meena, P. C. (2009). Evaluation of different management strategies for Lipaphis pseudobrassicae (Davis) on Brassica juncea. *INTERNATIONAL JOURNAL OF PEST MANAGEMENT*, *55*(1), 11–18. <https://doi.org/10.1080/09670870802422580>

Ahuja, D. B., Ahuja, U. R., Singh, S. K., & Singh, N. (2015). Comparison of Integrated Pest Management approaches and conventional (non-IPM) practices in late-winter-season cauliflower in Northern India. *CROP PROTECTION*, *78*, 232–238. <https://doi.org/10.1016/j.cropro.2015.08.007>

Ainsworth, G. C., Oyler, E., & Read, W. H. (1938). Observations on the spotting of tomato fruits by Botrytis cinerea pers. *ANNALS OF APPLIED BIOLOGY*, *25*(2), 308–321. <https://doi.org/10.1111/j.1744-7348.1938.tb02336.x>

Aiuchi, D., Baba, Y., Inami, K., Shinya, R., Tani, M., Kuramochi, K., Horie, S., & Koike, M. (2007). Screening of Verticillium lecanii (Lecanicillium spp.) hybrid strains based on evaluation of pathogenicity against cotton aphid and greenhouse whitefly, and viability on the leaf surface. *JAPANESE JOURNAL OF APPLIED ENTOMOLOGY AND ZOOLOGY*, *51*(3), 205–212. <https://doi.org/10.1303/jjaez.2007.205>

Ajayi, O., & Dewar, A. M. (1983). THE EFFECTS OF BARLEY YELLOW DWARF VIRUS, APHIDS AND HONEYDEW ON CLADOSPORIUM INFECTION OF WINTER-WHEAT AND BARLEY. *ANNALS OF APPLIED BIOLOGY*, *102*(1), 57–65. <https://doi.org/10.1111/j.1744-7348.1983.tb02665.x>

Ajlan, A. M., & Potter, D. A. (1991). DOES IMMUNIZATION OF CUCUMBER AGAINST ANTHRACNOSE BY COLLETOTRICHUM-LAGENARIUM AFFECT HOST SUITABILITY FOR ARTHROPODS. *ENTOMOLOGIA EXPERIMENTALIS ET APPLICATA*, *58*(1), 83–91. <https://doi.org/10.1111/j.1570-7458.1991.tb01455.x>

Akbari, S., Mirfakhraie, S., Aramideh, S., & Safaralizadeh, M. H. (2020). Effect of fungal isolates and imidacloprid on cabbage aphid Brevicoryne brassicae and its parasitoid Diaeretiella rapae. *ZEMDIRBYSTE-AGRICULTURE*, *107*(3), 255–262. <https://doi.org/10.13080/z-a.2020.107.033>

Akello, J., & Sikora, R. (2012). Systemic acropedal influence of endophyte seed treatment on Acyrthosiphon pisum and Aphis fabae offspring development and reproductive fitness. *BIOLOGICAL CONTROL*, *61*(3), 215–221. <https://doi.org/10.1016/j.biocontrol.2012.02.007>

Akrich, A., Righi, K., Righi, F. A., & Elouissi, A. (2023). Characterization of a new isolate of Beauveria bassiana in Algeria and evaluation of its pathogenicity against the cowpea aphid (Aphis craccivora Koch). *EGYPTIAN JOURNAL OF BIOLOGICAL PEST CONTROL*, *33*(1). <https://doi.org/10.1186/s41938-023-00723-x>

Al-alawi, M. S., & Obeidat, M. (2013). Selection of Beauveria bassiana (Balsamo) vuillemin isolates for management of myzus persicae (sultzar) (HOM.: Aphidae) based on virulence and growth related characteristics. *American Journal of Agricultural and Biological Science*, *9*(1), 94–100. Scopus. <https://doi.org/10.3844/ajabssp.2014.91.100>

Alam, S. T., Sarowar, S., Mondal, H. A., Makandar, R., Chowdhury, Z., Louis, J., & Shah, J. (2022). Opposing effects of MYZUS PERSICAE-INDUCED LIPASE 1 and jasmonic acid influence the outcome of Arabidopsis thaliana–Fusarium graminearum interaction. *Molecular Plant Pathology*, *23*(8), 1141–1153. Scopus. <https://doi.org/10.1111/mpp.13216>

Alavo, T. B. C., Sermann, H., & Bochow, H. (2002a). Biocontrol of aphids using verticillium lecanii in Greenhouse: Factor reducing the effectiveness of the entomopathogenic fungus. *Archives of Phytopathology and Plant Protection*, *34*(6), 407–424. Scopus. <https://doi.org/10.1080/713710567>

Alavo, T. B. C., Sermann, H., & Bochow, H. (2002b). Virulence of strains of the entomopathogenic fungus verticillium lecanii to aphids: Strain improvement. *Archives of Phytopathology and Plant Protection*, *34*(6), 379–398. Scopus. <https://doi.org/10.1080/716061669>

Albehadli, K., & Mohmed, A. S. (2022). *Laboratory Evaluation of the Aphidicidal Activity of Some Entomopathogenic Fungal Culture Filtrate against Aphid Schizaphis Graminum* (L. A. H. M. Jawad, F. F. K. Hussain, A. F. Almurshedi, T. H. Ali, H. K. Al-Mussawi, S. K. Kadhem, & S. Majeed, Eds.; Vol. 2398). American Institute of Physics Inc.; Scopus. <https://doi.org/10.1063/5.0093653>

Alderman, S. C. (2013). Survival, Germination, and Growth of Epichloe typhina and Significance of Leaf Wounds and Insects in Infection of Orchardgrass. *PLANT DISEASE*, *97*(3), 323–328. <https://doi.org/10.1094/PDIS-01-12-0075-RE>

Ali, K., & Swart, W. (2007). Effect of Mixed Cropping of PisLm sativum L. on Acyrthosiphon pisum (HarrisJ Infestation and Ascochyta Blight Infection in Ethiopia. *Pest Managment Journal of Ethiopia*, *11*, 69–79.

Ali, S. A. M., Saleh, A. A. A., & Saleh, F. M. (2020a). Biocontrol of certain piercing sucking pests infesting cucumber plants in Egypt. *Plant Archives*, *20*, 3347–3357. Scopus.

Ali, S. A. M., Saleh, A. A. A., & Saleh, F. M. (2020b). Bioefficacy of plant extracts and entomopathogenic fungi (trichoderma album) in controling myzus persicae and bemisia tabaci. *Plant Archives*, *20*, 1450–1459. Scopus.

Ali, S., Farooqi, M. A., Sajjad, A., Ullah, M. I., Qureshi, A. K., Siddique, B., Waheed, W., Sarfraz, M., & Asghar, A. (2018). Compatibility of entomopathogenic fungi and botanical extracts against the wheat aphid, Sitobion avenae (Fab.) (Hemiptera: Aphididae). *EGYPTIAN JOURNAL OF BIOLOGICAL PEST CONTROL*, *28*. <https://doi.org/10.1186/s41938-018-0101-9>

Ali, S., Sajjad, A., Shakeel, Q., Farooqi, M. A., Aqueel, M. A., Tariq, K., Ullah, M. I., Iqbal, A., Jamal, A., Saeed, M. F., & Manachini, B. (2022). Influence of Bacterial Secondary Symbionts in Sitobion avenae on Its Survival Fitness against Entomopathogenic Fungi, Beauveria bassiana and Metarhizium brunneum. *INSECTS*, *13*(11). <https://doi.org/10.3390/insects13111037>

Alins, G., Lordan, J., Rodríguez-Gasol, N., Arnó, J., & Peñalver-Cruz, A. (2023). Earwig Releases Provide Accumulative Biological Control of the Woolly Apple Aphid over the Years. *INSECTS*, *14*(11). <https://doi.org/10.3390/insects14110890>

Al-Khawaldeh, M. M., Araj, S. E., Alananbeh, K. M., & Al Antary, T. M. (2020). WHEAT CULTIVABLE FUNGAL ENDOPHYTES IN JORDAN. *FRESENIUS ENVIRONMENTAL BULLETIN*, *29*(2), 1229–1240.

Allegrucci, N., Velazquez, M. S., Russo, M. L., Vianna, M. F., Abarca, C., & Scorsetti, A. C. (2020). Establishment of the entomopathogenic fungus Beauveria bassiana as an endophyte in Capsicum annuum and its effects on the aphid pest Myzus persicae (Nom optera: Aphididae). *REVISTA DE BIOLOGIA TROPICAL*, *68*(4), 1084–1094.

Allen, D. J., Emechebe, A. M., & Ndimande, B. (1981). IDENTIFICATION OF RESISTANCE IN COWPEA TO DISEASES OF THE AFRICAN SAVANNAS. *TROPICAL AGRICULTURE*, *58*(3), 267–274.

Al-Naemi, F., & Hatcher, P. E. (2013). Contrasting effects of necrotrophic and biotrophic plant pathogens on the aphid Aphis fabae. *ENTOMOLOGIA EXPERIMENTALIS ET APPLICATA*, *148*(3), 234–245. <https://doi.org/10.1111/eea.12091>

Alnajjar, A., Basedow, T., & Schulz, F. A. (1989). EFFECTS OF GRADUALLY DIFFERING INTENSITIES OF GROWING WHEAT ON PESTS AND DISEASES, YIELDS AND ECONOMY .1. PHYTOMEDICAL ASPECTS. *ZEITSCHRIFT FUR PFLANZENKRANKHEITEN UND PFLANZENSCHUTZ-JOURNAL OF PLANT DISEASES AND PROTECTION*, *96*(6), 561–584.

Alyaseri, I. I., Hamdan, F. Q., Dagher, G. M., & Hasan, N. A. (2020). Effectiveness of Conocarpus lancifolius Extract against Insects and Pathogenic Fungi. *Indian Journal of Ecology*, *47*, 204–210. Scopus.

Ambethgar, V. (2018). *Strategic Approaches for Applications of Entomopathogenic Fungi to Counter Insecticide Resistance in Agriculturally Important Insect Pests* (WOS:000529445300013). <https://doi.org/10.1007/978-981-13-0393-7_13>

Amnuaykanjanasin, A., Jirakkakul, J., Panyasiri, C., Panyarakkit, P., Nounurai, P., Chantasingh, D., Eurwilaichitr, L., Cheevadhanarak, S., & Tanticharoen, M. (2013a). Erratum to: Infection and colonization of tissues of the aphid Myzus persicae and cassava mealybug Phenacoccus manihoti by the fungus Beauveria bassiana (BioControl, 10.1007/s10526-012-9499-2). *BioControl*, *58*(3), 393–396. Scopus. <https://doi.org/10.1007/s10526-013-9504-4>

Amnuaykanjanasin, A., Jirakkakul, J., Panyasiri, C., Panyarakkit, P., Nounurai, P., Chantasingh, D., Eurwilaichitr, L., Cheevadhanarak, S., & Tanticharoen, M. (2013b). Infection and colonization of tissues of the aphid Myzus persicae and cassava mealybug Phenacoccus manihoti by the fungus Beauveria bassiana. *BioControl*, *58*(3), 379–391. Scopus. <https://doi.org/10.1007/s10526-012-9499-2>

Amutha, M. (2021). In planta colonisation of Beauveria bassiana in cotton plant and its effect against insect pests. *Journal of Biological Control*, *35*(3), 137–145. Scopus. <https://doi.org/10.18311/jbc/2021/28489>

Andersen, M., Magan, N., Mead, A., & Chandler, D. (2006). Development of a population-based threshold model of conidial germination for analysing the effects of physiological manipulation on the stress tolerance and infectivity of insect pathogenic fungi. *ENVIRONMENTAL MICROBIOLOGY*, *8*(9), 1625–1634. <https://doi.org/10.1111/j.1462-2920.2006.01055.x>

Anwar, W., Amin, H., Khan, H. A. A., Akhter, A., Bashir, U., Anjum, T., Kalsoom, R., Javed, M. A., & Zohaib, K. A. (2023). Chitinase of Trichoderma longibrachiatum for control of Aphis gossypii in cotton plants. *SCIENTIFIC REPORTS*, *13*(1). <https://doi.org/10.1038/s41598-023-39965-y>

Anwar, W., Nawaz, K., Javed, M. A., Akhter, A., Shahid, A. A., Haider, M. S., Rehman, M. Z. U., & Ali, S. (2021). Characterization of fungal flora associated with sternorrhyncha insects of cotton plants. *Biologia*, *76*(2), 533–547. Scopus. <https://doi.org/10.2478/s11756-020-00549-0>

Aponte, M., & Blaiotta, G. (2016). Potential Role of Yeast Strains Isolated from Grapes in the Production of Taurasi DOCG. *FRONTIERS IN MICROBIOLOGY*, *7*. <https://doi.org/10.3389/fmicb.2016.00809>

Aqueel, M. A., & Leather, S. R. (2013). Virulence of Verticillium lecanii (Z.) against cereal aphids; does timing of infection affect the performance of parasitoids and predators? *PEST MANAGEMENT SCIENCE*, *69*(4), 493–498. <https://doi.org/10.1002/ps.3398>

Arinanto, L. S., Hoffmann, A. A., Ross, P. A., & Gu, X. Y. (2024). Hormetic effect induced by Beauveria bassiana in Myzus persicae. *PEST MANAGEMENT SCIENCE*, *80*(8), 3726–3733. <https://doi.org/10.1002/ps.8075>

Arnold, A. J., Cayley, G. R., Dunne, Y., Etheridge, P., Griffiths, D. C., Jenkyn, J. F., Phillips, F. T., Pye, B. J., Scott, G. C., & Woodcock, C. M. (1984). BIOLOGICAL EFFECTIVENESS OF ELECTROSTATICALLY CHARGED ROTARY ATOMIZERS .2. TRIALS WITH CEREALS, 1982. *ANNALS OF APPLIED BIOLOGY*, *105*(2), 361–367. <https://doi.org/10.1111/j.1744-7348.1984.tb03060.x>

Arnold, A. J., Cayley, G. R., Dunne, Y., Etheridge, P., Griffiths, D. C., Phillips, F. T., Pye, B. J., Scott, G. C., & Vojvodic, P. R. (1984). BIOLOGICAL EFFECTIVENESS OF ELECTROSTATICALLY CHARGED ROTARY ATOMIZERS .1. TRIALS ON FIELD BEANS AND BARLEY, 1981. *ANNALS OF APPLIED BIOLOGY*, *105*(2), 353–359. <https://doi.org/10.1111/j.1744-7348.1984.tb03059.x>

Arnyas, E., Bereczki, J., Tóth, A., Varga, K., Pecsenye, K., Tartally, A., Kövics, G., Karsa, D., & Varga, Z. (2009). Oviposition preferences of Maculinea alcon as influenced by aphid (Aphis gentianae) and fungal (Puccinia gentianae) infestation of larval host plants. *ECOLOGICAL ENTOMOLOGY*, *34*(1), 90–97. <https://doi.org/10.1111/j.1365-2311.2008.01048.x>

Arora, R. K. (2009). *Late Blight: An Increasing Threat to Seed Potato Production in the North-Western Plains of India* (WOS:000305705300022). *834*, 201–204. <https://doi.org/10.17660/ActaHortic.2009.834.22>

Asalf, B., Ficke, A., & Klingen, I. (2021a). Interaction between the bird cherry-oat aphid (Rhopalosiphum padi) and stagonospora nodorum blotch (parastagonospora nodorum) on wheat. *Insects*, *12*(1), 1–13. Scopus. <https://doi.org/10.3390/insects12010035>

Asalf, B., Ficke, A., & Klingen, I. (2021b). Interaction between the Bird Cherry-Oat Aphid (Rhopalosiphum padi) and Stagonospora Nodorum Blotch (Parastagonospora nodorum) on Wheat. *Insects*, *12*(1). <https://doi.org/10.3390/insects12010035>

Asante, S. K. (1995). FUNCTIONAL-RESPONSES OF THE EUROPEAN EARWIG AND 2 SPECIES OF COCCINELLIDS TO DENSITIES OF ERIOSOMA-LANIGERUM (HAUSMANN) (HEMIPTERA, APHIDIDAE). *JOURNAL OF THE AUSTRALIAN ENTOMOLOGICAL SOCIETY*, *34*, 105–109.

Ashouri, A., Arzanian, N., Askary, H., & Rasoulian, G. R. (2004). Pathogenicity of the fungus, Verticillium lecanii, to the green peach aphid, Myzus persicae (Hom.: Aphididae). *Communications in Agricultural and Applied Biological Sciences*, *69*(3), 205–209. Scopus.

Ashraf, M., Hussain, D., Hussain, S., Din, N., Ali, Q., Jahan, M. S., Yasin, M., & Farooq, M. (2024). Potential of two entomopathogenic fungi, Beauveria bassiana and metarhizium anisopliae as biocontrol agents of Rhpalosiphum padi and Shizaphis graminum, (Homoptera: Aphididae) in laboratory and field. *INTERNATIONAL JOURNAL OF TROPICAL INSECT SCIENCE*, *44*(1), 307–312. <https://doi.org/10.1007/s42690-024-01160-5>

Askary, H., Benhamou, N., & Brodeur, J. (1999). Ultrastructural and cytochemical characterization of aphid invasion by the hyphomycete Verticillium lecanii. *JOURNAL OF INVERTEBRATE PATHOLOGY*, *74*(1), 1–13. <https://doi.org/10.1006/jipa.1999.4857>

Askary, H., Carrière, Y., Bélanger, R. R., & Brodeur, J. (1998). Pathogenicity of the fungus Verticillium lecanii to aphids and powdery mildew. *Biocontrol Science and Technology*, *8*(1), 23–32. Scopus. <https://doi.org/10.1080/09583159830405>

Åsman, K. (2007). Aphid infestation in field grown lettuce and biological control with entomopathogenic fungi (deuteromycotina: Hyphomycetes). *Biological Agriculture and Horticulture*, *25*(2), 153–173. Scopus. <https://doi.org/10.1080/01448765.2007.9755043>

Atrchian, H., Mahdian, K., & Izadi, H. (2024). Compatibility of the Entomopathogenic Fungus Metarhizium anisopliae (Ascomycota: Hypocreales) and the Predatory Coccinellid Menochilus sexmaculatus (Col.: Coccinellidae) for Controlling Aphis gossypii (Hem.: Aphididae). *NEOTROPICAL ENTOMOLOGY*, *53*(4), 907–916. <https://doi.org/10.1007/s13744-024-01163-4>

Babikova, Z., Gilbert, L., Bruce, T., Dewhirst, S. Y., Pickett, J. A., & Johnson, D. (2014). Arbuscular mycorrhizal fungi and aphids interact by changing host plant quality and volatile emission. *Functional Ecology*, *28*(2), 375–385. Scopus. <https://doi.org/10.1111/1365-2435.12181>

Bahar, M. H., Backhouse, D., Gregg, P. C., & Mensah, R. (2011). Efficacy of a Cladosporium sp fungus against Helicoverpa armigera (Lepidoptera: Noctuidae), other insect pests and beneficial insects of cotton. *BIOCONTROL SCIENCE AND TECHNOLOGY*, *21*(12), 1387–1397. <https://doi.org/10.1080/09583157.2011.622036>

Bajorat, B., Blumendeller, C., & Schonbeck, F. (1995). Influence of direct and indirect damages to root systems on plant efficiency. *ZEITSCHRIFT FUR PFLANZENKRANKHEITEN UND PFLANZENSCHUTZ-JOURNAL OF PLANT DISEASES AND PROTECTION*, *102*(6), 561–573.

Baki, D., & Erler, F. (2024). Evaluation of indigenous isolates of Beuveria bassiana (Balsamo) Vuillemin (Deuteromycotina: Hyphomycetes) against the cotton aphid Aphis gossypii Glover. *Journal of Plant Diseases and Protection*. Scopus. <https://doi.org/10.1007/s41348-024-00952-8>

Balfour, A., & Khan, A. (2012). Effects of Verticillium lecanii (Zimm.) Viegas on Toxoptera citricida Kirkaldy (Homoptera: Aphididae) and its Parasitoid Lysiphlebus testaceipes Cresson (Hymenoptera: Braconidae). *PLANT PROTECTION SCIENCE*, *48*(3), 123–130. <https://doi.org/10.17221/59/2011-PPS>

Bálint, J., Szabó, A. K., Tófalvi, B., Puia, C., & Balog, A. (2016). Comparing disease resistance of local and international plum cultivars (Prunus domestica) from Eastern Transylvania, Romania. *JOURNAL OF PLANT DISEASES AND PROTECTION*, *123*(6), 317–320. <https://doi.org/10.1007/s41348-016-0048-6>

Baltenberger, D. E., Ohm, H. W., & Foster, J. E. (1988). RECURRENT SELECTION FOR TOLERANCE TO BARLEY YELLOW DWARF VIRUS IN OAT. *CROP SCIENCE*, *28*(3), 477–480. <https://doi.org/10.2135/cropsci1988.0011183X002800030009x>

Banttari, E. E., & Wilcoxson, R. D. (1964). EFFECT OF PEA APHIDS ON SPRING BLACK STEM OF ALFALFA. *PHYTOPATHOLOGY*, *54*(11), 1415-+.

Barker, S. J., Edmonds-Tibbett, T. L., Forsyth, L. M., Klingler, J. P., Toussaint, J. P., Smith, F. A., & Smith, S. E. (2005). Root infection of the reduced mycorrhizal colonization (rmc) mutant of tomato reveals genetic interaction between symbiosis and parasitism. *PHYSIOLOGICAL AND MOLECULAR PLANT PATHOLOGY*, *67*(6), 277–283. <https://doi.org/10.1016/j.pmpp.2006.03.003>

Barnard, A. J., McEwen, J., Hornby, D., & Beane, J. (1989). EFFECTS OF ALDICARB, BENOMYL, DAZOMET, PERMETHRIN, PIRIMICARB, PHORATE AND NITROGEN-FERTILIZER ON MAIZE (ZEA-MAYS) GROWN FOR 14 CONSECUTIVE YEARS ON THE SAME SITE. *JOURNAL OF AGRICULTURAL SCIENCE*, *112*, 339–349. <https://doi.org/10.1017/S0021859600085798>

Barta, M. (2009). Entomophthoralean fungi associated with aphid s in woody plants in the arboretum mlyňany SAS. *Folia Oecologica*, *36*(1), 1–7. Scopus.

Barta, M., & Cagáň, L. (2003). Entomophthoralean fungi associated with the common nettle aphid (Microlophium carnosum Buckton) and the potential role of nettle patches as reservoirs for the pathogens in landscape. *Anzeiger Fur Schadlingskunde*, *76*(1), 6–13. Scopus. <https://doi.org/10.1046/j.1439-0280.2003.03004.x>

Basit, A., Farhan, M., Abbas, M., Wang, Y., Zhao, D. G., Mridha, A. U., Al-tawaha, A. R. M. S., Bashir, M. A., Arif, M., Ahmed, S., Alajmi, R. A., Metwally, D. M., & El-Khadragy, M. (2021). Do microbial protein elicitors PeaT1 obtained from Alternaria tenuissima and PeBL1 from Brevibacillus laterosporus enhance defense response against tomato aphid (Myzus persicae)? *Saudi Journal of Biological Sciences*, *28*(6), 3242–3248. Scopus. <https://doi.org/10.1016/j.sjbs.2021.02.063>

Basit, A., Hanan, A., Nazir, T., Majeed, M. Z., & Qiu, D. W. (2019). Molecular and Functional Characterization of Elicitor PeBC1 Extracted from Botrytis cinerea Involved in the Induction of Resistance against Green Peach Aphid (Myzus persicae) in Common Beans (Phaseolus vulgaris L.). *INSECTS*, *10*(2). <https://doi.org/10.3390/insects10020035>

Basit, A., Humza, M., Majeed, M. Z., Shakeel, M., Idrees, A., Hu, C. X., Gui, S. H., & Liu, T. X. (2024). Systemic resistance induced in tomato plants by Beauveria bassiana-derived proteins against tomato yellow leaf curl virus and aphid Myzus persicae. *PEST MANAGEMENT SCIENCE*, *80*(4), 1821–1830. <https://doi.org/10.1002/ps.7906>

Bastias, D. A., Bustos, L. B., Jauregui, R., Barrera, A., Acuna-Rodriguez, I. S., Molina-Montenegro, M. A., & Gundel, P. E. (2022). Epichloe Fungal Endophytes Influence Seed-Associated Bacterial Communities. *FRONTIERS IN MICROBIOLOGY*, *12*. <https://doi.org/10.3389/fmicb.2021.795354>

Bastías, D. A., Martínez-Ghersa, M. A., Newman, J. A., Card, S. D., Mace, W. J., & Gundel, P. E. (2018). The plant hormone salicylic acid interacts with the mechanism of anti-herbivory conferred by fungal endophytes in grasses. *PLANT CELL AND ENVIRONMENT*, *41*(2), 395–405. <https://doi.org/10.1111/pce.13102>

Bastías, D. A., Martínez-Ghersa, M. A., Newman, J. A., Card, S. D., Mace, W. J., & Gundel, P. E. (2019). Sipha maydis sensitivity to defences of Lolium multiflorum and its endophytic fungus Epichloe occultans. *PEERJ*, *7*. <https://doi.org/10.7717/peerj.8257>

Bastias, D. A., Ueno, A. C., Assefh, C. R. M., Alvarez, A. E., Young, C. A., & Gundel, P. E. (2017). Metabolism or behavior: Explaining the performance of aphids on alkaloid-producing fungal endophytes in annual ryegrass (Lolium multiflorum). *OECOLOGIA*, *185*(2), 245–256. <https://doi.org/10.1007/s00442-017-3940-2>

Bataeva, Y. V., Grigoryan, L. N., Bogun, A. G., Kislichkina, A. A., Platonov, M. E., Kurashov, E. A., Krylova, J. V., Fedorenko, A. G., & Andreeva, M. P. (2023). Biological Activity and Composition of Metabolites of Potential Agricultural Application from Streptomyces carpaticus K-11 RCAM04697 (SCPM-O-B-9993). *Microbiology (Russian Federation)*, *92*(3), 459–467. Scopus. <https://doi.org/10.1134/S0026261723600155>

Battaglia, D., Bossi, S., Cascone, P., Digilio, M. C., Prieto, J. D., Fanti, P., Guerrieri, E., Iodice, L., Lingua, G., Lorito, M., Maffei, M. E., Massa, N., Ruocco, M., Sasso, R., & Trotta, V. (2013). Tomato Below Ground-Above Ground Interactions: Trichoderma longibrachiatum Affects the Performance of Macrosiphum euphorbiae and Its Natural Antagonists. *MOLECULAR PLANT-MICROBE INTERACTIONS*, *26*(10), 1249–1256. <https://doi.org/10.1094/MPMI-02-13-0059-R>

Baudracco-Arnas, S., & Pitrat, M. (1996). A genetic map of melon (Cucumis melo L.) with RFLP, RAPD, isozyme, disease resistance and morphological markers. *Theoretical and Applied Genetics*, *93*(1–2), 57–64. Scopus. <https://doi.org/10.1007/BF00225727>

Baverstock, J., Clark, S. J., Alderson, P. G., & Pell, J. K. (2009). Intraguild interactions between the entomopathogenic fungus Pandora neoaphidis and an aphid predator and parasitoid at the population scale. *Journal of Invertebrate Pathology*, *102*(2), 167–172. Scopus. <https://doi.org/10.1016/j.jip.2009.07.014>

Baverstock, J., Elliot, S. L., Alderson, P. G., & Pell, J. K. (2005). Response of the entomopathogenic fungus Pandora neoaphidis to aphid-induced plant volatiles. *Journal of Invertebrate Pathology*, *89*(2), 157–164. Scopus. <https://doi.org/10.1016/j.jip.2005.05.006>

Baverstock, J., Roy, H. E., Clark, S. J., Alderson, P. G., & Pell, J. K. (2006). Effect of fungal infection on the reproductive potential of aphids and their progeny. *JOURNAL OF INVERTEBRATE PATHOLOGY*, *91*(2), 136–139. <https://doi.org/10.1016/j.jip.2005.11.005>

Bayhan, S. Ö., Ulusoy, M. R., & Bayhan, E. (2006). Aphids and their predators in Malatya region and around, Turkey. *Journal of Biological Sciences*, *6*(5), 954–957. Scopus. <https://doi.org/10.3923/jbs.2006.954.957>

Bayissa, W., Ekesi, S., Mohamed, S. A., Kaaya, G. P., Wagacha, J. M., Hanna, R., & Maniania, N. K. (2016). Interactions among vegetable-infesting aphids, the fungal pathogen Metarhizium anisopliae (Ascomycota: Hypocreales) and the predatory coccinellid Cheilomenes lunata (Coleoptera: Coccinellidae). *BIOCONTROL SCIENCE AND TECHNOLOGY*, *26*(2), 274–290. <https://doi.org/10.1080/09583157.2015.1099148>

Bayissa, W., Ekesi, S., Mohamed, S. A., Kaaya, G. P., Wagacha, J. M., Hanna, R., & Maniania, N. K. (2017). Selection of fungal isolates for virulence against three aphid pest species of crucifers and okra. *JOURNAL OF PEST SCIENCE*, *90*(1), 355–368. <https://doi.org/10.1007/s10340-016-0781-4>

Baysal, F., & Çinar, A. (2007a). *Determination of phytopathological and entomological problems and using plant protection methods in organic tomato growing in turkey* (WOS:000245943000077). 459-+. <https://doi.org/10.17660/ActaHortic.2007.729.77>

Baysal, F., & Çinar, A. (2007b). *Determination of phytopathological and entomological problems and using plant protection methods in organic tomato growing in Turkey*. *729*, 459–462. Scopus. <https://doi.org/10.17660/ActaHortic.2007.729.77>

Beck, A., Haitz, F., Thier, I., Siems, K., Jakupovic, S., Rupp, S., & Zibek, S. (2021). Novel mannosylerythritol lipid biosurfactant structures from castor oil revealed by advanced structure analysis. *JOURNAL OF INDUSTRIAL MICROBIOLOGY & BIOTECHNOLOGY*, *48*(7–8). <https://doi.org/10.1093/jimb/kuab042>

Bedford, I. D., Kelly, A., Banks, G. K., Fuog, D., & Markham, P. G. (1998). The effect of Pymetrozine, a feeding inhibitor of Homoptera, in preventing transmission of cauliflower mosaic caulimovirus by the aphid species Myzus persicae (Sulzer). *Annals of Applied Biology*, *132*(3), 453–462. Scopus. <https://doi.org/10.1111/j.1744-7348.1998.tb05221.x>

Beers, E. H., Horton, D. R., & Miliczky, E. (2016). Pesticides used against Cydia pomonella disrupt biological control of secondary pests of apple. *BIOLOGICAL CONTROL*, *102*, 35–43. <https://doi.org/10.1016/j.biocontrol.2016.05.009>

Beers, E. H., Mills, N. J., Shearer, P. W., Horton, D. R., Milickzy, E. R., Amarasekare, K. G., & Gontijo, L. M. (2016). Nontarget effects of orchard pesticides on natural enemies: Lessons from the field and laboratory. *BIOLOGICAL CONTROL*, *102*, 44–52. <https://doi.org/10.1016/j.biocontrol.2016.04.010>

Bellesini Vigna, F., de Rensis, A. P. A. S., Rodrigues, L. R., Oliva, M. B., Rossi, M. M., & de Sene Pinto, A. (2024). Potential of entomopathogenic fungi to control insect pests and disease at lettuce and arugula crops. *Comunicata Scientiae*, *15*. Scopus. <https://doi.org/10.14295/cs.v15.3250>

Ben Saad, A. A., & Bishop, G. W. (1976). Effect of artificial honeydews on insect communities in potato fields. *Environmental Entomology*, *5*(6), 453–457. Scopus. <https://doi.org/10.1093/ee/5.3.453>

Bensaci, O. A., Daoud, H., Lombarkia, N., & Rouabah, K. (2015). Formulation of the endophytic fungus Cladosporium oxysporum Berk. & M.A. Curtis, isolated from Euphorbia bupleuroides subsp. Luteola, as a new biocontrol tool against the black bean aphid (Aphis fabae Scop.). *Journal of Plant Protection Research*, *55*(1), 80–87. Scopus. <https://doi.org/10.1515/jppr-2015-0011>

Bensaci, O. A., Rouabah, K., Aliat, T., Lombarkia, N., Plushikov, V. G., Kucher, D. E., Dokukin, P. A., Temirbekova, S. K., & Rebouh, N. Y. (2022). Biological Pests Management for Sustainable Agriculture: Understanding the Influence of Cladosporium-Bioformulated Endophytic Fungi Application to Control Myzus persicae (Sulzer, 1776) in Potato (Solanum tuberosum L.). *PLANTS-BASEL*, *11*(15). <https://doi.org/10.3390/plants11152055>

Berber, G., & Birgücü, A. K. (2022). Effects of Two Different Isolates of Entomopathogen Fungus, Beauveria bassiana (Balsamo) Vuillemin on Myzus persicae Sulzer (Hemiptera: Aphididae). *Tarim Bilimleri Dergisi*, *28*(1), 121–132. Scopus. <https://doi.org/10.15832/ankutbd.828767>

Berestetskiy, A. O., Gannibal, F. B., Minkovich, E. V., Osterman, I. A., Salimova, D. R., Sergiev, P. V., & Sokornova, S. V. (2018). Spectrum of Biological Activity of the Alternaria Fungi Isolated from the Phyllosphere of Herbaceous Plants. *MICROBIOLOGY*, *87*(6), 806–816. <https://doi.org/10.1134/S0026261718060036>

Berestetskiy, A. O., Grigoryeva, E. N., Petrova, M. O., & Stepanycheva, E. A. (2018). Insecticidal and phytotoxic activity of extracts from cultures of some cereal pathogens. *Mikologiya I Fitopatologiya*, *52*(6), 408–419. Scopus. <https://doi.org/10.1134/S0026364818060016>

Berestetskiy, A. O., Inyusheva, V. V., Petrova, M. O., Sokornova, S., & Stepanycheva, E. A. (2019). Insecticidal, acaricidal, and cytotoxic activity of extracts from some phyllosphere fungi and soil hypocrealean micromycetes. *Mikologiya I Fitopatologiya*, *53*(1), 17–25. Scopus. <https://doi.org/10.1134/S0026364819010033>

Bhaskar, P. B., Ahuja, I., Janeja, H. S., & Banga, S. S. (2002). Intergeneric hybridization between Erucastrum canariense and Brassica rapa.: Genetic relatedness between Ec and A genomes. *THEORETICAL AND APPLIED GENETICS*, *105*(5), 754–758. <https://doi.org/10.1007/s00122-002-0915-3>

Bhatnagar, S., Kumari, R., & Kaur, I. (2024). Seaweed and a biocontrol agent and their effects on the growth and production of Brassica juncea: A sustainable approach. *WORLD JOURNAL OF MICROBIOLOGY & BIOTECHNOLOGY*, *40*(1). <https://doi.org/10.1007/s11274-023-03835-6>

Bhuiyan, M. A. B., Abdullah, H. M., Arman, S. E., Rahman, S. S., & Al Mahmud, K. (2023). BananaSqueezeNet: A very fast, lightweight convolutional neural network for the diagnosis of three prominent banana leaf diseases. *SMART AGRICULTURAL TECHNOLOGY*, *4*. <https://doi.org/10.1016/j.atech.2023.100214>

Biango-Daniels, M. N., Wang, T. W., & Hodge, K. T. (2018). Draft Genome Sequence of the Patulin-Producing Fungus Paecilomyces niveus Strain CO7. *GENOME ANNOUNCEMENTS*, *6*(25). <https://doi.org/10.1128/genomeA.00556-18>

Binns, M. R., Macfadyen, S., & Umina, P. A. (2022). The dual role of earwigs (Dermaptera) in winter grain crops in Australia. *JOURNAL OF APPLIED ENTOMOLOGY*, *146*(3), 272–283. <https://doi.org/10.1111/jen.12959>

Birch, A. N. E., Tithecott, M. T., & Bisby, F. A. (1985). Vicia johannis and wild relatives of the faba bean: A taxonomic study. *Economic Botany*, *39*(2), 177–190. Scopus. <https://doi.org/10.1007/BF02907843>

Biryol, S., Demirbag, Z., Erdogan, P., & Demir, I. (2022). Development of Beauveria bassiana (Ascomycota: Hypocreales) as a mycoinsecticide to control green peach aphid, Myzus persicae (Homoptera: Aphididae) and investigation of its biocontrol potential. *JOURNAL OF ASIA-PACIFIC ENTOMOLOGY*, *25*(1). <https://doi.org/10.1016/j.aspen.2022.101878>

Bischoff, R., Pokharel, P., Miedtke, P., Piepho, H. P., & Petschenka, G. (2024). Environmental complexity and predator density mediate a stable earwig-woolly apple aphid interaction. *BASIC AND APPLIED ECOLOGY*, *74*, 108–114. <https://doi.org/10.1016/j.baae.2023.12.003>

Bobev, S. G., Kehajov, D. K., Maes, M., Willekens, K., & Bost, M. A. (2012). *Compost Influence on Some Air-Borne Diseases and Pests in Strawberry* (WOS:000304527500118). *927*, 959–966. <https://doi.org/10.17660/ActaHortic.2012.927.118>

Bocco, R., Lee, M., Kim, D., Ahn, S., Park, J. W., Lee, S. Y., & Han, J. H. (2021). Endophytic Isaria javanica pf185 Persists after Spraying and Controls Myzus persicae (Hemiptera: Aphididae) and Colletotrichum acutatum (Glomerellales: Glomerellaceae) in Pepper. *INSECTS*, *12*(7). <https://doi.org/10.3390/insects12070631>

Böckmann, E. (2022). Effects of insect net coverage in field vegetables on pests, diseases, natural enemies, and yield. *JOURNAL OF PLANT DISEASES AND PROTECTION*, *129*(6), 1401–1415. <https://doi.org/10.1007/s41348-022-00644-1>

Boni, S. B., Mwashimaha, R. A., Mlowe, N., Sotelo-Cardona, P., & Nordey, T. (2021). Efficacy of indigenous entomopathogenic fungi against the black aphid, Aphis fabae Scopoli under controlled conditions in Tanzania. *INTERNATIONAL JOURNAL OF TROPICAL INSECT SCIENCE*, *41*(2), 1643–1651. <https://doi.org/10.1007/s42690-020-00365-8>

Bonman, J. M., Bockelman, H. E., Jackson, L. F., & Steffenson, B. J. (2005). Disease and insect resistance in cultivated barley accessions from the USDA national small grains collection. *CROP SCIENCE*, *45*(4), 1271–1280. <https://doi.org/10.2135/cropsci2004.0546>

Bonner, T. J., Pell, J. K., & Gray, S. N. (2003). A novel computerised image analysis method for the measurement of production of conidia from the aphid pathogenic fungus Erynia neoaphidis. *FEMS Microbiology Letters*, *220*(1), 75–80. Scopus. <https://doi.org/10.1016/S0378-1097(03)00063-6>

Borkakati, R. N., Saikia, D. K., & Ramanujam, B. (2019). Evaluation of entomopathogenic fungi against sucking pests of Bhut Jalakia. *Journal of Biological Control*, *33*(2), 155–159. Scopus. <https://doi.org/10.18311/jbc/2019/22605>

Bouchery, Y., Givord, L., & Monestiez, P. (1990). Comparison of short- and long-feed transmission of the cauliflower mosaic virus Cabb-S strain and SΔII hybrid by two species of aphid: Myzus persicae (Sulzer) and Brevicoryne brassicae (L.). *Research in Virology*, *141*(6), 677–683. Scopus. <https://doi.org/10.1016/0923-2516(90)90040-P>

Boukar, O., Fatokun, C. A., Huynh, B. L., Roberts, P. A., & Close, T. J. (2016). Genomic Tools in Cowpea Breeding Programs: Status and Perspectives. *FRONTIERS IN PLANT SCIENCE*, *7*. <https://doi.org/10.3389/fpls.2016.00757>

Bournoville, R. (1996). Net reproductive rate of the pea aphid (Acyrthosiphon pisum Harris) and medicagenic acid content of lucerne. *AGRONOMIE*, *16*(2), 89–94. <https://doi.org/10.1051/agro:19960202>

Bowen, R., Bardeau, A., Schultz, S., & Hartman, G. L. (2022). Registration of seven disease- and pest-resistant vegetable soybean germplasm lines. *JOURNAL OF PLANT REGISTRATIONS*, *16*(2), 438–445. <https://doi.org/10.1002/plr2.20215>

Boykova, I., Yuzikhin, O., Novikova, I., Ulianich, P., Eliseev, I., Shaposhnikov, A., Yakimov, A., & Belimov, A. (2023). Strain Streptomyces sp. P-56 Produces Nonactin and Possesses Insecticidal, Acaricidal, Antimicrobial and Plant Growth-Promoting Traits. *MICROORGANISMS*, *11*(3). <https://doi.org/10.3390/microorganisms11030764>

Braman, S. K., & Quick, J. C. (2018). Differential Bee Attraction Among Crape Myrtle Cultivars (Lagerstroemia spp.: Myrtales: Lythraceae). *ENVIRONMENTAL ENTOMOLOGY*, *47*(5), 1203–1208. <https://doi.org/10.1093/ee/nvy117>

Braun, H. J. (1977). BEECH (FAGUS-SYLVATICA-L) BARK DISEASE, CAUSED BY CRYPTOCOCCUS-FAGI BAR .2. PROGRESS OF DISEASE. *EUROPEAN JOURNAL OF FOREST PATHOLOGY*, *7*(2), 76–93.

Breen, J. P. (1992). Temperature and seasonal effects on expression of Acremonium endophyte-enhanced resistance to Schizaphis graminum (Homoptera: Aphididae). *Environmental Entomology*, *21*(1), 68–74. Scopus. <https://doi.org/10.1093/ee/21.1.68>

Breen, J. P. (1993). ENHANCED RESISTANCE TO 3 SPECIES OF APHIDS (HOMOPTERA, APHIDIDAE) IN ACREMONIUM ENDOPHYTE-INFECTED TURFGRASSES. *JOURNAL OF ECONOMIC ENTOMOLOGY*, *86*(4), 1279–1286. <https://doi.org/10.1093/jee/86.4.1279>

Brezeanu, C., Antal-Tremurici, A., Bute, A., Calara, M., Bouruc, D., & Brezeanu, P. M. (2022). *Tomato cultivar trials for productivity, quality, and quality perception in organic farming system*. *1354*, 335–342. Scopus. <https://doi.org/10.17660/ActaHortic.2022.1354.43>

Brobyn, P. J., Clark, S. J., & Wilding, N. (1988). The effect of fungus infection of Metopolophium dirhodum [Hom.: Aphididae] on the oviposition behaviour of the aphid parasitoid Aphidius rhopalosiphi [Hym.: Aphidiidae]. *Entomophaga*, *33*(3), 333–338. Scopus. <https://doi.org/10.1007/BF02372623>

Brotman, Y., Silberstein, L., Kovalski, I., Perin, C., Dogimont, C., Pitrat, M., Klingler, J., Thompson, G. A., & Perl-Treves, R. (2002). Resistance gene homologues in melon are linked to genetic loci conferring disease and pest resistance. *Theoretical and Applied Genetics*, *104*(6–7), 1055–1063. Scopus. <https://doi.org/10.1007/s00122-001-0808-x>

Brown, M. W., & Tworkoski, T. (2004). Pest management benefits of compost mulch in apple orchards. *AGRICULTURE ECOSYSTEMS & ENVIRONMENT*, *103*(3), 465–472. <https://doi.org/10.1016/j.agee.2003.11.006>

Bubica Bustos, L. M., Ueno, A. C., Di Leo, T. D., Crocco, C. D., Martínez-Ghersa, M. A., Molina-Montenegro, M. A., & Gundel, P. E. (2020). Maternal exposure to ozone modulates the endophyte-conferred resistance to aphids in lolium multiflorum plants. *Insects*, *11*(9), 1–16. Scopus. <https://doi.org/10.3390/insects11090548>

Bueno, V. H. P., Parra, J. R. P., Bettiol, W., & van Lenteren, J. C. (2020). *Biological Control in Brazil* (WOS:000861824200008).

Bugti, G. A., Bin, W., Na, C., & Feng, L. H. (2018). Pathogenicity of Beauveria bassiana strain 202 against sap-sucking insect pests. *Plant Protection Science*, *54*(2), 111–117. Scopus. <https://doi.org/10.17221/45/2017-PPS>

Bultman, T. L., Aguilera, A., & Sullivan, T. (2012). Influence of fungal isolates infecting tall fescue on multitrophic interactions. *FUNGAL ECOLOGY*, *5*(3), 372–378. <https://doi.org/10.1016/j.funeco.2011.06.004>

Burlakoti, R. R., & Dossett, M. (2020). *Past efforts and future perspectives of managing major diseases of red raspberries in British Columbia* (WOS:000948143900056). *1277*, 397–402. <https://doi.org/10.17660/ActaHortic.2020.1277.56>

Bushra, S., Tariq, M., Naeem, M., Ashfaq, M., Bodlah, I., & Ali, M. (2017). Effect of semiochemicals and plant extracts on performance of aphid parasitoid, diaeretiella rapae. *Pakistan Journal of Zoology*, *49*(2), 615–621. Scopus. <https://doi.org/10.17582/journal.pjz/2017.49.2.615.621>

Bustos, L. B. M., Ueno, A. C., Biganzoli, F., Card, S. D., Mace, W. J., Martínez-Ghersa, M. A., & Gundel, P. E. (2022). Can Aphid Herbivory Induce Intergenerational Effects of Endophyte-conferred Resistance in Grasses? *JOURNAL OF CHEMICAL ECOLOGY*, *48*(11–12), 867–881. <https://doi.org/10.1007/s10886-022-01390-2>

Bustos, L. M. B., Ueno, A. C., Di Leo, T. D., Crocco, C. D., Martínez-Ghersa, M. A., Molina-Montenegro, M. A., & Gundel, P. E. (2020). Maternal Exposure to Ozone Modulates the Endophyte-Conferred Resistance to Aphids in Lolium multiflorum Plants. *INSECTS*, *11*(9). <https://doi.org/10.3390/insects11090548>

Butt, T. M., & Beckett, A. (1984a). Ultrastructure and behaviour of the spindle pole body of the aphid-pathogenic fungus Erynia neoaphidis. *Protoplasma*, *120*(1–2), 61–71. Scopus. <https://doi.org/10.1007/BF01287618>

Butt, T. M., & Beckett, A. (1984b). Ultrastructure of mitosis in the aphid-pathogenic fungus Erynia neoaphidis. *Protoplasma*, *120*(1–2), 72–83. Scopus. <https://doi.org/10.1007/BF01287619>

Butt, T. M., Ibrahim, L., Clark, S. J., & Beckett, A. (1995). THE GERMINATION BEHAVIOR OF METARHIZIUM-ANISOPLIAE ON THE SURFACE OF APHID AND FLEA BEETLE CUTICLES. *MYCOLOGICAL RESEARCH*, *99*, 945–950. <https://doi.org/10.1016/S0953-7562(09)80754-5>

Buxton, J. H., & Madge, D. S. (1976). EVALUATION OF EUROPEAN EARWIG (FORFICULA-AURICULARIA) AS A PREDATOR OF DAMSON-HOP APHID (PHORODON-HUMULI) .1. FEEDING EXPERIMENTS. *ENTOMOLOGIA EXPERIMENTALIS ET APPLICATA*, *19*(2), 109–114. <https://doi.org/10.1111/j.1570-7458.1976.tb02587.x>

Buxton, J., & Wardlow, L. (1992). Two years of trials with biological control programmes in all ‐year‐round chrysanthemums. *EPPO Bulletin*, *22*(3), 503–511. Scopus. <https://doi.org/10.1111/j.1365-2338.1992.tb00537.x>

Cai, N., Nong, X., Liu, R., McNeill, M. R., Wang, G., Zhang, Z., & Tu, X. (2023). The Conserved Cysteine-Rich Secretory Protein MaCFEM85 Interacts with MsWAK16 to Activate Plant Defenses. *International Journal of Molecular Sciences*, *24*(4). Scopus. <https://doi.org/10.3390/ijms24044037>

Calvin, W., Beuzelin, J. M., Liburd, O. E., Branham, M. A., & Simon, L. J. (2021). Effects of biological insecticides on the sugarcane aphid, Melanaphis sacchari (Zehntner) (Hemiptera: Aphididae), in sorghum. *CROP PROTECTION*, *142*. <https://doi.org/10.1016/j.cropro.2020.105528>

Campbell, C. A. M. (1978). REGULATION OF DAMSON-HOP APHID (PHORODON-HUMULI (SCHRANK)) ON HOPS (HUMULUS-LUPULUS L) BY PREDATORS. *JOURNAL OF HORTICULTURAL SCIENCE & BIOTECHNOLOGY*, *53*(3), 235–242. <https://doi.org/10.1080/00221589.1978.11514824>

Cantone, F. A., & Vandenberg, J. D. (1999a). Genetic transformation and mutagenesis of the entomopathogenic fungus Paecilomyces fumosoroseus. *JOURNAL OF INVERTEBRATE PATHOLOGY*, *74*(3), 281–288. <https://doi.org/10.1006/jipa.1999.4885>

Cantone, F. A., & Vandenberg, J. D. (1999b). Use of the green fluorescent protein for investigations of Paecilomyces fumosoroseus in insect hosts. *JOURNAL OF INVERTEBRATE PATHOLOGY*, *74*(2), 193–197. <https://doi.org/10.1006/jipa.1999.4864>

Cardozo, W. V. (2023). *Sanitation of colonial vineyards in the interandean valleys of Bolivia* (P. Roca, Ed.; Vol. 56). EDP Sciences; Scopus. <https://doi.org/10.1051/bioconf/20235601020>

Carroll, D. P., & Hoyt, S. C. (1984). AUGMENTATION OF EUROPEAN EARWIGS (DERMAPTERA, FORFICULIDAE) FOR BIOLOGICAL-CONTROL OF APPLE APHID (HOMOPTERA, APHIDIDAE) IN AN APPLE ORCHARD. *JOURNAL OF ECONOMIC ENTOMOLOGY*, *77*(3), 738–740. <https://doi.org/10.1093/jee/77.3.738>

Carroll, D. P., Walker, J. T. S., & Hoyt, S. C. (1985). EUROPEAN EARWIGS (DERMAPTERA, FORFICULIDAE) FAIL TO CONTROL APPLE APHIDS ON BEARING APPLE-TREES AND WOOLLY APPLE APHIDS (HOMOPTERA, APHIDIDAE) IN APPLE ROOTSTOCK STOOL BEDS. *JOURNAL OF ECONOMIC ENTOMOLOGY*, *78*(4), 972–974. <https://doi.org/10.1093/jee/78.4.972>

Cascone, P., Iodice, L., Gualtieri, L., Russo, A., Cesaro, P., Yang, Z., Ruocco, M., Monti, M. M., Massa, N., Lingua, G., & Guerrieri, E. (2024). Feeding specialization shapes the bottom-up effect of arbuscular mycorrhizal fungi across a plant–aphid–parasitoid system. *Plants People Planet*. Scopus. <https://doi.org/10.1002/ppp3.10493>

Casida, J. E., & Durkin, K. A. (2017). Pesticide chemical research in toxicology: Lessons from nature. *Chemical Research in Toxicology*, *30*(1), 94–104. Scopus. <https://doi.org/10.1021/acs.chemrestox.6b00303>

Castagnoli, M., Liguori, M., Simoni, S., & Duso, C. (2005). Toxicity of some insecticides to Tetranychus urticae, Neoseiulus californicus and Tydeus californicus. *BIOCONTROL*, *50*(4), 611–622. <https://doi.org/10.1007/s10526-004-8121-7>

Castro, J. A., Pascuas, N. V., Vera, M. E. B., & Ramirez, E. C. (2006). EVALUATION OF THE EXTRACT OF PARROTWEED (Bocconia frutescens L.) IN THE MANAGEMENT OF FITOSANATARY PROBLEMS OF AGRICULTURAL INTEREST. *CULTURA Y DROGA*, *13*, 177–210.

Caussanel, C., & Albouy, V. (1991). DERMAPTERA OF FRANCE - PESTS OR USEFUL INSECTS. *BULLETIN DE LA SOCIETE ZOOLOGIQUE DE FRANCE-EVOLUTION ET ZOOLOGIE*, *116*(3–4), 229–234.

Cerkauskas, R. F., Koike, S. T., Azad, H. R., Lowery, D. T., & Stobbs, L. W. (2006). Diseases, pests, and abiotic disorders of greenhouse-grown water spinach (Ipomoea aquatica) in Ontario and California. *CANADIAN JOURNAL OF PLANT PATHOLOGY*, *28*(1), 63–70. <https://doi.org/10.1080/07060660609507271>

Cerkauskas, R. F., Stobbs, L. W., Lowery, D. T., Van Driel, L., Liu, W., & VanSchagen, J. (1998). Diseases, pests, and abiotic problems associated with oriental cruciferous vegetables in southern Ontario in 1993-1994. *CANADIAN JOURNAL OF PLANT PATHOLOGY*, *20*(1), 87–94. <https://doi.org/10.1080/07060669809500449>

Çetin, G., Hantas, C., Soyergin, S., Burak, M., & Eris, A. (2008). *Studies on Integrated Pest Management (IPM) in Sweet Cherry Orchards in the Marmara Region of Turkey* (WOS:000260482900150). 925-+. <https://doi.org/10.17660/ActaHortic.2008.795.150>

Chakraborty, S., Britton, M., Martínez-García, P. J., & Dandekar, A. M. (2016). Deep RNA-Seq profile reveals biodiversity, plant-microbe interactions and a large family of NBS-LRR resistance genes in walnut (Juglans regia) tissues. *AMB EXPRESS*, *6*. <https://doi.org/10.1186/s13568-016-0182-3>

Chamkhi, I., Hnini, M., & Aurag, J. (2022). Conventional Medicinal Uses, Phytoconstituents, and Biological Activities of Euphorbia officinarum L.: A Systematic Review. *Advances in Pharmacological and Pharmaceutical Sciences*, *2022*. Scopus. <https://doi.org/10.1155/2022/9971085>

Chandler, D. (1992). THE POTENTIAL OF ENTOMOPATHOGENIC FUNGI TO CONTROL THE LETTUCE ROOT APHID, PEMPHIGUS-BURSARIUS. *PHYTOPARASITICA*, *20*, S11–S15. <https://doi.org/10.1007/BF02980401>

Chandler, D. (1997). Selection of an isolate of the insect pathogenic fungus Metarhizium anisopliae virulent to the lettuce root aphid, Pemphigus bursarius. *BIOCONTROL SCIENCE AND TECHNOLOGY*, *7*(1), 95–104. <https://doi.org/10.1080/09583159731081>

Chandler, D., Heale, J. B., & Gillespie, A. T. (1993). COMPETITIVE INTERACTION BETWEEN STRAINS OF VERTICILLIUM-LECANII ON 2 INSECT HOSTS. *ANNALS OF APPLIED BIOLOGY*, *122*(3), 435–440. <https://doi.org/10.1111/j.1744-7348.1993.tb04046.x>

Charters, M. D., Sait, S. M., & Field, K. J. (2020). Aphid Herbivory Drives Asymmetry in Carbon for Nutrient Exchange between Plants and an Arbuscular Mycorrhizal Fungus. *Current Biology*, *30*(10), 1801-1808.e5. Scopus. <https://doi.org/10.1016/j.cub.2020.02.087>

Chaudhary, R., Peng, H. C., He, J., MacWilliams, J., Teixeira, M., Tsuchiya, T., Chesnais, Q., Mudgett, M. B., & Kaloshian, I. (2019). Aphid effector Me10 interacts with tomato TFT7, a 14-3-3 isoform involved in aphid resistance. *New Phytologist*, *221*(3), 1518–1528. Scopus. <https://doi.org/10.1111/nph.15475>

Chehab, E. W., Kaspi, R., Savchenko, T., Rowe, H., Negre-Zakharov, F., Kliebenstein, D., & Dehesh, K. (2008). Distinct Roles of Jasmonates and Aldehydes in Plant-Defense Responses. *PLOS ONE*, *3*(4). <https://doi.org/10.1371/journal.pone.0001904>

Chelkowski, J., Tyrka, M., & Sobkiewicz, A. (2003). Resistance genes in barley (Hordeum vulgare L.) and their identification with molecular markers. *JOURNAL OF APPLIED GENETICS*, *44*(3), 291–309.

Chen, B., Li, Z. Y., & Feng, M. G. (2008). Occurrence of entomopathogenic fungi in migratory alate aphids in Yunnan Province of China. *BioControl*, *53*(2), 317–326. Scopus. <https://doi.org/10.1007/s10526-006-9063-z>

Chen, B., Zhang, Y., Sun, Z. W., Liu, Z. W., Zhang, D. M., Yang, J., Wang, G. I., Wu, J. H., Ke, H. F., Meng, C. S., Wu, L. Z., Yan, Y. Y., Cui, Y. R., Li, Z. K., Wu, L. Q., Zhang, G. Y., Wang, X. F., & Ma, Z. Y. (2021). Tissue-specific expression of GhnsLTPs identified via GWAS sophisticatedly coordinates disease and insect resistance by regulating metabolic flux redirection in cotton. *PLANT JOURNAL*, *107*(3), 831–846. <https://doi.org/10.1111/tpj.15349>

Chen, C., & Feng, M. (2002). Evidence for transmission of aphid-pathogenic fungi by migratory flight of Myzus persicae alates. *Chinese Science Bulletin*, *47*(23), 1987–1989. Scopus. <https://doi.org/10.1360/02tb9431>

Chen, L. G., Zhang, L. P., & Yu, D. Q. (2010). Wounding-Induced WRKY8 Is Involved in Basal Defense in Arabidopsis. *MOLECULAR PLANT-MICROBE INTERACTIONS*, *23*(5), 558–565. <https://doi.org/10.1094/MPMI-23-5-0558>

Chen, Y., Meissle, M., Xue, J. B., Zhang, N., Ma, S. L., Guo, A. P., Liu, B., Peng, Y. F., Song, X. Y., Yang, Y., & Li, Y. H. (2023). Expression of Cry1Ab/2Aj Protein in Genetically Engineered Maize Plants and Its Transfer in the Arthropod Food Web. *PLANTS-BASEL*, *12*(23). <https://doi.org/10.3390/plants12234057>

Cheng, Y., & Jones, R. A. C. (1999). Distribution and incidence of necrotic and non-necrotic strains of bean yellow mosaic virus in wild and crop lupins. *AUSTRALIAN JOURNAL OF AGRICULTURAL RESEARCH*, *50*(4), 589–599. <https://doi.org/10.1071/A98116>

Cheong, P. C. H., Glare, T. R., Rostás, M., Haines, S., Brookes, J. J., & Ford, S. (2020). Lack of involvement of chitinase in direct toxicity of Beauveria bassiana cultures to the aphid Myzus persicae. *JOURNAL OF INVERTEBRATE PATHOLOGY*, *169*. <https://doi.org/10.1016/j.jip.2019.107276>

Chinniah, C., Ravikumar, A., Kalyanasundaram, M., & Parthiban, P. (2016). Management of sucking pests, by integration of organic sources of amendments and foliar application of entomopathogenic fungi on chilli. *Journal of Biopesticides*, *9*(1), 34–40. Scopus.

Chitty, R. P., & Gange, A. C. (2022). Reciprocal interactions between aphids and arbuscular mycorrhizal fungi across plant generations. *Arthropod-Plant Interactions*, *16*(1), 33–43. Scopus. <https://doi.org/10.1007/s11829-021-09875-9>

Choi, B. H., Kim, C. S., Jeong, Y. J., Park, I. H., Han, S. G., & Yoon, T. M. (2021). Resistance evaluation of g, cg, or m series apple rootstocks to soil-borne diseases (Phytophthora root rot, white root rot, and southern blight) and woolly apple aphid. *Horticultural Science and Technology*, *39*(2), 167–174. Scopus. <https://doi.org/10.7235/HORT.20210015>

Christensen, M. J., & Latch, G. C. M. (1991). VARIATION AMONG ISOLATES OF ACREMONIUM ENDOPHYTES (A-COENOPHIALUM AND POSSIBLY A-TYPHINUM) FROM TALL FESCUE (FESTUCA-ARUNDINACEA). *MYCOLOGICAL RESEARCH*, *95*, 1123–1126. <https://doi.org/10.1016/S0953-7562(09)80558-3>

Christias, C., Hatzipapas, P., Dara, A., Kaliafas, A., & Chrysanthis, G. (2001). Alternaria alternata, a new pathotype pathogenic to aphids. *BIOCONTROL*, *46*(1), 105–124. <https://doi.org/10.1023/A:1009930112152>

Chu, Y. C., & Chang, J. C. (2017a). *Assessment of feasibility for guava (&ITPsidium guajava&IT) grown in the fixed structure of field net-house* (WOS:000428259300014). *1166*, 101–105. <https://doi.org/10.17660/ActaHortic.2017.1166.14>

Chu, Y. C., & Chang, J. C. (2017b). *Assessment of feasibility for guava (Psidium guajava) grown in the fixed structure of field net-house* (L. Wen-Li, Ed.; Vol. 1166, pp. 101–106). International Society for Horticultural Science; Scopus. <https://doi.org/10.17660/ActaHortic.2017.1166.14>

Clement, S. L., Hu, J., Stewart, A. V., Wang, B., & Elberson, L. R. (2011). Detrimental and neutral effects of a wild grass-fungal endophyte symbiotum on insect preference and performance. *Journal of Insect Science*, *11*. Scopus. <https://doi.org/10.1673/031.011.7701>

Clement, S. L., Lester, D. G., Wilson, A. D., Johnson, R. C., & Bouton, J. H. (1996). Expression of Russian wheat aphid (Homoptera: Aphididae) resistance in genotypes of tall fescue harboring different isolates of Acremonium endophyte. *JOURNAL OF ECONOMIC ENTOMOLOGY*, *89*(3), 766–770. <https://doi.org/10.1093/jee/89.3.766>

Clement, S. L., Wilson, A. D., Lester, D. G., & Davitt, C. M. (1997). Fungal endophytes of wild barley and their effects on Diuraphis noxia population development. *ENTOMOLOGIA EXPERIMENTALIS ET APPLICATA*, *82*(3), 275–281. <https://doi.org/10.1046/j.1570-7458.1997.00141.x>

Clements, R. J., Turner, J. W., Irwin, J. A. G., Langdon, P. W., & Bray, R. A. (1984). BREEDING DISEASE RESISTANT, APHID RESISTANT LUCERNE FOR SUB-TROPICAL QUEENSLAND. *AUSTRALIAN JOURNAL OF EXPERIMENTAL AGRICULTURE*, *24*(125), 178–188. <https://doi.org/10.1071/EA9840178>

Clifton, E. H., Castrillo, L. A., Jaronski, S. T., & Hajek, A. E. (2023). Cryptic diversity and virulence of Beauveria bassiana recovered from Lycorma delicatula (spotted lanternfly) in eastern Pennsylvania. *FRONTIERS IN INSECT SCIENCE*, *3*. <https://doi.org/10.3389/finsc.2023.1127682>

Clifton, E. H., Jaronski, S. T., Coates, B. S., Hodgson, E. W., & Gassmann, A. J. (2018). Effects of endophytic entomopathogenic fungi on soybean aphid and identification of Metarhizium isolates from agricultural fields. *PLOS ONE*, *13*(3). <https://doi.org/10.1371/journal.pone.0194815>

Cole, R. A. (1997a). Comparison of feeding behaviour of two Brassica pests Brevicoryne brassicae and Myzus persicae on wild and cultivated brassica species. *Entomologia Experimentalis et Applicata*, *85*(2), 135–143. Scopus.

Cole, R. A. (1997b). Comparison of feeding behaviour of two Brassica pests Brevicoryne brassicae and Myzus persicae on wild and cultivated Brassica species. *ENTOMOLOGIA EXPERIMENTALIS ET APPLICATA*, *85*(2), 135–143. <https://doi.org/10.1046/j.1570-7458.1997.00243.x>

Coleman, J. S., & Jones, C. G. (1988). ACUTE OZONE STRESS ON EASTERN COTTONWOOD (POPULUS-DELTOIDES BARTR) AND THE PEST POTENTIAL OF THE APHID, CHAITOPHORUS-POPULICOLA THOMAS (HOMOPTERA, APHIDIDAE). *ENVIRONMENTAL ENTOMOLOGY*, *17*(2), 207–212. <https://doi.org/10.1093/ee/17.2.207>

Collinson, N. P., Mann, R. C., Giri, K., Malipatil, M., Kaur, J., Spangenberg, G., & Valenzuela, I. (2020). Novel bioassay to assess antibiotic effects of fungal endophytes on aphids. *PLOS ONE*, *15*(2). <https://doi.org/10.1371/journal.pone.0228813>

Conceschi, M. R., D’Alessandro, C. P., Moral, R. A., Demétrio, C. G. B., & Júnior, I. D. (2016). Transmission potential of the entomopathogenic fungi Isaria fumosorosea and Beauveria bassiana from sporulated cadavers of Diaphorina citri and Toxoptera citricida to uninfected D. citri adults. *BioControl*, *61*(5), 567–577. Scopus. <https://doi.org/10.1007/s10526-016-9733-4>

Conijn, C. G. M. (2014). *Developments in the Control of Lily Diseases* (WOS:000343865600023). *1027*, 213–229. <https://doi.org/10.17660/ActaHortic.2014.1027.23>

Conner, P. J. (2014a). Performance of 19 Pecan Cultivars and Selections in Southern Georgia. *HORTTECHNOLOGY*, *24*(3), 407–412. <https://doi.org/10.21273/HORTTECH.24.3.407>

Conner, P. J. (2014b). Performance of Nine Pecan Cultivars in Southern Georgia. *JOURNAL OF THE AMERICAN POMOLOGICAL SOCIETY*, *68*(3), 118–124.

Converse, R. H. (1981). INFECTION OF CULTIVATED STRAWBERRIES BY TOMATO RINGSPOT VIRUS. *PHYTOPATHOLOGY*, *71*(11), 1149–1152. <https://doi.org/10.1094/Phyto-71-1149>

Coppola, M., Cascone, P., Chiusano, M. L., Colantuono, C., Lorito, M., Pennacchio, F., Rao, R., Woo, S. L., Guerrieri, E., & Digilio, M. C. (2017). Trichoderma harzianum enhances tomato indirect defense against aphids. *INSECT SCIENCE*, *24*(6), 1025–1033. <https://doi.org/10.1111/1744-7917.12475>

Coppola, M., Cascone, P., Di Lelio, I., Woo, S. L., Lorito, M., Rao, R., Pennacchio, F., Guerrieri, E., & Digilio, M. C. (2019). Trichoderma atroviride P1 Colonization of Tomato Plants Enhances Both Direct and Indirect Defense Barriers Against Insects. *FRONTIERS IN PHYSIOLOGY*, *10*. <https://doi.org/10.3389/fphys.2019.00813>

Coppola, M., Corrado, G., Coppola, V., Cascone, P., Martinelli, R., Digilio, M. C., Pennacchio, F., & Rao, R. (2015). Prosystemin Overexpression in Tomato Enhances Resistance to Different Biotic Stresses by Activating Genes of Multiple Signaling Pathways. *PLANT MOLECULAR BIOLOGY REPORTER*, *33*(5), 1270–1285. <https://doi.org/10.1007/s11105-014-0834-x>

Coppola, M., Di Lelio, I., Romanelli, A., Gualtieri, L., Molisso, D., Ruocco, M., Avitabile, C., Natale, R., Cascone, P., Guerrieri, E., Pennacchio, F., & Rao, R. (2019). Tomato Plants Treated with Systemin Peptide Show Enhanced Levels of Direct and Indirect Defense Associated with Increased Expression of Defense-Related Genes. *PLANTS-BASEL*, *8*(10). <https://doi.org/10.3390/plants8100395>

Coppola, M., Diretto, G., Digilio, M. C., Woo, S. L., Giuliano, G., Molisso, D., Pennacchio, F., Lorito, M., & Rao, R. (2019). Transcriptome and Metabolome Reprogramming in Tomato Plants by Trichoderma harzianum strain T22 Primes and Enhances Defense Responses Against Aphids. *FRONTIERS IN PHYSIOLOGY*, *10*. <https://doi.org/10.3389/fphys.2019.00745>

Costa, C., & Stassen, P. J. C. (2008). *Preliminary Results of Research on New Apple Rootstocks in South Africa* (WOS:000261331900033). 225-+. <https://doi.org/10.17660/ActaHortic.2008.772.33>

Costa, C., & Stassen, P. J. C. (2011). *Overview of Apple Rootstocks in South Africa* (WOS:000313512600050). *903*, 385–390. <https://doi.org/10.17660/ActaHortic.2011.903.50>

Costello, M. J. (1994). BROCCOLI GROWTH, YIELD AND LEVEL OF APHID INFESTATION IN LEGUMINOUS LIVING MULCHES. *BIOLOGICAL AGRICULTURE & HORTICULTURE*, *10*(3), 207–222. <https://doi.org/10.1080/01448765.1994.9754669>

Costello, M. J. (1995). SPECTRAL REFLECTANCE FROM A BROCCOLI CROP WITH VEGETATION OR SOIL AS BACKGROUND - INFLUENCE ON IMMIGRATION BY BREVICORYNE-BRASSICAE AND MYZUS-PERSICAE. *ENTOMOLOGIA EXPERIMENTALIS ET APPLICATA*, *75*(2), 109–118. <https://doi.org/10.1111/j.1570-7458.1995.tb01916.x>

Cottrell, T. E., & Riddick, E. W. (2012). Limited transmission of the ectoparasitic fungus Hesperomyces virescens between lady beetles. *Psyche (London)*. Scopus. <https://doi.org/10.1155/2012/814378>

Cowger, C., Weisz, R., Anderson, J. M., & Horton, J. R. (2010). Maize Debris Increases Barley Yellow Dwarf Virus Severity in North Carolina Winter Wheat. *AGRONOMY JOURNAL*, *102*(2), 688–695. <https://doi.org/10.2134/agronj2009.0357>

Cravedi, P., & Jörg, E. (1996). *Special challenges for IFP in stone and soft fruit*. *422*, 48–56. Scopus. <https://doi.org/10.17660/ActaHortic.1996.422.7>

Cross, J., Fountain, M., Markó, V., & Nagy, C. (2015). Arthropod ecosystem services in apple orchards and their economic benefits. *ECOLOGICAL ENTOMOLOGY*, *40*, 82–96. <https://doi.org/10.1111/een.12234>

Curtis, J. E., Price, T. V., & Ridland, P. M. (2003). Initial development of a spray formulation which promotes germination and growth of the fungal entomopathogen Verticillium lecanii (Zimmerman) Viegas (Deuteromycotina: Hyphomycetes) on capsicum leaves (Capsicum annuum grossum Sendt. Var. California Wonder) and infection of Myzus persicae Sulzer (Homoptera: Aphididae). *BIOCONTROL SCIENCE AND TECHNOLOGY*, *13*(1), 35–46. <https://doi.org/10.1080/0958315021000054377>

Da Cunha, J. C. S., Swoboda, M. H., & Sword, G. A. (2022). Olfactometer Responses of Convergent Lady Beetles Hippodamia convergens (Coleoptera: Coccinellidae) to Odor Cues from Aphid-Infested Cotton Plants Treated with Plant-Associated Fungi. *Insects*, *13*(2). Scopus. <https://doi.org/10.3390/insects13020157>

Da Silva, R. R., Vargas-Flores, J., Sánchez-Choy, J., Oliva-Paredes, R., Alarcón-Castillo, T., & Panduro, P. P. V. (2020). Beauveria bassiana and Metarhizium anisopliae as compatible and efficient controllers of plague insects in aquaponic crops. *SCIENTIA AGROPECUARIA*, *11*(3), 419–426. <https://doi.org/10.17268/sci.agropecu.2020.03.14>

Dai, Y. J., Ji, W. W., Chen, T., Zhang, W. J., Liu, Z. H., Ge, F., & Yuan, S. (2010). Metabolism of the Neonicotinoid Insecticides Acetamiprid and Thiacloprid by the Yeast Rhodotorula mucilaginosa Strain IM-2. *JOURNAL OF AGRICULTURAL AND FOOD CHEMISTRY*, *58*(4), 2419–2425. <https://doi.org/10.1021/jf903787s>

Dalinova, A., Chisty, L., Kochura, D., Garnyuk, V., Petrova, M., Prokofieva, D., Yurchenko, A., Dubovik, V., Ivanov, A., Smirnov, S., Zolotarev, A., & Berestetskiy, A. (2020). Isolation and Bioactivity of Secondary Metabolites from Solid Culture of the Fungus, Alternaria sonchi. *BIOMOLECULES*, *10*(1). <https://doi.org/10.3390/biom10010081>

Damsteegt, V. D., Stone, A. L., Smith, O. P., McDaniel, L., Sherman, D. J., Dardick, C., Hammond, J., Jordan, R., & Schneider, W. L. (2013). A previously undescribed potyvirus isolated and characterized from arborescent Brugmansia. *ARCHIVES OF VIROLOGY*, *158*(6), 1235–1244. <https://doi.org/10.1007/s00705-012-1600-8>

Dang, J. L., Gleason, M. L., Wang, B. L., & Feng, J. (2023). Effects of Peganum harmala extracts and synthetic chemical fungicides on controlling early blight of tomato in the central shaanxi plain of China. *CROP PROTECTION*, *166*. <https://doi.org/10.1016/j.cropro.2022.106177>

Dang, J. L., Shi, X. R., Lin, Y. Q., Gleason, M. L., & Feng, J. (2024). Controlling Phytophthora blight of pepper in Guizhou Province of China using Stellera chamaejasme extracts and synthetic chemical fungicides. *PLANT PROTECTION SCIENCE*, *60*(1), 53–64. <https://doi.org/10.17221/84/2023-PPS>

Dang, M., Liu, M., Huang, L., Ou, X., Long, C., Liu, X., Ren, Y., Zhang, P., Huang, M., & Liu, A. (2020). Design, synthesis, and bioactivities of novel pyridazinone derivatives containing 2-phenylthiazole or oxazole skeletons. *Journal of Heterocyclic Chemistry*, *57*(11), 4088–4098. Scopus. <https://doi.org/10.1002/jhet.4118>

Dara, S. K. (2016). Managing Strawberry Pests with Chemical Pesticides and Non-Chemical Alternatives. *International Journal of Fruit Science*, *16*, 129–141. Scopus. <https://doi.org/10.1080/15538362.2016.1195311>

Daskalova, N., & Spetsov, P. (2020). Taxonomic Relationships and Genetic Variability of Wild Secale L. Species as a Source for Valued Traits in Rye, Wheat and Triticale Breeding. *CYTOLOGY AND GENETICS*, *54*(1), 71–81. <https://doi.org/10.3103/S0095452720010041>

Davidson, A. W., & Potter, D. A. (1995). RESPONSE OF PLANT-FEEDING, PREDATORY, AND SOIL-INHABITING INVERTEBRATES TO ACREMONIUM ENDOPHYTE AND NITROGEN-FERTILIZATION IN TALL FESCUE TURF. *JOURNAL OF ECONOMIC ENTOMOLOGY*, *88*(2), 367–379. <https://doi.org/10.1093/jee/88.2.367>

Davies, D. H. K., McRoberts, N., Foster, G. N., Whytock, G. P., Evans, K. A., McKinlay, R. D., Wale, S., Brit Crop Protect, C., Brit Crop Protect, C., & Brit Crop Protect, C. (1997). *COIRE: The impact of the presence of weeds, pests and diseases on crop qualities* (WOS:000071437600114). 711–716.

Davila Olivas, N. H., Frago, E., Thoen, M. P. M., Kloth, K. J., Becker, F. F. M., van Loon, J. J. A., Gort, G., Keurentjes, J. J. B., van Heerwaarden, J., & Dicke, M. (2017). Natural variation in life history strategy of Arabidopsis thaliana determines stress responses to drought and insects of different feeding guilds. *MOLECULAR ECOLOGY*, *26*(11), 2959–2977. <https://doi.org/10.1111/mec.14100>

Davis, J. A., Radcliffe, E. B., Thill, C. A., & Ragsdale, D. W. (2012). Resistance to Aphids, Late Blight and Viruses in Somatic Fusions and Crosses of Solanum tuberosum L. and Solanum bulbocastanum Dun. *AMERICAN JOURNAL OF POTATO RESEARCH*, *89*(6), 489–500. <https://doi.org/10.1007/s12230-012-9272-1>

de Araujo, J. M., Marques, E. J., & de Oliveira, J. V. (2009). Potential of Metarhizium anisopliae and Beauveria bassiana Isolates and Neem Oil to Control the Aphid Lipaphis erysimi (Kalt.) (Hemiptera: Aphididae). *NEOTROPICAL ENTOMOLOGY*, *38*(4), 520–525. <https://doi.org/10.1590/S1519-566X2009000400014>

de Azevedo, A. G. C., Eilenberg, J., Steinwender, B. M., & Sigsgaard, L. (2019). Non-target effects of Metarhizium brunneum (BIPESCO 5/F 52) in soil show that this fungus varies between being compatible with, or moderately harmful to, four predatory arthropods. *BIOLOGICAL CONTROL*, *131*, 18–24. <https://doi.org/10.1016/j.biocontrol.2019.01.002>

de Azevedo, A. G. C., Steinwender, B. M., Eilenberg, J., & Sigsgaard, L. (2017). Interactions among the Predatory Midge Aphidoletes aphidimyza (Diptera: Cecidomyiidae), the Fungal Pathogen Metarhizium brunneum (Ascomycota: Hypocreales), and Maize-Infesting Aphids in Greenhouse Mesocosms. *INSECTS*, *8*(2). <https://doi.org/10.3390/insects8020044>

de Azevedo, A. G. C., Stuart, R. M., & Sigsgaard, L. (2018). Presence of a generalist entomopathogenic fungus influences the oviposition behaviour of an aphid-specific predator. *BioControl*, *63*(5), 655–664. Scopus. <https://doi.org/10.1007/s10526-018-9889-1>

de Ilarduya, O. M., Moore, A. E., & Kaloshian, I. (2001). The tomato Rme1 locus is required for Mi-1-mediated resistance to root-knot nematodes and the potato aphid. *PLANT JOURNAL*, *27*(5), 417–425. <https://doi.org/10.1046/j.1365-313X.2001.01112.x>

De Vos, M., Van Oosten, V. R., Van Poecke, R. M. P., Van Pelt, J. A., Pozo, M. J., Mueller, M. J., Buchala, A. J., Métraux, J. P., Van Loon, L. C., Dicke, M., & Pieterse, C. M. J. (2005). Signal signature and transcriptome changes of Arabidopsis during pathogen and insect attack. *MOLECULAR PLANT-MICROBE INTERACTIONS*, *18*(9), 923–937. <https://doi.org/10.1094/MPMI-18-0923>

De Zutter, N., Audenaert, K., Ameye, M., De Boevre, M., De Saeger, S., Haesaert, G., & Smagghe, G. (2017). The plant response induced in wheat ears by a combined attack of Sitobion avenae aphids and Fusarium graminearum boosts fungal infection and deoxynivalenol production. *MOLECULAR PLANT PATHOLOGY*, *18*(1), 98–109. <https://doi.org/10.1111/mpp.12386>

De Zutter, N., Audenaert, K., Ameye, M., Haesaert, G., & Smagghe, G. (2016). Effect of the mycotoxin deoxynivalenol on grain aphid Sitobion avenae and its parasitic wasp Aphidius ervi through food chain contamination. *ARTHROPOD-PLANT INTERACTIONS*, *10*(4), 323–329. <https://doi.org/10.1007/s11829-016-9432-1>

De Zutter, N., Audenaert, K., Arroyo-Manzanares, N., De Boevre, M., Van Poucke, C., De Saeger, S., Haesaert, G., & Smagghe, G. (2016). Aphids transform and detoxify the mycotoxin deoxynivalenol via a type II biotransformation mechanism yet unknown in animals. *SCIENTIFIC REPORTS*, *6*. <https://doi.org/10.1038/srep38640>

De Zutter, N., Audenaert, K., Haesaert, G., & Smagghe, G. (2012). Preference of cereal aphids for different varieties of winter wheat. *ARTHROPOD-PLANT INTERACTIONS*, *6*(3), 345–350. <https://doi.org/10.1007/s11829-012-9184-5>

Degefu, D. T., Yeshanew, E. S., & Gashawbeza, G. T. (2014). Efficacy of dose-dependent indigenous microbial insecticides against cotton aphid, Aphis gossypii Glover (Homoptera: Aphididae) at various temperature regimes. *INTERNATIONAL JOURNAL OF PEST MANAGEMENT*, *60*(3), 173–179. <https://doi.org/10.1080/09670874.2014.951101>

Delserone, L. M., Cole, H., & Frank, J. A. (1987). THE EFFECTS OF INFECTIONS BY PYRENOPHORA-TERES AND BARLEY YELLOW DWARF VIRUS ON THE FREEZING HARDINESS OF WINTER BARLEY. *PHYTOPATHOLOGY*, *77*(10), 1435–1437. <https://doi.org/10.1094/Phyto-77-1435>

Demirözer, O., Uzun, A., Arici, Ş. E., Gep, I., & Bakay, R. (2016). Insecticidal effect of Fusarium subglutinans on Frankliniella occidentalis (Pergande) (Thysanoptera: Thripidae). *Hellenic Plant Protection Journal*, *9*(2), 66–72. Scopus. <https://doi.org/10.1515/hppj-2016-0008>

Dennis, P., Wratten, S. D., & Sotherton, N. W. (1990). FEEDING-BEHAVIOR OF THE STAPHYLINID BEETLE TACHYPORUS-HYPNORUM IN RELATION TO ITS POTENTIAL FOR REDUCING APHID NUMBERS IN WHEAT. *ANNALS OF APPLIED BIOLOGY*, *117*(2), 267–276. <https://doi.org/10.1111/j.1744-7348.1990.tb04212.x>

Dennis, P., Wratten, S. D., & Sotherton, N. W. (1991). MYCOPHAGY AS A FACTOR LIMITING PREDATION OF APHIDS (HEMIPTERA, APHIDIDAE) BY STAPHYLINID BEETLES (COLEOPTERA, STAPHYLINIDAE) IN CEREALS. *BULLETIN OF ENTOMOLOGICAL RESEARCH*, *81*(1), 25–31. <https://doi.org/10.1017/S0007485300053207>

Desneux, N., Mouttet, R., Bearez, P., & Poncet, C. (2012). *Indirect Two-Way Interactions between Aphids and a Pathogen on Roses* (WOS:000304527500026). *927*, 237–244.

De-Thier, J. S., Pyati, P., Bell, J., Readshaw, J. J., Brown, A. P., & Fitches, E. C. (2023). Heterologous production of the insecticidal pea seed albumin PA1 protein by Pichia pastoris and protein engineering to potentiate aphicidal activity via fusion to snowdrop lectin Galanthus nivalis agglutinin; GNA). *MICROBIAL CELL FACTORIES*, *22*(1). <https://doi.org/10.1186/s12934-023-02176-1>

Devi, K. U., Reddy, N. N. R., Sridevi, D., Sridevi, V., & Mohan, C. M. (2004). Esterase-mediated tolerance to a formulation of the organophosphate insecticide monocrotophos in the entomopathogenic fungus, Beauveria bassiana (Balsamo) Vuill: A promising biopesticide. *PEST MANAGEMENT SCIENCE*, *60*(4), 408–412. <https://doi.org/10.1002/ps.801>

Dhillon, M. K., Singh, N., & Yadava, D. K. (2022). Preventable yield losses and management of mustard aphid, Lipaphis erysimi (Kaltenbach) in different cultivars of Brassica juncea (L.) Czern & Coss. *CROP PROTECTION*, *161*. <https://doi.org/10.1016/j.cropro.2022.106070>

Di Lelio, I., Coppola, M., Comite, E., Molisso, D., Lorito, M., Woo, S. L., Pennacchio, F., Rao, R. S., & Digilio, M. C. (2021). Temperature Differentially Influences the Capacity of Trichoderma Species to Induce Plant Defense Responses in Tomato Against Insect Pests. *FRONTIERS IN PLANT SCIENCE*, *12*. <https://doi.org/10.3389/fpls.2021.678830>

Di Piero, R. M., de Novaes, Q. S., & Pascholati, S. F. (2010). Effect of Agaricus brasiliensis and Lentinula edodes Mushrooms on the Infection of Passionflower with Cowpea aphid-borne mosaic virus. *BRAZILIAN ARCHIVES OF BIOLOGY AND TECHNOLOGY*, *53*(2), 269–278. <https://doi.org/10.1590/S1516-89132010000200004>

Di Piero, R. M., Novaes, Q. S. de, & Pascholati, S. F. (2010). Effect of Agaricus brasiliensis and Lentinula edodes mushrooms on the infection of passionflower with Cowpea aphid-borne mosaic virus. *Brazilian Archives of Biology and Technology*, *53*, 269–278.

Diao, H. L., Xing, P. X., Tian, J., Han, Z. H., Wang, D., Xiang, H. M., Liu, T. X., & Ma, R. Y. (2022). Toxicity of crude toxin protein produced by Cordyceps fumosorosea IF-1106 against Myzus persicae (Sulze). *JOURNAL OF INVERTEBRATE PATHOLOGY*, *194*. <https://doi.org/10.1016/j.jip.2022.107825>

Diao, H., Xing, P., Tian, J., Han, Z., Wang, D., Xiang, H., Liu, T., & Ma, R. (2022). Toxicity of crude toxin protein produced by Cordyceps fumosorosea IF-1106 against Myzus persicae (Sulze). *J Invertebr Pathol*, *194*, 107825. <https://doi.org/10.1016/j.jip.2022.107825>

Díaz, B. M., Legarrea, S., Marcos-García, M. A., & Fereres, A. (2010). The spatio-temporal relationships among aphids, the entomophthoran fungus, Pandora neoaphidis, and aphidophagous hoverflies in outdoor lettuce. *Biological Control*, *53*(3), 304–311. Scopus. <https://doi.org/10.1016/j.biocontrol.2009.12.002>

Diaz, C. E., Andres, M. F., Lacret, R., Cabrera, R., Gimenez, C., Kaushik, N., & Gonzalez-Coloma, A. (2024). Antifeedant, antifungal and nematicidal compounds from the endophyte Stemphylium solani isolated from wormwood. *Scientific Reports*, *14*(1). Scopus. <https://doi.org/10.1038/s41598-024-64467-w>

Dib, H., Jamont, M., Sauphanor, B., & Capowiez, Y. (2011). Predation potency and intraguild interactions between generalist (Forficula auricularia) and specialist (Episyrphus balteatus) predators of the rosy apple aphid (Dysaphis plantaginea). *BIOLOGICAL CONTROL*, *59*(2), 90–97. <https://doi.org/10.1016/j.biocontrol.2011.07.012>

Dib, H., Jamont, M., Sauphanor, B., & Capowiez, Y. (2016a). Individual and combined effects of the generalist Forficula auricularia and the specialist Episyrphus balteatus on Dysaphis plantaginea—Are two predators better than one? *ENTOMOLOGIA EXPERIMENTALIS ET APPLICATA*, *161*(1), 1–10. <https://doi.org/10.1111/eea.12484>

Dib, H., Jamont, M., Sauphanor, B., & Capowiez, Y. (2016b). The feasibility and efficacy of early-season releases of a generalist predator (Forficula auricularia L.) to control populations of the RAA (Dysaphis plantaginea Passerini) in Southeastern France. *BULLETIN OF ENTOMOLOGICAL RESEARCH*, *106*(2), 233–241. <https://doi.org/10.1017/S0007485315001042>

Dib, H., Siegwart, M., Delattre, T., Perrin, M., & Lavigne, C. (2020). Does combining Forficula auricularia L. (Dermaptera: Forficulidae) with Harmonia axyridis Pallas (Coleoptera: Coccinellidae) enhance predation of rosy apple aphid, Dysaphis plantaginea Passerini (Hemiptera: Aphididae)? *BIOLOGICAL CONTROL*, *151*. <https://doi.org/10.1016/j.biocontrol.2020.104394>

Diehl, T., & Fehrmann, H. (1989). WHEAT FUSARIOSES - INFLUENCE OF INFECTION DATE, TISSUE-INJURY AND APHIDS ON LEAF AND EAR ATTACK. *ZEITSCHRIFT FUR PFLANZENKRANKHEITEN UND PFLANZENSCHUTZ-JOURNAL OF PLANT DISEASES AND PROTECTION*, *96*(4), 393–407.

Dik, A. J., Fokkema, N. J., & Vanpelt, J. A. (1991). CONSUMPTION OF APHID HONEYDEW, A WHEAT YIELD REDUCTION FACTOR, BY PHYLLOSPHERE YEASTS UNDER FIELD CONDITIONS. *NETHERLANDS JOURNAL OF PLANT PATHOLOGY*, *97*(4), 209–232. <https://doi.org/10.1007/BF01989819>

Dik, A. J., Fokkema, N. J., & Vanpelt, J. A. (1992). INFLUENCE OF CLIMATIC AND NUTRITIONAL FACTORS ON YEAST POPULATION-DYNAMICS IN THE PHYLLOSPHERE OF WHEAT. *MICROBIAL ECOLOGY*, *23*(1), 41–52. <https://doi.org/10.1007/BF00165906>

Dillard, H. R., Wicks, T. J., & Philp, B. (1993). A GROWER SURVEY OF DISEASES, INVERTEBRATE PESTS, AND PESTICIDE USE ON POTATOES GROWN IN SOUTH-AUSTRALIA. *AUSTRALIAN JOURNAL OF EXPERIMENTAL AGRICULTURE*, *33*(5), 653–661. <https://doi.org/10.1071/EA9930653>

Ding, X. Y., Zhang, H. M., Li, M., Yin, Z. Y., Chu, Z. H., Zhao, X. Y., Li, Y., & Ding, X. H. (2021). AtMYB12-ExpressingTransgenic Tobacco Increases Resistance to Several Phytopathogens and Aphids. *FRONTIERS IN AGRONOMY*, *3*. <https://doi.org/10.3389/fagro.2021.694333>

Dinu, M. M., Bloemhard, C. M. J., van Holstein-Saj, R., & Messelink, G. J. (2017). *Exploring opportunities to induce epizootics in greenhouse aphid populations* (WOS:000428256600047). *1164*, 371–375. <https://doi.org/10.17660/ActaHortic.2017.1164.47>

Dixit, S., Upadhyay, S. K., Singh, H., Sidhu, O. P., Verma, P. C., & Chandrashekar, K. (2013). Enhanced Methanol Production in Plants Provides Broad Spectrum Insect Resistance. *PLOS ONE*, *8*(11). <https://doi.org/10.1371/journal.pone.0079664>

Domingues, A., & Ornelas, L. (2022). *Periods of risk for the occurrence of the most common diseases and pests in Proteaceae in the Azores islands* (J. A. Rodríguez-Pérez & E. W. Hoffman, Eds.; Vol. 1347, pp. 137–142). International Society for Horticultural Science; Scopus. <https://doi.org/10.17660/ActaHortic.2022.1347.20>

Dorschner, K. W., Feng, M. G., & Baird, C. R. (1991). Virulence of an aphid-derived isolate of Beauveria bassiana (Fungi: Hyphomycetes) to the hop aphid, Phorodon humuli (Homoptera: Aphididae). *Environmental Entomology*, *20*(6), 690–693. Scopus. <https://doi.org/10.1093/ee/20.2.690>

Draghici, I., Draghici, R., & Croitoru, M. (2014). RESEARCHES ON THE EFFECT OF PHYTOSANITARY TREATMENTS TO GRAIN SORGHUM IN SANDY SOIL CONDITIONS. *SCIENTIFIC PAPERS-SERIES A-AGRONOMY*, *57*, 163–167.

Draghici, R., Draghici, I., & Dima, M. (2014). VULNERABILITY TO CONTAMINATION WITH PLANT PATHOGENS AND PESTS IN SPECIFIC CROP ROTATIONS OF AGROFORESTRY HOLDINGS ON SANDY SOILS. *SCIENTIFIC PAPERS-SERIES A-AGRONOMY*, *57*, 168–173.

Drakulic, J., Ajigboye, O., Swarup, R., Bruce, T., & Ray, R. V. (2016). Aphid Infestation Increases Fusarium langsethiae and T-2 and HT-2 Mycotoxins in Wheat. *APPLIED AND ENVIRONMENTAL MICROBIOLOGY*, *82*(22), 6548–6556. <https://doi.org/10.1128/AEM.02343-16>

Drakulic, J., Bruce, T. J. A., & Ray, R. V. (2017). Direct and host-mediated interactions between Fusarium pathogens and herbivorous arthropods in cereals. *PLANT PATHOLOGY*, *66*(1), 3–13. <https://doi.org/10.1111/ppa.12546>

Drakulic, J., Caulfield, J., Woodcock, C., Jones, S. P. T., Linforth, R., Bruce, T. J. A., & Ray, R. V. (2015). Sharing a Host Plant (Wheat [Triticum aestivum]) Increases the Fitness of Fusarium graminearum and the Severity of Fusarium Head Blight but Reduces the Fitness of Grain Aphids (Sitobion avenae). *APPLIED AND ENVIRONMENTAL MICROBIOLOGY*, *81*(10), 3492–3501. <https://doi.org/10.1128/AEM.00226-15>

Drakulic, J., Kahar, M. H., Ajigboye, O., Bruce, T., & Ray, R. V. (2016). Contrasting Roles of Deoxynivalenol and Nivalenol in Host-Mediated Interactions between Fusarium graminearum and Sitobion avenae. *TOXINS*, *8*(12). <https://doi.org/10.3390/toxins8120353>

Drizou, F., Bruce, T. J. A., Ray, R. V., & Graham, N. S. (2018). Infestation by Myzus persicae Increases Susceptibility of Brassica napus cv. “Canard” to Rhizoctonia solani AG 2-1. *FRONTIERS IN PLANT SCIENCE*, *9*. <https://doi.org/10.3389/fpls.2018.01903>

Du, X., Li, Q. Z., Shang, J. L., Liu, J. G., Qian, B. D., Jing, Q., Dong, T. F., Fan, D. D., Wang, H. Y., Zhao, L. C., Lieff, S., & Davies, T. (2019). Detecting advanced stages of winter wheat yellow rust and aphid infection using RapidEye data in North China Plain. *GISCIENCE & REMOTE SENSING*, *56*(7), 1093–1113. <https://doi.org/10.1080/15481603.2019.1613804>

Duan, X., Pan, S., Fan, M., Chu, B., Ma, Z., Gao, F., & Zhao, Z. (2022). Cultivar Mixture Enhances Crop Yield by Decreasing Aphids. *Agronomy*, *12*(2). Scopus. <https://doi.org/10.3390/agronomy12020335>

Dubcovsky, J., Lukaszewski, A. J., Echaide, M., Antonelli, E. F., & Porter, D. R. (1998). Molecular characterization of two Triticum speltoides interstitial translocations carrying leaf rust and greenbug resistance genes. *Crop Science*, *38*(6), 1655–1660. Scopus. <https://doi.org/10.2135/cropsci1998.0011183X003800060040x>

Dungga, N. E., Syaiful, S. A., Alfiani, A., Amin, A. R., Dachlan, A., Sahur, A., Dermawan, R., Idris, A. I., & Iop. (2020). *Growth and production of chili (Capsicum annuum L.) on the application of Trichoderma sp. And Azolla liquid organic fertilizer* (WOS:000604461700119). *486*. <https://doi.org/10.1088/1755-1315/486/1/012119>

Dupuis, B., Nkuriyingoma, P., & Ballmer, T. (2024). Economic Impact of Potato Virus Y (PVY) in Europe. *POTATO RESEARCH*, *67*(1), 55–72. <https://doi.org/10.1007/s11540-023-09623-x>

Duso, C., Malagnini, V., Pozzebon, A., Castagnoli, M., Liguori, M., & Simoni, S. (2008). Comparative toxicity of botanical and reduced-risk insecticides to Mediterranean populations of Tetranychus urticae and Phytoseiulus persimilis (Acari Tetranychidae, Phytoseiidae). *BIOLOGICAL CONTROL*, *47*(1), 16–21. <https://doi.org/10.1016/j.biocontrol.2008.06.011>

Easton, H. S., & Cornege, E. (1984). GRASSLANDS ORANGA LUCERNE (MEDICAGO-SATIVA). *NEW ZEALAND JOURNAL OF EXPERIMENTAL AGRICULTURE*, *12*(4), 283–286.

Edwards, C. A., Huelsman, M. F., Yardim, E. N., Shuster, W. D., & Brit Crop Protect, C. (1996). *Cultural inputs into integrated crop management and minimizing losses of processing tomatoes to pests in the US* (WOS:A1996BG73G00101). 597–602.

Eichenseer, H., & Dahlman, D. L. (1992). ANTIBIOTIC AND DETERRENT QUALITIES OF ENDOPHYTE-INFECTED TALL FESCUE TO 2 APHID SPECIES (HOMOPTERA, APHIDIAE). *ENVIRONMENTAL ENTOMOLOGY*, *21*(5), 1046–1051. <https://doi.org/10.1093/ee/21.5.1046>

Eichenseer, H., Dahlman, D. L., & Bush, L. P. (1991). INFLUENCE OF ENDOPHYTE INFECTION, PLANT-AGE AND HARVEST INTERVAL ON RHOPALOSIPUM-PADI SURVIVAL AND ITS RELATION TO QUANTITY OF N-FORMYL AND N-ACETYL LOLINE IN TALL FESCUE. *ENTOMOLOGIA EXPERIMENTALIS ET APPLICATA*, *60*(1), 29–38. <https://doi.org/10.1111/j.1570-7458.1991.tb01519.x>

Eisenhauer, N., Hörsch, V., Moeser, J., & Scheu, S. (2010). Synergistic effects of microbial and animal decomposers on plant and herbivore performance. *BASIC AND APPLIED ECOLOGY*, *11*(1), 23–34. <https://doi.org/10.1016/j.baae.2009.11.001>

Ekbom, B. S., & Åhman, I. (1980). The fungus Verticillium fusisporum as an insect pathogen. *Journal of Invertebrate Pathology*, *36*(1), 136–138. Scopus. <https://doi.org/10.1016/0022-2011(80)90149-4>

Elayabalan, S., Subramaniam, S., & Selvarajan, R. (2015). Banana bunchy top disease (BBTD) symptom expression in banana and strategies for transgenic resistance: A review. *EMIRATES JOURNAL OF FOOD AND AGRICULTURE*, *27*(1), 55–74. <https://doi.org/10.9755/ejfa.v27il.19197>

Elbanhawy, A. A., Elsherbiny, E. A., Abd El-Mageed, A. E., & Abdel-Fattah, G. M. (2019). Potential of fungal metabolites as a biocontrol agent against cotton aphid, Aphis gossypii Glover and the possible mechanisms of action. *PESTICIDE BIOCHEMISTRY AND PHYSIOLOGY*, *159*, 34–40. <https://doi.org/10.1016/j.pestbp.2019.05.013>

Ellis, C., Karafyllidis, L., & Turner, J. G. (2002). Constitutive activation of jasmonate signaling in an Arabidopsis mutant correlates with enhanced resistance to Erysiphe cichoracearum, Pseudomonas syringae, and Myzus persicae. *MOLECULAR PLANT-MICROBE INTERACTIONS*, *15*(10), 1025–1030. <https://doi.org/10.1094/MPMI.2002.15.10.1025>

Ellsbury, M. M., Pratt, R. G., & Knight, W. E. (1985). EFFECTS OF SINGLE AND COMBINED INFECTION OF ARROWLEAF CLOVER WITH BEAN YELLOW MOSAIC-VIRUS AND A PHYTOPHTHORA SP ON REPRODUCTION AND COLONIZATION BY PEA APHIDS (HOMOPTERA, APHIDIDAE). *ENVIRONMENTAL ENTOMOLOGY*, *14*(3), 356–359. <https://doi.org/10.1093/ee/14.3.356>

Elmekabaty, M. R., Hussain, M. A., & Ansari, M. A. (2020). Evaluation of commercial and non-commercial strains of entomopathogenic fungi against large raspberry aphid Amphorophora idaei. *BioControl*, *65*(1), 91–99. Scopus. <https://doi.org/10.1007/s10526-019-09981-x>

Elsharkawy, M. M., & Mousa, K. M. (2015). Induction of systemic resistance against Papaya ring spot virus (PRSV) and its vector Myzus persicae by Penicillium simplicissimum GP17-2 and silica (Sio2) nanopowder. *INTERNATIONAL JOURNAL OF PEST MANAGEMENT*, *61*(4), 353–358. <https://doi.org/10.1080/09670874.2015.1070930>

Emaru, A., Nyaanga, J. G., & Saidi, M. (2024). Integrating Metarhizium anisopliae entomopathogenic fungi with border cropping reduces black bean aphids (Aphis fabae) damage and enhances yield and quality of French bean. *HELIYON*, *10*(13). <https://doi.org/10.1016/j.heliyon.2024.e33037>

Erban, T., Shcherbachenko, E., Talacko, P., & Harant, K. (2021). A single honey proteome dataset for identifying adulteration by foreign amylases and mining various protein markers natural to honey. *JOURNAL OF PROTEOMICS*, *239*. <https://doi.org/10.1016/j.jprot.2021.104157>

Erdos, Z., Chandler, D., Bass, C., & Raymond, B. (2021). Controlling insecticide resistant clones of the aphid, Myzus persicae, using the entomopathogenic fungus Akanthomyces muscarius: Fitness cost of resistance under pathogen challenge. *Pest Management Science*, *77*(11), 5286–5293. Scopus. <https://doi.org/10.1002/ps.6571>

Erdos, Z., Studholme, D. J., Sharma, M. D., Chandler, D., Bass, C., & Raymond, B. (2024). Manipulating multi-level selection in a fungal entomopathogen reveals social conflicts and a method for improving biocontrol traits. *PLoS Pathogens*, *20*(3 March), 1–26. Scopus. <https://doi.org/10.1371/journal.ppat.1011775>

Erol, A. B., Abdelaziz, O., Birgücü, A. K., Senoussi, M. M., Oufroukh, A., & Karaca, I. (2020). Effects of some entomopathogenic fungi on the aphid species, Aphis gossypii Glover (Hemiptera: Aphididae). *EGYPTIAN JOURNAL OF BIOLOGICAL PEST CONTROL*, *30*(1). <https://doi.org/10.1186/s41938-020-00311-3>

Erol, A. B., & Erdogan, O. (2020). EFFECTS OF SOME BIOINSECTICIDES ON MACROSIPHUM ROSAE (L.) AND MYZUS PERSICAE (SULZER) (HEMIPTERA: APHIDIDAE). *FRESENIUS ENVIRONMENTAL BULLETIN*, *29*(12A), 11311–11316.

Evidente, A., Andolfi, A., Cimmino, A., Ganassi, S., Altomare, C., Favilla, M., De Cristofaro, A., Vitagliano, S., & Agnese Sabatini, M. (2009). Bisorbicillinoids produced by the fungus trichoderma citrinoviride affect feeding preference of the aphid schizaphis graminum. *Journal of Chemical Ecology*, *35*(5), 533–541. Scopus. <https://doi.org/10.1007/s10886-009-9632-6>

Evidente, A., Ricciardiello, G., Andolfi, A., Sabatini, M. A., Ganassi, S., Altomare, C., Favilla, M., & Melck, D. (2008). Citrantifidiene and citrantifidiol: Bioactive metabolites produced by Trichoderma citrinoviride with potential antifeedant activity toward aphids. *JOURNAL OF AGRICULTURAL AND FOOD CHEMISTRY*, *56*(10), 3569–3573. <https://doi.org/10.1021/jf073541h>

Fahmy, B. F. G., Abou Ghadir, N. M. F., Manaa, S. H., & Abou Ghadir, M. F. (2015). Occurrence of Entomopathogenic Fungi in Grain Aphids in Upper Egypt, with Reference to Certain Pathogenic Tests Using Scanning Electron Microscope. *EGYPTIAN JOURNAL OF BIOLOGICAL PEST CONTROL*, *25*(1), 177–181.

Fallah, N., Pang, Z., Zhang, C., Tayyab, M., Yang, Z., Lin, Z., Lin, W., Ishimwe, C., Ntambo, M. S., & Zhang, H. (2023). Complementary effects of biochar, secondary metabolites, and bacteria biocontrol agents rejuvenate ratoon sugarcane traits and stimulate soil fertility. *Industrial Crops and Products*, *202*. Scopus. <https://doi.org/10.1016/j.indcrop.2023.117081>

Fang, W. G., Leng, B., Xiao, Y. H., Jin, K., Ma, J. C., Fan, Y. H., Feng, J., Yang, X. Y., Zhang, Y. J., & Pei, Y. (2005). Cloning of Beauveria bassiana chitinase gene Bbchit1 and its application to improve fungal strain virulence. *APPLIED AND ENVIRONMENTAL MICROBIOLOGY*, *71*(1), 363–370. <https://doi.org/10.1128/AEM.71.1.363-370.2005>

Farag, N. A. (2008). Impact of entomopathogenic fungi on the aphid, brevicoryne brassicae L. and its associated predator, coccinella undecimpunctata L. *Egyptian Journal of Biological Pest Control*, *18*(2), 297–301. Scopus.

Farzadfar, S., Ahoonmanesh, A., Mosahebi, G. H., Ohshima, K., Koohi-Habibi, M., Pourrahim, R., & Golnaraghi, A. R. (2007). Partial biological and molecular characterization of Cauliflower mosaic virus isolates in Iran. *Plant Pathology Journal*, *6*(4), 291–298. Scopus. <https://doi.org/10.3923/ppj.2007.291.298>

Farzadfar, S., Ahoonmanesh, A., Mosahebi, G. H., Pourrahim, R., & Golnaraghi, A. R. (2007). Occurrence and distribution of Cauliflower mosaic virus on cruciferous plants in Iran. *Plant Pathology Journal*, *6*(1), 22–29. Scopus. <https://doi.org/10.3923/ppj.2007.22.29>

Faticov, M., Ekholm, A., Roslin, T., & Tack, A. J. M. (2020). Climate and host genotype jointly shape tree phenology, disease levels and insect attacks. *OIKOS*, *129*(3), 391–401. <https://doi.org/10.1111/oik.06707>

Fatope, M. O., Marwah, R. G., Onifade, A. K., Ochei, J. E., & Al Mahroqi, Y. K. S. (2006). 13C NMR analysis and antifungal and insecticidal activities of Oman dill herb oil. *PHARMACEUTICAL BIOLOGY*, *44*(1), 44–49. <https://doi.org/10.1080/13880200500530716>

Fazio, G., Robinson, T. L., & Aldwinckle, H. S. (2015). The Geneva Apple Rootstock Breeding Program. In J. Janick (Ed.), *PLANT BREEDING REVIEWS, VOL 39* (WOS:000385573900008; Vol. 39, pp. 379–424). <https://doi.org/10.1002/9781119107743>

Feng, M. G., & Chen, C. (2002). Incidence of infected Myzus persicae alatae trapped in flight imply place-to-place dissemination of entomophthoralean fungi in aphid populations through migration. *Journal of Invertebrate Pathology*, *81*(1), 53–56. Scopus. <https://doi.org/10.1016/S0022-2011(02)00114-3>

Feng, M. G., & Hua, L. (2005). Factors affecting the sporulation capacity during long-term storage of the aphid-pathogenic fungus Pandora neoaphidis grown on broomcorn millet. *FEMS Microbiology Letters*, *245*(2), 205–211. Scopus. <https://doi.org/10.1016/j.femsle.2005.03.015>

Feng, M. G., & Johnson, J. B. (1990). RELATIVE VIRULENCE OF 6 ISOLATES OF BEAUVERIA-BASSIANA ON DIURAPHIS-NOXIA (HOMOPTERA, APHIDIDAE). *ENVIRONMENTAL ENTOMOLOGY*, *19*(3), 785–790. <https://doi.org/10.1093/ee/19.3.785>

Feng, M. G., & Johnson, J. B. (1991). Bioassay of four entomophthoralean fungi (Entomophthorales) against Diuraphis noxia and Metopolophium dirhodum (Homoptera: Aphididae). *Environmental Entomology*, *20*(6), 338–345. Scopus. <https://doi.org/10.1093/ee/20.1.338>

Feng, M. G., Johnson, J. B., & Halbert, S. E. (1991). Natural control of cereal aphids (Homoptera: Aphididae) by entomopathogenic fungi (Zygomycetes: Entomophthorales) and parasitoids (Hymenoptera: Braconidae and encyrtidae) on irrigated spring wheat in Southwestern Idaho. *Environmental Entomology*, *20*(6), 1699–1710. Scopus. <https://doi.org/10.1093/ee/20.6.1699>

Feng, M. G., Johnson, J. B., & Kish, L. P. (1990a). Survey of entomopathogenic fungi naturally infecting cereal aphids (Homoptera: Aphididae) of irrigated grain crops in Southwestern Idaho. *Environmental Entomology*, *19*(6), 1534–1542. Scopus. <https://doi.org/10.1093/ee/19.5.1534>

Feng, M. G., Johnson, J. B., & Kish, L. P. (1990b). Virulence of verticillium lecanii and an aphid-derived isolate of beauveria bassiana (Fungi: Hyphomycetes) for Six Species of Cereal-Infesting Aphids (Homoptera: Aphididae). *Environmental Entomology*, *19*(6), 815–820. Scopus. <https://doi.org/10.1093/ee/19.3.815>

Feng, M. G., & Nowierski, R. M. (1992). Spatial patterns and sampling plans for cereal aphids [Hom.: Aphididae] killed by entomophthoralean fungi and hymenopterous parasitoids in spring wheat. *Entomophaga*, *37*(2), 265–275. Scopus. <https://doi.org/10.1007/BF02372427>

Fenner, E. D., Scapini, T., da Costa Diniz, M., Giehl, A., Treichel, H., Álvarez-Pérez, S., & Alves, S. L., Jr. (2022). Nature’s Most Fruitful Threesome: The Relationship between Yeasts, Insects, and Angiosperms. *J Fungi (Basel)*, *8*(10). <https://doi.org/10.3390/jof8100984>

Fergany, M., Kaur, B., Monforte, A. J., Pitrat, M., Rys, C., Lecoq, H., Dhillon, N. P. S., & Dhaliwal, S. S. (2011). Variation in melon (Cucumis melo) landraces adapted to the humid tropics of southern India. *Genetic Resources and Crop Evolution*, *58*(2), 225–243. Scopus. <https://doi.org/10.1007/s10722-010-9564-6>

Fernández-Grandon, G. M., Harte, S. J., Ewany, J., Bray, D., & Stevenson, P. C. (2020). Additive Effect of Botanical Insecticide and Entomopathogenic Fungi on Pest Mortality and the Behavioral Response of Its Natural Enemy. *PLANTS-BASEL*, *9*(2). <https://doi.org/10.3390/plants9020173>

Ferreira, J. M., Fernandes, É. K. K., Kim, J. S., & Soares, F. E. F. (2024). The Combination of Enzymes and Conidia of Entomopathogenic Fungi against Aphis gossypii Nymphs and Spodoptera frugiperda Larvae. *Journal of Fungi*, *10*(4). Scopus. <https://doi.org/10.3390/jof10040292>

Fideghelli, C. (2002). *The Italian national peach breeding project* (WOS:000181492600008). 73–79. <https://doi.org/10.17660/ActaHortic.2002.592.8>

Fiedler, Z., & Sosnowska, D. (2007). Nematophagous fungus Paecilomyces lilacinus (Thom) Samson is also a biological agent for control of greenhouse insects and mite pests. *BioControl*, *52*(4), 547–558. Scopus. <https://doi.org/10.1007/s10526-006-9052-2>

Filajdic, N., Sutton, T. B., Walgenbach, J. F., & Unrath, C. R. (1995). THE INFLUENCE OF THE APPLE APHID SPIREA APHID COMPLEX ON INTENSITY OF ALTERNARIA BLOTCH OF APPLE AND FRUIT-QUALITY CHARACTERISTICS AND YIELD. *PLANT DISEASE*, *79*(7), 691–694. <https://doi.org/10.1094/PD-79-0691>

Fingu-Mabola, J. C., Bawin, T., & Francis, F. (2021a). Direct and indirect effect via endophytism of entomopathogenic fungi on the fitness of myzus persicae and its ability to spread plrv on tobacco. *Insects*, *12*(2), 1–18. Scopus. <https://doi.org/10.3390/insects12020089>

Fingu-Mabola, J. C., Bawin, T., & Francis, F. (2021b). Direct and Indirect Effect via Endophytism of Entomopathogenic Fungi on the Fitness of Myzus persicae and Its Ability to Spread PLRV on Tobacco. *INSECTS*, *12*(2). <https://doi.org/10.3390/insects12020089>

Fingu-Mabola, J. C., Martin, C., Bawin, T., Verheggen, F. J., & Francis, F. (2020a). Does the infectious status of aphids influence their preference towards healthy, virus-infected and endophytically colonized plants? *Insects*, *11*(7), 1–16. Scopus. <https://doi.org/10.3390/insects11070435>

Fingu-Mabola, J. C., Martin, C., Bawin, T., Verheggen, F. J., & Francis, F. (2020b). Does the Infectious Status of Aphids Influence Their Preference Towards Healthy, Virus-Infected and Endophytically Colonized Plants? *INSECTS*, *11*(7). <https://doi.org/10.3390/insects11070435>

Finlayson, D. G. (1979). COMBINED EFFECTS OF SOIL-INCORPORATED AND FOLIAR-APPLIED INSECTICIDES IN BED-SYSTEM PRODUCTION OF BRASSICA CROPS. *CANADIAN JOURNAL OF PLANT SCIENCE*, *59*(2), 399–410. <https://doi.org/10.4141/cjps79-063>

Fitches, E., Philip, J., Hinchliffe, G., Vercruysse, L., Chougule, N., & Gatehouse, J. A. (2008). An evaluation of garlic lectin as an alternative carrier domain for insecticidal fusion proteins. *INSECT SCIENCE*, *15*(6), 483–495. <https://doi.org/10.1111/j.1744-7917.2008.00237.x>

Fitches, E., Wiles, D., Douglas, A. E., Hinchliffe, G., Audsley, N., & Gatehouse, J. A. (2008). The insecticidal activity of recombinant garlic lectins towards aphids. *INSECT BIOCHEMISTRY AND MOLECULAR BIOLOGY*, *38*(10), 905–915. <https://doi.org/10.1016/j.ibmb.2008.07.002>

Fitzgerald, J. (2004). Laboratory bioassays and field evaluation of insecticides for the control of Anthonomus rubi, Lygus rugulipennis and Chaetosiphon fragaefolii, and effects on beneficial species, in UK strawberry production. *CROP PROTECTION*, *23*(9), 801–809. <https://doi.org/10.1016/j.cropro.2003.12.005>

Fokkema, N. J., Riphagen, I., Poot, R. J., & Dejong, C. (1983). APHID HONEYDEW, A POTENTIAL STIMULANT OF COCHLIOBOLUS-SATIVUS AND SEPTORIA-NODORUM AND THE COMPETITIVE ROLE OF SAPROPHYTIC MYCOFLORA. *TRANSACTIONS OF THE BRITISH MYCOLOGICAL SOCIETY*, *81*(OCT), 355–363. <https://doi.org/10.1016/S0007-1536(83)80087-4>

Forlano, P., Mang, S. M., Caccavo, V., Fanti, P., Camele, I., Battaglia, D., & Trotta, V. (2022). Effects of Below-Ground Microbial Biostimulant Trichoderma harzianum on Diseases, Insect Community, and Plant Performance in Cucurbita pepo L. under Open Field Conditions. *MICROORGANISMS*, *10*(11). <https://doi.org/10.3390/microorganisms10112242>

Forslund, K., Pettersson, J., Bryngelsson, T., & Jonsson, L. (2000). Aphid infestation induces PR-proteins differently in barley susceptible or resistant to the birdcherry-oat aphid (Rhopalosiphum padi). *PHYSIOLOGIA PLANTARUM*, *110*(4), 496–502. <https://doi.org/10.1111/j.1399-3054.2000.1100411.x>

Fournier, V., & Brodeur, J. (2000). Dose-response susceptibility of pest aphids (Homoptera: Aphididae) and their control on hydroponically grown lettuce with the entomopathogenic fungus Verticillium lecanii, azadirachtin, and insecticidal soap. *ENVIRONMENTAL ENTOMOLOGY*, *29*(3), 568–578. <https://doi.org/10.1603/0046-225X-29.3.568>

Francis, F., Jaber, K., Colinet, F., Portetelle, D., & Haubruge, E. (2011). Purification of a new fungal mannose-specific lectin from Penicillium chrysogenum and its aphicidal properties. *FUNGAL BIOLOGY*, *115*(11), 1093–1099. <https://doi.org/10.1016/j.funbio.2011.06.010>

Fránová, J., Pribylová, J., & Koloniuk, I. (2019). Molecular and biological characterization of a new strawberry cytorhabdovirus. *Viruses*, *11*(11). Scopus. <https://doi.org/10.3390/v11110982>

Freimoser, F. M., Jensen, A. B., Tuor, U., Aebi, M., & Eilenberg, J. (2001). Isolation and in vitro cultivation of the aphid pathogenic fungus Entomophthora planchoniana. *Canadian Journal of Microbiology*, *47*(12), 1082–1087. Scopus. <https://doi.org/10.1139/cjm-47-12-1082>

French, N., Collingwood, C. A., Saynor, M., & Brock, A. M. (1981). EFFECTS OF PESTICIDE TREATMENTS ON THE YIELD OF SWEDES. *ANNALS OF APPLIED BIOLOGY*, *98*(1), 53–63. <https://doi.org/10.1111/j.1744-7348.1981.tb00422.x>

Frewin, A. J., Schaafsma, A. W., & Hallett, R. H. (2012). Susceptibility of Aphelinus certus to foliar-applied insecticides currently or potentially registered for soybean aphid control. *PEST MANAGEMENT SCIENCE*, *68*(2), 202–208. <https://doi.org/10.1002/ps.2245>

Fricaux, T., Le Navenant, A., Siegwart, M., Rault, M., Coustau, C., & Le Goff, G. (2023). The Molecular Resistance Mechanisms of European Earwigs from Apple Orchards Subjected to Different Management Strategies. *INSECTS*, *14*(12). <https://doi.org/10.3390/insects14120944>

Fuchs, B., Breuer, T., Findling, S., Krischke, M., Mueller, M. J., Holzschuh, A., & Krauss, J. (2017). Enhanced aphid abundance in spring desynchronizes predator–prey and plant–microorganism interactions. *Oecologia*, *183*(2), 469–478. Scopus. <https://doi.org/10.1007/s00442-016-3768-1>

Fuchs, B., & Krauss, J. (2019). Can Epichloe endophytes enhance direct and indirect plant defence? *FUNGAL ECOLOGY*, *38*, 98–103. <https://doi.org/10.1016/j.funeco.2018.07.002>

Fuchs, B., Krischke, M., Mueller, M. J., & Krauss, J. (2017). Herbivore-specific induction of defence metabolites in a grass-endophyte association. *FUNCTIONAL ECOLOGY*, *31*(2), 318–324. <https://doi.org/10.1111/1365-2435.12755>

Fuchs, B., Kuhnert, E., & Krauss, J. (2020). Contrasting Effects of Grass-Endophyte Chemotypes on a Tri-Trophic Cascade. *JOURNAL OF CHEMICAL ECOLOGY*, *46*(4), 422–429. <https://doi.org/10.1007/s10886-020-01163-9>

Fuentes-Contreras, E., & Niemeyer, H. M. (2002). Direct and indirect effects of wheat cultivars with different levels of resistance on parasitoids and entomopathogenic fungi of cereal aphids. *Ecoscience*, *9*(11), 37–43. Scopus. <https://doi.org/10.1080/11956860.2002.11682688>

Gafni, A., Calderon, C. E., Harris, R., Buxdorf, K., Dafa-Berger, A., Zeilinger-Reichert, E., & Levy, M. (2015). Biological control of the cucurbit powdery mildew pathogen Podosphaera xanthii by means of the epiphytic fungus Pseudozyma aphidis and parasitism as a mode of action. *Frontiers in Plant Science*, *6*(MAR). Scopus. <https://doi.org/10.3389/fpls.2015.00132>

Gai, Y. P., Zhao, Y. N., Zhao, H. N., Yuan, C. Z., Yuan, S. S., Li, S., Zhu, B. S., & Ji, X. L. (2017). The Latex Protein MLX56 from Mulberry (Morus multicaulis) Protects Plants against Insect Pests and Pathogens. *FRONTIERS IN PLANT SCIENCE*, *8*. <https://doi.org/10.3389/fpls.2017.01475>

Galimberti, A., & Alyokhin, A. (2018). Lethal and Sublethal Effects of Mineral Oil on Potato Pests. *JOURNAL OF ECONOMIC ENTOMOLOGY*, *111*(3), 1261–1267. <https://doi.org/10.1093/jee/toy046>

Ganassi, S., De Cristofaro, A., Grazioso, P., Altomare, C., Logrieco, A., & Sabatini, M. A. (2007). Detection of fungal metabolites of various Trichoderma species by the aphid Schizaphis graminum. *ENTOMOLOGIA EXPERIMENTALIS ET APPLICATA*, *122*(1), 77–86. <https://doi.org/10.1111/j.1570-7458.2006.00494.x>

Ganassi, S., Grazioso, P., De Cristofaro, A., Fiorentini, F., Sabatini, M. A., Evidente, A., & Altomare, C. (2016). Long Chain Alcohols Produced by Trichoderma citrinoviride Have Phagodeterrent Activity against the Bird Cherry-Oat Aphid Rhopalosiphum padi. *FRONTIERS IN MICROBIOLOGY*, *7*. <https://doi.org/10.3389/fmicb.2016.00297>

Ganassi, S., Grazioso, P., Moretti, A., & Sabatini, M. A. (2010). Effects of the fungus Lecanicillium lecanii on survival and reproduction of the aphid Schizaphis graminum. *BioControl*, *55*(2), 299–312. Scopus. <https://doi.org/10.1007/s10526-009-9250-9>

Ganassi, S., Moretti, A., Logrieco, A., Bonvicini, P. A. M., & Sabatini, M. A. (2004). *A laboratory study on colonisation of aphids by some filamentous fungi* (WOS:000189426400045). 299–306.

Ganassi, S., Moretti, A., Stornelli, C., Fratello, B., Pagliai, A. M. B., Logrieco, A., & Sabatini, M. A. (2001). Effect of Fusarium, Paecilomyces and Trichoderma formulations against aphid Schizaphis graminum. *MYCOPATHOLOGIA*, *151*(3), 131–138. <https://doi.org/10.1023/A:1017940604692>

García-Gutiérrez, C., & González-Maldonado, M. B. (2010). USE OF BIOINSECTICIDES FOR VEGETABLE PEST CONTROL IN POUR COMMUNITIES. *REVISTA RA XIMHAI*, *6*(1), 17–22.

Geedi, R., Canas, L., Reding, M. E., & Ranger, C. M. (2023). Attraction of Myzus persicae (Hemiptera: Aphididae) to Volatiles Emitted From the Entomopathogenic Fungus Beauveria bassiana. *Environmental Entomology*, *52*(1), 31–38. Scopus. <https://doi.org/10.1093/ee/nvac100>

Ghazanfar, M. U., Raza, W., Wakil, W., Hussain, I., & Qamar, M. I. (2020). Management of late blight and sucking insect pests of potato with application of salicylic acid and β-aminobutyric acid under greenhouse conditions. *Sarhad Journal of Agriculture*, *36*(2), 646–654. Scopus. <https://doi.org/10.17582/JOURNAL.SJA/2020/36.2.646.654>

Ghosh, S. K., Chakraborty, N., & Biswas, P. P. (2014a). *In Vitro Biological Control of Aphid of Papaya by Beauveria bassiana* (WOS:000343860500014). *1022*, 113–117.

Ghosh, S. K., Chakraborty, N., & Biswas, P. P. (2014b). *In vitro biological control of aphid of papaya by beauveria bassiana*. *1022*, 113–118. Scopus. <https://doi.org/10.17660/actahortic.2014.1022.14>

Gindin, G., Barash, I., Harari, N., & Raccah, B. (1994). Effect of endotoxic compounds isolated from Verticillium lecanii on the sweetpotato whitefly, Bemisia tabaci. *Phytoparasitica*, *22*(3), 189–196. Scopus. <https://doi.org/10.1007/BF02980318>

Gindin, G., Geschtovt, N. U., Raccah, B., & Barash, I. (2000). Pathogenicity of Verticillium lecanii to different developmental stages of the silverleaf whitefly, Bemisia argentifolii. *PHYTOPARASITICA*, *28*(3), 229–239. <https://doi.org/10.1007/BF02981801>

Giordano, L. D., Boiteux, L. S., Quezado-Duval, A. M., Fonseca, M. E. D., Resende, F. V., Reis, A., González, M., Marcos, W., & Mendonça, N. J. L. (2010). “BRS Tospodoro”: A high lycopene processing tomato cultivar adapted to organic cropping systems and with multiple resistance to pathogens. *HORTICULTURA BRASILEIRA*, *28*(2), 241–245. <https://doi.org/10.1590/S0102-05362010000200019>

Gliszczyńska, A., Gładkowski, W., Dancewicz, K., & Gabryś, B. (2015). Enantioselective Microbial Hydroxylation as a Useful Tool in the Production of Jasmonate Derivatives with Aphid Deterrent Activity. *Current Microbiology*, *71*(1), 83–94. Scopus. <https://doi.org/10.1007/s00284-015-0831-9>

Gliszczyńska, A., Gładkowski, W., Dancewicz, K., Gabryś, B., & Szczepanik, M. (2016). Transformation of β-damascone to (+)-(S)-4-hydroxy-β-damascone by fungal strains and its evaluation as a potential insecticide against aphids Myzus persicae and lesser mealworm Alphitobius diaperinus Panzer. *Catalysis Communications*, *80*, 39–43. Scopus. <https://doi.org/10.1016/j.catcom.2016.03.018>

Gliszczynska, A., Semba, D., Szczepanik, M., Dancewicz, K., & Gabrys, B. (2016). Alkyl-Substituted -Lactones Derived from Dihydrojasmone and Their Stereoselective Fungi-Mediated Conversion: Production of New Antifeedant Agents. *MOLECULES*, *21*(9). <https://doi.org/10.3390/molecules21091226>

Godfrey-Sam-Aggrey, W., & Balcha, A. (1988). Insect fauna in a declining citrus orchard at Melka Werer in Ethiopia. *FAO Plant Protection Bulletin*, *36*(2), 75–81. Scopus.

Goettel, M. S., Koike, M., Kim, J. J., Aiuchi, D., Shinya, R., & Brodeur, J. (2008). Potential of Lecanicillium spp. For management of insects, nematodes and plant diseases. *JOURNAL OF INVERTEBRATE PATHOLOGY*, *98*(3), 256–261. <https://doi.org/10.1016/j.jip.2008.01.009>

Gomez, S. K., Maurya, A. K., Irvin, L., Kelly, M. P., Schoenherr, A. P., Huguet-Tapia, J. C., & Bombarely, A. (2023). A snapshot of the transcriptome of Medicago truncatula (Fabales: Fabaceae) shoots and roots in response to an arbuscular mycorrhizal fungus and the pea aphid (Acyrthosiphon pisum) (Hemiptera: Aphididae). *Environmental Entomology*, *52*(4), 667–680. Scopus. <https://doi.org/10.1093/ee/nvad070>

Gonthier, D. J., Sullivan, T. J., Brown, K. L., Wurtzel, B., Lawal, R., VandenOever, K., Buchan, Z., & Bultman, T. L. (2008). Stroma-forming endophyte Epichloe glyceriae provides wound-inducible herbivore resistance to its grass host. *OIKOS*, *117*(4), 629–633. <https://doi.org/10.1111/j.0030-1299.2008.16483.x>

González-Coloma, A., Sainz, P., Andrés, M. F., Martínez-Díaz, R. A., Bailén, M., Navarro-Rocha, J., & Díaz, C. E. (2019). Chemical composition and biological activities of artemisia pedemontana subsp. Assoana essential oils and hydrolate. *Biomolecules*, *9*(10). Scopus. <https://doi.org/10.3390/biom9100558>

González-Mas, N., Cuenca-Medina, M., Gutiérrez-Sánchez, F., & Quesada-Moraga, E. (2019). Bottom-up effects of endophytic Beauveria bassiana on multitrophic interactions between the cotton aphid, Aphis gossypii, and its natural enemies in melon. *JOURNAL OF PEST SCIENCE*, *92*(3), 1271–1281. <https://doi.org/10.1007/s10340-019-01098-5>

González‐Mas, N., Cuenca‐Medina, M., Gutiérrez‐Sánchez, F., & Quesada‐Moraga, E. (2020). Correction to: Bottom‐up effects of endophytic Beauveria bassiana on multitrophic interactions between the cotton aphid, Aphis gossypii, and its natural enemies in melon (Journal of Pest Science, (2019), 92, 3, (1271-1281), 10.1007/s10340-019-01098-5). *Journal of Pest Science*, *93*(1), 553. Scopus. <https://doi.org/10.1007/s10340-019-01152-2>

González-Mas, N., Gutiérrez-Sánchez, F., Sánchez-Ortiz, A., Grandi, L., Turlings, T. C. J., Muñoz-Redondo, J. M., Moreno-Rojas, J. M., & Quesada-Moraga, E. (2021). Endophytic Colonization by the Entomopathogenic Fungus Beauveria Bassiana Affects Plant Volatile Emissions in the Presence or Absence of Chewing and Sap-Sucking Insects. *FRONTIERS IN PLANT SCIENCE*, *12*. <https://doi.org/10.3389/fpls.2021.660460>

González-Mas, N., Quesada-Moraga, E., Plaza, M., Fereres, A., & Moreno, A. (2019). Changes in feeding behaviour are not related to the reduction in the transmission rate of plant viruses by Aphis gossypii (Homoptera: Aphididae) to melon plants colonized by Beauveria bassiana (Ascomycota: Hypocreales). *BIOLOGICAL CONTROL*, *130*, 95–103. <https://doi.org/10.1016/j.biocontrol.2018.11.001>

González-Mas, N., Sánchez-Ortiz, A., Valverde-García, P., & Quesada-Moraga, E. (2019). Effects of Endophytic Entomopathogenic Ascomycetes on the Life-History Traits of Aphis gossypii Glover and Its Interactions with Melon Plants. *INSECTS*, *10*(6). <https://doi.org/10.3390/insects10060165>

Goto, K., Horikoshi, R., Mitomi, M., Oyama, K., Hirose, T., Sunazuka, T., & Ōmura, S. (2018). Synthesis and insecticidal efficacy of pyripyropene derivatives focusing on the C-1, C-7, and C-11 positions’ substituent groups. *Journal of Antibiotics*, *71*(9), 785–797. Scopus. <https://doi.org/10.1038/s41429-018-0064-9>

Grabarczyk, M., Winska, K., Maczka, W., Zarowska, B., Maciejewska, G., Dancewicz, K., Gabrys, B., & Aniol, M. (2016). Synthesis, biotransformation and biological activity of halolactones obtained from β-ionone. *TETRAHEDRON*, *72*(5), 637–644. <https://doi.org/10.1016/j.tet.2015.12.005>

Grabmaier, A., Heigl, F., Eisenhauer, N., van der Heijden, M. G. A., & Zaller, J. G. (2014). Stable isotope labelling of earthworms can help deciphering belowground-aboveground interactions involving earthworms, mycorrhizal fungi, plants and aphids. *Pedobiologia*, *57*(4–6), 197–203. Scopus. <https://doi.org/10.1016/j.pedobi.2014.10.002>

Gray, S. N., Robinson, P., Wilding, N., & Markham, P. (1990). Effect of oleic acid on vegetative growth of the aphid-pathogenic fungus Erynia neoaphidis. *FEMS Microbiology Letters*, *68*(1–2), 131–136. Scopus. <https://doi.org/10.1016/0378-1097(90)90138-G>

Gray, S. N., Wilding, N., & Markham, P. (1991). In vitro germination of single conidia of the aphid-pathogenic fungus Erynia neoaphidis and phenotypic variation among sibling strains. *FEMS Microbiology Letters*, *79*(2–3), 273–278. Scopus. <https://doi.org/10.1111/j.1574-6968.1991.tb04541.x>

Greaves, D. A., Hooper, A. J., & Walpole, B. J. (1983). IDENTIFICATION OF BARLEY YELLOW DWARF VIRUS AND CEREAL APHID INFESTATIONS IN WINTER-WHEAT BY AERIAL-PHOTOGRAPHY. *PLANT PATHOLOGY*, *32*(2), 159–172. <https://doi.org/10.1111/j.1365-3059.1983.tb01315.x>

Greer, S. F., Surendran, A., Grant, M., & Lillywhite, R. (2023). The current status, challenges, and future perspectives for managing diseases of brassicas. *Frontiers in Microbiology*, *14*. Scopus. <https://doi.org/10.3389/fmicb.2023.1209258>

Grell, M. N., Jensen, A. B., Olsen, P. B., Eilenberg, J., & Lange, L. (2011). Secretome of fungus-infected aphids documents high pathogen activity and weak host response. *Fungal Genetics and Biology*, *48*(4), 343–352. Scopus. <https://doi.org/10.1016/j.fgb.2010.12.003>

Gross, J., Just, J., & Wetzel, S. (2009). The antimicrobial defense of the invasive ladybird Harmonia axyridis in Central Europe compared to the native species Coccinella septempunctata. In J. Handel (Ed.), *MITTEILUNGEN DER DEUTSCHEN GESELLSCHAFT FUR ALLGEMEINE UND ANGEWANDTE ENTOMOLOGIE, BAND 17* (WOS:000277208800011; Vol. 17, pp. 61–65).

Guenthner, J. F., Wiese, M. V., Pavlista, A. D., Sieczka, J. B., & Wyman, J. (1999). Assessment of pesticide use in the US potato industry. *AMERICAN JOURNAL OF POTATO RESEARCH*, *76*(1), 25–29. <https://doi.org/10.1007/BF02853554>

Guesmi-Jouini, J., Garrido-Jurado, I., López-Díaz, C., Ben Halima-Kamel, M., & Quesada-Moraga, E. (2014). Establishment of fungal entomopathogens Beauveria bassiana and Bionectria ochroleuca (Ascomycota: Hypocreales) as endophytes on artichoke Cynara scolymus. *JOURNAL OF INVERTEBRATE PATHOLOGY*, *119*, 1–4. <https://doi.org/10.1016/j.jip.2014.03.004>

Guo, Z. L., Li, F., Cheng, F. S., & Song, Q. Y. (2020). *Insecticidal activity of the crude extract from Epichloë bromicola against Rhopalosiphum padi* (N. Hamid & S. Shiquan, Eds.; Vol. 189). EDP Sciences; Scopus. <https://doi.org/10.1051/e3sconf/202018902008>

Gupta, K., Mishra, S. K., Gupta, S., Pandey, S., Panigrahi, J., & Wani, S. H. (2021). Functional Role of miRNAs: Key Players in Soybean Improvement. *PHYTON-INTERNATIONAL JOURNAL OF EXPERIMENTAL BOTANY*, *90*(5), 1339–1362. <https://doi.org/10.32604/phyton.2021.015239>

Gurulingappa, P., Gee, P. M., & Sword, G. A. (2011). In Vitro and In Planta Compatibility of Insecticides and the Endophytic Entomopathogen, Lecanicillium lecanii. *Mycopathologia*, *172*(2), 161–168. Scopus. <https://doi.org/10.1007/s11046-011-9410-1>

Gurulingappa, P., McGee, P. A., & Sword, G. (2011). Endophytic Lecanicillium lecanii and Beauveria bassiana reduce the survival and fecundity of Aphis gossypii following contact with conidia and secondary metabolites. *CROP PROTECTION*, *30*(3), 349–353. <https://doi.org/10.1016/j.cropro.2010.11.017>

Gurulingappa, P., Sword, G. A., Murdoch, G., & McGee, P. A. (2010). Colonization of crop plants by fungal entomopathogens and their effects on two insect pests when in planta. *BIOLOGICAL CONTROL*, *55*(1), 34–41. <https://doi.org/10.1016/j.biocontrol.2010.06.011>

Haase, J., Brandl, R., Scheu, S., & Schädler, M. (2008). ABOVE- AND BELOWGROUND INTERACTIONS ARE MEDIATED BY NUTRIENT AVAILABILITY. *ECOLOGY*, *89*(11), 3072–3081. <https://doi.org/10.1890/07-1983.1>

Hadidi, A., Sun, L., & Randles, J. W. (2022). Modes of Viroid Transmission. *Cells*, *11*(4). Scopus. <https://doi.org/10.3390/cells11040719>

Hagley, E. A. C., & Allen, W. R. (1990). THE GREEN APPLE APHID, APHIS-POMI DEGEER (HOMOPTERA, APHIDIDAE), AS PREY OF POLYPHAGOUS ARTHROPOD PREDATORS IN ONTARIO. *CANADIAN ENTOMOLOGIST*, *122*(11–12), 1221–1228. <https://doi.org/10.4039/Ent1221221-11>

Hajyieva, H., & Soroka, S. (2008). Phytosanitary situation in sugar beet crops in Belarus. *ZEMDIRBYSTE-AGRICULTURE*, *95*(3), 65–73.

Halder, J., Majumder, S., & Rai, A. B. (2021). Compatibility and combined efficacy of entomopathogenic fungi and neonicotinoid insecticides against Myzus persicae (Sulzer): An ecofriendly approach. *Entomologia Hellenica*, *30*(1), 24–32. Scopus. <https://doi.org/10.12681/eh.25417>

Halder, J., Rai, A. B., & Kodandaram, M. H. (2013). Compatibility of Neem Oil and Different Entomopathogens for the Management of Major Vegetable Sucking Pests. *NATIONAL ACADEMY SCIENCE LETTERS-INDIA*, *36*(1), 19–25. <https://doi.org/10.1007/s40009-012-0091-1>

Hall, A. E., Cisse, N., Thiaw, S., Elawad, H. O. A., Ehlers, J. D., Ismail, A. M., Fery, R. L., Roberts, P. A., Kitch, L. W., Murdock, L. L., Boukar, O., Phillips, R. D., & McWatters, K. H. (2003). Development of cowpea cultivars and germplasm by the Bean/Cowpea CRSP. *FIELD CROPS RESEARCH*, *82*(2–3), 103–134. <https://doi.org/10.1016/S0378-4290(03)00033-9>

Hall, R. A. (1980). CONTROL OF APHIDS BY THE FUNGUS, VERTICILLIUM LECANII: EFFECT OF SPORE CONCENTRATION. *Entomologia Experimentalis et Applicata*, *27*(1), 1–5. Scopus. <https://doi.org/10.1111/j.1570-7458.1980.tb02939.x>

Hall, R. A. (1982). Control of whitefly, Trialeurodes vaporariorum and cotton aphid, Aphis gossypii in glasshouses by two isolates of the fungus, Verticillium lecanii. *Annals of Applied Biology*, *101*(1), 1–11. Scopus. <https://doi.org/10.1111/j.1744-7348.1982.tb00794.x>

Hall, R. A., & Burges, H. D. (1979). Control of aphids in glasshouses with the fungus, Verticillium lecanii. *Annals of Applied Biology*, *93*(3), 235–246. Scopus. <https://doi.org/10.1111/j.1744-7348.1979.tb06538.x>

Hamouche, Z., Zippari, C., Boucherf, A., Cavallo, G., Djelouah, K., Tamburini, G., Verrastro, V., Biondi, A., & Cornara, D. (2024). Impact of biopesticides on the probing and feeding behavior of Aphis gossypii. *CABI AGRICULTURE & BIOSCIENCE*, *5*(1). <https://doi.org/10.1186/s43170-024-00269-4>

Hamshou, M., Smagghe, G., Shahidi-Noghabi, S., De Geyter, E., Lannoo, N., & Van Damme, E. J. M. (2010). Insecticidal properties of Sclerotinia sclerotiorum agglutinin and its interaction with insect tissues and cells. *INSECT BIOCHEMISTRY AND MOLECULAR BIOLOGY*, *40*(12), 883–890. <https://doi.org/10.1016/j.ibmb.2010.08.008>

Hamshou, M., Van Damme, E. J. M., Caccia, S., Cappelle, K., Vandenborre, G., Ghesquière, B., Gevaert, K., & Smagghe, G. (2013). High entomotoxicity and mechanism of the fungal GalNAc/Gal-specific Rhizoctonia solani lectin in pest insects. *JOURNAL OF INSECT PHYSIOLOGY*, *59*(3), 295–305. <https://doi.org/10.1016/j.jinsphys.2012.12.003>

Han, C., Zhang, G., Mei, Y., Shan, Z., Shi, K., Zhou, S., & Shao, H. (2023). Chemical profile of Artemisia vulgaris L. essential oil and its phytotoxic, insecticidal, and antimicrobial activities. *South African Journal of Botany*, *162*, 20–28. Scopus. <https://doi.org/10.1016/j.sajb.2023.08.058>

Han, C., Zhou, S., Mei, Y., Cao, Q., Shi, K., & Shao, H. (2022). Phytotoxic, insecticidal, and antimicrobial activities of Ajania tibetica essential oil. *Frontiers in Plant Science*, *13*. Scopus. <https://doi.org/10.3389/fpls.2022.1028252>

Hanan, A., Basit, A., Nazir, T., Majeed, M. Z., & Qiu, D. (2020). Anti-insect activity of a partially purified protein derived from the entomopathogenic fungus Lecanicillium lecanii (Zimmermann) and its putative role in a tomato defense mechanism against green peach aphid. *Journal of Invertebrate Pathology*, *170*. Scopus. <https://doi.org/10.1016/j.jip.2019.107282>

Hanan, A., Nazir, T., Basit, A., Ahmad, S., & Qiu, D. (2020a). Potential of Lecanicillium lecanii (Zimm.) as a microbial control agent for green peach aphid, Myzus persicae (Sulzer) (Hemiptera: Aphididae). *Pakistan Journal of Zoology*, *52*(1), 131–137. Scopus. <https://doi.org/10.17582/journal.pjz/2020.52.1.131.137>

Hanan, A., Nazir, T., Basit, A., Ahmad, S., & Qiu, D. W. (2020b). Potential of Lecanicillium lecanii (Zimm.) as a Microbial Control Agent for Green Peach Aphid, Myzus persicae (Sulzer) (Hemiptera: Aphididae). *PAKISTAN JOURNAL OF ZOOLOGY*, *52*(1), 131–137. <https://doi.org/10.17582/journal.pjz/2020.52.1.1.131.137>

Hanel, A., Orpet, R. J., Hilton, R., Nottingham, L., Northfield, T. D., & Schmidt-Jeffris, R. (2023). Turning a Pest into a Natural Enemy: Removing Earwigs from Stone Fruit and Releasing Them in Pome Fruit Enhances Pest Control. *INSECTS*, *14*(12). <https://doi.org/10.3390/insects14120906>

Hao, Z. P., Feng, Z. B., Sheng, L., Fei, W. X., & Hou, S. M. (2024). Facilitation of Sclerotinia sclerotiorum infestation by aphid feeding behaviour is not affected by aphid resistance in oilseed rape. *Heliyon*, *10*(11). Scopus. <https://doi.org/10.1016/j.heliyon.2024.e32429>

Hao, Z. P., Sheng, L., Feng, Z. B., Fei, W. X., & Hou, S. M. (2024). Aphids May Facilitate the Spread of Sclerotinia Stem Rot in Oilseed Rape by Carrying and Depositing Ascospores. *Journal of Fungi*, *10*(3). Scopus. <https://doi.org/10.3390/jof10030202>

Hao, Z., Xie, W., & Chen, B. (2019). Arbuscular mycorrhizal symbiosis affects plant immunity to viral infection and accumulation. *Viruses*, *11*(6). Scopus. <https://doi.org/10.3390/v11060534>

Happe, A. K., Roquer-Beni, L., Bosch, J., Alins, G., & Mody, K. (2018). Earwigs and woolly apple aphids in integrated and organic apple orchards: Responses of a generalist predator and a pest prey to local and landscape factors. *AGRICULTURE ECOSYSTEMS & ENVIRONMENT*, *268*, 44–51. <https://doi.org/10.1016/j.agee.2018.09.004>

Hareendranath, V., Nair, K. P. V., & Paulose, S. (1987). FUSARIUM-PALLIDOROSEUM (COOKE) SACC AS A FUNGAL PATHOGEN OF APHIS-CRACCIVORA KOCH. *ENTOMON*, *12*(4), 392–394.

Harper, A. M., & Huang, H. C. (1986). EVALUATION OF THE ENTOMOPHAGOUS FUNGUS VERTICILLIUM-LECANII (MONILIALES, MONILIACEAE) AS A CONTROL AGENT FOR INSECTS. *ENVIRONMENTAL ENTOMOLOGY*, *15*(2), 281–284. <https://doi.org/10.1093/ee/15.2.281>

Harper, A. M., Huang, H. C., & Kozub, G. C. (1988). SURVIVAL OF VERTICILLIUM-ALBO-ATRUM ON INSECT BODIES AND IN INSECT FECES AT VARIOUS TEMPERATURES. *JOURNAL OF ECONOMIC ENTOMOLOGY*, *81*(6), 1799–1802. <https://doi.org/10.1093/jee/81.6.1799>

Härri, S. A., Krauss, J., & Müller, C. B. (2008). Natural enemies act faster than endophytic fungi in population control of cereal aphids. *Journal of Animal Ecology*, *77*(3), 605–611. Scopus. <https://doi.org/10.1111/j.1365-2656.2008.01373.x>

Harris-Shultz, K., Knoll, J., Punnuri, S., Niland, E., & Ni, X. Z. (2020). Evaluation of strains of Beauveria bassiana and Isaria fumosorosea to control sugarcane aphids on grain sorghum. *AGROSYSTEMS GEOSCIENCES & ENVIRONMENT*, *3*(1). <https://doi.org/10.1002/agg2.20047>

Hartfield, C. M., Campbell, C. A. M., Hardie, J., Pickett, J. A., & Wadhams, L. J. (2001). Pheromone traps for the dissemination of an entomopathogen by the Damson-hop aphid Phorodon humuli. *BIOCONTROL SCIENCE AND TECHNOLOGY*, *11*(3), 401–410. <https://doi.org/10.1080/09583150120055817>

Harun-Or-Rashid, M., Khan, A., Hossain, M. T., & Chung, Y. R. (2017). Induction of Systemic Resistance against Aphids by Endophytic Bacillus velezensis YC7010 via Expressing PHYTOALEXIN DEFICIENT4 in Arabidopsis. *FRONTIERS IN PLANT SCIENCE*, *8*. <https://doi.org/10.3389/fpls.2017.00211>

Harwood, J. D., Phillips, S. W., Lello, J., Sunderland, K. D., Glen, D. M., Bruford, M. W., Harper, G. L., & Symondson, W. O. C. (2009). Invertebrate biodiversity affects predator fitness and hence potential to control pests in crops. *BIOLOGICAL CONTROL*, *51*(3), 499–506. <https://doi.org/10.1016/j.biocontrol.2009.09.007>

Hassan, S. A. (1982). COMPARISON OF 3 DIFFERENT STRAINS OF PHYTOSEIULUS-PERSIMILIS TO CONTROL TETRANYCHUS-URTICAE ON CUCUMBER IN GLASSHOUSES. *ZEITSCHRIFT FUR ANGEWANDTE ENTOMOLOGIE-JOURNAL OF APPLIED ENTOMOLOGY*, *93*(2), 131–140.

Hatano, E., Baverstock, J., Kunert, G., Pell, J. K., & Weisser, W. W. (2012). Entomopathogenic fungi stimulate transgenerational wing induction in pea aphids, Acyrthosiphon pisum (Hemiptera: Aphididae). *Ecological Entomology*, *37*(1), 75–82. Scopus. <https://doi.org/10.1111/j.1365-2311.2011.01336.x>

Hatting, J. L., Humber, R. A., Poprawski, T. J., & Miller, R. M. (1999). A survey of fungal pathogens of aphids from South Africa, with special reference to cereal aphids. *BIOLOGICAL CONTROL*, *16*(1), 1–12. <https://doi.org/10.1006/bcon.1999.0731>

Hatting, J. L., Poprawski, T. J., & Miller, R. M. (2000). Prevalences of fungal pathogens and other natural enemies of cereal aphids (Homoptera: Aphididae) in wheat under dryland and irrigated conditions in South Africa. *BIOCONTROL*, *45*(2), 179–199. <https://doi.org/10.1023/A:1009981718582>

Hatting, J. L., Wraight, S. P., & Miller, R. M. (2004). Efficacy of Beauveria bassiana (Hyphomycetes) for control of Russian wheat aphid (Homoptera: Aphididae) on resistant wheat under field conditions. *BIOCONTROL SCIENCE AND TECHNOLOGY*, *14*(5), 459–473. <https://doi.org/10.1080/09583150410001683501>

Hatzipapas, P., Kalosaka, K., Dara, A., & Christias, C. (2002). Spore germination and appressorium formation in the entomopathogenic Alternaria alternata. *MYCOLOGICAL RESEARCH*, *106*, 1349–1359. <https://doi.org/10.1017/S0953756202006792>

Hayden, T. P., Bidochka, M. J., & Khachatourians, G. G. (1992). ENTOMOPATHOGENICITY OF SEVERAL FUNGI TOWARD THE ENGLISH GRAIN APHID (HOMOPTERA, APHIDIDAE) AND ENHANCEMENT OF VIRULENCE WITH HOST PASSAGE OF PAECILOMYCES-FARINOSUS. *JOURNAL OF ECONOMIC ENTOMOLOGY*, *85*(1), 58–64. <https://doi.org/10.1093/jee/85.1.58>

He, Y. L., Chen, T. X., Zhang, H. J., White, J. F., & Li, C. J. (2022). Fungal Endophytes Help Grasses to Tolerate Sap-Sucking Herbivores Through a Hormone-Signaling System. *JOURNAL OF PLANT GROWTH REGULATION*, *41*(6), 2122–2137. <https://doi.org/10.1007/s00344-021-10430-2>

Heiska, S., Tikkanen, O. P., Rousi, M., Turtola, S., Tirkkonen, V., Meier, B., & Julkunen-Tiitto, R. (2007). The susceptibility of herbal willow to Melampsora rust and herbivores. *EUROPEAN JOURNAL OF PLANT PATHOLOGY*, *118*(3), 275–285. <https://doi.org/10.1007/s10658-007-9145-5>

Helfenstein, J., Pawlowski, M. L., Hill, C. B., Stewart, J., Lagos-Kutz, D., Bowen, C. R., Frossard, E., & Hartman, G. L. (2015). Zinc deficiency alters soybean susceptibility to pathogens and pests. *JOURNAL OF PLANT NUTRITION AND SOIL SCIENCE*, *178*(6), 896–903. <https://doi.org/10.1002/jpln.201500146>

Helyer, N., Gill, G., Bywater, A., & Chambers, R. (1992). ELEVATED HUMIDITIES FOR CONTROL OF CHRYSANTHEMUM PESTS WITH VERTICILLIUM-LECANII. *PESTICIDE SCIENCE*, *36*(4), 373–378. <https://doi.org/10.1002/ps.2780360412>

Henderson, G., Holland, P. G., & Werren, G. L. (1979). THE NATURAL-HISTORY OF A SUB-ARCTIC ADVENTIVE - EPILOBIUM-ANGUSTIFOLIUM L (ONAGRACEAE) AT SCHEFFERVILLE, QUEBEC. *NATURALISTE CANADIEN*, *106*(4), 425–437.

Heo, I., Kim, S., Han, G. H., Im, S., Kim, J. W., Hwang, D. Y., Jang, J. W., Lee, J. Y., Woo, S. D., & Shin, T. Y. (2023). Characteristics of insecticidal substances from the entomopathogenic fungus Metarhizium pinghaense 15R against cotton aphid in Korea. *JOURNAL OF ASIA-PACIFIC ENTOMOLOGY*, *26*(1). <https://doi.org/10.1016/j.aspen.2022.102013>

Herlinda, S. (2010). Spore Density and Viability of Entomopathogenic Fungal Isolates from Indonesia, and Their Virulence against Aphis gossypii Glover (Homoptera: Aphididae). *TROPICAL LIFE SCIENCES RESEARCH*, *21*(1), 11–19.

Hersanti, H., Hidayat, S., Susanto, A., Virgiawan, R., & Joni, I. M. (2018). *The effectiveness of Penicillium sp. Mixed with silica nanoparticles in controlling Myzus persicae* (I. M. Joni & C. Panatarani, Eds.; Vol. 1927). American Institute of Physics Inc.; Scopus. <https://doi.org/10.1063/1.5021222>

Hesketh, H., Alderson, P. G., Pye, B. J., & Pell, J. K. (2008). The development and multiple uses of a standardised bioassay method to select hypocrealean fungi for biological control of aphids. *Biological Control*, *46*(2), 242–255. Scopus. <https://doi.org/10.1016/j.biocontrol.2008.03.006>

Hewitt, K. G., Hofmann, R. W., Ball, O. J., Cox, N., Bryant, R. H., Finch, S. C., & Popay, A. J. (2024). Root aphid (Aploneura lentisci) population size on perennial ryegrass is determined by drought and endophyte strain. *JOURNAL OF PEST SCIENCE*, *97*(1), 369–384. <https://doi.org/10.1007/s10340-023-01630-8>

Hewson, R. T., & Sagenmüller, A. (2000). Some contributions to integrated crop management in Europe. *Pest Management Science*, *56*(11), 954–956. Scopus. [https://doi.org/10.1002/1526-4998(200011)56:11<954::AID-PS222>3.0.CO;2-8](https://doi.org/10.1002/1526-4998(200011)56:11%3c954::AID-PS222%3e3.0.CO;2-8)

Hinchliffe, G., Bown, D. P., Gatehouse, J. A., & Fitches, E. (2010). Insecticidal activity of recombinant avidin produced in yeast. *JOURNAL OF INSECT PHYSIOLOGY*, *56*(6), 629–639. <https://doi.org/10.1016/j.jinsphys.2010.01.007>

Holubec, V., Havlícková, H., Hanusová, R., & Bocková, R. (1998). *Wild Triticeae as genetic resources of aphid, rust and powdery mildew resistance* (WOS:000085946900042). 341–350.

Homayoonzadeh, M., Esmaeily, M., Talebi, K., Allahyari, H., Reitz, S., & Michaud, J. P. (2022). Inoculation of cucumber plants with Beauveria bassiana enhances resistance to Aphis gossypii (Hemiptera: Aphididae) and increases aphid susceptibility to pirimicarb. *EUROPEAN JOURNAL OF ENTOMOLOGY*, *119*, 1–11. <https://doi.org/10.14411/eje.2022.001>

Honda, K., Hitora, Y., & Tsukamoto, S. (2023). Akanthomins A–C, aphidicolin analogs from a fungus Akanthomyces sp., that inhibit cell cycle. *Phytochemistry*, *216*. Scopus. <https://doi.org/10.1016/j.phytochem.2023.113885>

Horikawa, M., Shimazu, M., Aibe, M., Kaku, H., Inai, M., & Tsunoda, T. (2018). A role of uroleuconaphins, polyketide red pigments in aphid, as a chemopreventor in the host defense system against infection with entomopathogenic fungi. *Journal of Antibiotics*, *71*(12), 992–999. Scopus. <https://doi.org/10.1038/s41429-018-0093-4>

Horikoshi, R., Goto, K., Mitomi, M., Oyama, K., Hirose, T., Sunazuka, T., & Omura, S. (2022). Afidopyropen, a novel insecticide originating from microbial secondary extracts. *SCIENTIFIC REPORTS*, *12*(1). <https://doi.org/10.1038/s41598-022-06729-z>

Horton, D. R., Broers, D. A., Lewis, R. R., Granatstein, D., Zack, R. S., Unruh, T. R., Moldenke, A. R., & Brown, J. J. (2003). Effects of mowing frequency on densities of natural enemies in three Pacific Northwest pear orchards. *ENTOMOLOGIA EXPERIMENTALIS ET APPLICATA*, *106*(2), 135–145. <https://doi.org/10.1046/j.1570-7458.2003.00018.x>

Hsiao, W. F., Bidochka, M. J., & Khachatourians, G. G. (1992). EFFECT OF TEMPERATURE AND RELATIVE-HUMIDITY ON THE VIRULENCE OF THE ENTOMOPATHOGENIC FUNGUS, VERTICILLIUM-LECANII, TOWARD THE OAT-BIRD BERRY APHID, RHOPALOSIPHUM-PADI (HOM, APHIDIDAE). *JOURNAL OF APPLIED ENTOMOLOGY-ZEITSCHRIFT FUR ANGEWANDTE ENTOMOLOGIE*, *114*(5), 484–490. <https://doi.org/10.1111/j.1439-0418.1992.tb01155.x>

Hu, L., Yang, Y., Wang, Z., Wen, C., & Cheng, X. (2023). A real-world data analysis-based study of Chinese medicine treatment patterns after breast cancer surgery. *Medicine (United States)*, *102*(50), E36642. Scopus. <https://doi.org/10.1097/MD.0000000000036642>

Hu, Q., Min, L., Yang, X. Y., Jin, S. X., Zhang, L., Li, Y. Y., Ma, Y. Z., Qi, X. W., Li, D. Q., Liu, H. B., Lindsey, K., Zhu, L. F., & Zhang, X. L. (2018). Laccase GhLac1 Modulates Broad-Spectrum Biotic Stress Tolerance via Manipulating Phenylpropanoid Pathway and Jasmonic Acid Synthesis. *PLANT PHYSIOLOGY*, *176*(2), 1808–1823. <https://doi.org/10.1104/pp.17.01628>

Hu, Z. B., Luo, H. A., Wang, X. G., Huang, M. Z., Huang, L., Pang, H. L., Mao, C. H., Pei, H., Huang, C. Q., Liu, P. L., & Liu, A. P. (2014). Synthesis and evaluation o-benzyl oxime-ether derivatives containing ß-methoxyacrylate moiety for insecticidal and fungicidal activities. *Bulletin of the Korean Chemical Society*, *35*(4), 1073–1076. Scopus. <https://doi.org/10.5012/bkcs.2014.35.4.1073>

Hua, L., & Feng, M. G. (2003). New use of broomcorn millets for production of granular cultures of aphid-pathogenic fungus Pandora neoaphidis for high sporulation potential and infectivity to Myzus persicae. *FEMS Microbiology Letters*, *227*(2), 311–317. Scopus. <https://doi.org/10.1016/S0378-1097(03)00711-0>

Huang, D., Huang, M., Liu, W., Liu, A., Liu, X., Chen, X., Pei, H., Sun, J., Yin, D., & Wang, X. (2017). Design, synthesis and biological evaluation of 1H-pyrazole-5-carboxamide derivatives as potential fungicidal and insecticidal agents. *Chemical Papers*, *71*(11), 2053–2061. Scopus. <https://doi.org/10.1007/s11696-017-0198-4>

Huang, J. H., Xiang, M. M., & Jiang, Z. D. (2012). Endophytic Fungi of Bitter Melon (Momordica charantia) in Guangdong Province, China. *GREAT LAKES ENTOMOLOGIST*, *45*(1–2), 19–28.

Huang, J., Zhou, W., Zhang, X., & Li, Y. (2023). Roles of long non-coding RNAs in plant immunity. *PLoS Pathogens*, *19*(5). Scopus. <https://doi.org/10.1371/journal.ppat.1011340>

Huang, Z. H., & Feng, M. G. (2008). Resting spore formation of aphid-pathogenic fungus Pandora nouryi depends on the concentration of infective inoculum. *Environmental Microbiology*, *10*(7), 1912–1916. Scopus. <https://doi.org/10.1111/j.1462-2920.2008.01577.x>

Huang, Z. H., Feng, M. G., Chen, X. X., & Liu, S. S. (2008). Pathogenic fungi and parasitoids of aphids present in air captures of migratory alates in the low-latitude plateau of Yunnan, China. *Environmental Entomology*, *37*(5), 1264–1271. Scopus. [https://doi.org/10.1603/0046-225X(2008)37[1264:PFAPOA]2.0.CO;2](https://doi.org/10.1603/0046-225X(2008)37%5b1264:PFAPOA%5d2.0.CO;2)

Huerta-Cepas, J., Capella-Gutierrez, S., Pryszcz, L. P., Denisov, I., Kormes, D., Marcet-Houben, M., & Gabaldón, T. (2011). PhylomeDB v3.0: An expanding repository of genome-wide collections of trees, alignments and phylogeny-based orthology and paralogy predictions. *NUCLEIC ACIDS RESEARCH*, *39*, D556–D560. <https://doi.org/10.1093/nar/gkq1109>

Hung, K. Y., Michailides, T. J., Millar, J. G., Wayadande, A., & Gerry, A. C. (2015). House Fly (Musca domestica L.) Attraction to Insect Honeydew. *PLOS ONE*, *10*(5). <https://doi.org/10.1371/journal.pone.0124746>

Hvam, A., & Toft, S. (2005). Effects of prey quality on the life history of a harvestman. *JOURNAL OF ARACHNOLOGY*, *33*(2), 582–590. <https://doi.org/10.1636/04-93.1>

Iasur-Kruh, L., Taha-Salaime, L., Robinson, W. E., Sharon, R., Droby, S., Perlman, S. J., & Zchori-Fein, E. (2015). Microbial Associates of the Vine Mealybug Planococcus ficus (Hemiptera: Pseudococcidae) under Different Rearing Conditions. *MICROBIAL ECOLOGY*, *69*(1), 204–214. <https://doi.org/10.1007/s00248-014-0478-2>

Ibrahim, L., Butt, T. M., & Jenkinson, P. (2002). Effect of artificial culture media on germination, growth, virulence and surface properties of the entomopathogenic hyphomycete Metarhizium anisopliae. *MYCOLOGICAL RESEARCH*, *106*, 705–715. <https://doi.org/10.1017/S0953756202006044>

Ibrahim, M. H., Barry, N. M., Khaleel, A. I., Mohmed, A. S., & Jwada, R. A. (2020). Bioefficacy of crude filtrates of two species entomopathogenic fungi to control the aphis fabae scopoli (HEMIPTERA: APHIDIDAE) under laboratory conditions. *Plant Archives*, *20*, 2411–2414. Scopus.

Ikeura, H. (2014). Use of plant volatile for plant pathogens and pests management. In *Basic and Appl. Asp. Of Biopesticides* (Vol. 9788132218777, pp. 181–192). Springer India; Scopus. <https://doi.org/10.1007/978-81-322-1877-7_9>

Ilyas, A., & Nappu, M. B. (2021). *Study of technology packages to support increasing production of chili pepper commodities in South Sulawesi*. *807*. Scopus. <https://doi.org/10.1088/1755-1315/807/4/042062>

Im, Y., Park, S. E., Lee, S. Y., Kim, J. C., & Kim, J. S. (2022). Early-Stage Defense Mechanism of the Cotton Aphid Aphis gossypii Against Infection With the Insect-Killing Fungus Beauveria bassiana JEF-544. *FRONTIERS IN IMMUNOLOGY*, *13*. <https://doi.org/10.3389/fimmu.2022.907088>

Inayat, R., Khurshid, A., Boamah, S., Zhang, S. W., & Xu, B. L. (2022). Mortality, Enzymatic Antioxidant Activity and Gene Expression of Cabbage Aphid (Brevicoryne brassicae L.) in Response to Trichoderma longibrachiatum T6. *FRONTIERS IN PHYSIOLOGY*, *13*. <https://doi.org/10.3389/fphys.2022.901115>

Iosob, G. A., Cristea, T. O., Antal-Tremurici, A., Calara, M., & Benchea, C. (2024). Disease and pest management for solanaceous vegetables in the NE region of Romania. *Acta Horticulturae*, *1391*, 357–364. Scopus. <https://doi.org/10.17660/ActaHortic.2024.1391.49>

Irwin, J. A. G., Lloyd, D. L., & Lowe, K. F. (2001). Lucerne biology and genetic improvement—An analysis of past activities and future goals in Australia. *AUSTRALIAN JOURNAL OF AGRICULTURAL RESEARCH*, *52*(7), 699–712. <https://doi.org/10.1071/AR00181>

Islam, M. S., Subbiah, V. K., & Siddiquee, S. (2022). Efficacy of entomopathogenic trichoderma isolates against sugarcane woolly aphid, ceratovacuna lanigera zehntner (Hemiptera: Aphididae). *Horticulturae*, *8*(1). Scopus. <https://doi.org/10.3390/horticulturae8010002>

Islam, M. T. (2022). Current Status and Future Prospects of Cladosporium sp., a Biocontrol Agent for Sustainable Plant Protection. *BIOCONTROL SCIENCE*, *27*(4), 185–191. <https://doi.org/10.4265/bio.27.185>

Ismailova, L. I., & Gafurova, V. L. (1981). ECOLOGICAL AND BIOLOGICAL CHARACTERISTICS OF FUNGI FOUND IN SUCTORIAL INSECTS. *MIKOLOGIYA I FITOPATOLOGIYA*, *15*(5), 389–394.

Ivanoff, S. S. (1957). THE HOMEGARDEN CANTALOUPE, A VARIETY WITH COMBINED RESISTANCE TO DOWNY MILDEW, POWDERY MILDEW, AND APHIDS. *PHYTOPATHOLOGY*, *47*(9), 552–556.

Ivascu, A., Buciumanu, A., Lazar, V., & Tamas, D. (2007). *Recent trends in peach breeding in Romania*. *760*, 467–472. Scopus. <https://doi.org/10.17660/ActaHortic.2007.760.65>

Iversen, T., & Harding, S. (2007). Biological and other alternative control methods against the woolly beech aphid Phyllaphis fagi L. on beech Fagus sylvatica seedlings in forest nurseries. *JOURNAL OF PEST SCIENCE*, *80*(3), 159–166. <https://doi.org/10.1007/s10340-007-0168-7>

Ivkovic, M., Gapare, W. J., Wharton, T., Jovanovic, T., Elms, S., McRae, T. A., & Wu, H. X. (2010). Risks affecting breeding objectives for radiata pine in Australia. *AUSTRALIAN FORESTRY*, *73*(4), 265–278. <https://doi.org/10.1080/00049158.2010.10676338>

Jaber, L. R., & Araj, S. E. (2018). Interactions among endophytic fungal entomopathogens (Ascomycota: Hypocreales), the green peach aphid Myzus persicae Sulzer (Homoptera: Aphididae), and the aphid endoparasitoid Aphidius colemani Viereck (Hymenoptera: Braconidae). *Biological Control*, *116*, 53–61. Scopus. <https://doi.org/10.1016/j.biocontrol.2017.04.005>

Jaber, L. R., & Vidal, S. (2009). Interactions between an endophytic fungus, aphids and extrafloral nectaries: Do endophytes induce extrafloral-mediated defences in vicia faba? *Functional Ecology*, *23*(4), 707–714. Scopus. <https://doi.org/10.1111/j.1365-2435.2009.01554.x>

Jackson, C. W., Heale, J. B., & Hall, R. A. (1985). TRAITS ASSOCIATED WITH VIRULENCE TO THE APHID MACROSIPHONIELLA-SANBORNI IN 18 ISOLATES OF VERTICILLIUM-LECANII. *ANNALS OF APPLIED BIOLOGY*, *106*(1), 39–48. <https://doi.org/10.1111/j.1744-7348.1985.tb03092.x>

Jadon, K. S., Singh, S. K., Patel, N., & Sharma, A. K. (2020). Integrated pest management module for cumin (Cuminum cyminum) production under arid environment. *INDIAN JOURNAL OF AGRICULTURAL SCIENCES*, *90*(6), 1120–1124.

Jahan, F., Abbasipour, H., Askarianzadeh, A., Hassanshahi, G., & Saeedizadeh, A. (2014). Biology and Life Table Parameters of Brevicoryne brassicae (Hemiptera: Aphididae) on Cauliflower Cultivars. *JOURNAL OF INSECT SCIENCE*, *14*. <https://doi.org/10.1093/jisesa/ieu146>

James, R. R., Shaffer, B. T., Croft, B., & Lighthart, B. (1995). Field evaluation of Beauveria bassiana: Its persistence and effects on the pea aphid and a non-target coccinellid in alfalfa. *BIOCONTROL SCIENCE AND TECHNOLOGY*, *5*(4), 425–437. <https://doi.org/10.1080/09583159550039620>

Jandricic, S. E., Filotas, M., Sanderson, J. P., & Wraight, S. P. (2014). Pathogenicity of conidia-based preparations of entomopathogenic fungi against the greenhouse pest aphids Myzus persicae, Aphis gossypii, and Aulacorthum solani (Hemiptera: Aphididae). *JOURNAL OF INVERTEBRATE PATHOLOGY*, *118*, 34–46. <https://doi.org/10.1016/j.jip.2014.02.003>

Jankevica, L., Minova, S., Metla, Z., & Daugavietis, M. (2018). *Development of new environmentally natural insecticides product from coniferous biomass against insect pests*. *18*, 251–258. Scopus. <https://doi.org/10.5593/sgem2018/5.1/S20.033>

Jasim, W. A., & Mohammed, A. A. (2019). Efficacy of entomopathogenic fungi Verticillium lecanii and Isaria fumosorosea against Myzus persicae under laboratory conditions. *Plant Archives*, *19*, 1416–1419. Scopus.

Javed, K., Javed, H., Mukhtar, T., & Qiu, D. W. (2019). Pathogenicity of some entomopathogenic fungal strains to green peach aphid, Myzus persicae Sulzer (Homoptera: Aphididae). *EGYPTIAN JOURNAL OF BIOLOGICAL PEST CONTROL*, *29*(1). <https://doi.org/10.1186/s41938-019-0183-z>

Javedi, K., Javed, H., Mukhtar, T., & Qiu, D. W. (2019). EFFICACY OF Beauveria bassiana AND Verticillium lecanii FOR THE MANAGEMENT OF WHITEFLY AND APHID. *PAKISTAN JOURNAL OF AGRICULTURAL SCIENCES*, *56*(3), 669–674. <https://doi.org/10.21162/PAKJAS/19.8396>

Javornik, B., Jakse, J., Stajner, N., Kozjak, P., & Cerenak, A. (2005). *Molecular genetic hop (Humulus lupulus L.) research in Slovenia* (WOS:000229258900002). 31–34. <https://doi.org/10.17660/ActaHortic.2005.668.2>

Jenkyn, J. F., & Plumb, R. T. (1983). EFFECTS OF FUNGICIDES AND INSECTICIDES APPLIED TO SPRING BARLEY SOWN ON DIFFERENT DATES IN 1976-79. *ANNALS OF APPLIED BIOLOGY*, *102*(3), 421–433. <https://doi.org/10.1111/j.1744-7348.1983.tb02715.x>

Jenkyn, J. F., & Rawlinson, C. J. (1977). EFFECTS OF FUNGICIDES AND INSECTICIDES ON MILDEW, VIRUSES AND ROOT YIELD OF SWEDES. *PLANT PATHOLOGY*, *26*(4), 166–174. <https://doi.org/10.1111/j.1365-3059.1977.tb01055.x>

Jensen, R. E., Enkegaard, A., & Steenberg, T. (2019). Increased fecundity of Aphis fabae on Vicia faba plants following seed or leaf inoculation with the entomopathogenic fungus Beauveria bassiana. *PLOS ONE*, *14*(10). <https://doi.org/10.1371/journal.pone.0223616>

Jeong, J. K., Kyu, C. K., & Roberts, D. W. (2005). Impact of the entomopathogenic fungus Verticillium lecanii on development of an aphid parasitoid, Aphidius colemani. *Journal of Invertebrate Pathology*, *88*(3), 254–256. Scopus. <https://doi.org/10.1016/j.jip.2005.01.004>

Jesionek, A., Kokotkiewicz, A., Krolicka, A., Zabiegala, B., & Luczkiewicz, M. (2018). Elicitation strategies for the improvement of essential oil content in Rhododendron tomentosum (Ledum palustre) bioreactor-grown microshoots. *INDUSTRIAL CROPS AND PRODUCTS*, *123*, 461–469. <https://doi.org/10.1016/j.indcrop.2018.07.013>

Jespersen, L. B., & Toft, S. (2003). Compensatory growth following early nutritional stress in the Wolf Spider Pardosa prativaga. *FUNCTIONAL ECOLOGY*, *17*(6), 737–746. <https://doi.org/10.1111/j.1365-2435.2003.00788.x>

Jia, C., Ma, R., Qian, X., Qin, Z., & Xu, H. (2023). Synthesis，Characterization，Biological Activity and Structure-activity Relationship of 6-N-heterocyclic Substituted Sanguinarine Derivatives. *Gaodeng Xuexiao Huaxue Xuebao/Chemical Journal of Chinese Universities*, *44*(11). Scopus. <https://doi.org/10.7503/cjcu20230231>

Jin, K., Luo, Z. B., Jiang, X. D., Zhang, Y. J., Zhou, Y. H., & Pei, Y. (2011). Carbon catabolite repressor gene BbCre1 influences carbon source uptake but does not have a big impact on virulence in Beauveria bassiana. *JOURNAL OF INVERTEBRATE PATHOLOGY*, *106*(3), 400–406. <https://doi.org/10.1016/j.jip.2010.11.008>

Jin, K., Zhang, Y. J., Fang, W. G., Luo, Z. B., Zhou, Y. H., & Pei, Y. (2010). Carboxylate Transporter Gene JEN1 from the Entomopathogenic Fungus Beauveria bassiana Is Involved in Conidiation and Virulence. *APPLIED AND ENVIRONMENTAL MICROBIOLOGY*, *76*(1), 254–263. <https://doi.org/10.1128/AEM.00882-09>

Johnson, J. (1941). Chemical inactivation and the reactivation of a plant virus. *PHYTOPATHOLOGY*, *31*(8), 679–701.

Johnson, M. C., Bush, L. P., & Siegel, M. R. (1986). INFECTION OF TALL FESCUE WITH ACREMONIUM-COENOPHIALUM BY MEANS OF CALLUS-CULTURE. *PLANT DISEASE*, *70*(5), 380–382. <https://doi.org/10.1094/PD-70-380>

Johnson, M. C., Dahlman, D. L., Siegel, M. R., Bush, L. P., Latch, G. C. M., Potter, D. A., & Varney, D. R. (1985). INSECT FEEDING DETERRENTS IN ENDOPHYTE-INFECTED TALL FESCUE. *APPLIED AND ENVIRONMENTAL MICROBIOLOGY*, *49*(3), 568–571. <https://doi.org/10.1128/AEM.49.3.568-571.1985>

Jouda, G., Monia, B. H. K., & Naima, B. (2010a). First report of aphidopathogenic fungi fusarium semitectum (Berkeley and Ravenel, 1875) and fusarium sacchari (Butler and Hafiz Khan) Gams (1971) on capitophorus elaeagni (Del Guercio) (Hemiptera: Aphididae). *African Journal of Agricultural Research*, *5*(4), 290–293. Scopus.

Jouda, G., Monia, B. K., & Naima, B. (2010b). First report of aphidopathogenic fungi Fusarium semitectum (Berkeley and Ravenel, 1875) and Fusarium sacchari (Butler and Hafiz Khan) Gams (1971) on Capitophorus elaeagni (Del Guercio) (Hemiptera: Aphididae). *AFRICAN JOURNAL OF AGRICULTURAL RESEARCH*, *5*(4), 290–293. <https://doi.org/10.5897/AJAR09.279>

Joudrey, P., & Bjørnson, S. (2007). Effects of an unidentified microsporidium on the convergent lady beetle, Hippodamia convergens Guérin-Méneville (Coleoptera: Coccinellidae), used for biological control. *Journal of Invertebrate Pathology*, *94*(2), 140–143. Scopus. <https://doi.org/10.1016/j.jip.2006.09.001>

Julio, L. F., Burillo, J., Giménez, C., Cabrera, R., Díaz, C. E., Sanz, J., & González-Coloma, A. (2015). Chemical and biocidal characterization of two cultivated Artemisia absinthium populations with different domestication levels. *Industrial Crops and Products*, *76*, 787–792. Scopus. <https://doi.org/10.1016/j.indcrop.2015.07.041>

Juliya, R. F. (2019). Genetic diversity of Beauveria bassiana in semi natural and agricultural habitats and its biocontrol potential against cowpea aphid, Aphis craccivora Koch. *BRAZILIAN JOURNAL OF MICROBIOLOGY*, *50*(3), 697–704. <https://doi.org/10.1007/s42770-019-00102-5>

Juliya, R. F. (2020). Phylogeny, chitinase activity, and pathogenicity of Beauveria, Metarhizium and Lecanicillium species against cowpea aphid, Aphis craccivora Koch. *INTERNATIONAL JOURNAL OF TROPICAL INSECT SCIENCE*, *40*(2), 309–314. <https://doi.org/10.1007/s42690-019-00082-x>

Kamil, D., Prameeladevi, T., Ganesh, S., Prabhakaran, N., Nareshkumar, R., & Thomas, S. P. (2017). Green synthesis of silver nanoparticles by entomopathogenic fungus Beauveria bassiana and their bioefficacy against mustard aphid (Lipaphis erysimi Kalt.). *INDIAN JOURNAL OF EXPERIMENTAL BIOLOGY*, *55*(8), 555–561.

Kang, B. R., Han, J. H., Kim, J. J., & Kim, Y. C. (2018). Dual Biocontrol Potential of the Entomopathogenic Fungus, Isaria javanica, for Both Aphids and plant Fungal Pathogens. *MYCOBIOLOGY*, *46*(4), 440–447. <https://doi.org/10.1080/12298093.2018.1538073>

Kant, K., Sharma, Y. K., Ramanujam, B., Tyagi, S. K., Ranjan, J. K., Mishra, B. K., Meena, S. S., Vishal, M. K., & Meena, S. R. (2013). Biorational approaches for management of aphid (Hyadaphis coriandri Das) on fennel. *INDIAN JOURNAL OF HORTICULTURE*, *70*(2), 300–303.

Kapongo, J. P., Shipp, L., Kevan, P., & Broadbent, B. (2008). Optimal concentration of Beauveria bassiana vectored by bumble bees in relation to pest and bee mortality in greenhouse tomato and sweet pepper. *BIOCONTROL*, *53*(5), 797–812. <https://doi.org/10.1007/s10526-007-9142-9>

Kariluoto, K. T. (1982). NEW RECORDS OF THE GENUS ENTOMOPHTHORA-FRES INFESTING INSECTS IN FINLAND. *ANNALES ENTOMOLOGICI FENNICI*, *48*(2), 49–50.

Kariuki, E. M., Lovo, E. E., Price, T., Parikh, V., Duren, E. B., Avery, P. B., & Minteer, C. R. (2022). The consumption and survival rate of Lilioceris cheni (Coleoptera: Chrysomelidae) on air potato leaves exposed to Cordyceps javanica (Hypocreales: Cordycipitaceae). *FLORIDA ENTOMOLOGIST*, *105*(3), 258–261.

Kasal-Slavik, T., Eschweiler, J., Kleist, E., Mumm, R., Goldbach, H. E., Schouten, A., & Wildt, J. (2017). Early biotic stress detection in tomato (Solanum lycopersicum) by BVOC emissions. *PHYTOCHEMISTRY*, *144*, 180–188. <https://doi.org/10.1016/j.phytochem.2017.09.006>

Kaushik, N., Díaz, C. E., Chhipa, H., Fernando Julio, L., Fe Andrés, M., & González-Coloma, A. (2020). Chemical composition of an aphid antifeedant extract from an endophytic fungus, trichoderma sp. Efi671. *Microorganisms*, *8*(3). Scopus. <https://doi.org/10.3390/microorganisms8030420>

Kavetsou, E., Koutsoukos, S., Daferera, D., Polissiou, M. G., Karagiannis, D., Perdikis, D. C., & Detsi, A. (2019). Encapsulation of Mentha pulegium Essential Oil in Yeast Cell Microcarriers: An Approach to Environmentally Friendly Pesticides. *Journal of Agricultural and Food Chemistry*, *67*(17), 4746–4753. Scopus. <https://doi.org/10.1021/acs.jafc.8b05149>

Kayange, C. D. M., Njera, D., Nyirenda, S. P., & Mwamlima, L. (2019). Effectiveness of Tephrosia vogelii and Tephrosia candida Extracts against Common Bean Aphid (Aphis fabae) in Malawi. *ADVANCES IN AGRICULTURE*, *2019*. <https://doi.org/10.1155/2019/6704834>

Kelemu, S., White, J. F., & Rao, I. M. (2001). *The role of endophytic fungi in Brachiaria, a tropical forage grass* (WOS:000184566200281). 605–607.

Kempler, C., & Daubeny, H. A. (2008). *Red raspberry cultivars and selections from the Pacific Agri-Food Research Centre* (WOS:000254834300006). 71–75. <https://doi.org/10.17660/ActaHortic.2008.777.6>

Kern, M. (2008). *Development of new insecticides and fungicides* (WOS:000254824400015). 125–134. <https://doi.org/10.17660/ActaHortic.2008.776.15>

Khairi, M. M. A. (2019). *Genetics and Breeding of Jojoba [Simmondsia chinensis (Link) Schneider]* (WOS:000558932100009). <https://doi.org/10.1007/978-3-030-23265-8_8>

Khalil, S. K., Bartos, J., & Landa, Z. (1985). Effectiveness of Verticillium lecanii to reduce populations of aphids under glasshouse and field conditions. *Agriculture, Ecosystems and Environment*, *12*(2), 151–156. Scopus. <https://doi.org/10.1016/0167-8809(85)90076-3>

Khamis, W. M., Behiry, S. I., Marey, S. A., Al-Askar, A. A., Amer, G., Heflish, A. A., Su, Y. M., Abdelkhalek, A., & Gaber, M. K. (2023). Phytochemical analysis and insight into insecticidal and antifungal activities of Indian hawthorn leaf extract. *SCIENTIFIC REPORTS*, *13*(1). <https://doi.org/10.1038/s41598-023-43749-9>

Khamis, W. M., Heflish, A. A., El-Messeiry, S., Behiry, S. I., Al-Askar, A. A., Su, Y. M., Abdelkhalek, A., & Gaber, M. K. (2023). Swietenia mahagoni Leaves Extract: Antifungal, Insecticidal, and Phytochemical Analysis. *SEPARATIONS*, *10*(5). <https://doi.org/10.3390/separations10050301>

Khanal, D., Maharjan, S., Lamichhane, J., Neupane, P., Sharma, S., & Pandey, P. (2020). Efficacy of Biorational Compounds against Mustard Aphid (Lipaphis erysimi Kalt.) and English Grain Aphid (Sitobion avenae Fab.) under Laboratory Conditions in Nepal. *ADVANCES IN AGRICULTURE*, *2020*. <https://doi.org/10.1155/2020/9817612>

Khanal, D., Upadhyaya, N., Poudel, K., Adhikari, S., Maharjan, S., Pandey, P., & Joseph, M. N. (2023). Efficacy of entomo-pathogenic fungus and botanical pesticides against mustard aphid (Lipaphis erysimi Kalt.) at field condition Rupandehi Nepal. *JOURNAL OF KING SAUD UNIVERSITY SCIENCE*, *35*(8). <https://doi.org/10.1016/j.jksus.2023.102849>

Khatib, S., Sobeh, M., Faraloni, C., & Bouissane, L. (2023). Tanacetum species: Bridging empirical knowledge, phytochemistry, nutritional value, health benefits and clinical evidence. *Frontiers in Pharmacology*, *14*. Scopus. <https://doi.org/10.3389/fphar.2023.1169629>

Khedhri, S., Khammassi, M., Bouhachem, S. B., Pieracci, Y., Flamini, G., Gargouri, S., Amri, I., & Hamrouni, L. (2024). Tunisian Eucalyptus essential oils: Exploring their potential for biological applications. *PLANT BIOSYSTEMS*, *158*(1), 40–50. <https://doi.org/10.1080/11263504.2023.2287531>

Khedhri, S., Khammassi, M., Bouhachem, S. B., Pieracci, Y., Mabrouk, Y., Seger, E., Amri, I., Flamini, G., & Hamrouni, L. (2023). Metabolite profiling of four Tunisian Eucalyptus essential oils and assessment of their insecticidal and antifungal activities. *HELIYON*, *9*(12). <https://doi.org/10.1016/j.heliyon.2023.e22713>

Khursheed, A., Rather, M. A., Jain, V., Wani, A. R., Rasool, S., Nazir, R., Malik, N. A., & Majid, S. A. (2022). Plant based natural products as potential ecofriendly and safer biopesticides: A comprehensive overview of their advantages over conventional pesticides, limitations and regulatory aspects. *Microbial Pathogenesis*, *173*. Scopus. <https://doi.org/10.1016/j.micpath.2022.105854>

Kikuchi, O., & Satoh, K. (1998). Effect of Fungicides on Entomopathogenic Fungus, Verticillium lecanii. *Japanese Journal of Applied Entomology and Zoology*, *42*(3), 107–113. Scopus. <https://doi.org/10.1303/jjaez.42.107>

Kiliç, E. (2021). An evaluation of some entomopathogenic fungi for green peach aphid (Myzus persicae [Sulzer]), (homoptera: Aphididae) under laboratory conditons. *Applied Ecology and Environmental Research*, *19*(1), 413–423. Scopus. <https://doi.org/10.15666/aeer/1901_413423>

Kim, J. J. (2007). Influence of Lecanicillium attenuatum on the development and reproduction of the cotton aphid, Aphis gossypii. *BIOCONTROL*, *52*(6), 789–799. <https://doi.org/10.1007/s10526-006-9050-4>

Kim, J. J., Goettel, M. S., & Gillespie, D. R. (2007). Potential of Lecanicillium species for dual microbial control of aphids and the cucumber powdery mildew fungus, Sphaerotheca fuliginea. *Biological Control*, *40*(3), 327–332. Scopus. <https://doi.org/10.1016/j.biocontrol.2006.12.002>

Kim, J. J., Goettel, M. S., & Gillespie, D. R. (2008). Evaluation of Lecanicillium longisporum, Vertalec® for simultaneous suppression of cotton aphid, Aphis gossypii, and cucumber powdery mildew, Sphaerotheca fuliginea, on potted cucumbers. *Biological Control*, *45*(3), 404–409. Scopus. <https://doi.org/10.1016/j.biocontrol.2008.02.003>

Kim, J. J., Goettel, M. S., & Gillespie, D. R. (2010). Evaluation of Lecanicillium longisporum, Vertalec® against the cotton aphid, Aphis gossypii, and cucumber powdery mildew, Sphaerotheca fuliginea in a greenhouse environment. *Crop Protection*, *29*(6), 540–544. Scopus. <https://doi.org/10.1016/j.cropro.2009.12.011>

Kim, J. J., Jeong, G., Han, J. H., & Lee, S. (2013). Biological Control of Aphid Using Fungal Culture and Culture Filtrates of Beauveria bassiana. *MYCOBIOLOGY*, *41*(4), 221–224. <https://doi.org/10.5941/MYCO.2013.41.4.221>

Kim, J. J., & Kim, K. C. (2008). Selection of a highly virulent isolate of Lecanicillium attenuatum against cotton aphid. *Journal of Asia-Pacific Entomology*, *11*(1), 1–4. Scopus. <https://doi.org/10.1016/j.aspen.2008.02.001>

Kim, J. J., & Roberts, D. W. (2012). The relationship between conidial dose, moulting and insect developmental stage on the susceptibility of cotton aphid, Aphis gossypii, to conidia of Lecanicillium attenuatum, an entomopathogenic fungus. *Biocontrol Science and Technology*, *22*(3), 319–331. Scopus. <https://doi.org/10.1080/09583157.2012.656580>

Kim, J. S., & Je, Y. H. (2010a). A novel biopesticide production: Attagel-mediated precipitation of chitinase from Beauveria bassiana SFB-205 supernatant for thermotolerance. *APPLIED MICROBIOLOGY AND BIOTECHNOLOGY*, *87*(5), 1639–1648. <https://doi.org/10.1007/s00253-010-2543-1>

Kim, J. S., & Je, Y. H. (2010b). Relation of aphicidal activity with cuticular degradation by beauveria bassiana SFB-205 supernatant incorporated with polyoxyethylene-(3)-isotridecyl ether. *Journal of Microbiology and Biotechnology*, *20*(3), 506–509. Scopus.

Kim, J. S., & Je, Y. H. (2010c). Relation of Aphicidal Activity with Cuticular Degradation by Beauveria bassiana SFB-205 Supernatant Incorporated with Polyoxyethylene-(3)-Isotridecyl Ether. *JOURNAL OF MICROBIOLOGY AND BIOTECHNOLOGY*, *20*(3), 506–509. <https://doi.org/10.4014/jmb.0909.09012>

Kim, J. S., Je, Y. H., Skinner, M., & Parker, B. L. (2013). Effect of ethoxylation of isotridecyl alcohol on aphicidal activity of fungal supernatant formulation. *JOURNAL OF PESTICIDE SCIENCE*, *38*(1–2), 85–87. <https://doi.org/10.1584/jpestics.D12-052>

Kim, J. S., Je, Y. H., & Woo, E. O. (2010). Roles of adjuvants in aphicidal activity of enzymes from Beauveria bassiana (Ascomycota: Hypocreales) SFB-205 supernatant. *JOURNAL OF ASIA-PACIFIC ENTOMOLOGY*, *13*(4), 345–350. <https://doi.org/10.1016/j.aspen.2010.06.002>

Kim, J. S., Je, Y. H., & Yu, Y. M. (2011). Mass Production of Aphicidal Beauveria bassiana SFB-205 Supernatant with the Parameter of Chitinase. *JOURNAL OF MICROBIOLOGY AND BIOTECHNOLOGY*, *21*(6), 604–612. <https://doi.org/10.4014/jmb.1101.01001>

Kim, J. S., Roh, J. Y., Choi, J. Y., & Je, Y. H. (2010). Influence of two FPLC fractions from Beauveria bassiana SFB-205 supernatant on the insecticidal activity against cotton aphid. *BIOCONTROL SCIENCE AND TECHNOLOGY*, *20*(1), 77–81. <https://doi.org/10.1080/09583150903419538>

Kim, J. S., Roh, J. Y., Choi, J. Y., Wang, Y., Shim, H. J., & Je, Y. H. (2010). Correlation of the aphicidal activity of Beauveria bassiana SFB-205 supernatant with enzymes. *FUNGAL BIOLOGY*, *114*(1), 120–128. <https://doi.org/10.1016/j.mycres.2009.10.011>

Kim, S., Hwang, D. Y., Shin, T. Y., & Kwak, J. H. (2023). Correlation of fruit tree rhizosphere soils with entomopathogenic fungi. *ENTOMOLOGICAL RESEARCH*, *53*(9), 333–342. <https://doi.org/10.1111/1748-5967.12666>

Kindler, S. D., Breen, J. P., & Springer, T. L. (1991). REPRODUCTION AND DAMAGE BY RUSSIAN WHEAT APHID (HOMOPTERA, APHIDIDAE) AS INFLUENCED BY FUNGAL ENDOPHYTES AND COOL-SEASON TURFGRASSES. *JOURNAL OF ECONOMIC ENTOMOLOGY*, *84*(2), 685–692. <https://doi.org/10.1093/jee/84.2.685>

Kirchmair, M., Neuhauser, S., Strasser, H., Voloshchuk, N., Hoffmann, M., & Huber, L. (2009). *Biological Control of Grape Phylloxera—A Historical Review and Future Prospects* (WOS:000305457200001). *816*, 13–17. <https://doi.org/10.17660/ActaHortic.2009.816.1>

Kish, L. P., Majchrowicz, I., & Biever, K. D. (1994a). PREVALENCE OF NATURAL FUNGAL MORTALITY OF GREEN PEACH APHID (HOMOPTERA, APHIDIDAE) ON POTATOES AND NON-SOLANACEOUS HOSTS IN WASHINGTON AND IDAHO. *ENVIRONMENTAL ENTOMOLOGY*, *23*(5), 1326–1330. <https://doi.org/10.1093/ee/23.5.1326>

Kish, L. P., Majchrowicz, I., & Biever, K. D. (1994b). Prevalence of natural fungal mortality of green peach aphid (Homoptera: Aphididae) on potatoes and nonsolanaceous hosts in Washington and Idaho. *Environmental Entomology*, *23*(5), 1326–1339. Scopus. <https://doi.org/10.1093/ee/23.5.1326>

Klein, E., Brault, V., Klein, D., Weyens, G., Lefèbvre, M., Ziegler-Graff, V., & Gilmer, D. (2014). Divergence of host range and biological properties between natural isolate and full-length infectious cDNA clone of the Beet mild yellowing virus2ITB. *Molecular Plant Pathology*, *15*(1), 22–30. Scopus. <https://doi.org/10.1111/mpp.12061>

Klisiewicz, J. M. (1986). SUSCEPTIBILITY OF YELLOW STARTHISTLE TO SELECTED PLANT-PATHOGENS. *PLANT DISEASE*, *70*(4), 295–297. <https://doi.org/10.1094/PD-70-295>

Kluth, S., Kruess, A., & Tscharntke, T. (2001). Interactions between the rust fungus Puccinia punctiformis and ectophagous and endophagous insects on creeping thistle. *JOURNAL OF APPLIED ECOLOGY*, *38*(3), 548–556.

Kluth, S., Kruess, A., & Tscharntke, T. (2002). Insects as vectors of plant pathogens: Mutualistic and antagonistic interactions. *Oecologia*, *133*(2), 193–199. <https://doi.org/10.1007/s00442-002-1016-3>

Knoll, J. E., Uchimiya, M., Hayes, C. M., Punnuri, S. M., Harris-Shultz, K. R., & Smith, J. S. (2023). Registration of three sweet sorghum lines with high tolerance to sorghum aphid (Melanaphis sorghi). *JOURNAL OF PLANT REGISTRATIONS*, *17*(3), 551–560. <https://doi.org/10.1002/plr2.20310>

Knudsen, G. R., Johnson, J. B., & Eschen, D. J. (1990). ALGINATE PELLET FORMULATION OF A BEAUVERIA-BASSIANA (FUNGI, HYPHOMYCETES) ISOLATE PATHOGENIC TO CEREAL APHIDS. *JOURNAL OF ECONOMIC ENTOMOLOGY*, *83*(6), 2225–2228. <https://doi.org/10.1093/jee/83.6.2225>

Knudsen, G. R., & Schotzko, D. J. (1999). Spatial simulation of epizootics caused by Beauveria bassiana in Russian wheat aphid populations. *BIOLOGICAL CONTROL*, *16*(3), 318–326. <https://doi.org/10.1006/bcon.1999.0713>

Knudsen, G. R., Schotzko, D. J., & Krag, C. R. (1994). FUNGAL ENTOMOPATHOGEN EFFECT ON NUMBERS AND SPATIAL PATTERNS OF THE RUSSIAN WHEAT APHID (HOMOPTERA, APHIDIDAE) ON PREFERRED AND NONPREFERRED HOST PLANTS. *ENVIRONMENTAL ENTOMOLOGY*, *23*(6), 1558–1567. <https://doi.org/10.1093/ee/23.6.1558>

Koch, K. A., Potter, B. D., & Ragsdale, D. W. (2010). Non-target impacts of soybean rust fungicides on the fungal entomopathogens of soybean aphid. *JOURNAL OF INVERTEBRATE PATHOLOGY*, *103*(3), 156–164. <https://doi.org/10.1016/j.jip.2009.12.003>

Kong, C. H., Hu, F., & Xu, X. H. (2002). Allelopathic potential and chemical constituents of volatiles from Ageratum conyzoides under stress. *JOURNAL OF CHEMICAL ECOLOGY*, *28*(6), 1173–1182. <https://doi.org/10.1023/A:1016229616845>

Kószegi, B., Linc, G., Juhász, A., Láng, L., & Molnár-Láng, M. (2000). Occurrence of the 1RS/1BL wheat-rye translocation in Hungarian wheat varieties. *Acta Agronomica Hungarica*, *48*(3), 227–236. Scopus. <https://doi.org/10.1556/AAgr.48.2000.3.2>

Krishnaveni, S., Muthukrishnan, S., Liang, G. H., Wilde, G., & Manickam, A. (1999). Induction of chitinases and β-1,3-glucanases in resistant and susceptible cultivars of sorghum in response to insect attack, fungal infection and wounding. *PLANT SCIENCE*, *144*(1), 9–16. <https://doi.org/10.1016/S0168-9452(99)00049-7>

Krízkovská, B., Viktorová, J., & Lipov, J. (2022). Approved Genetically Modified Potatoes (Solanum tuberosum) for Improved Stress Resistance and Food Safety. *JOURNAL OF AGRICULTURAL AND FOOD CHEMISTRY*, *70*(38), 11833–11843. <https://doi.org/10.1021/acs.jafc.2c03837>

Kuhlmann, F., & Müller, C. (2009). Independent responses to ultraviolet radiation and herbivore attack in broccoli. *JOURNAL OF EXPERIMENTAL BOTANY*, *60*(12), 3467–3475. <https://doi.org/10.1093/jxb/erp182>

Kuhlmann, F., & Müller, C. (2010). UV-B impact on aphid performance mediated by plant quality and plant changes induced by aphids. *PLANT BIOLOGY*, *12*(4), 676–684. <https://doi.org/10.1111/j.1438-8677.2009.00257.x>

Kulkarni, N. S., & Kumar, V. (2020). Influence of aphids Aphis craccivora on yield parameters of lucerne (Medicago sativa L.) and their management with different IPM components. *RANGE MANAGEMENT AND AGROFORESTRY*, *41*(1), 178–181.

Kumar, A., & Chauhan, J. S. (2005). Status and future thrust areas of rapeseed-mustard research in India. *INDIAN JOURNAL OF AGRICULTURAL SCIENCES*, *75*(10), 621–635.

Kumar, M. P. S., Keerthana, A., Singh, S. K., Rai, D., Jaiswal, A., & Reddy, M. S. S. (2024). Exploration of culturable bacterial associates of aphids and their interactions with entomopathogens. *ARCHIVES OF MICROBIOLOGY*, *206*(3). <https://doi.org/10.1007/s00203-024-03830-x>

Kumar, M., Yusuf, M. A., Nigam, M., & Kumar, M. (2018). An Update on Genetic Modification of Chickpea for Increased Yield and Stress Tolerance. *MOLECULAR BIOTECHNOLOGY*, *60*(8), 651–663. <https://doi.org/10.1007/s12033-018-0096-1>

Kuriyama, T., Kakemoto, E., Takahashi, N., Imamura, K. I., Oyama, K., Suzuki, E., Harimaya, K., Yaguchi, T., & Ozoe, Y. (2004). Receptor assay-guided isolation of anti-GABAergic insecticidal alkaloids from a fungal culture. *Journal of Agricultural and Food Chemistry*, *52*(12), 3884–3887. Scopus. <https://doi.org/10.1021/jf049870g>

Lacatena, F., Marra, R., Mazzei, P., Piccolo, A., Digilio, M. C., Giorgini, M., Woo, S. L., Cavallo, P., Lorito, M., & Vinale, F. (2019). Chlamyphilone, a Novel Pochonia chlamydosporia Metabolite with Insecticidal Activity. *MOLECULES*, *24*(4). <https://doi.org/10.3390/molecules24040750>

Lacey, L. A., Horton, D. R., Chauvin, R. L., & Stocker, J. M. (1999). Comparative efficacy of Beauveria bassiana, Bacillus thuringiensis, and aldicarb for control of Colorado potato beetle in an irrigated desert agroecosystem and their effects on biodiversity. *ENTOMOLOGIA EXPERIMENTALIS ET APPLICATA*, *93*(2), 189–200. <https://doi.org/10.1046/j.1570-7458.1999.00578.x>

Lacey, L. A., Mesquita, A. L. M., Mercadier, G., Debire, R., Kazmer, D. J., & Leclant, F. (1997). Acute and sublethal activity of the entomopathogenic fungus Paecilomyces fumosoroseus (Deuteromycotina: Hyphomycetes) on adult Aphelinus asychis (Hymenoptera: Aphelinidae). *ENVIRONMENTAL ENTOMOLOGY*, *26*(6), 1452–1460. <https://doi.org/10.1093/ee/26.6.1452>

Lagnaoui, A., & Radcliffe, E. B. (1998). Potato fungicides interfere with entomopathogenic fungi impacting population dynamics of green peach aphid. *AMERICAN JOURNAL OF POTATO RESEARCH*, *75*(1), 19–25. <https://doi.org/10.1007/BF02883513>

Lahoz, E., Carrieri, R., Crescenzi, A., & Fanigliulo, A. (2015). *Viral and Fungal Diseases of Processing and Fresh Tomato in the Mediterranean Basin* (WOS:000358011100014). *1069*, 99–105.

Laihonen, M., Saikkonen, K., Helander, M., de Aldana, B. V. R., Zabalgogeazcoa, I., & Fuchs, B. (2022). Epichloe Endophyte-Promoted Seed Pathogen Increases Host Grass Resistance Against Insect Herbivory. *FRONTIERS IN MICROBIOLOGY*, *12*. <https://doi.org/10.3389/fmicb.2021.786619>

Lakshminarayana, R. (1993). IMPACT OF HIGH-YIELDING VARIETIES OF TOBACCO (NICOTIANA-TABACUM) AND CHANGING ASPECTS OF THEIR CULTIVATION. *INDIAN JOURNAL OF AGRICULTURAL SCIENCES*, *63*(11), 685–693.

LaLone, C. A., Villeneuve, D. L., Lyons, D., Helgen, H. W., Robinson, S. L., Swintek, J. A., Saari, T. W., & Ankley, G. T. (2016). Sequence alignment to predict across species susceptibility (seqapass): A web-based tool for addressing the challenges of cross-species extrapolation of chemical toxicity. *Toxicological Sciences*, *153*(2), 228–245. Scopus. <https://doi.org/10.1093/toxsci/kfw119>

Lammerink, J., & Hart, R. W. (1985). TINA, A NEW SWEDE CULTIVAR WITH RESISTANCE TO DRY ROT AND CLUBROOT. *NEW ZEALAND JOURNAL OF EXPERIMENTAL AGRICULTURE*, *13*(4), 417–420. <https://doi.org/10.1080/03015521.1985.10426113>

Latch, G. C. M. (2015). Diseases and endophytes. In *Tall Fescue for the Twenty-First Century* (pp. 119–127). wiley; Scopus. <https://doi.org/10.2134/agronmonogr53.c8>

Latch, G. C. M., Christensen, M. J., & Gaynor, D. L. (1985). APHID DETECTION OF ENDOPHYTE INFECTION IN TALL FESCUE. *NEW ZEALAND JOURNAL OF AGRICULTURAL RESEARCH*, *28*(1), 129–132. <https://doi.org/10.1080/00288233.1985.10427006>

Laterrot, H. (1995). *Practical situation and fresh hopes for the genetic control of the main pathogens of the Mediterranean fresh market tomato crops* (WOS:000071774700059). 490–502. <https://doi.org/10.17660/ActaHortic.1995.412.59>

Latiff, N., Omar, D., Sajap, A. S., Awang, R. M., Rajimin, R., Muning, M., & Peng, T. L. (2022). Effectiveness of Isolates of Metarhizium anisopliae against Aphis gossypii (Hemiptera: Aphididae) on Capsicum annum and Solanum melongena. *International Journal of Agriculture and Biology*, *28*(3), 187–192. Scopus. <https://doi.org/10.17957/IJAB/15.1969>

Latteur, G., & Jansen, J. P. (2002). Effects of 20 fungicides on the infectivity of conidia of the aphid entomopathogenic fungus Erynia neoaphidis. *BioControl*, *47*(4), 435–444. Scopus. <https://doi.org/10.1023/A:1015639017666>

Lawson, R. H., & Hsu, H. T. (1996). *Lily diseases and their control* (WOS:000071766300021). 175–187. <https://doi.org/10.17660/ActaHortic.1996.414.21>

Lazebnik, J., Arpaia, S., Baldacchino, F., Banzato, P., Moliterni, S., Vossen, J. H., van de Zande, E. M., & van Loon, J. J. A. (2017). Effects of a genetically modified potato on a non-target aphid are outweighed by cultivar differences. *Journal of Pest Science*, *90*(3), 855–864. Scopus. <https://doi.org/10.1007/s10340-017-0831-6>

Lazebnik, J., Tibboel, M., Dicke, M., & van Loon, J. J. A. (2017). Inoculation of susceptible and resistant potato plants with the late blight pathogen Phytophthora infestans: Effects on an aphid and its parasitoid. *Entomologia Experimentalis et Applicata*, *163*(3), 305–314. Scopus. <https://doi.org/10.1111/eea.12582>

Leal, S. C. M., Bertoli, D. J., Ball, B. V., & Butt, T. M. (1994). PRESENCE OF DOUBLE-STRANDED RNAS AND VIRUS-LIKE PARTICLES IN THE ENTOMOPATHOGENIC FUNGUS METARHIZIUM-ANISOPLIAE. *BIOCONTROL SCIENCE AND TECHNOLOGY*, *4*(1), 89–94. <https://doi.org/10.1080/09583159409355316>

Lebeda, A., Krístková, E., Kitner, M., Mieslerová, B., Jemelková, M., & Pink, D. A. C. (2014). Wild Lactuca species, their genetic diversity, resistance to diseases and pests, and exploitation in lettuce breeding. *EUROPEAN JOURNAL OF PLANT PATHOLOGY*, *138*(3), 597–640. <https://doi.org/10.1007/s10658-013-0254-z>

Lebeda, A., Křístkova, E., Kitner, M., Mieslerová, B., Jemelková, M., & Pink, D. A. C. (2015). *Resistance of wild Lactuca genetic resources to diseases and pests, and their exploitation in lettuce* (S. E. Ashmore, M. E. Dulloo, M. Taylor, H. Jaenicke, & L. Guarino, Eds.; Vol. 1101, pp. 133–140). International Society for Horticultural Science; Scopus. <https://doi.org/10.17660/ActaHortic.2015.1101.20>

Lebeda, A., Krístková, E., Kitner, M., Mieslerová, B., Jemelková, M., & Pink, D. A. C. (2015). *Resistance of wild Lactuca genetic resources to diseases and pests, and their exploitation in lettuce breeding* (WOS:000378649000020). *1101*, 133–139. <https://doi.org/10.17660/ActaHortic.2015.1101.20>

Leckie, D., Astley, D., Crute, I. R., Ellis, P. R., Pink, D. A. C., Boukema, I., Monteiro, A. A., & Dias, S. (1996). *The location and exploitation of genes for pest and disease resistance in European gene bank collections of horticultural brassicas* (WOS:000071638600010). 95–101. <https://doi.org/10.17660/ActaHortic.1996.407.10>

Lee, G. J., Song, M. G., Kim, S. D., Nam, S. Y., Heo, J. W., Yoon, J. B., & Kim, D. E. (2016). Effects of Rain-shelter Types on Growth and Fruit Quality of Red Pepper (Capsicum annuum L. var. ’Keummaru’) Cultivation in Paddy. *KOREAN JOURNAL OF HORTICULTURAL SCIENCE & TECHNOLOGY*, *34*(3), 355–362. <https://doi.org/10.12972/kjhst.20160036>

Lee, J. Y., Kang, S. W., Yoon, C. S., Kim, J. J., Choi, D. R., & Kim, S. W. (2006). Verticillium lecanii spore formulation using UV protectant and wetting agent and the biocontrol of cotton aphids. *BIOTECHNOLOGY LETTERS*, *28*(13), 1041–1045. <https://doi.org/10.1007/s10529-006-9036-4>

Lee, S. C., Wu, M., & Wong, S. M. (1993). NUCLEOTIDE-SEQUENCE OF A SINGAPORE ISOLATE OF ZUCCHINI YELLOW MOSAIC-VIRUS COAT PROTEIN GENE REVEALED AN ALTERED DAG MOTIF. *VIRUS GENES*, *7*(4), 381–387. <https://doi.org/10.1007/BF01703393>

Lee, W. W., Shin, T. Y., Bae, S. M., & Woo, S. D. (2015). Screening and evaluation of entomopathogenic fungi against the green peach aphid, Myzus persicae, using multiple tools. *Journal of Asia-Pacific Entomology*, *18*(3), 607–615. Scopus. <https://doi.org/10.1016/j.aspen.2015.07.012>

Lee, Y. S., Han, J. H., Kang, B. R., & Kim, Y. C. (2019). Dibutyl succinate, produced by an insect-pathogenic fungus, Isaria javanica pf185, is a metabolite that controls of aphids and a fungal disease, anthracnose. *Pest Management Science*, *75*(3), 852–858. Scopus. <https://doi.org/10.1002/ps.5191>

Lefort, F., Fleury, D., Fleury, I., Coutant, C., Kuske, S., Kehrli, P., & Maignet, P. (2014). Pathogenicity of entomopathogenic fungi to the green peach aphid myzus persicae sulzer (Aphididae) and the european tarnished bug lygus rugulipennis poppius (Miridae). *Egyptian Journal of Biological Pest Control*, *24*(2), 379–386. Scopus.

Lehman, W. F., Erwin, D. C., & Stanford, E. H. (1967). ROOT ROT TOLERANCE IN NEW ALFALFA STRAINS NOW AVAILABLE TO PLANT BREEDERS. *CALIFORNIA AGRICULTURE*, *21*(4), 6-+.

Lei, J. X., Finlayson, S. A., Salzman, R. A., Shan, L. B., & Zhu-Salzman, K. (2014). BOTRYTIS-INDUCED KINASE1 Modulates Arabidopsis Resistance to Green Peach Aphids via PHYTOALEXIN DEFICIENT4. *PLANT PHYSIOLOGY*, *165*(4), 1657–1670. <https://doi.org/10.1104/pp.114.242206>

Lei, J., & Zhu-Salzman, K. (2015). Enhanced aphid detoxification when confronted by a host with elevated ROS production. *Plant Signaling and Behavior*, *10*(4), 1–4. Scopus. <https://doi.org/10.1080/15592324.2015.1010936>

Lenfant, C., Lyoussoufi, A., Chen, X., Darcier, F. F., & Sauphanor, B. (1994). POTENTIAL OF FORFICULA-AURICULARIA L AS PREDATOR OF PEAR-PSYLLA CACOPSYLLA-PYRI (L). *ENTOMOLOGIA EXPERIMENTALIS ET APPLICATA*, *73*(1), 51–60. <https://doi.org/10.1111/j.1570-7458.1994.tb01838.x>

Leopoldo, R. G. R., Manuel, L. M., Lourdes, V. N., & Teófilo, S. C. (2017). Biocontrol capacity of Beauveria brongniartii (Sacc.) and Metarhizium anisopliae (Metsch.) in the control of aphids Macrosiphum euphorbiae (Hemiptera: Aphididae). *JOURNAL OF THE SELVA ANDINA RESEARCH SOCIETY*, *8*(1), 48–68.

Li, J., Liu, X. H., Wang, Q. M., Sun, J. Y., & He, D. X. (2021). Genome-wide identification and analysis of cystatin family genes in Sorghum (Sorghum bicolor (L. Moench). *PEERJ*, *9*. <https://doi.org/10.7717/peerj.10617>

Li, K., Li, Y., Yi, Y., Xu, L., Ye, J., Ou, X., Li, J., & Hu, A. (2020). Design, Synthesis and Biological Activity of 5-Pyrazole Carboxamides. *Gaodeng Xuexiao Huaxue Xuebao/Chemical Journal of Chinese Universities*, *41*(4), 716–725. Scopus. <https://doi.org/10.7503/cjcu20190504>

Li, M., Li, K., Yi, Y., Luo, X., Qiu, L., Zhang, L., Wang, M., Ye, J., Ou, X., Li, J., Chen, Y., & Hu, A. (2023). Synthesis, crystal structure, biological evaluation, docking study and DFT calculation of novel strobilurins containing oxime ether phenyl ring or dihydrobenzofuran moiety. *Journal of Molecular Structure*, *1286*. Scopus. <https://doi.org/10.1016/j.molstruc.2023.135636>

Li, M. Y., Wang, Y., Lei, X., Xu, C. T., Wang, D. D., Liu, S., & Li, S. G. (2021). Molecular characterization of a catalase gene from the green peach aphid (Myzus persicae). *ARCHIVES OF INSECT BIOCHEMISTRY AND PHYSIOLOGY*, *108*(2). <https://doi.org/10.1002/arch.21835>

Li, T., Blande, J. D., Gundel, P. E., Helander, M., & Saikkonen, K. (2014). Epichloe Endophytes Alter Inducible Indirect Defences in Host Grasses. *PLOS ONE*, *9*(6). <https://doi.org/10.1371/journal.pone.0101331>

Li, W., & Sheng, C. F. (2007). Occurrence and distribution of entomophthoralean fungi infecting aphids in mainland China. *Biocontrol Science and Technology*, *17*(4), 433–439. Scopus. <https://doi.org/10.1080/09583150701213802>

Li, X., Zhu, K., Han, Q., Lu, X. X., Li, M. J., Ling, Y., & Duan, H. X. (2023). Design, Synthesis and Bioactivity Study on Novel Furan a-Butenolactone Compounds. *CHINESE JOURNAL OF ORGANIC CHEMISTRY*, *43*(1), 202–213. <https://doi.org/10.6023/cjoc202206010>

Li, Y., Nan, Z., Matthew, C., Wang, Y., & Duan, T. (2023). Arbuscular mycorrhizal fungus changes alfalfa (Medicago sativa) metabolites in response to leaf spot (Phoma medicaginis) infection, with subsequent effects on pea aphid (Acyrthosiphon pisum) behavior. *New Phytologist*, *239*(1), 286–300. Scopus. <https://doi.org/10.1111/nph.18924>

Li, Z., Lin, J., Ma, J., Wu, D., & Zhang, Y. (2008). Influence of different drying temperatures for solid substrate after fermentation on conidia characteristics of the entomopathogenic fungus Beauveria bassiana. *Wei sheng wu xue bao = Acta microbiologica Sinica*, *48*(7), 887–892. Scopus.

Ling, L., & Yang, J. Y. (1940). A mosaic disease of rape and others cultivated crucifers in China. *PHYTOPATHOLOGY*, *30*(4), 338–342.

Liswarni, Y., & Putra, F. S. (2020). Colonization of beauveria bassiana (Bals.) vuill on chili (capsicum annum) and its effect on populations of Myzus persicae. *Journal of Biopesticides*, *13*(1), 40–46. Scopus.

Liu, C. P., & Liu, S. D. (2009). Formulation and characterization of the microencapsulated entomopathogenic fungus Metarhizium anisopliae MA126. *JOURNAL OF MICROENCAPSULATION*, *26*(5), 377–384. <https://doi.org/10.1080/02652040802365455>

Liu, H. L., Che, Z. J., Zeng, X. R., Zhang, G. Z., Wang, H., & Yu, D. Y. (2016). Identification of single nucleotide polymorphisms in soybean associated with resistance to common cutworm (Spodoptera litura Fabricius). *EUPHYTICA*, *209*(1), 49–62. <https://doi.org/10.1007/s10681-016-1631-4>

Liu, S., Lenoir, C. J. G., Amaro, T., Rodriguez, P. A., Huitema, E., & Bos, J. I. B. (2022). Virulence strategies of an insect herbivore and oomycete plant pathogen converge on host E3 SUMO ligase SIZ1. *NEW PHYTOLOGIST*, *235*(4), 1599–1614. <https://doi.org/10.1111/nph.18184>

Liu, X. C., Du, C. X., Tan, Y. Q., Yue, C., & Fan, H. F. (2024). Interplant communication increases aphid resistance and alters rhizospheric microbes in neighboring plants of aphid-infested cucumbers. *PEST MANAGEMENT SCIENCE*. <https://doi.org/10.1002/ps.8222>

Liu, X., Qiu, X., Duan, Z., Ping, D., Zhou, X., Yang, J., Zhou, W., & Wan, Y. (2018). A novel strain of Pseudozyma aphidis from mulberry parasitises the conidia of mulberry powdery mildew fungus Phyllactinia sp. And its biocontrol effect in the fields. *Biocontrol Science and Technology*, *28*(1), 62–76. Scopus. <https://doi.org/10.1080/09583157.2017.1416067>

Liu, Y., & Buchenauer, H. (2005a). Effect of infections with barley yellow dwarf virus and Fusarium spp. On assimilation of 14CO2 by flag leaves and translocation of photosynthates in wheat. *Zeitschrift Fur Pflanzenkrankheiten Und Pflanzenschutz*, *112*(6), 529–543. Scopus. <https://doi.org/10.1007/bf03356150>

Liu, Y., & Buchenauer, H. (2005b). Effect of infections with barley yellow dwarf virus and Fusarium spp. On assimilation of 14CO2 by flag leaves and translocation of photosynthates in wheat. *ZEITSCHRIFT FUR PFLANZENKRANKHEITEN UND PFLANZENSCHUTZ-JOURNAL OF PLANT DISEASES AND PROTECTION*, *112*(6), 529–543.

Liu, Y. J., Liu, J., Ying, S. H., Liu, S. S., & Feng, M. G. (2013). A Fungal Insecticide Engineered for Fast Per Os Killing of Caterpillars Has High Field Efficacy and Safety in Full-Season Control of Cabbage Insect Pests. *APPLIED AND ENVIRONMENTAL MICROBIOLOGY*, *79*(20), 6452–6458. <https://doi.org/10.1128/AEM.01594-13>

Liverani, A., Brandi, F., Leone, A., Sirri, S., & Giovannini, D. (2015). *Screening Controlled Pollination Peach Seedlings for Mildew and Brown Rot Resistance at CRA-FRF in the Frame of the Fruitbreedomics EU Project* (WOS:000378619300025). *1084*, 187–193. <https://doi.org/10.17660/ActaHortic.2015.1084.25>

Lloyd, D. L., Gramshaw, D., Hilder, T. B., Ludke, D. H., & Turner, J. W. (1985). PERFORMANCE OF NORTH-AMERICAN AND AUSTRALIAN LUCERNES IN THE QUEENSLAND SUBTROPICS .3. YIELD, PLANT-SURVIVAL AND APHID POPULATIONS IN RAINGROWN STANDS. *AUSTRALIAN JOURNAL OF EXPERIMENTAL AGRICULTURE*, *25*(1), 91–99. <https://doi.org/10.1071/EA9850091>

Lockhart, B. E., Menke, J., Dahal, G., & Olszewski, N. E. (2000). Characterization and genomic analysis of tobacco vein clearing virus, a plant pararetrovirus that is transmitted vertically and related to sequences integrated in the host genome. *Journal of General Virology*, *81*(6), 1579–1585. Scopus. <https://doi.org/10.1099/0022-1317-81-6-1579>

Lopez, D. C., Zhu-Salzman, K., Ek-Ramos, M. J., & Sword, G. A. (2014). The Entomopathogenic Fungal Endophytes Purpureocillium lilacinum (Formerly Paecilomyces lilacinus) and Beauveria bassiana Negatively Affect Cotton Aphid Reproduction under Both Greenhouse and Field Conditions. *PLOS ONE*, *9*(8). <https://doi.org/10.1371/journal.pone.0103891>

Lordan, J., Alegre, S., Gatius, F., Sarasúa, M. J., & Alins, G. (2015). Woolly apple aphid Eriosoma lanigerum Hausmann ecology and its relationship with climatic variables and natural enemies in Mediterranean areas. *BULLETIN OF ENTOMOLOGICAL RESEARCH*, *105*(1), 60–69. <https://doi.org/10.1017/S0007485314000753>

Loskutov, I. G., Blinova, E. V., Gavrilova, O. P., & Gagkaeva, T. Y. (2017). The valuable characteristics and resistance to Fusarium disease of oat genotypes. *Russian Journal of Genetics: Applied Research*, *7*(3), 290–298. Scopus. <https://doi.org/10.1134/S2079059717030108>

Loureiro, E. D. S., & Moino Jr, A. (2006). Pathogenicity of hyphomycet fungi to aphids Aphis gossypii glover and Myzus Persicae (Sulzer) (Hemiptera: Aphididae). *Neotropical Entomology*, *35*(5), 660–665. Scopus. <https://doi.org/10.1590/S1519-566X2006000500014>

Lu, Y. S., Chen, M. C., & Chien, C. C. (2011). Application of bio-vinegar from rice husk on prevention and cure of plant blight. *Taiwanese Journal of Agricultural Chemistry and Food Science*, *49*(4), 159–166. Scopus.

Lubanga, U. K., Karungi, J., Kyamanywa, S., & Ekbom, B. (2012). Assessing the potential of trap cropping in the management of different insect taxa on white cabbage. *INTERNATIONAL JOURNAL OF TROPICAL INSECT SCIENCE*, *32*(4), 218–223. <https://doi.org/10.1017/S1742758412000306>

Lundgren, J. G., & Weber, D. C. (2010). Changes in digestive rate of a predatory beetle over its larval stage: Implications for dietary breadth. *JOURNAL OF INSECT PHYSIOLOGY*, *56*(4), 431–437. <https://doi.org/10.1016/j.jinsphys.2009.11.020>

Luo, F. F., Tang, G. R., Hong, S., Gong, T. Y., Xin, X. F., & Wang, C. S. (2023). Promotion of Arabidopsis immune responses by a rhizosphere fungus via supply of pipecolic acid to plants and selective augment of phytoalexins. *SCIENCE CHINA-LIFE SCIENCES*, *66*(5), 1119–1133. <https://doi.org/10.1007/s11427-022-2238-8>

Luo, K., Ouellet, T., Zhao, H. Y., Wang, X. K., & Kang, Z. S. (2021). Wheat-Fusarium graminearum Interactions Under Sitobion avenae Influence: From Nutrients and Hormone Signals. *FRONTIERS IN NUTRITION*, *8*. <https://doi.org/10.3389/fnut.2021.703293>

Luo, Z., Zhang, Y., Jin, K., Ma, J., Wang, X., & Pei, Y. (2009). Construction of Beauveria bassiana T-DNA insertion mutant collections and identification of thermosensitive and osmosensitive mutants. *Wei Sheng Wu Xue Bao = Acta Microbiologica Sinica*, *49*(10), 1301–1305. Scopus.

Ma, G., Xia, C., Chen, D., Deng, H., Wang, X., Shao, Y., & Sun, R. (2023). Insecticide screening to control Myzus persicae in tobacco fields and their safety evaluation for Rhynocoris fuscipes. *Tobacco Science and Technology*, *56*(8), 20–27. Scopus. <https://doi.org/10.16135/j.issn1002-0861.2022.0779>

Ma, L., Chen, F., Wang, W., Xu, L., & Lu, Z. Q. (2020). Identification of two clip domain serine proteases involved in the pea aphid’s defense against bacterial and fungal infection. *INSECT SCIENCE*, *27*(4), 735–744. <https://doi.org/10.1111/1744-7917.12673>

Ma, R. Z., Reese, J. C., Black Iv, W. C., & Bramel-Cox, P. (1998). Chlorophyll Loss in a Greenbug-Susceptible Sorghum Due to Pectinases and Pectin Fragments. *Journal of the Kansas Entomological Society*, *71*(1), 51–60. Scopus.

Ma, Z. W., Geudens, N., Kieu, N. P., Sinnaeve, D., Ongena, M., Martins, J. C., & Höfte, M. (2016). Biosynthesis, Chemical Structure, and Structure-Activity Relationship of Orfamide Lipopeptides Produced by Pseudomonas protegens and Related Species. *FRONTIERS IN MICROBIOLOGY*, *7*. <https://doi.org/10.3389/fmicb.2016.00382>

Mackay, W. A., George, S. W., McKenney, C., Sloan, J. J., Cabrera, R. I., Reinert, J. A., Colbaugh, P., Lockett, L., & Crow, W. (2008). Performance of garden roses in north-central Texas under minimal input conditions. *HORTTECHNOLOGY*, *18*(3), 417–422. <https://doi.org/10.21273/HORTTECH.18.3.417>

Macuphe, N., Oguntibeju, O. O., & Nchu, F. (2021). Evaluating the Endophytic Activities of Beauveria bassiana on the Physiology, Growth, and Antioxidant Activities of Extracts of Lettuce (Lactuca sativa L.). *PLANTS-BASEL*, *10*(6). <https://doi.org/10.3390/plants10061178>

Madge, D. S., & Buxton, J. H. (1976). EVALUATION OF EUROPEAN EARWIG (FORFICULA-AURICULARIA) AS A PREDATOR OF DAMSON-HOP APHID (PHORODON-HUMULI) .2. CHOICE OF PREY. *ENTOMOLOGIA EXPERIMENTALIS ET APPLICATA*, *19*(3), 221–226. <https://doi.org/10.1111/j.1570-7458.1976.tb02601.x>

Magyar, D., Strażyński, P., Grewling, Ł., Pashley, C. H., Satchwell, J., Bobvos, J., & Ladányi, M. (2023). The contribution of aphids (Aphidoidea) to atmospheric concentrations of Alternaria and Cladosporium spores. *Aerobiologia*, *39*(3), 345–361. Scopus. <https://doi.org/10.1007/s10453-023-09797-4>

Mahendiran, G., Lal, S., & Sharma, O. C. (2022). Pests and Their Management on Temperate Fruits: (Apple, Pear, Peach, Apricot, Cherry, Persimmon, Walnut, Olive, Kiwifruit and Strawberry). In *Trends in Horticultural Entomology* (pp. 891–941). Springer Nature; Scopus. <https://doi.org/10.1007/978-981-19-0343-4_36>

Mahillon, M., Groux, R., Bussereau, F., Brodard, J., Debonneville, C., Demal, S., Kellenberger, I., Peter, M., Steinger, T., & Schumpp, O. (2022). Virus Yellows and Syndrome “Basses Richesses” in Western Switzerland: A Dramatic 2020 Season Calls for Urgent Control Measures. *Pathogens*, *11*(8). Scopus. <https://doi.org/10.3390/pathogens11080885>

Mahmood, H. R., D’Asheesh, T. A., Kareem, M. A., & Alkhuzaie, M. M. (2020). New bio-nano technique for controlling the green peach aphid Myzus persicae. *Biochemical and Cellular Archives*, *20*, 4217–4221. Scopus.

Mahmood, Z., Steenberg, T., Mahmood, K., Labouriau, R., & Kristensen, M. (2019). Endophytic Beauveria bassiana in maize affects survival and fecundity of the aphid Sitobion avenae. *BIOLOGICAL CONTROL*, *137*. <https://doi.org/10.1016/j.biocontrol.2019.104017>

Mahmoud, G. A. E., Zohri, A. N. A., Kamal-Eldin, N. A., & Abdelhamid, N. M. R. (2023). APPLICATION OF ASPERGILLUS ORYZAE ASU44 (OL314732) AND THEIR KOJIC ACID AS PESTICIDES AGAINST COTTON APHID, APHIS GOSSYPII. *Bulletin of Pharmaceutical Sciences. Assiut*, *46*(1), 63–82. Scopus. <https://doi.org/10.21608/BFSA.2023.300763>

Maketon, M., Chakanya, N., Prem-udomkit, K., & Maketon, C. (2013). Interaction Between Entomopathogenic Fungi and Some Aphid Species in Thailand. *Gesunde Pflanzen*, *65*(3), 93–105. Scopus. <https://doi.org/10.1007/s10343-013-0302-9>

Makhmadjanov, S. P., Tokhetova, L. A., Daurenbek, N. M., Tagaev, A. M., & Kostakov, A. K. (2023). COTTON ADVANCED LINES ASSESSMENT IN THE SOUTHERN REGION OF KAZAKHSTAN. *SABRAO JOURNAL OF BREEDING AND GENETICS*, *55*(2), 279–290. <https://doi.org/10.54910/sabrao2023.55.2.1>

Malagnoux, L., Capowiez, Y., & Rault, M. (2015). Impact of insecticide exposure on the predation activity of the European earwig Forficula auricularia. *ENVIRONMENTAL SCIENCE AND POLLUTION RESEARCH*, *22*(18), 14116–14126. <https://doi.org/10.1007/s11356-015-4520-9>

Malinga, L. N., & Laing, M. D. (2021). Efficacy of three biopesticides against cotton pests under field conditions in South Africa. *CROP PROTECTION*, *145*. <https://doi.org/10.1016/j.cropro.2021.105578>

Manfrino, R. G., Gutierrez, A. C., Rueda Páramo, M. E., Salto, C. E., & López Lastra, C. C. (2016). Prevalence of entomophthoralean fungi (Entomophthoromycota) of aphids in relation to developmental stages. *Pest Management Science*, *72*(8), 1566–1571. Scopus. <https://doi.org/10.1002/ps.4188>

Manfrino, R. G., Gutiérrez, A. C., Steinkraus, D. C., Salto, C. E., & López Lastra, C. C. (2014). Prevalence of entomophthoralean fungi (Entomophthoromycota) of aphids (Hemiptera: Aphididae) on solanaceous crops in Argentina. *Journal of Invertebrate Pathology*, *121*, 21–23. Scopus. <https://doi.org/10.1016/j.jip.2014.06.003>

Manfrino, R. G., Hatting, J. L., Humber, R., Salto, C. E., & Lopez Lastra, C. C. (2014). Natural occurrence of entomophthoroid fungi (Entomophthoromycota) of aphids (Hemiptera: Aphididae) on cereal crops in Argentina. *Annals of Applied Biology*, *164*(1), 151–158. Scopus. <https://doi.org/10.1111/aab.12089>

Manfrino, R. G., & Rocca, M. (2024). Susceptibility of Orius insidiosus to Beauveria bassiana, Akanthomyces muscarius, and Cordyceps fumosorosea and their effects on predator behavior. *ENTOMOLOGIA EXPERIMENTALIS ET APPLICATA*. <https://doi.org/10.1111/eea.13493>

Manoussopoulos, Y., Mantzoukas, S., Lagogiannis, I., Goudoudaki, S., & Kambouris, M. (2019). Effects of Three Strawberry Entomopathogenic Fungi on the Prefeeding Behavior of the Aphid Myzus persicae. *JOURNAL OF INSECT BEHAVIOR*, *32*(2), 99–108. <https://doi.org/10.1007/s10905-019-09709-w>

Mantzoukas, S., Daskalaki, E., Kitsiou, F., Papantzikos, V., Servis, D., Bitivanos, S., Patakioutas, G., & Eliopoulos, P. A. (2022). Dual Action of Beauveria bassiana (Hypocreales; Cordycipitaceae) Endophytic Stains as Biocontrol Agents against Sucking Pests and Plant Growth Biostimulants on Melon and Strawberry Field Plants. *MICROORGANISMS*, *10*(11). <https://doi.org/10.3390/microorganisms10112306>

Mantzoukas, S., & Lagogiannis, I. (2019). Endophytic Colonization of Pepper (Capsicum annum) Controls Aphids (Myzus persicae Sulzer). *APPLIED SCIENCES-BASEL*, *9*(11). <https://doi.org/10.3390/app9112239>

Mantzoukas, S., Papantzikos, V., Katsogiannou, S., Papanikou, A., Koukidis, C., Servis, D., Eliopoulos, P., & Patakioutas, G. (2023). Biostimulant and Bioinsecticidal Effect of Coating Cotton Seeds with Endophytic Beauveria bassiana in Semi-Field Conditions. *MICROORGANISMS*, *11*(8). <https://doi.org/10.3390/microorganisms11082050>

Mantzoukas, S., Tamez-Guerra, P., Zavala-Garcia, F., Lagogiannis, I., & Ek-Ramos, M. J. (2022). Entomopathogenic fungi tested in planta on pepper and in field on sorghum, to control commercially important species of aphids. *World Journal of Microbiology and Biotechnology*, *38*(5). Scopus. <https://doi.org/10.1007/s11274-022-03268-7>

Markó, V., Blommers, L. H. M., Bogya, S., & Helsen, H. (2008). Kaolin particle films suppress many apple pests, disrupt natural enemies and promote woolly apple aphid. *JOURNAL OF APPLIED ENTOMOLOGY*, *132*(1), 26–35. <https://doi.org/10.1111/j.1439-0418.2007.01233.x>

Marshall, A. T., & Beers, E. H. (2022). Exclusion netting affects apple arthropod communities. *BIOLOGICAL CONTROL*, *165*. <https://doi.org/10.1016/j.biocontrol.2021.104805>

Marti, O. G., & Olson, D. M. (2007). Effect of tillage on cotton aphids (Homoptera: Aphididae), pathogenic fungi, and predators in south central Georgia cotton fields. *Journal of Entomological Science*, *42*(3), 354–367. Scopus. <https://doi.org/10.18474/0749-8004-42.3.354>

Martín, A., Martín, L. M., Cabrera, A., Ramirez, M. C., Gimenez, M. J., Rubiales, D., Hernandez, P., & Ballesteros, J. (1998). *The potential of Hordeum chilense in breeding Triticeae species* (WOS:000085946900046). 377–386.

Martin, M. J., Li, Y. M., Ma, L., Feng, Y., & Lu, Z. Q. (2021). Fight or Flight? Alternative Defense of the Pea Aphids, Acyrthosiphon pisum on Different Host Plants. *INSECTS*, *12*(7). <https://doi.org/10.3390/insects12070614>

Martínez, J. A., Jowkar, M. M., Obando-Ulloa, J. M., Varó, P., Moreno, E., Monforte, A. J., & Fernández-Trujillo, J. P. (2009). Uncommon disorders and decay in near-isogenic lines of melon and reference cultivars. *HORTICULTURA BRASILEIRA*, *27*(4), 505–514. <https://doi.org/10.1590/S0102-05362009000400017>

Martins, I. C. F., Silva, R. J., Alencar, J., Silva, K. P., Cividanes, F. J., Duarte, R. T., Agostini, L. T., & Polanczyk, R. A. (2014). Interactions Between the Entomopathogenic Fungi Beauveria bassiana (Ascomycota: Hypocreales) and the Aphid Parasitoid Diaeretiella rapae (Hymenoptera: Braconidae) on Myzus persicae (Hemiptera: Aphididae). *JOURNAL OF ECONOMIC ENTOMOLOGY*, *107*(3), 933–938. <https://doi.org/10.1603/EC13542>

Martins, S. J., Medeiros, F. H. V., Andrade, R. C., Nunez, A. M. P., Souza, B., Moino, A., & Filgueiras, C. C. (2016). Dual role of milk on aphid and powdery mildew control in kale. *SCIENTIA HORTICULTURAE*, *203*, 126–130. <https://doi.org/10.1016/j.scienta.2016.03.023>

Martinuz, A., Schouten, A., Menjivar, R. D., & Sikora, R. A. (2012a). Effectiveness of systemic resistance toward Aphis gossypii (Hom., Aphididae) as induced by combined applications of the endophytes Fusarium oxysporum Fo162 and Rhizobium etli G12. *Biological Control*, *62*(3), 206–212. Scopus. <https://doi.org/10.1016/j.biocontrol.2012.05.006>

Martinuz, A., Schouten, A., Menjivar, R. D., & Sikora, R. A. (2012b). Effectiveness of systemic resistance toward Aphis gossypii (Hom., Aphididae) as induced by combined applications of the endophytes Fusarium oxysporum Fo162 and Rhizobium etli G12. *BIOLOGICAL CONTROL*, *62*(3). <https://doi.org/10.1016/j.biocontrol.2012.05.006>

Masheva, S., Yankova, V., Markova, D., Boteva, H., & Dincheva, T. (2011). DISEASES AND PESTS ON TRIBULUS TERRESTRIS L.- WILD GROWING PLANT AND SEMI-CROP. *BIOTECHNOLOGY & BIOTECHNOLOGICAL EQUIPMENT*, *25*(2), 2391–2393. <https://doi.org/10.5504/BBEQ.2011.0042>

Masuda, T., & Kikuchi, O. (1992). Pathogenicity of Verticillium lecanii Isolates to Whitefly and Aphids. *Japanese Journal of Applied Entomology and Zoology*, *36*(4), 239–245. Scopus. <https://doi.org/10.1303/jjaez.36.239>

Mathulwe, L. L., Malan, A. P., & Stokwe, N. F. (2023a). Infection of insects and persistence of Metarhizium (Hypocreales: Clavicipitaceae) species on apple bark. *AFRICAN ENTOMOLOGY*, *31*. <https://doi.org/10.17159/2254-8854/2023/a13944>

Mathulwe, L. L., Malan, A. P., & Stokwe, N. F. (2023b). The occurrence of entomopathogenic fungi in apple orchards and their biocontrol potential against Eriosoma lanigerum. *AFRICAN ENTOMOLOGY*, *31*. <https://doi.org/10.17159/2254-8854/2023/a13728>

Mayntz, D., & Toft, S. (2000). Effect of nutrient balance on tolerance to low quality prey in a wolf spider (Araneae: Lycosidae). *EKOLOGIA-BRATISLAVA*, *19*, 153–158.

McCarville, M. T., O’Neal, M., Tylka, G. L., Kanobe, C., & Macintosh, G. C. (2012). A nematode, fungus, and aphid interact via a shared host plant: Implications for soybean management. *Entomologia Experimentalis et Applicata*, *143*(1), 55–66. Scopus. <https://doi.org/10.1111/j.1570-7458.2012.01227.x>

McEwen, J., Bardner, R., Briggs, G. G., Bromilow, R. H., Cockbain, A. J., Day, J. M., Fletcher, K. E., Legg, B. J., Roughley, R. J., Salt, G. A., Simpson, H. R., Webb, R. M., Witty, J. F., & Yeoman, D. P. (1981). THE EFFECTS OF IRRIGATION, NITROGEN-FERTILIZER AND THE CONTROL OF PESTS AND PATHOGENS ON SPRING-SOWN FIELD BEANS (VICIA-FABA L) AND RESIDUAL EFFECTS ON 2 FOLLOWING WINTER-WHEAT CROPS. *JOURNAL OF AGRICULTURAL SCIENCE*, *96*(FEB), 129–150. <https://doi.org/10.1017/S0021859600031944>

McEwen, J., Cockbain, A. J., Fletcher, K. E., Salt, G. A., Wall, C., Whitehead, A. G., & Yeoman, D. P. (1979). EFFECTS OF ALDICARB, TRIAZOPHOS AND BENOMYL PLUS ZINEB ON THE INCIDENCE OF PESTS AND PATHOGENS AND ON THE YIELDS AND NITROGEN UPTAKES OF LEAFLESS PEAS (PISUM-SATIVUM-L). *JOURNAL OF AGRICULTURAL SCIENCE*, *93*(DEC), 687–692. <https://doi.org/10.1017/S0021859600039101>

McIntyre, J. L., Dodds, J. A., & Hare, J. D. (1981). EFFECTS OF LOCALIZED INFECTIONS OF NICOTIANA-TABACUM BY TOBACCO MOSAIC-VIRUS ON SYSTEMIC RESISTANCE AGAINST DIVERSE PATHOGENS AND AN INSECT. *PHYTOPATHOLOGY*, *71*(3), 297–301. <https://doi.org/10.1094/Phyto-71-297>
[truncated: 4,280,419 more chars]
